# Glial responses during epileptogenesis in *Mus musculus* point to potential therapeutic targets

**DOI:** 10.1371/journal.pone.0201742

**Published:** 2018-08-16

**Authors:** Georgia Kalozoumi, Olga Kel-Margoulis, Elizabeth Vafiadaki, David Greenberg, Hélène Bernard, Hermona Soreq, Antoine Depaulis, Despina Sanoudou

**Affiliations:** 1 Clinical Genomics and Pharmacogenomics Unit, 4^th^ Department of Internal Medicine, Attikon Hospital, Medical School, National and Kapodistrian University of Athens, Athens, Greece; 2 geneXplain GmbH, Wolfenbüttel, Germany; 3 Molecular Biology Division, Biomedical Research Foundation of the Academy of Athens, Athens, Greece; 4 The Edmond and Lily Safra Center for Brain Sciences, The Hebrew University of Jerusalem, Jerusalem, Israel; 5 INSERM, Grenoble, France; 6 Univ. Grenoble Alpes, Grenoble Institut des Neurosciences, Grenoble, France; 7 CHU de Grenoble, Hôpital Michallon, Grenoble, France; Nathan S Kline Institute, UNITED STATES

## Abstract

The Mesio-Temporal Lobe Epilepsy syndrome is the most common form of intractable epilepsy. It is characterized by recurrence of focal seizures and is often associated with hippocampal sclerosis and drug resistance. We aimed to characterize the molecular changes occurring during the initial stages of epileptogenesis in search of new therapeutic targets for Mesio-Temporal Lobe Epilepsy. We used a mouse model obtained by intra-hippocampal microinjection of kainate and performed hippocampal whole genome expression analysis at 6h, 12h and 24h post-injection, followed by multilevel bioinformatics analysis. We report significant changes in immune and inflammatory responses, neuronal network reorganization processes and glial functions, predominantly initiated during *status epilepticus* at 12h and persistent after the end of *status epilepticus* at 24h post-kainate. Upstream regulator analysis highlighted Cyba, Cybb and Vim as central regulators of multiple overexpressed genes implicated in glial responses at 24h. *In silico* microRNA analysis indicated that *miR-9*, *miR-19b*, *miR-129*, and *miR-223* may regulate the expression of glial-associated genes at 24h. Our data support the hypothesis that glial-mediated inflammatory response holds a key role during epileptogenesis, and that microglial cells may participate in the initial process of epileptogenesis through increased ROS production via the NOX complex.

## Introduction

Most brain diseases are a result of a progressive cascade of molecular and cellular events that go by undetected over a period of several years, until a pathological phenotype becomes clinically detectable [1]. Although the genetic background may influence the occurrence of these pathologies, it is often one or more initial insults earlier in life that serve as triggers [2]. A major challenge in the treatment of these conditions is the identification of the primary steps of the process that could serve as an effective therapeutic target [3]. Mesio-Temporal Lobe Epilepsy (MTLE), the most common form of intractable epilepsies, is a prime example of such a progressive disease. Numerous reports describe that all MTLE patients experience one or several “trigger” insults such as complex febrile seizures, head trauma, intracerebral infections and/or ischemic episodes during early childhood [4,5]. These initial insults trigger a cascade of molecular events over a period of several years, during which no clinical symptoms are observed (“silent period”), which ultimately leads to recurrent focal seizures, the main symptoms, generated in the mesio-temporal limbic structures [2,6]. The latency period of MTLE epileptogenesis, appears to span the time window from the initial insult to the occurrence of the first spontaneous seizures [3]. Studies on animal models have demonstrated that this latency period is a time of intense functional and morphological reorganization including neurodegeneration, neurogenesis, gliosis, axonal damage or sprouting, dendritic plasticity, blood–brain barrier (BBB) damage, recruitment of inflammatory cells into brain tissue, reorganisation of the extracellular matrix and reconstruction of the cyto-architecture of individual neuronal cells [3,7].

Recent advances in systems biology, high-throughput technologies and sophisticated data mining approaches have emerged as a powerful way to discover new therapeutic targets in progressive brain diseases. In the case of MTLE, microarray and RNA-seq approaches are increasingly used for the study of representative animal models. Yet no effective therapeutic targets or accurate biomarkers for epileptogenesis have arisen to date [8–14]. Limiting factors associated with this slow progress are likely associated with the choice of specific animal models, time-points post *status epilepticus* (SE), brain structures, experimental design and data analysis [15]. For example, many of these studies have used *systemic* injections of an excitotoxin, kainate (KA), a glutamate analogue, to induce a SE for several hours in the rat [16]. This approach results in bilateral lesions in different brain structures and leads mainly to *generalized* convulsive seizures, two features that differ from what is observed in MTLE patients and may be confounding [17]. In addition, in the vast majority of studies, brain samples were collected while the animals were experiencing seizures, either during the SE or, later, during (i) epileptogenesis when spikes and bursts of spikes occur without behavioral symptoms and/or (ii) the chronic phase when seizures occur regularly, raising the concern that most changes observed could be the consequence of seizure occurrence [13]. Importantly, most analyses have largely aimed at describing the molecular pathway changes without extending the investigation to the upstream regulators (e.g. transcription factors) orchestrating these modifications that could provide the basis for the discovery of new therapeutic targets.

In the present study, our objective was to determine the early mechanisms of epileptogenesis so as to effectively block disease progression at the earliest possible stage. In the clinical setting, it is critical to have treatments available that will act during, or at the end, of the SE, before the drastic morphological and functional reorganization that is associated with epileptogenesis occurs [3]. We chose the established intra-hippocampal KA-injection induced mouse model of MTLE, since it reproduces most of the histopathological and electrophysiological features of human MTLE [18,19]. In this model, spontaneous focal epileptic seizures associated with mild behavioral expression develop progressively in the hippocampus, leading to a stereotypical EEG pattern at 16–18 days post KA-injection. This phenotype remains stable for several months, with only occasional propagation of the seizures to the cortex, similarly to MTLE patients [20–22]. In addition, cell loss in the CA1, CA3 and hilus areas, as well as astrocyte proliferation and granule cell dispersion are observed in the injected hippocampus, findings highly reminiscent of the hippocampal sclerosis observed in most MTLE patients [18,19,23–27]. Furthermore, in this model, differential effects of antiepileptic drugs on focal seizures were reported, with weak response to several classical drugs, as in MTLE patients [19,28,29]. Altogether, this mouse model meets many clinicians’ requirements for modelling human MTLE [17,22]. Using this model, a microarray-based study indicated important gene expression changes at 6h after KA injection, i.e. during the SE, as well as 14 days later, i.e., when recurrent focal seizures are recorded in the hippocampus [13]. However, during these two time-points, we have described important epileptiform activities [19,21,30] that likely lead to important gene regulations *per se*, which are not necessarily involved in epileptogenesis. Here, we rather focused on the epileptogenic mechanisms activated during SE (6 and 12h) and once SE activity is over (24h), before isolated or bursts of spikes occur [21,30]. Through a comprehensive transcriptomics and multi-level bioinformatics approach, comprising functional classification signaling network prediction and *in silico* microRNA analysis, we were able to depict the early molecular pathway changes implicated in epileptogenesis, and identify key regulatory molecules that deserve further investigation of their potential as therapeutic targets.

## Results

### The status epilepticus is completed at 24h post KA

**Fig 1:**
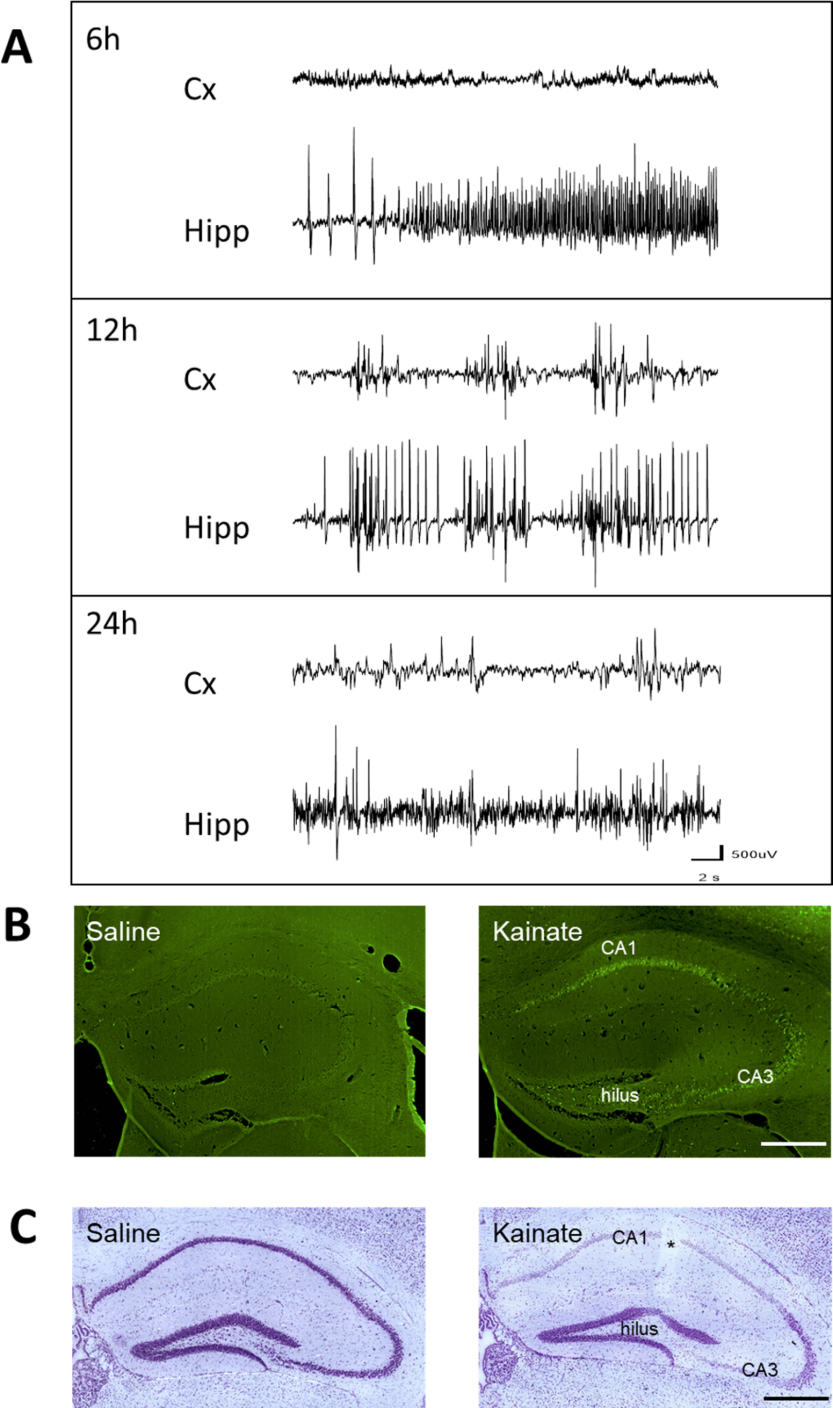
Electroencephalographic and histological consequences of kainate injection into the dorsal hippocampus in mice. A. EEG recordings performed at 6, 12 and 24h post-KA (n = 6), showing examples of EEG patterns recorded during the focal status epilepticus in the cortex (Cx) and injected hippocampus (Hipp). At 6 h post-KA there was no epileptiform activity in the cortex, whereas a discharge of spike and poly-spikes was observed in the hippocampus. At 12h, bursts of spikes were regularly observed in the hippocampus and ipsilateral cortex, whereas only occasional spikes were recorded in either the cortex or the hippocampus between 22 and 24h. B. Fluoro-jade B labeling 24h post-injection in saline-injected mouse (left) and KA-injected mouse (right), indicating the presence of injured cells in CA1, CA3 and hilus area. C. Nissl staining 24h post-injection in saline-injected mouse (left) and KA-injected mouse (right) indicating that most cells were pyknotic in CA1, CA3 and hilus area. Bar = 500 μm. * = track of the injection cannula.

All KA-injected animals included in this study (n = 9/time-point) displayed the behavioral characteristics of SE (mild asymmetric clonic movements of the forelimbs, clonic deviations of the head, rotations and/or prolonged periods of immobilization, occasional bilateral clonic seizures of the forelimbs). These behavioral features have been shown to be associated with hippocampal and cortical EEG spikes and bursts of spikes, 3-4h post KA and for up to 18h [19,30]. In our current study this was confirmed in the 6 animals implanted with cortical and hippocampal electrodes, where we first observed isolated spikes, bursts of spikes and polyspikes in the hippocampus during the 2-3h that follow KA injection, i.e. when animals recovered from anesthesia. We then recorded hippocampal bursts of spikes and polyspikes every minute for up to 18h-20h in all animals (Fig 1A). This activity was interrupted about every hour by hippocampal discharges of spikes and polyspikes lasting up to 20 s, which were associated with mild clonic movements of the forelimbs. We observed bursts of spikes in the cortex that first occurred 4-6h post-KA and then regularly in concomitance with hippocampal bursts, in line with our previous reports [21].When we recorded these animals between 22h and 24h post KA, we observed only occasional spikes in either the cortex or the hippocampus (Fig 1A). This indicated that the SE was completed at that time, as reported previously [21]. These data confirm that, in this model, the focal SE is terminated at 24h post-KA.

### Advanced stage of cell loss at 24h post KA

Fluoro-jade B staining was performed in 6 animals sacrificed 24h post KA injection, as a measure of the ongoing neuronal insult. A strong signal was observed across the CA1, CA3 and hilus regions of the injected hippocampi (Fig 1B), as compared to saline-injected animals or to the contralateral side of the hippocampus, where only scattered cells were stained (Fig 1C). Furthermore, Nissl staining, widely used for the study of neuronal morphology and pathology, revealed an almost total loss of neurons in the CA1, CA3 and hilus areas at 24h (Fig 1D), as compared to saline-injected animals (Fig 1E), with only pyknotic cells being observed in these regions, in accordance with previous reports in this model [18,30]. These data confirm that the phase of increased cell death is largely completed in the KA-injected hippocampus 24h post KA.

### Kainate injection triggers significant transcript changes in the mouse hippocampus during the first 24 hours

To determine if KA treatment had an effect on hippocampal gene expression we performed correlation coefficient analysis across all saline- and KA-injected microarray datasets. Primary evidence confirming the KA effect on gene expression involved the reduced inter-group (KA-injected vs. saline-injected mice) correlation coefficient values (average 94.9%) compared to intra-group (KA- or saline-injected mice) values (average 98.1%). KA therefore, appears to induce several changes in hippocampal gene expression, across all the investigated time-points, with a more prominent effect towards 24h post-injection (inter-group correlation coefficients of 6h KA vs saline samples was 96.2% and 24h KA vs saline samples 93.5%).

**Fig 2:**
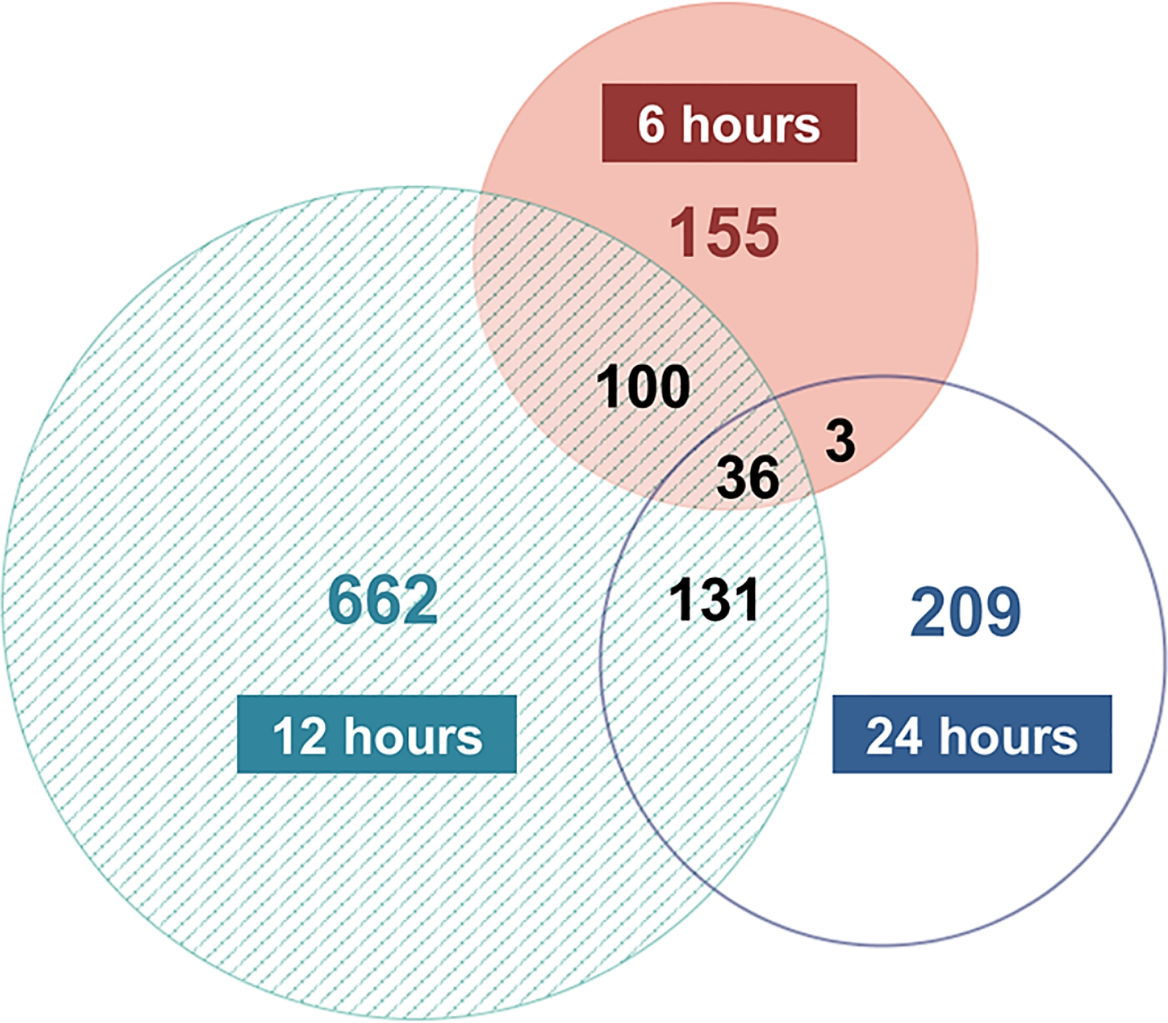
Comparison of the significant transcript changes across 6, 12 and 24h post KA injection. Area-proportional Venn diagram with the number of significantly changed probe sets at each post-injection time point. A total of 54 animals were used (n = 9/time-point/treatment) for the microarray analysis, and SAM was applied for the analysis of KA- versus saline-injected hippocampi at each time point interrogated (thresholds: fold change ≥ |2|, FDR = 0%).

To determine the specific gene expression changes induced to the hippocampus by KA exposure, SAM was applied for the analysis of KA- versus saline-injected hippocampi at each post-injection time point. The number of highly and significantly changed probe sets per time point ranged from 294 at 6h to 929 at 12h and 379 at 24h post-injection (S1–S3 Tables). The fold changes of individual probe sets ranged from 69.3 (neuronal PAS domain protein) to -20.35 (arachidonate 12-lipoxygenase, 12R type). Interestingly, at 24h, most of the significant changes involved probe set over-expression (~77%), in contrast with the earlier time points, during which the levels of over- and under-expressed probe sets were approximately equal (~55% genes were under-expressed). Cross comparison of the three probe set lists revealed that 155, 662, and 209 probe sets were uniquely changed at 6, 12 and 24, whereas 36 probe sets were consistently changed across all time-points (Fig 2).

Representative transcripts that were significantly changed in at least one of the time points were further evaluated by qRT-PCR. The results were consistent with our microarray findings, with the latter fold estimates being overall more modest, a likely reflection of the distinct nature of the two technical approaches and in agreement with the literature [31,32] (S4 Table).

### Molecular mechanisms implicated in the mouse hippocampus response to kainate at 6, 12 and 24 hours

To identify the significantly changed biological processes, molecular functions, and cellular components for each time point of the present study, we analysed the significantly changed transcripts using the geneXplain “Mapping to ontologies (TRANSPATH®)” workflow. All different levels of GO terms were assessed, so as to determine the most detailed and comprehensive level for the specific set of data. Level 6 was selected for the biological process (S2–S4 Figs and S5–S7 Tables) and molecular function analysis (S8–S10 Tables), whereas level 5 was best suited for the cellular component categorization (S11–S13 Tables).

The GO Biological Process analysis revealed that the end of KA-induced SE is followed by several distinct significant changes. Specifically, ~35% of the significantly changed biological processes are unique at 24h, i.e. they are not observed during SE (i.e., at 6 and 12h) in our study. These include categories related to glial cells (e.g. “regulation of glial cell proliferation”, “astrocyte development”), neuronal network reorganization (e.g. “neuron projection development”, “axon regeneration”), immune and inflammatory response (e.g. “regulation of phagocytosis”, “positive regulation of leukocyte activation”), and lipid metabolism (e.g. “regulation of lipid metabolic process”, “regulation of lipid transport”) (S8 Table).

Uniquely changed biological processes were also observed during both time points of SE investigated. Specifically, at 6h post KA injection ~18% of the enriched groups including e.g. “protein de-phosphorylation” and “regulation of cellular ketone metabolic process” (S5 Table) were not observed at other time points. Conversely, at 12h ~43% of uniquely changed biological processes that were observed, consist of responses to endogenous stimuli (“cellular response to cytokine stimulus”, “response to hydrogen peroxide”) and ions (e.g. “response to calcium ion”), neuronal transmission (e.g. “positive regulation of transmission of nerve impulse”), immune cell differentiation (“regulation of myeloid leukocyte differentiation) and migration (regulation of leukocyte migration), apoptosis (e.g. “regulation of apoptotic signalling pathway”), and carbohydrate metabolism (e.g. regulation of carbohydrate metabolic process) (S6 Table).

Interestingly, the biological processes that changed only during SE (i.e. 6 and 12h) comprise ~23% and ~6.5% of the enriched categories at 6 and 12h, respectively. These groups include “transcription, DNA-dependent”, “small GTPase mediated signal transduction” and “regulation of synaptic transmission” (S5 and S6 Tables). Since they do not remain significantly changed once SE is completed at 24h, they are likely to be associated with the epileptiform activity and/or the neuronal cell death observed during SE.

Importantly, the comparison of the SE at 12h and the end of SE (24h) revealed a considerable overlap. Specifically, ~44% of the changes in biological processes that were enriched at 24h appear to be initiated at 12h. These include groups related to cell migration (e.g. “regulation of cell migration”, “positive regulation of cell motility”), cytoskeletal organisation (e.g. “cytoskeleton organisation”), reactive oxygen species (e.g. “regulation of reactive oxygen species metabolic process”) and inflammation and immune response (e.g. “positive regulation of immune response”, “regulation of inflammatory response”) (S6 and S7 Tables). The persistence of these changes, despite the absence of epileptiform activity at 24h, suggests that these gene regulation events are triggered during SE but are unlikely to depend upon the occurrence of epileptiform activity.

The consistent changes across all time points of the study represent approximately ~55%, ~15% and ~19% of the enriched biological processes observed at 6, 12 and 24h (S2–S4 Figs and S5–S7 Tables). These categories are associated with essential cell functions such as gene/protein expression/modification, cell proliferation/cell death, cell communication/signal transduction and ion homeostasis and transport, and also include groups related to response to external stimulus, stress and wounding. The number of significantly changed genes in each of these categories appeared to peak at 12h post KA injection, when the total number of significantly changed genes is almost tripled in comparison with 6h and 24h. These observations imply that these changes may be associated with the response to KA in this model (cell death, epileptiform events) and are less likely to be implicated in epileptogenesis *per se*.

The GO Molecular Function analysis revealed that ~30%, ~43% and 47% of the enriched categories at 6, 12, and 24h respectively, were uniquely changed (S8–S10 Tables). Specifically, the end of SE (24h) was associated with distinct changes in molecular functions that included ligand-receptor binding (e.g. “insulin-like growth factor I binding”, “interleukin-1 receptor binding”) and enzyme activity (e.g. “cysteine-type peptidase activity”, “aldo-keto reductase (NADP) activity”) (S10 Table). The unique changes observed at 6h included “phosphatase activity” and “transcription regulatory region sequence-specific DNA binding” (S8 Table), whilst the enrichment of molecular functions such as “nuclear hormone receptor binding”, “chemokine receptor binding”, “neuropeptide Y receptor activity” and “ionotropic glutamate receptor activity” was limited to the 12h time-point (S9 Table). Groups that were found to be enriched only during the two time points of SE include “transition metal ion binding” and “transcription regulatory region DNA binding” (S8 and S9 Tables). Significantly changed categories observed for the first time at 12h, that remain enriched after the end of SE at 24h, include groups related to ion homeostasis (e.g. “calcium ion binding” “gated channel activity”), and kinase activity (“protein kinase activity”, “protein kinase binding”) (S9 and S10 Tables). Of note, the only molecular functions that changed across all time points are “gated channel activity” and “heparin binding”.

The GO Cellular Component analysis showed that the distinct changes following the end of epileptiform activity at 24h include cytoskeletal components (e.g. “cytoskeleton”, “microtubule organizing center”) and specific parts of the plasma membrane (e.g. “extrinsic to plasma membrane”, “internal side of plasma membrane”) (S13 Table). The uniquely changed groups at 12h included other specific subcellular membrane components (e.g. “neuron projection membrane” “intrinsic to endoplasmic reticulum membrane”), sub-nuclear regions (e.g. “nucleolus” “chromatin”), and “tight junction”, amongst others (S12 Table). Conversely, at 6h only one uniquely changed group was identified, i.e. “nucleoplasm part”, whilst the two points of SE shared only “nuclear lumen” (S11 and S12 Tables). The 12h time-point shared more changed groups with the end of SE (24h), such as “cytosol”, neuron-specific topologies (e.g. “dendrite”, “axon”, “neuron spine”) and groups related to vesicles (e.g. “cytoplasmic membrane-bounded vesicle lumen”) (S12 and S13 Tables). Notably, the cellular component “nucleus” was identified as the most enriched category across all time points of the study, which is in line with the extensive changes related to regulation of gene expression observed over the course of the first 24h post KA injection.

### In silico prediction of upstream regulators modulating the 24h transcript changes

A master regulator analysis was performed using the geneXplain software to identify hierarchically high regulatory molecules of significantly upregulated genes, which could potentially serve as candidate therapeutic targets for early disease stages, upon successful translation of the findings in human MTLE. For this purpose, we focused on the analysis of significantly changed genes at 24h, hypothesizing that these reflect molecular pathways triggered during SE (i.e. before 24h) and remain high, or reach significant upregulation levels in the period that follows SE and precedes the occurrence of the first spikes, when no epileptiform events are yet observed. Therefore, this analysis was performed for the 24h time point considering only the upregulated transcripts and enabled the prediction of central regulators likely orchestrating the observed gene overexpression at this time-point.

For the 291 overexpressed transcripts at 24h, 79 different master regulators were identified (Score >0.2, Z-Score >1), each one regulating a range of 16 to 96 overexpressed genes (data not shown). Importantly, 26 master regulators were themselves overexpressed: 25 showed increased expression at 24h (*Calca*, *S1pr3*, *Spp1*, *Lgals1*, *Cd9*, *Gcg*, *Itgav*, *Vim*, *Nfkbia*, *Hmgn1*, *Il11*, *Tgm2*, *Lif*, *Icam1*, *Cybb*, *Rnd3*, *Nek6*, *Eif2ak2*, *Nedd9*, *Yes1*, *Pak3*, *Birc3*, *Rfwd2*, *Myd88 and Capn2*), 11 of which were already overexpressed at 12 hours (*S1pr3*, *Spp1*, *Vim*, *Nfkbia*, *Il11*, *Tgm2*, *Icam1*, *Cybb*, *Rnd3*, *Nedd9 and Birc3*), whilst one (*Cyba*) was only overexpressed at 12 hours (S14 Table). The finding of predicted master regulators being themselves significantly overexpressed at 12h and 24h is not only a valuable proof of concept, but importantly, enables the mapping of the extended molecular mechanisms regulating the observed changes.

**Fig 3:**
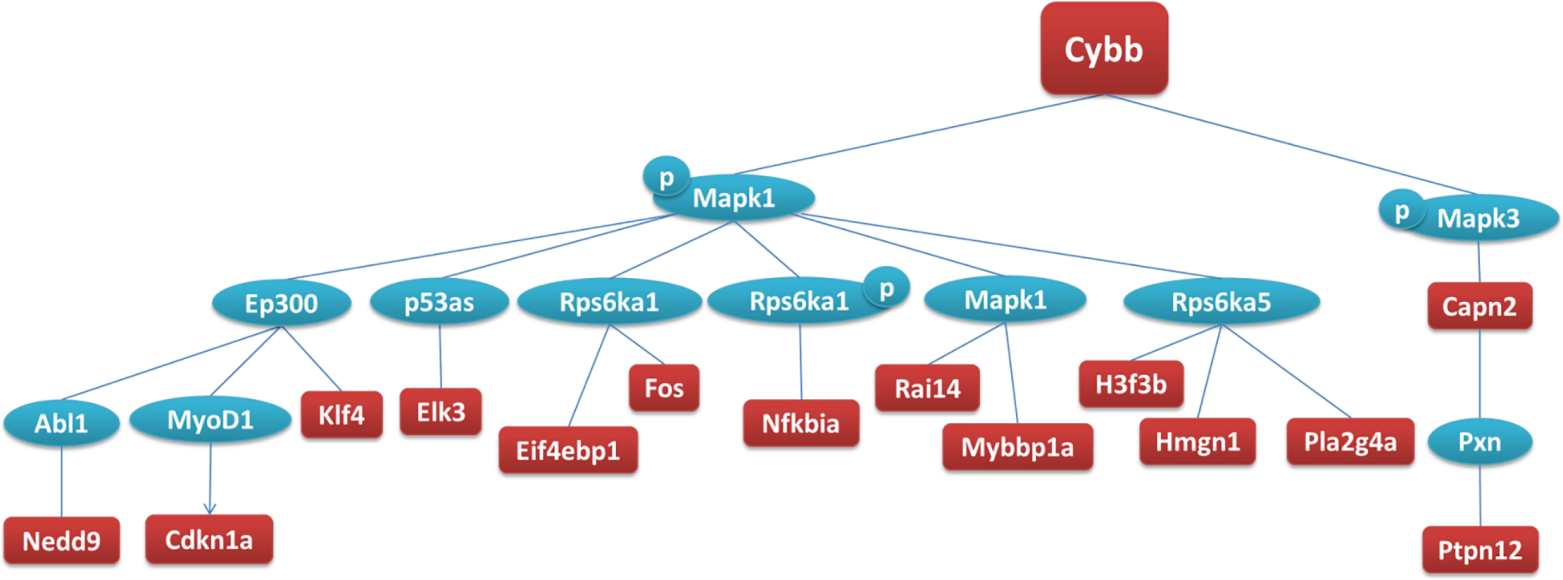
Schematic representation of the network of downstream molecules regulated by Cybb at 24h. The analysis was performed using the “Master regulators in networks (TRANSPATH®)” workflow (geneXplain 2.2 web edition) (thresholds: Score >0.2 and Z-Score >1), for all the significantly changed transcripts detected by microarrays in KA- versus saline-injected hippocampi at 24h (SAM analysis, thresholds: fold change > |2|, FDR = 0%, n = 9/time-point/treatment). Red: genes overexpressed at 24h, blue: genes not significantly changed, p: phosphorylation.

Specifically, each of the 26 overexpressed master regulators regulates a total of 16 to 67 overexpressed genes at 24h. Three of the 26 overexpressed master regulators were of particular interest because of their biological role in the CNS and the genes they regulate. These 3 genes, namely: i) cytochrome b, alpha subunit (*Cyba* or *p22-phox*) which codes for a component of the NADPH oxidase (*NOX*); ii) cytochrome b, beta subunit (*Cybb* or *p91-phox* or *Nox2*), which forms a heterodimer with *p22-phox* (Fig 3); and, ii) vimentin (*Vim*), which codes for a cytoskeletal protein (Table 1), may be of interest for consideration as potential therapeutic targets and merit further investigation.

**Table 1:** Selected, predicted, statistically significant upstream regulators of the transcripts significantly changed at 24h.

Upstream regulator	Fold Change at 12h	Fold Change at 24h	Z-Score	Number of significantly changed genes at 24h	Significantly changed genes at 24h
***Cyba***	2.23	n/c	1.81	42	*Bag2*, *Bgn*, *Birc3*, *C3ar1*, *Calca*, *Capn2*, *Casp8*, *Ccl9*, *Cdkn1a*, *Cybb*, *Eif2ak2*, *Eif4ebp1*, *Elk3*, *Eng*, *Fcgr2b*, *Fos*, *Fosl2*, *Gfap*, *H3f3a*, *H3f3b*, *Hmgn1*, *Hspb1*, *Icam1*, *Il1rn*, *Itgav*, *Klf4*, *Lif*, *Msn*, *Mybbp1a*, *Nedd9*, *Nfkbia*, *Pdcd6ip*, *Pla2g4a*, *Ptpn12*, *Rai14*, *Rnd3*, *Tgm2*, *Timp1*, *Tubb6*, *Vim*, *Wwtr1*, *Yes1*
***Cybb***	2.92	4.75	4.52	15	*Capn2*, *Cdkn1a*, *Cybb*, *Eif4ebp1*, *Elk3*, *Fos*, *H3f3b*, *Hmgn1*, *Klf4*, *Mybbp1a*, *Nedd9*, *Nfkbia*, *Pla2g4a*, *Ptpn12*, *Rai14*
***Lgals1***	n/c	2.73	6.58	20	*Capn2*, *Cdkn1a*, *Eif4ebp1*, *Elk3*, *Fcgr2b*, *Fos*, *Gfap*, *H3f3b*, *Hmgn1*, *Klf4*, *Lgals1*, *Mybbp1a*, *Nedd9*, *Nfkbia*, *Pdcd6ip*, *Pla2g4a*, *Ptpn12*, *Rai14*, *Vim*, *Yes1*
***Nedd9***	4.16	4.70	3.83	41	*Arf6*, *Arpp21*, *Birc3*, *C3ar1*, *Capn2*, *Casp8*, *Ccl9*, *Cdkn1a*, *Cybb*, *Eif2ak2*, *Eif4ebp1*, *Elk3*, *Eng*, *Fcgr2b*, *Fos*, *Gfap*, *H3f3b*, *Hbegf*, *Hmgn1*, *Hspb1*, *Icam1*, *Il1rn*, *Itgav*, *Klf4*, *Msn*, *Mybbp1a*, *Nedd9*, *Nfkbia*, *Pdcd6ip*, *Pla2g4a*, *Plek*, *Ptpn12*, *Rai14*, *Rnd3*, *S100a10*, *Timp1*, *Tnc*, *Tubb6*, *Vim*, *Wwtr1*, *Yes1*
***Vim***	2.79	3.65	5.47	15	*Capn2*, *Cdkn1a*, *Eif4ebp1*, *Elk3*, *Fos*, *H3f3b*, *Hmgn1*, *Klf4*, *Mybbp1a*, *Nedd9*, *Nfkbia*, *Pla2g4a*, *Ptpn12*, *Rai14*, *Vim*

The analysis was performed using the “Master regulators in networks (TRANSPATH®)” workflow (geneXplain 2.2 web edition), for all the significantly changed transcripts at 24h, applying the thresholds Score >0.2 and Z-Score >1. The predicted upstream regulators were filtered to exclude those that did not present with statistically significant overexpression themselves at 12h or 24h, and those that were overexpressed at 6h. The number and symbol of their respective significantly overexpressed downstream gene targets at 24h is included. (n/c: not changed).

### MicroRNAs associated with the 24h gene expression changes

The search for central regulators of multiple changed genes at 24h was extended to the post-transcriptional level, aiming to explore the potential of microRNAs as potential therapeutic targets for epileptogenesis. Both upregulated and downregulated transcripts were considered in this analysis. Although the overexpression of a target mRNA would be typically accompanied by decreased levels of the regulatory microRNA [33], a microRNA could also promote their target’s mRNA expression, e.g. via binding to the 5’UTR of their target mRNA and accelerating mRNA interaction with polysomes [34].

The retrieval of experimentally validated microRNA-mRNA interactions via miRWalk identified 98 microRNAs that target at least two transcripts each, and in total 163 out of the 379 significantly changed transcripts (↑124 genes, S15 Table; ↓39 genes, S16 Table). Among them, *miR-466* presented with the largest number of experimentally validated target transcripts (i.e., 22 over-expressed and 10 under-expressed genes) at 24h post KA. In-depth literature mining identified those microRNAs that targeted >10 significantly changed genes, so as to pinpoint central regulatory microRNAs that may be implicated in epileptogenic processes. Four microRNAs emerged as likely modulators, namely *miR-9*, *miR-19b*, *miR-129* and *miR-223*, because of their established role in the CNS and/or epilepsy, as well as the biological role of their target genes in microglia, astroglia and/or the NOX and ROS pathways, as elaborated in the Discussion.

## Discussion

### The end of the focal status epilepticus is a critical period for targeting epileptogenesis

In the present study, we used focal *SE* induced by intra-hippocampal injection of KA in the mouse to model an initial insult that could trigger the progressive evolution towards chronic MTLE. In addition to its close similarities with key features of MTLE associated with hippocampal sclerosis [17], this model offers several methodological advantages for performing high throughput analyses such as global gene expression screening. Specifically, when induced in C57/Bl6 mice, the focal SE is reproducible across animals and rarely leads to death, unlike systemic injection of KA or pilocarpine [19]. Consequently, no subsequent injection of benzodiazepine is required to interrupt the SE, which could have interfered with the gene regulation, as suggested by previous work in the same model [35]. Whether the anesthetic used (Chloral hydrate) interfered with gene regulation remains possible. However, all data collected in KA-injected animals were compared to data collected in saline-injected mice under the same anesthesia conditions. Furthermore, the duration of the focal SE is rather stereotyped in this model, with a termination around 15-18h, as demonstrated by previous reports [19,21,24,30] and as confirmed by the EEG recordings at 22–24 h post-KA in the present study. Following intra-hippocampal injection of KA, cell death is triggered rapidly, is restricted to the dorsal hippocampus, around the injection site, and is highly reproducible across animals [30]. Our data, in line with previous reports, suggest that most cell death was completed by 24h post KA and that epileptiform activities observed during epileptogenesis [21] have not yet begun. Therefore, the 24h time-point appears as an important pivot where processes involved in the initiation of epileptogenesis can be studied without interfering with processes associated with the recurrence of epileptiform activities observed during SE or epileptogenesis. In addition, this time-window appears as most relevant for potential therapeutic targeting in the clinic, i.e., during patient hospitalization, generally a few hours after a SE [36].

Our analysis spanned the first 24h post KA, in an effort to gain insight on the molecular mechanisms that are affected by the initial insult and remain changed after the end of SE. We suggest that these changes reflect the epileptogenic processes that take place in this model. Specifically, we aimed at identifying the pathways and the timeline involved in the early phase of epileptogenesis, which focuses on the affected biological pathways and processes rather than on the specific transcripts involved, and addressed the time points when those appeared to be modified. Further, we sought the predictive power of the network by using advanced bioinformatics tools. In that aspect, what we advocate is the type of network and cell types to which future therapeutic agents could be addressed.

Interestingly, at the first time point of our study, 6h post KA injection, amongst the top enriched biological functions that are associated with regulation of transcription, we observed multiple transcription factor modifications that overlap with the findings of previous studies investigating the acute phase post KA administration [9,13]. Importantly, most of the overlapping genes (i.e. *Fos*, *Fosb*, *Jun*, *Junb*, *Nr4a1*, *Nr4a2*, *Nr4a3*) are associated with the immediate response to neuro-excitatory stimuli in the CNS, such as KA [37], electrical stimulation [38–40] or exercise [41], and are widely known as immediate and early genes (IEGs). The list of significantly changed IEGs extends to the upregulated *Bdnf*, *Arc* and *Homer1* that have been associated with synaptic plasticity regulation [42,43]. Notably, the expression changes in these IEGs persist at 12h post injection, but the effect ceases after the end of epileptiform activities, at 24h, suggesting that their expression is mostly associated with the over-excitation provoked by KA in the hippocampus during the SE. These observations are in agreement with our recent proteomic findings, showing downregulation of immediate response proteins associated with synaptic plasticity at 24h post injection [44].

This transient gene expression pattern peaks at 12h post injection, where the number of significantly changed genes almost triples in comparison to the 6 and 24h time points. A representative example of this observation is the downregulation of many gene categories that are associated with seizure manifestation and often targeted by anticonvulsants, including glutamate receptors (*Gria1*, *Gria3*, *Grin1*, *Grm1*, *Grm5*, *Grm8*), GABA receptors (*Gabrg2*, *Gabra5*), voltage-gated potassium channels (*Kcna2*, *Kcnab1*, *Kcnc1*, *Kcnc2*, *Kcnh3*, *Kcns2*, *Kcnq5*, *Kcnq3*), sodium channels (*Scn1a*, *Scn2a1*, *Scn2b*, *Scn3b*, *Scn8a*, *Nalcn*) and calcium channels (*Cacna1b*, *Cacna 1d*, *Cacna1h*). Importantly, only a few of these genes remain under-expressed at 24h.

These observations suggest that a large number of the transcript changes at 6 and 12h post injection is associated with the intense hyperactivity associated with the focal SE and may only be indirectly associated with epileptogenesis. Consequently, an emphasis was given on the analysis of the 24h changes, which should be better suited for the search of new therapeutic targets in MTLE.

### Glial responses during epileptogenesis point at novel central regulators of epileptogenesis

Our extensive bioinformatics analysis of the 24h time-point data, in combination with in depth data mining and cross-disciplinary expert input, led to the delineation of a subset of significantly changed biological functions which may play a critical role in MTLE epileptogenesis. Accordingly, the prominent changes we observed in genes orchestrating the immune and inflammatory responses in the CNS, in combination with experimental evidence on increased glial proliferation in experimental and human MTLE, directed our search for central regulators of epileptogenesis.

### Immune and inflammatory responses mediated by glia

Focusing on the evidence for immune and inflammatory processes at 24h, we observed multiple enriched GO biological processes associated with these functions. The enriched categories range from more inclusive ones such as “positive regulation of immune response” and “regulation of inflammatory response” (S7 Table), to more specific functions such as “regulation of phagocytosis”, “positive regulation of chemotaxis” “positive regulation of leukocyte migration”, “regulation of interleukin-1 production”, and extend to the level of molecular pathways, e.g. “cytokine-mediated signaling pathway” “pattern recognition receptor signaling pathway” and “Toll signaling pathway”. These data complement and extend the only previous study at 24h, on a rat model of electrically induced SE, where evidence for the manifestation of stress response, along with a prominent immune and inflammatory response were noted [12]. The observed similarities between this model and our KA-induced mouse model suggest the primary association of these mechanisms with the MTLE phenotype, independently of the epileptogenic trigger.

Notably, neuronal responses in the CNS are usually accompanied by the activation of astrocytes and microglial cells. Consistently with this association, we detected marked overexpression of genes that serve as markers of microglial activation (*Tspo* f = 4.18) [45,46] or reactive astrocytes (*Gfap* f = 3.9, *Vim* f = 3.65) [47–49]. Importantly, these changes are consistent with our immunohistochemistry and proteomics data showing upregulation of these proteins at 24h (Gfap) and 3 days post injection (Gfap, Vim), [44]. In addition, pro-inflammatory genes previously shown to be expressed by glial cells during inflammatory response, such as *Pla2g4a* (a.k.a. cPLA2, f = 2.65) [50–52] and *Klf4* (f = 4.05) [53,54], were significantly upregulated, along with *Il11* (f = 12.44), which acts to suppress inflammation [55,56]. The molecular players of hippocampal inflammation described herein, allow the delineation of the biological basis of the previously described massive proliferation of astroglial cells over the sclerotic hippocampus in the same mouse model [18,24,30], in agreement with findings in MTLE patients [57,58],[59]. In particular, we had previously shown in the MTLE mouse, using immunohistochemistry, that such astroglia proliferation occurred early after the initial KA-induced insult [24,26] and then extend radially-orientated fibers, forming a dense glial scaffold in close contact with the dentate granule cells [24]. Furthermore, we demonstrated that this glial reorganization develops progressively in the dentate gyrus within the same time-frame as epilepsy development in this structure [21].

These inflammatory processes are thought to play a central role in epileptogenesis and MTLE progression [60–63], according to studies in both animal models [30,63,64] and human epileptic tissue [65]. In particular, exacerbation of inflammatory response is observed after the initial insult in experimental models of epilepsy with *SE*, suggesting that uncontrolled inflammatory response may contribute to epileptogenesis [62,65]. Moreover, proliferation and/or morphological changes of microglia have been reported in the hippocampus during the first 4–5 days after SE induced by systemic KA or pilocarpine in mice and rats [24,66–69]. This was associated with the expression of several pro-inflammatory cytokines (IL-1b, IL-6, TNFβ) and the important cell loss that occurs during the first days that follow SE [30,61,70,71]. In reverse, depletion of microglia reduced seizure susceptibility and epileptogenesis in SE-induced models [72,73]. Therefore, the molecular players emerging from the present study, and especially an understanding of the upstream regulators and microRNAs orchestrating these processes, in combination with follow-up translational research could unveil promising new therapeutic targets.

**Fig 4:**
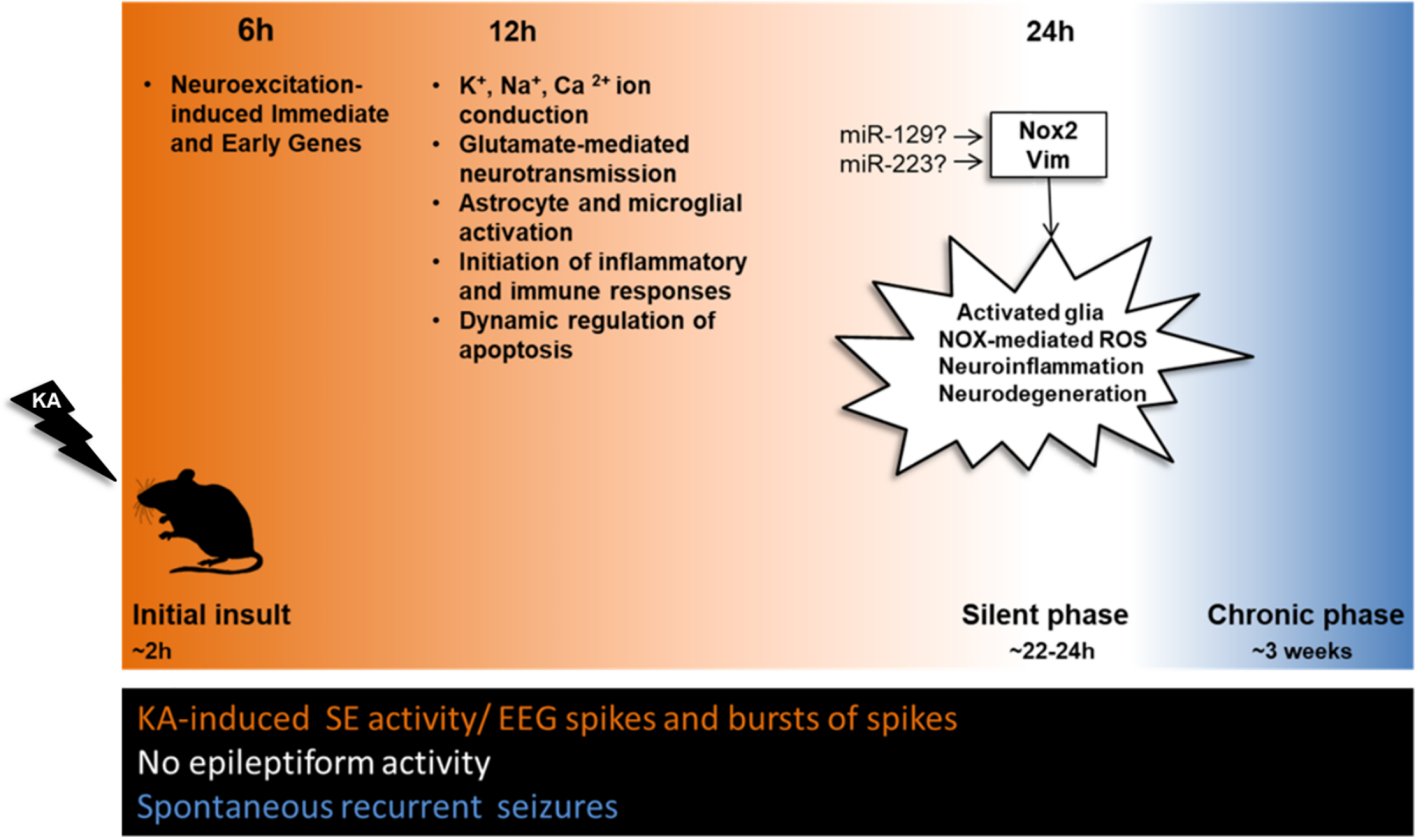
Summary diagram of the key biological changes detected during epileptogenesis. At 6h multiple neuroexcitation-induced immediate and early response genes are significantly changed. At 12h specific ion conduction, neurotransmission, astrocyte/microglial activation, inflammation and apoptosis related mechanisms are significantly altered. Importantly, at 24h, the silent phase of epileptogenesis, *Nox2* (*Cybb*) and *Vim* are predicted to act as upstream regulators of key biological processes ultimately leading to activated glia, NOX-mediated ROS, neuroinflammation and neurodegeneration. The presence or absence of KA-induced epileptiform activity is denoted with orange and white color, respectively. The spontaneous epileptiform activity is marked with blue color.

Towards this direction and building on our findings at 12h, when several of these processes (including: astrocyte and microglial activation, inflammatory response regulation and leukocyte recruitment and extravasation) appear to be initiated, we performed an extensive upstream (master) regulator analysis. Among the master regulators identified, of particular interest was *Vim*, as it was both significantly overexpressed itself in our data (f = 3.65), and a predicted master regulator of 15 overexpressed genes at 24h (Fig 4 and Table 1). According to the literature, some of these 15 genes (e.g., *Capn2*, *Nfkbia*, *Pla2g4a*, *Ptpn12*) may play a critical role in MTLE epileptogenesis through the modulation of inflammatory processes mediated by astrocytes. Consistently with these findings, an increase of Vim immunohistolabeling was observed in elongated astrocytes of the dentate gyrus in the KA-MTLE mouse model [24]. Furthermore, recent studies conducted in *Vim KO* mice described an essential role for Vim in microglia activation induced by both LPS treatment *in vitro* and cerebral ischemia *in vivo*, whilst *Vim* deletion also conferred neuroprotection via the inhibition of the detrimental inflammatory effects of microglia [74]. Importantly, Vim’s downstream targets include the overexpressed *Klf4* gene, which is associated with microglia activation [53], thus supporting a role for Vim in facilitating glial-mediated inflammation in KA-MTLE. Overall, the present results are in full agreement with a critical role of astroglial and microglial changes in the progressive development of MTLE, and suggest that Vim could play a central role in this process.

Our *in-silico* microRNA analysis complements these findings, with the identification of microRNAs that may act as central regulators of glial responses at 24h post KA. MicroRNAs have been implicated in a number of molecular processes impaired in epilepsy and are being explored as potential biomarkers and therapeutic targets [75]. In the present analysis, four microRNAs emerged as experimentally validated regulators of a multitude of the KA-MTLE significantly changed genes in our 24h experiments, including genes involved in glial responses. In specific, *miR-223* emerged as an experimentally validated regulator of *Vim*, and has been previously reported to be overexpressed in the rat hippocampus, at 24h post electrically-induced SE (Fig 4) [76]. *miR-19b*, is an experimentally validated regulator of genes implicated in phagocytosis signaling (e.g. *Pros1*, *Tub*) [77,78], a microglial function significantly altered in epilepsy. Importantly, it was recently highlighted as a potential biomarker of human TLE [79]. In addition, *miR-9*, one of the most abundant microRNAs in the rodent hippocampus, has been shown to target *Gfap*, a key component in astrocyte reactivity [80,81]. *miR-129* emerged as another likely regulator of mRNA changes at 24h post KA. To date, *miR-129* has been shown to increase seizure susceptibility in chronic epilepsy, specifically at 21 days post KA in the rat, by repressing the expression of the potassium channel *Kcna1* (*Kv1*.*1*) [82]. Our analysis suggests that its involvement may start earlier and impact multiple molecular pathways in experimental epilepsy.

### The NOX and ROS pathways play a critical role in MTLE epileptogenesis

Beyond regulation of transcript levels by microRNAs, our master regulator analysis identified two subunits of the membrane-bound NADPH oxidase (NOX) hexamer complex, namely *Cybb* and *Cyba*, as upstream regulators of a multitude of overexpressed genes at 24h post KA injection (Fig 3 and Table 1). Importantly, both *Cybb* (↑12h, f = 2.92; 24h, f = 4.75) (Fig 4) and *Cyba* (↑12h, f = 2.31) were also significantly overexpressed themselves in our study. The NOX complex is located in the membranes of phagosomes and endoplasmic reticulum, as well as in the plasma membrane, and catalyzes the production of reactive oxygen species (ROS, e.g. O_2_^**-.**^, H_2_O_2_) [83]. The increased production of ROS via the NOX complex of activated microglial cells [84–90] and neurons [87,91–93] has been shown to induce the transcription of pro-apoptotic genes, as well as the activation of apoptotic mechanisms. In the pilocarpine model of MTLE, NOX activation triggered the upregulation of NMDA receptors [94] and led to neurodegeneration [95,96]. The central role of NOX in these processes was confirmed with the use of a NOX complex inhibitor, which appeared to limit them [96,97]. Excitation via glutamate receptors has also been shown to induce ROS production via NOX [85,87,93,98,99], whilst KA-induced seizures in rats trigger NOX complex activation and increased O_2_^**-.**^, in parallel to microglial activation [100]. Recently, ROS were found to be elevated in neurons in a model of SE induced by perforant pathway stimulation, and to contribute to cell death whereas a NADPH oxidase inhibitor showed protective effects [101]. These findings are in line with a recent study showing high levels of lipid peroxidation by-products, in combination with increased protein levels of two other NOX subunits, *Ncf1 (p47phox)* and *Ncf2 (p67phox)*, in neurons in hippocampal samples resected from drug-resistant MTLE patients [102]. Consequently, the role of NOX enzymes appears to be central in acquired epilepsy (Fig 4). However, their involvement in established MTLE may be different than the early stage of epileptogenesis and remains to be explored.

Interestingly, NOX may also be subject to post-transcriptional regulation by microRNAs, according to our *in silico* microRNA analysis. Specifically, *miR-129* has been shown to interact with the ROS-producing NOX subunit *Cybb*, whilst it also targets the inflammation suppressor *Il11*, both of which were found to be overexpressed at 24h post KA in this study (Fig 4).

### Conclusions

Our findings support the hypothesis that microglial cells participate in the neurodegeneration observed in the KA-induced MTLE mouse model through increased ROS production via the NOX complex. Since neurodegeneration is mostly completed by 24h in this model, it is likely that NOX activation in microglial cells, but also neurons, orchestrate other mechanisms that lead to epileptogenesis, consistently with the multiple significantly changed downstream targets of *Cyba* and *Cybb*. The spatiotemporal pattern of the seizure-induced alterations in the NOX complex activity within the epilepsy focus is yet to be determined. Consequently, the NOX complex merits further investigation to fully characterize its role in the establishment of MTLE, and determine its potential in combating the disease.

## Materials and methods

### Animals

All animal procedures were carried out in accordance with the rules of the European Committee Council Directive 2010/63/EU. The detailed protocol was first submitted to our local ethical committee (Comité Local Grenoble Institut Neurosciences, agreement # C2EA-04), then, upon approval, to the French Ministry of Education and Research via the APAFIS website. It was approved under the registration n° 01 389 02.

Experiments were conducted on adult C57Bl/6j male mice (Janvier, Le-Genest-St-Isle, France), 8–10 weeks of age, and 20-25g of weight (n = 72, S1 Fig). Following surgery, they were housed in individual cages with food and water *ad libitum* and kept in a 12h light-dark cycle (room temperature = 22 ± 1°C). All efforts were made to minimize animal suffering and reduce the number of animals used in each series of experiments.

### Intra-hippocampal injection

Mice were stereotactically injected with either 50 nL of a 20mM solution of KA (Sigma-Aldrich Chimie, St Quentin Fallavier, France) in NaCl 0.9% (i.e., 1 nmol) or saline (50 nL of NaCl 0.9%) unilaterally in the dorsal hippocampus, while under chloral hydrate (400 mg/kg, i.p.) generalized anesthesia, [anteroposterior (AP), -2; mediolateral (ML), -1.5; dorso-ventral (DV), -2] with bregma as the reference [103], as previously described [19,21,24,30]. The use of chloral hydrate was preferred since anesthetics that are antagonists of the glutamate receptors (e.g., ketamine) or that act as an antiepileptic (e.g., barbiturates) could block or reduce the initial *status epilepticus* or alter the development of epileptogenesis [35]. Similarly, the use of halothane or isoflurane was avoided because of their effects on the blood brain barrier [104] and on the development of epileptogenesis [105]. Once they recovered from anesthesia (i.e., about 2h), the mice were visually inspected for up to 8h, to determine their behavior during the KA-induced SE. Indeed, following intra-hippocampal KA injection, the animals displayed mild asymmetric clonic movements of the forelimbs, clonic deviations of the head, rotations and/or prolonged periods of immobilization and, in most cases, bilateral clonic seizures of the forelimbs associated with rearing [19,21,24,30]. Only mice showing this characteristic behavioral pattern of SE following KA injection were included in the subsequent stages of the analysis.

### Electroencephalographic recordings of status epilepticus

After KA injection, a group of six mice were implanted with a bipolar electrode in the injected hippocampus, at the same coordinates as the injection site, and with three monopolar electrodes over the left and right fronto-parietal cortices and the cerebellum (reference electrode) as previously described [19,21,24]. Electroencephalographic (EEG) activity was recorded using a digital acquisition computer-based system (Coherence, Deltamed, France; sampling rate 256 Hz), in freely moving mice placed in Plexiglas test cages contained in a Faraday cage. Recordings were initiated immediately after recovery from anesthesia (about 2h post KA injection) and for 8h, to verify that SE was initiated. Recordings (2h) were then performed at 12h and then at 22h post KA.

### Histological assessment of cell loss

KA- or saline-injected mice (n = 6/group) were sacrificed at 24 h post KA injection with a lethal dose of pentobarbital (Nembutal, 100 mg/kg, i.p.) and perfused with PFA 4%. Their brains were removed, cryo-protected in 30% sucrose overnight and frozen in iso-pentane (-40°C). Twenty-μm thick sections were collected and mounted on 2% gelatine coated slides and air dried at 50 °C for 30 min. The slides were first immersed in a solution containing 1% sodium hydroxide in 80% ethanol (20 ml of 5% NaOH added to 80 ml absolute ethanol) for 3 min. This was followed by 2 minute incubations in 70% ethanol and distilled water. The slides were transferred to a 0.06% potassium permanganate solution for 10 min and rinsed in distilled water. After 20 min in a 0.0004% Fluoro-jade B staining solution, the slides were rinsed for 3x1 min in distilled water, dried at 50°C for 5 min and then cleared by immersion in xylene for 1 min before “cover-slipping” with p-xylene-pyrimidinium bromide. Brain slices were examined for fluorescence using a Leica DMI 6000 fluorescent microscope and the METAMORPH® image analysis software. Adjacent sections were stained with cresyl violet (Sigma-Aldrich Chimie, Saint-Quentin Fallavier, France), and examined to localize each recording and injection site with reference to the mouse brain atlas [103]. Only data from animals with (i) correct location of the hippocampal electrode, (ii) correct histological features in the injected hippocampus [19] were used.

### Gene expression microarray experiments

For the gene expression studies, KA- or saline-injected mice were decapitated at either 6h, 12h or 24h post-treatment (n = 9/time-point/treatment, i.e., total = 54 samples). Their brain was rapidly removed from the skull at 4°C, the injected hippocampus was dissected, and the anterior part was snap frozen in liquid nitrogen. The entire procedure was completed within 2 minutes.

Total RNA was extracted from dissected hippocampi using the Trizol extraction protocol as previously described [31]. The RNA obtained from the hippocampus of 9 different mice for each time point (6, 12, 24 hours post treatment) and treatment (KA or saline injection), was used for the preparation of 3 different RNA pools for each set of conditions (time-point/treatment), with each pool comprising the RNA of 3 different mice. Consequently, three biological replicate pools were used for each set of conditions (time-point/treatment). Each RNA pool was processed according to the recommended Affymetrix protocols for target preparation, and hybridization to Affymetrix Mouse Genome 430 2.0 GeneChips (each containing 45,100 probe sets, representative of 39,000 transcripts and variants from over 34,000 well characterized mouse genes).

### Identification of significant gene expression changes

The 18 data sets, originating from the 54 hippocampal samples, were first processed by the Affymetrix GeneChip Operating Software (GCOS), and signal values (reflecting expression levels) and “present/absent” calls (an Affymetrix computed measure representing confidence in gene expression presence) were computed for each probe set. The raw microarray data generated during the current study are available at the Gene Expression Omnibus (GEO) repository web site under the series number GSE88992.

For correlation coefficient analysis, r-values for overall, un-normalized gene expression signatures were calculated for all 18 data set pairwise comparisons. All data sets were then normalized to a slope of 1 with a reference data set (Sal 6hr-1). For filtering purposes, all the probe sets in each dataset were assessed for present/absent calls and only those with ≥3 present calls across the datasets of each time point were included in further analysis [106].

Significance Analysis of Microarrays (SAM) was used to identify significant fold changes between KA and saline injected hippocampi, for each post-injection time-point, as described [107]. A two-class unpaired data analysis was performed using a Δ threshold of 4.6–4.8 [the “Δ” parameter enables the user to examine the effect of the false-positive rate in determining significance] and a fold threshold of 2 [where “fold” is calculated as (average expression in KA injected specimens)/ (average expression in saline injected specimens)]. Probe sets were considered significantly changed in KA compared with saline samples if they were selected at a median false discovery rate (FDR) cut-off of 0% [108].

### Bioinformatical functional enrichment analysis

In order to discover functional enrichment amongst the differentially expressed probe sets of each time point, the geneXplain platform (geneXplain 2.2 web edition) [http://www.genexplain.com/] was utilized. The probe sets representing the significantly changed transcripts (fold change ≥ |2|, FDR = 0%) were subjected to the “Mapping to ontologies (TRANSPATH®)” workflow, which aids the classification of an input gene set to ontologies, whilst identifying terms that are overrepresented in the dataset. The functional classification was done according to the Gene Ontology (GO) categories “Biological process”, “Cellular component”, and “Molecular function”. All different levels of GO terms were assessed.

### Bioinformatical upstream regulator analysis

The significantly overexpressed transcripts (fold ≥2, 0% FDR) at the 24h time point were subjected to the “Master regulators in networks (TRANSPATH®)” workflow (geneXplain 2.2 web edition) to identify common signaling molecules upstream of the significantly changed genes [109,110]. This process perceives each upstream reaction as one distinct “step”, and results in the identification of “key nodes” or regulatory molecules that are up to ten (10) steps upstream of the input molecules. The regulatory molecules, namely “master regulators” or upstream regulators, are common for multiple genes of the input list. For each master regulator, the FDR, Specificity Score and Z-Score were calculated. FDR represents the probability for a given master regulator to occupy the observed or higher ranks by random chance, and the Z-Score is used as a measure of statistical significance (calculated as the deviation of the observed rank for each key node from the expected rank by random chance, divided by the standard deviation). The Specificity Score, referred to as “Score”, expresses the ratio of true positives to false positives for a given key node. Master regulators with FDR <0.05 were considered statistically significant, and the cutoff thresholds applied were Score >0.2 and Z-Score >1.

### MicroRNA *in silico* analysis

In order to identify microRNAs that may act as regulators of gene expression, the “Validated module” of miRWalk 2.0 database was utilized (Dweep, 2015). In detail, the official Gene symbols corresponding to the significantly changed transcripts (fold change ≥ |2|, FDR = 0%) at 24h post KA were submitted to the “Validated gene-microRNA interaction information retrieval system”, which performs elaborate data mining across the PubMed scientific literature, to retrieve experimentally validated microRNA-mRNA interactions for the input set of transcripts.

### Real-time quantitative RT-PCR analysis

Total RNA was quantitatively and qualitatively assessed using the Nanodrop ND-100 (Thermo, Wilmington, DE) and 1% agarose gel electrophoresis, respectively. All samples had a 28S:18S ribosomal RNA ratio equal to 2:1. The cDNA synthesis (Promega, Madison, WI) involved 0.4 μg RNA samples in 20 μl reactions. Duplicate real-time quantitative reverse transcriptase qRT–PCR reactions were run on the ABI prism 7900HT, using the SYBR green master mix (Applied biosystems, Foster City, CA) ROX as a passive reference dye for signal normalization across the plate and *Gapdh* as a reference control transcript. The annealing temperature was 60°C for all primers (S17 Table). Serial dilution of samples served to evaluate primer efficiency and determine the cDNA concentration that yields linear changes. RT controls verified lack of genomic DNA.

Fold change was calculated by the formula $\frac{2^{cyc:sal-cyc:ka}taget\_gene}{2^{cyc:sal-cyc:ka}GAPDH}$, where the cycle of amplification of the non-treated sample (saline) was subtracted from the treated sample (kainate) as an exponent of two for each transcript in question, divided by the same for the reference control transcript.

All qRT-PCR experiments were performed by scientists “blind” to the microarray results.

### Statistical analysis

Following real-time quantitative RT-PCR, relative quantitation was calculated with the Δ Δ CT method, using Gapdh as the reference gene for normalization purposes and the ΔCT values were then expressed relative to the respective group of samples that served as baseline for each set of comparisons. The significance threshold was set at p<0.05.

## Supporting information

S1 Fig Design of experiments conducted using the KA-MTLE mouse model. A total of 72 animals were used: 39 injected with KA, and 33 injected with saline as controls. EEG recordings were performed in 6 KA-injected animals. Hippocampal samples from 27 KA- and 27 saline-injected animals (3 different pools [biological replicates] of 3 mice/treatment/time point; 3 time points: 6h, 12h, 24h post injection) were used for microarray and qRT-PCR analyses. Hippocampal samples from 6 KA- and 6 saline-injected animals were used for histological assessment of cell loss.
(TIF)

S2 Fig GO biological processes (level 6) at 6h post KA injection. Level 6 GO Biological Process terms via REVIGO for all the significantly changed transcripts detected by microarrays in KA- versus saline-injected hippocampi at 6h post injection (SAM analysis, thresholds: fold change > |2|, FDR = 0%; n = 9/time-point/treatment). Highly similar GO terms are linked by edges and the line width indicates the degree of similarity. Increasing bubble color intensity is associated with increased numbers of significantly changed genes in each GO term, while increasing bubble size is associated with higher frequency of the GO term in the Gene Ontology Annotation database (UniProt-GOA), i.e. higher frequency denotes a more general term. Network images processed via Cytoscape.
(TIF)

S3 Fig GO biological processes (level 6) at 12h post KA injection. Level 6 GO Biological Process terms using REVIGO for all the significantly changed transcripts detected by microarrays in KA- versus saline-injected hippocampi at 12h post injection (SAM analysis, thresholds: fold change > |2|, FDR = 0%; n = 9/time-point/treatment). Highly similar GO terms are linked by edges and the line width indicates the degree of similarity. Increasing bubble color intensity is associated with increased numbers of significantly changed genes in each GO term, while increasing bubble size is associated with higher frequency of the GO term in the Gene Ontology Annotation database (UniProt-GOA), i.e. higher frequency denotes a more general term. Network images processed via Cytoscape.
(TIF)

S4 Fig GO biological processes (level 6) at 24h post KA injection. Level 6 GO Biological Process terms using REVIGO for all the significantly changed transcripts detected by microarrays in KA- versus saline-injected hippocampi at 24h post injection (SAM analysis, thresholds: fold change > |2|, FDR = 0%; n = 9/time-point/treatment). Highly similar GO terms are linked by edges and the line width indicates the degree of similarity. Increasing bubble color intensity is associated with increased numbers of significantly changed genes in each GO term, while increasing bubble size is associated with higher frequency of the GO term in the Gene Ontology Annotation database (UniProt-GOA), i.e. higher frequency denotes a more general term. Network images processed via Cytoscape.
(TIF)

S1 Table Significant gene expression changes detected by microarrays following SAM analysis at 6 hours post-injection (thresholds: ≥ 2 fold and 0% median FDR). (PDF)

S2 Table Significant gene expression changes detected by microarrays following SAM analysis at 12 hours post-injection (thresholds: ≥ 2 fold and 0% median FDR). (PDF)

S3 Table Significant gene expression changes detected by microarrays following SAM analysis at 24 hours post-injection (thresholds: ≥ 2 fold and 0% median FDR). (PDF)

S4 Table qRT-PCR validation of selected microarray statistically significant gene expression changes in KA-versus saline-injected hippocampi, with reference to *Gapdh*. (n/c: not changed)
(PDF)

S5 Table Significantly changed GO biological processes (level 6) at 6 hours post KA treatment, using the "Mapping to ontologies (TRANSPATH®)" workflow. All significantly changed genes at 6h were considered, and a threshold of p-value <0.05 was applied.
(PDF)

S6 Table Significantly changed GO biological processes (level 6) at 12 hours post KA treatment, using the "Mapping to ontologies (TRANSPATH)" analysis tool. All significantly changed genes at 12h were considered, and a threshold of p-value <0.05 was applied.
(PDF)

S7 Table Significantly changed GO biological processes (level 6) at 24 hours post KA treatment, using the "Mapping to ontologies (TRANSPATH)" analysis tool. All significantly changed genes at 24h were considered, and a threshold of p-value <0.05 was applied.
(PDF)

S8 Table Significantly changed GO molecular functions (level 6) at 6 hours post KA treatment, using the "Mapping to ontologies (TRANSPATH®)" workflow. All significantly changed genes at 6h were considered, and a threshold of p-value <0.05 was applied.
(PDF)

S9 Table Significantly changed GO molecular functions (level 6) at 12 hours post KA treatment, using the "Mapping to ontologies (TRANSPATH®)" workflow. All significantly changed genes at 12h were considered, and a threshold of p-value <0.05 was applied.
(PDF)

S10 Table Significantly changed GO cellular components (level 5) at 6 hours post KA treatment, using the "Mapping to ontologies (TRANSPATH®)" workflow. All significantly changed genes at 6h were considered, and a threshold of p-value <0.05 was applied.
(PDF)

S11 Table Significantly changed GO cellular components (level 5) at 12 hours post KA treatment, using the "Mapping to ontologies (TRANSPATH®)" workflow. All significantly changed genes at 12h were considered, and a threshold of p-value <0.05 was applied.
(PDF)

S12 Table Significantly changed GO cellular components (level 5) at 24 hours post KA treatment, using the "Mapping to ontologies (TRANSPATH®)" workflow. All significantly changed genes at 24h were considered, and a threshold of p-value <0.05 was applied.
(PDF)

S13 Table Significantly changed GO molecular functions (level 6) at 24 hours post KA treatment, using the "Mapping to ontologies (TRANSPATH®)" workflow. All significantly changed genes at 24h were considered, and a threshold of p-value <0.05 was applied.
(PDF)

S14 Table Predicted statistically significant upstream regulators of the 24h gene expression changes. The analysis was performed using the "Master regulators in networks (TRANSPATH®)" workflow, for all the significantly changed transcripts at 24h, applying the thresholds FDR <0.05, Score >0.2 and Z-Score >1. The predicted upstream regulators were filtered to exclude those that did not present with statistically significant overexpression themselves at 12h or 24h. The number and symbol of their respective significantly overexpressed downstream gene targets at 24h is included. (n/c: not changed)
(PDF)

S15 Table Experimentally validated mRNA-microRNA interactions for the underexpressed genes at 24h, according to the miRWalk search tool. The microRNAs are sorted by descending number of target genes in the dataset.
(PDF)

S16 Table Experimentally validated mRNA-microRNA interactions for the overexpressed genes at 24h, according to the miRWalk search tool. The microRNAs are sorted by descending number of target genes in the dataset.
(PDF)

S17 Table Primer sequences used for the evaluation of gene expression by qRT-PCR analysis. (PDF)
