# Supplementary figures and images for: Glial responses during epileptogenesis in Mus musculus point to potential therapeutic targets
Source: PLoS One. 2018 Aug 16;13(8):e0201742. doi: 10.1371/journal.pone.0201742 (PMC6095496; doi:10.1371/journal.pone.0201742)

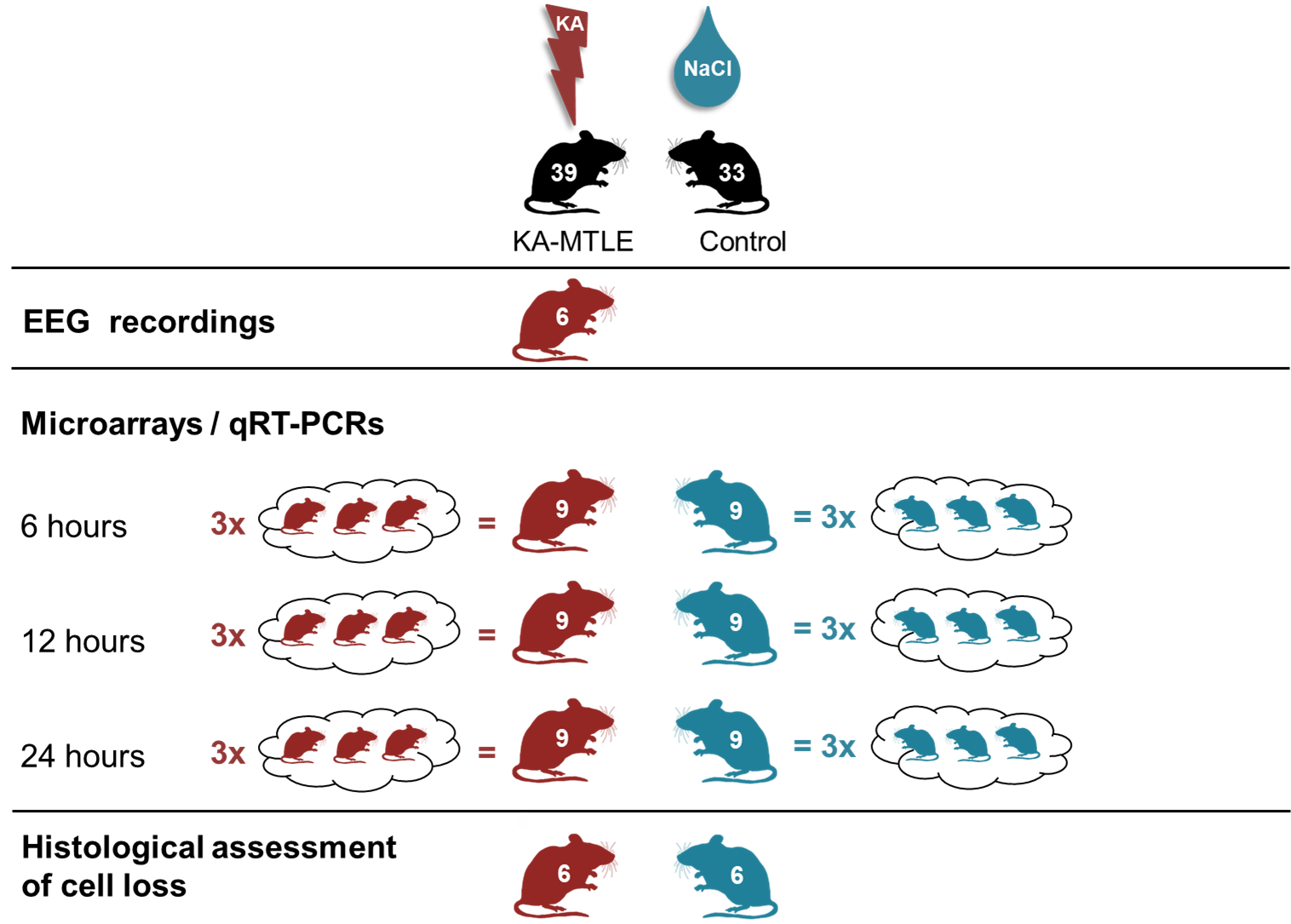

Supplement: S1 Fig — A total of 72 animals were used: 39 injected with KA, and 33 injected with saline as controls. EEG recordings were performed in 6 KA-injected animals. Hippocampal samples from 27 KA- and 27 saline-injected animals (3 different pools [biological replicates] of 3 mice/treatment/time point; 3 time points: 6h, 12h, 24h post injection) were used for microarray and qRT-PCR analyses. Hippocampal samples from 6 KA- and 6 saline-injected animals were used for histological assessment of cell loss. (TIF) [file pone.0201742.s001.tif]

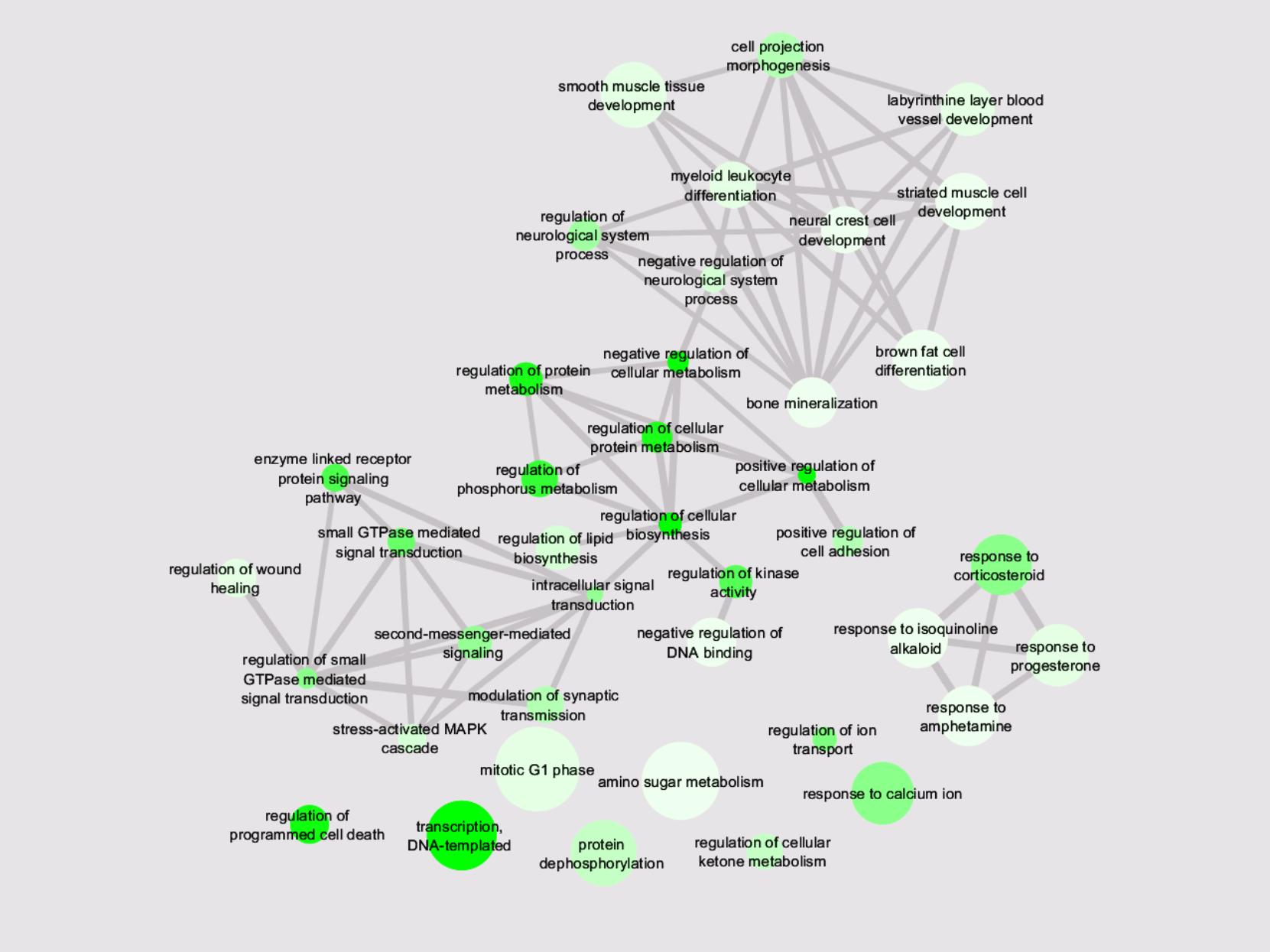

Supplement: S2 Fig — Level 6 GO Biological Process terms via REVIGO for all the significantly changed transcripts detected by microarrays in KA- versus saline-injected hippocampi at 6h post injection (SAM analysis, thresholds: fold change > |2|, FDR = 0%; n = 9/time-point/treatment). Highly similar GO terms are linked by edges and the line width indicates the degree of similarity. Increasing bubble color intensity is associated with increased numbers of significantly changed genes in each GO term, while increasing bubble size is associated with higher frequency of the GO term in the Gene Ontology Annotation database (UniProt-GOA), i.e. higher frequency denotes a more general term. Network images processed via Cytoscape. (TIF) [file pone.0201742.s002.tif]

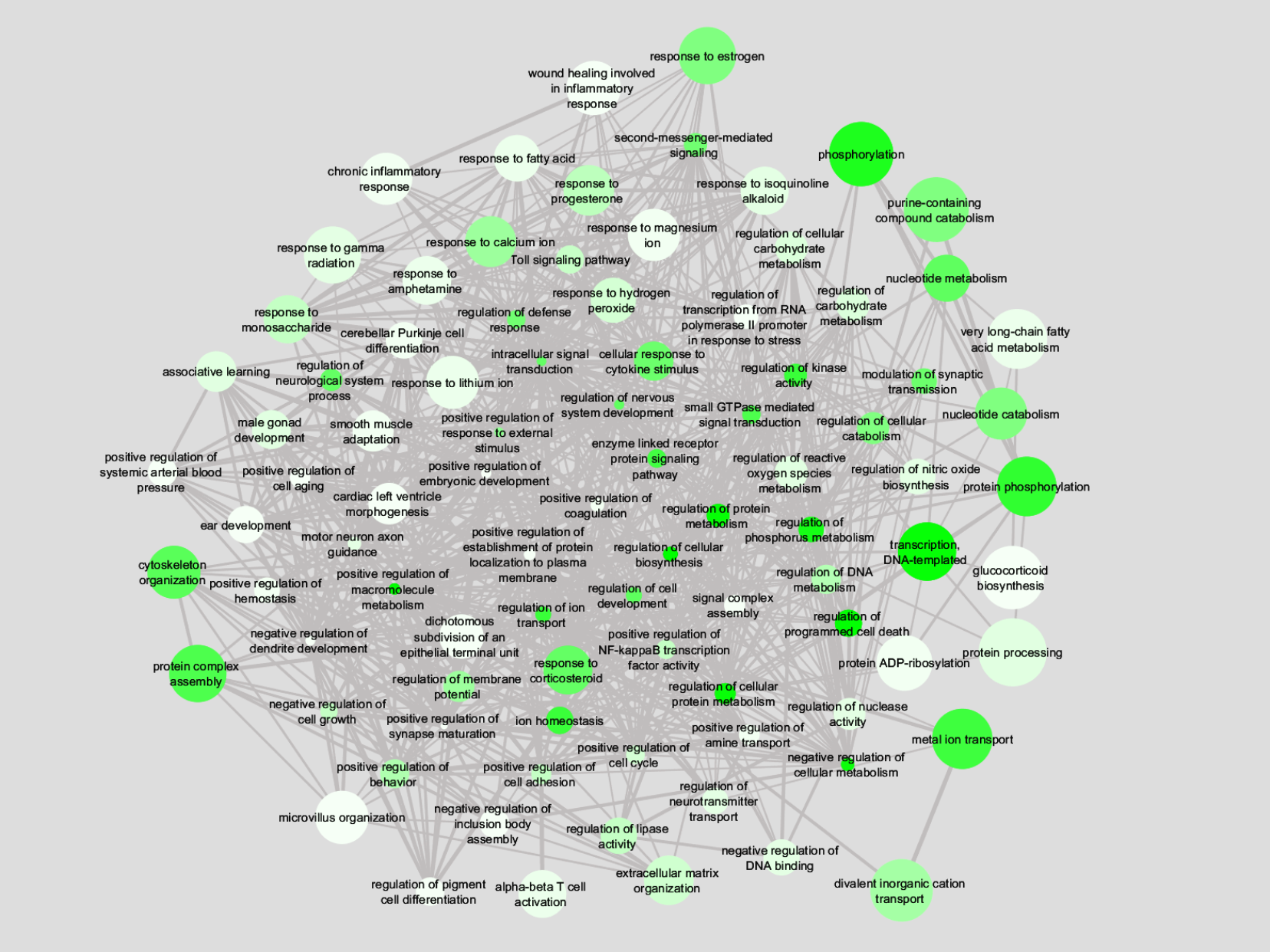

Supplement: S3 Fig — Level 6 GO Biological Process terms using REVIGO for all the significantly changed transcripts detected by microarrays in KA- versus saline-injected hippocampi at 12h post injection (SAM analysis, thresholds: fold change > |2|, FDR = 0%; n = 9/time-point/treatment). Highly similar GO terms are linked by edges and the line width indicates the degree of similarity. Increasing bubble color intensity is associated with increased numbers of significantly changed genes in each GO term, while increasing bubble size is associated with higher frequency of the GO term in the Gene Ontology Annotation database (UniProt-GOA), i.e. higher frequency denotes a more general term. Network images processed via Cytoscape. (TIF) [file pone.0201742.s003.tif]

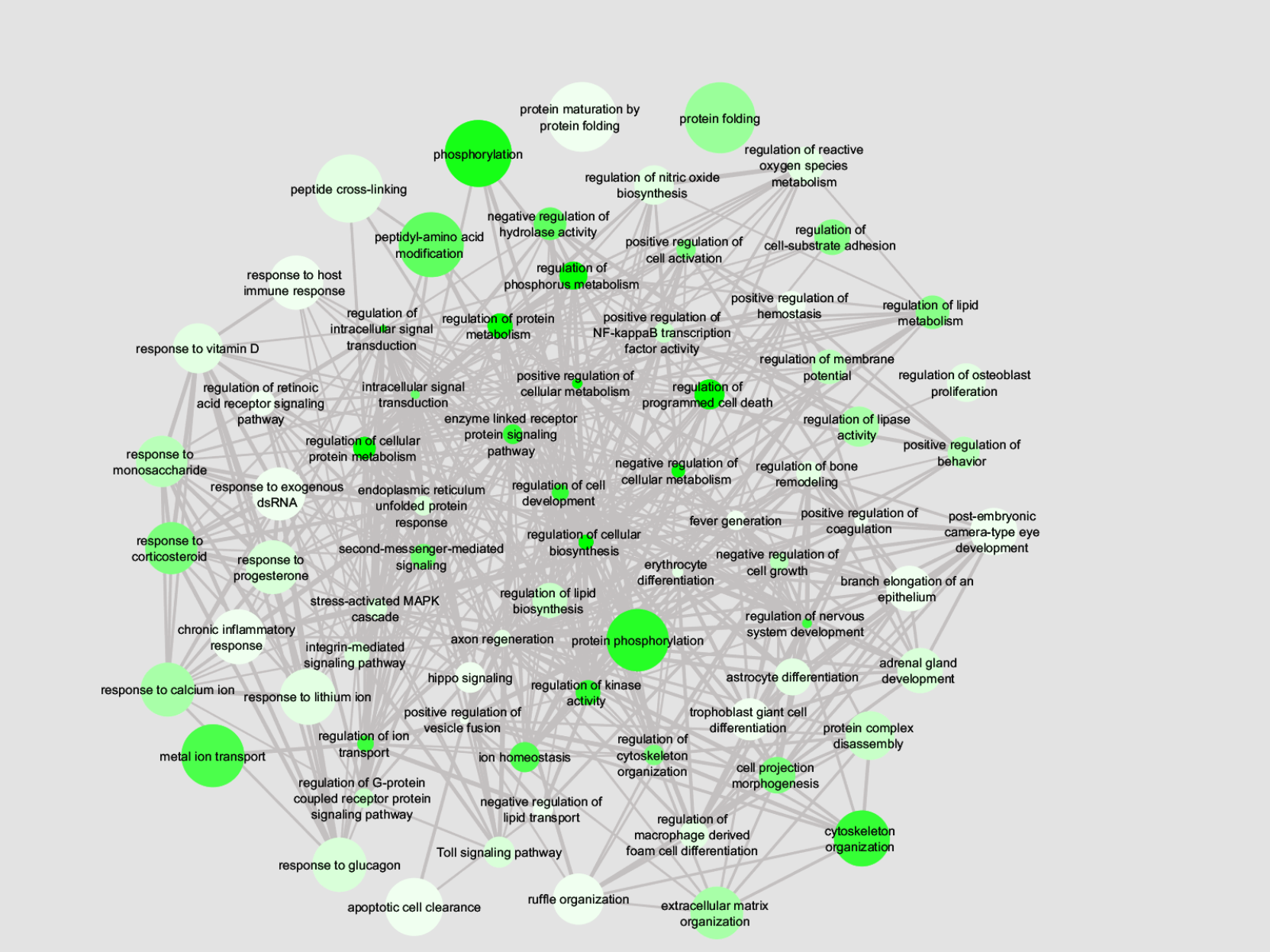

Supplement: S4 Fig — Level 6 GO Biological Process terms using REVIGO for all the significantly changed transcripts detected by microarrays in KA- versus saline-injected hippocampi at 24h post injection (SAM analysis, thresholds: fold change > |2|, FDR = 0%; n = 9/time-point/treatment). Highly similar GO terms are linked by edges and the line width indicates the degree of similarity. Increasing bubble color intensity is associated with increased numbers of significantly changed genes in each GO term, while increasing bubble size is associated with higher frequency of the GO term in the Gene Ontology Annotation database (UniProt-GOA), i.e. higher frequency denotes a more general term. Network images processed via Cytoscape. (TIF) [file pone.0201742.s004.tif]
