# Supplementary material for: Glial responses during epileptogenesis in Mus musculus point to potential therapeutic targets
Source: PLoS One. 2018 Aug 16;13(8):e0201742. doi: 10.1371/journal.pone.0201742 (PMC6095496; doi:10.1371/journal.pone.0201742)
Supplement: S1 Table — (PDF) [file pone.0201742.s005.pdf]

Table S1: Significant gene expression changes detected by microarrays following SAM analysis at 6 hours post-injection (thresholds: $\geq$ 2 fold and 0% median FDR).

Probe Set ID	Gene Symbol	Gene Title	Fold Change
1459372_at	Npas4	neuronal PAS domain protein 4	69.30
1443475_at	Hist1h3i	Histone cluster 1, H3i	24.81
1422134_at	Fosb	FBJ osteosarcoma oncogene B	16.46
1423100_at	Fos	FBJ osteosarcoma oncogene	12.20
1453851_a_at	Gadd45g	growth arrest and DNA-damage-inducible 45 gamma	11.88
1438133_a_at	Cyr61	cysteine rich protein 61	11.50
1418687_at	Arc	activity regulated cytoskeletal-associated protein	11.46
1417262_at	Ptgs2	prostaglandin-endoperoxide synthase 2	10.77
1421396_at	Pcsk1	proprotein convertase subtilisin/kexin type 1	10.43
1424248_at	Arpp21	cyclic AMP-regulated phosphoprotein, 21	9.55
1417263_at	Ptgs2	prostaglandin-endoperoxide synthase 2	8.99
1436387_at	C330006P03Rik	RIKEN cDNA C330006P03 gene	7.52
1448830_at	Dusp1	dual specificity phosphatase 1	7.42
1416505_at	Nr4a1	nuclear receptor subfamily 4, group A, member 1	7.30
1457823_at	Cyr61	cysteine rich protein 61	7.26
1415899_at	Junb	Jun-B oncogene	6.60
1422562_at	Rrad	Ras-related associated with diabetes	6.45
1453287_at	5730557B15Rik	RIKEN cDNA 5730557B15 gene	6.30
1422168_a_at	Bdnf	brain derived neurotrophic factor	6.10
1452160_at	Tiparp	TCDD-inducible poly(ADP-ribose) polymerase	5.99
1440179_x_at	Rnf217	ring finger protein 217	5.99
1428759_s_at	Ccdc49	coiled-coil domain containing 49	5.94
1435872_at	---	Transcribed locus	5.83
1450971_at	Gadd45b	growth arrest and DNA-damage-inducible 45 beta	5.68
1442014_at	---	Transcribed locus	5.60
1437166_at	Tinf2	Terf1 (TRF1)-interacting nuclear factor 2	5.49
1452161_at	Tiparp	TCDD-inducible poly(ADP-ribose) polymerase	5.43
1449773_s_at	Gadd45b	growth arrest and DNA-damage-inducible 45 beta	5.35
1455034_at	---	---	5.33
1417409_at	Jun	Jun oncogene	5.23
1434350_at	Axud1	AXIN1 up-regulated 1	5.11
1418322_at	Crem	cAMP responsive element modulator	5.06
1451386_at	Blvrb	biliverdin reductase B (flavin reductase (NADPH))	4.93
1437199_at	---	---	4.89
1419247_at	Rgs2	regulator of G-protein signaling 2	4.89
1421679_a_at	Cdkn1a	cyclin-dependent kinase inhibitor 1A (P21)	4.83
1415855_at	Kitl	kit ligand	4.77
1441300_at	Kcnf1	potassium voltage-gated channel, subfamily F, member 1	4.73
1439830_at	LOC675366	similar to mitogen activated protein kinase kinase kinase 5	4.51
1422452_at	Bag3	Bcl2-associated athanogene 3	4.40
1451680_at	Srxn1	sulfiredoxin 1 homolog (S. cerevisiae)	4.35

1424638_at	Cdkn1a	cyclin-dependent kinase inhibitor 1A (P21)	4.22
1438796_at	Nr4a3	nuclear receptor subfamily 4, group A, member 3	3.93
1451264_at	Frmd6	FERM domain containing 6	3.88
1448117_at	Kitl	kit ligand	3.86
1426721_s_at	Tiparp	TCDD-inducible poly(ADP-ribose) polymerase	3.86
1449977_at	Egr4	early growth response 4	3.85
1421340_at	LOC675366 /// Map3k5	mitogen activated protein kinase kinase kinase 5 /// similar to mitogen activated protein kinase kinase kinase 5	3.81
1416892_s_at	3110001A13Rik	RIKEN cDNA 3110001A13 gene	3.80
1455085_at	1700086L19Rik	RIKEN cDNA 1700086L19 gene	3.79
1418603_at	Avpr1a	arginine vasopressin receptor 1A	3.73
1450716_at	Adamts1	a disintegrin-like and metallopeptidase (reprolysin type) with thrombospondin type 1 motif, 1	3.65
1419248_at	Rgs2	regulator of G-protein signaling 2	3.64
1421267_a_at	Cited2	Cbp/p300-interacting transactivator, with Glu/Asp-rich carboxy-terminal domain, 2	3.63
1433599_at	Baz1a	bromodomain adjacent to zinc finger domain 1A	3.62
1416529_at	Emp1	epithelial membrane protein 1	3.60
1426875_s_at	Srxn1	sulfiredoxin 1 homolog (S. cerevisiae)	3.53
1437696_at	BC049807	cDNA sequence BC049807	3.53
1456783_at	Zdbf2	zinc finger, DBF-type containing 2	3.50
1429787_x_at	Zwint	ZW10 interactor	3.49
1416067_at	Ifrd1	interferon-related developmental regulator 1	3.49
1456610_at	Jmjd3	jumonji domain containing 3	3.48
1419209_at	Cxcl1	chemokine (C-X-C motif) ligand 1	3.44
1453059_at	2310046A06Rik	RIKEN cDNA 2310046A06 gene	3.42
1423389_at	Smad7	MAD homolog 7 (Drosophila)	3.41
1421756_a_at	Gpr19	G protein-coupled receptor 19	3.40
1416129_at	Errfi1	ERBB receptor feedback inhibitor 1	3.39
1417719_at	Sap30	sin3 associated polypeptide	3.39
1459056_at	Rasgrf1	RAS protein-specific guanine nucleotide-releasing factor 1	3.34
1447452_at	Reep3	Receptor accessory protein 3	3.33
1419816_s_at	Errfi1	ERBB receptor feedback inhibitor 1	3.26
1425162_at	Rorb	RAR-related orphan receptor beta	3.18
1442590_at	Tnfrsf23	tumor necrosis factor receptor superfamily, member 23	3.16
1450708_at	Scg2	secretogranin II	3.14
1449699_s_at	C330027C09Rik	RIKEN cDNA C330027C09 gene	3.11
1423903_at	Pvr	poliovirus receptor	3.11
1452519_a_at	Zfp36	zinc finger protein 36	3.09
1452207_at	Cited2	Cbp/p300-interacting transactivator, with Glu/Asp-rich carboxy-terminal domain, 2	3.07
1415844_at	Syt4	synaptotagmin IV	3.07
1440657_at	---	---	3.06
1444402_at	Zc3h12c	zinc finger CCCH type containing 12C	3.01
1419091_a_at	Anxa2	annexin A2	2.98
1417426_at	Srgn	serglycin	2.97
1448170_at	Siah2	seven in absentia 2	2.94
1426208_x_at	Plagl1	pleiomorphic adenoma gene-like 1	2.94
1436659_at	Dclk1	doublecortin-like kinase 1	2.93
1448303_at	Gpnmb	glycoprotein (transmembrane) nmb	2.83

1427893_a_at	Pmvk	phosphomevalonate kinase	2.82
1433582_at	1190002N15Rik /// LOC100044725	RIKEN cDNA 1190002N15 gene /// hypothetical protein LOC100044725	2.81
1418349_at	Hbegf	heparin-binding EGF-like growth factor	2.81
1451289_at	Dclk1	doublecortin-like kinase 1	2.79
1455372_at	Cpeb3	cytoplasmic polyadenylation element binding protein 3	2.79
1430089_at	5830469G19Rik	RIKEN cDNA 5830469G19 gene	2.73
1435595_at	1810011O10Rik	RIKEN cDNA 1810011O10 gene	2.72
1450744_at	Ell2	elongation factor RNA polymerase II 2	2.70
1433675_at	Snhg1	small nucleolar RNA host gene (non-protein coding) 1	2.69
1422609_at	Arpp19	cAMP-regulated phosphoprotein 19	2.68
1451612_at	Mt1	metallothionein 1	2.66
1420098_s_at	D13Ertd787e	DNA segment, Chr 13, ERATO Doi 787, expressed	2.65
1423312_at	Tpbg	trophoblast glycoprotein	2.59
1420558_at	Selp	selectin, platelet	2.58
1424271_at	Dclk1	doublecortin-like kinase 1	2.53
1446906_at	C81615	expressed sequence C81615	2.52
1450479_x_at	Ptpn12	protein tyrosine phosphatase, non-receptor type 12	2.46
1417483_at	Nfkbiz	nuclear factor of kappa light polypeptide gene enhancer in B-cells inhibitor, zeta	2.45
1455166_at	Arl5b	ADP-ribosylation factor-like 5B	2.41
1434646_s_at	LOC100041953 /// LOC100047209 /// Sap18	Sin3-associated polypeptide 18 /// similar to SAP18	2.41
1433883_at	Tpm4	tropomyosin 4	2.40
1441274_at	---	---	2.40
1417273_at	Pdk4	pyruvate dehydrogenase kinase, isoenzyme 4	2.38
1430535_at	Tsc22d2	TSC22 domain family 2	2.35
1449851_at	Per1	period homolog 1 (Drosophila)	2.32
1415802_at	Slc16a1	solute carrier family 16 (monocarboxylic acid transporters), member 1	2.27
1416600_a_at	Rcan1	regulator of calcineurin 1	2.27
1456048_at	Cpeb3	cytoplasmic polyadenylation element binding protein 3	2.27
1422533_at	Cyp51	cytochrome P450, family 51	2.26
1437868_at	BC023892	cDNA sequence BC023892	2.23
1437667_a_at	Bach2	BTB and CNC homology 2	2.22
1449119_at	Arih2	ariadne homolog 2 (Drosophila)	2.15
1437711_x_at	EG666231 /// EG668343 /// LOC546355 /// LOC627245 /// LOC632337 /// LOC665017 /// LOC676173 /// Odc1	ornithine decarboxylase, structural 1 /// similar to Ornithine decarboxylase (ODC) /// similar to Odc1 protein /// predicted gene, EG666231 /// predicted gene, EG668343	2.14
1451299_at	Prkx	protein kinase, X-linked	2.10
1451819_at	Zswim6	zinc finger, SWIM domain containing 6	2.09
1455271_at	LOC620695	hypothetical protein LOC620695	2.08
1453119_at	Otud1	OTU domain containing 1	2.07

1427005_at	Plk2	polo-like kinase 2 (Drosophila)	2.02
1444878_at	---	Adult male hippocampus cDNA, RIKEN full-length enriched library, clone:C630003L08 product:unclassifiable, full insert sequence	2.01
1460632_at	---	Transcribed locus	2.00
1455145_at	Pcdh19	protocadherin 19	-2.00
1440295_at	1110057K04Rik	RIKEN cDNA 1110057K04 gene	-2.00
1450219_at	Htr1a	5-hydroxytryptamine (serotonin) receptor 1A	-2.00
1428162_at	4933421E11Rik	RIKEN cDNA 4933421E11 gene	-2.00
1460369_at	BC003267	cDNA sequence BC003267	-2.01
1460570_at	Pgbd5	piggyBac transposable element derived 5	-2.01
1426651_at	Mrpl44	mitochondrial ribosomal protein L44	-2.01
1436908_at	LOC100044052	similar to pericentriolar material gene 1 protein	-2.02
1437147_at	Gabrg2	gamma-aminobutyric acid (GABA-A) receptor, subunit gamma 2	-2.02
1450227_at	Ankrd6	ankyrin repeat domain 6	-2.02
1436860_at	Senp7	SUMO1/sentrin specific peptidase 7	-2.02
1428972_at	Tctex1d2	Tctex1 domain containing 2	-2.02
1434140_at	Mcf2l	mcf.2 transforming sequence-like	-2.03
1441963_at	RP23-100C5.8	ProSAPiP1 protein	-2.03
1418954_at	Camkk1	calcium/calmodulin-dependent protein kinase kinase 1, alpha	-2.03
1428207_at	Bcl7a	B-cell CLL/lymphoma 7A	-2.03
1438068_at	---	Transcribed locus, strongly similar to XP_579813.1 hypothetical protein XP_579813 [Rattus norvegicus]	-2.04
1419574_at	Zfp292	zinc finger protein 292	-2.04
1429451_at	2610301B20Rik	RIKEN cDNA 2610301B20 gene	-2.04
1460576_at	LOC100047539	similar to SEC15 homolog	-2.04
1451398_at	Cbr4 /// LOC100046395	carbonyl reductase 4 /// similar to carbonic reductase 4	-2.05
1429089_s_at	2900026A02Rik	RIKEN cDNA 2900026A02 gene	-2.05
1436021_at	Mfsd4	major facilitator superfamily domain containing 4	-2.05
1425593_at	Tnpo2	transportin 2 (importin 3, karyopherin beta 2b)	-2.06
1417147_at	B230317C12Rik	RIKEN cDNA B230317C12 gene	-2.06
1418057_at	Tiam1	T-cell lymphoma invasion and metastasis 1	-2.06
1454973_at	Atf7ip	activating transcription factor 7 interacting protein	-2.07
1440836_at	Setd1b	SET domain containing 1B	-2.07
1429390_at	Acpl2	acid phosphatase-like 2	-2.07
1418048_at	1110059G10Rik	RIKEN cDNA 1110059G10 gene	-2.07
1435796_at	---	---	-2.08
1420871_at	Gucy1b3	guanylate cyclase 1, soluble, beta 3	-2.08
1439497_at	4933415E08Rik	RIKEN cDNA 4933415E08 gene	-2.08
1437183_at	LOC100045241 /// Lrrc4b	leucine rich repeat containing 4B /// similar to Leucine rich repeat containing 4B	-2.08
1434675_at	1700065O13Rik	RIKEN cDNA 1700065O13 gene	-2.09
1421043_s_at	Arhgef2	rho/rac guanine nucleotide exchange factor (GEF) 2	-2.09
1428305_at	Pcsk2	proprotein convertase subtilisin/kexin type 2	-2.09
1427990_at	Usp45	ubiquitin specific petidase 45	-2.09
1427025_at	Mtmr7	myotubularin related protein 7	-2.09
1437292_at	Rlbp1l2	retinaldehyde binding protein 1-like 2	-2.09
1449425_at	Wnt2	wingless-related MMTV integration site 2	-2.09
1450114_at	Ksr1	kinase suppressor of ras 1	-2.09

1457325_at	Kcns2	K+ voltage-gated channel, subfamily S, 2	-2.10
1453342_at	Cdc40	cell division cycle 40 homolog (yeast)	-2.11
1435809_at	Ankrd34	ankyrin repeat domain 34	-2.12
1439024_at	Bag4	BCL2-associated athanogene 4	-2.12
1436275_at	Kcnip2	Kv channel-interacting protein 2	-2.13
1447788_s_at	Tspyl3	TSPY-like 3	-2.13
1446041_at	---	---	-2.13
1419811_at	D16Wsu65e	DNA segment, Chr 16, Wayne State University 65, expressed	-2.14
1417302_at	Rcor2	REST corepressor 2	-2.14
1435984_at	1110033F14Rik	RIKEN cDNA 1110033F14 gene	-2.14
1435285_at	Mpped2	metallophosphoesterase domain containing 2	-2.14
1437023_at	Zfp12	zinc finger protein 12	-2.14
1421923_at	Sh3bp5	SH3-domain binding protein 5 (BTK-associated)	-2.14
1428186_at	Kctd6	potassium channel tetramerisation domain containing 6	-2.15
1434564_at	E2f3	E2F transcription factor 3	-2.15
1435822_at	D830012I24Rik	RIKEN cDNA D830012I24 gene	-2.15
1441983_at	---	0 day neonate cerebellum cDNA, RIKEN full-length enriched library, clone:C230096I08 product:hypothetical protein, full insert sequence	-2.15
1455450_at	---	---	-2.16
1418143_at	Vps45	vacuolar protein sorting 45 (yeast)	-2.17
1428748_at	5830428H23Rik	RIKEN cDNA 5830428H23 gene	-2.17
1452700_s_at	Kbtbd7	kelch repeat and BTB (POZ) domain containing 7	-2.17
1438413_at	Senp7	SUMO1/sentrin specific peptidase 7	-2.17
1423471_at	Ptbp2	polypyrimidine tract binding protein 2	-2.18
1435391_at	Prr8	proline rich 8	-2.18
1448063_at	Iqsec2	IQ motif and Sec7 domain 2	-2.18
1458515_at	Zfp128	zinc finger protein 128	-2.19
1417236_at	Ehd3	EH-domain containing 3	-2.19
1440264_at	---	Adult male aorta and vein cDNA, RIKEN full-length enriched library, clone:A530010O07 product:unclassifiable, full insert sequence	-2.20
1428192_at	Kbtbd7	kelch repeat and BTB (POZ) domain containing 7	-2.21
1437528_x_at	A730017C20Rik	RIKEN cDNA A730017C20 gene	-2.21
1441305_at	---	Adult male corpus striatum cDNA, RIKEN full-length enriched library, clone:C030044L04 product:unclassifiable, full insert sequence	-2.21
1428338_at	Spata2L	spermatogenesis associated 2-like	-2.22
1438417_at	Pwwp2b	PWWP domain containing 2B	-2.22
1444978_at	Foxp1	Forkhead box P1	-2.22
1438328_at	Hcfc2	host cell factor C2	-2.22
1417575_at	Otub2	OTU domain, ubiquitin aldehyde binding 2	-2.22
1456789_at	Zfp462	zinc finger protein 462	-2.22
1424044_at	Jmjd2b	jumonji domain containing 2B	-2.22
1447852_x_at	Rilpl1	Rab interacting lysosomal protein-like 1	-2.23
1441801_at	Kctd4	potassium channel tetramerisation domain containing 4	-2.23
1438710_at	Htr1a	5-hydroxytryptamine (serotonin) receptor 1A	-2.23

1434776_at	Sema5a	sema domain, seven thrombospondin repeats (type 1 and type 1-like), transmembrane domain (TM) and short cytoplasmic domain, (semaphorin) 5A	-2.24
1424784_at	OTTMUSG00000010657	predicted gene, OTTMUSG00000010657	-2.24
1437168_at	Srrp	serine-arginine repressor protein	-2.24
1434638_at	---	Adult male corpora quadrigemina cDNA, RIKEN full-length enriched library, clone:B230036G05 product:unclassifiable, full insert sequence	-2.25
1455463_at	Phyhip	phytanoyl-CoA hydroxylase interacting protein	-2.25
1418109_at	Gspt2	G1 to S phase transition 2	-2.25
1449173_at	Mpp2	membrane protein, palmitoylated 2 (MAGUK p55 subfamily member 2)	-2.26
1432813_at	2900064F13Rik	RIKEN cDNA 2900064F13 gene	-2.28
1452065_at	Vstm2a	V-set and transmembrane domain containing 2A	-2.29
1430301_at	Stxbp5	syntaxin binding protein 5 (tomosyn)	-2.30
1451245_at	Lrrc3b	leucine rich repeat containing 3B	-2.30
1439993_at	---	Transcribed locus	-2.31
1429105_at	Dlgap1	discs, large (Drosophila) homolog-associated protein 1	-2.31
1425344_at	Narf	nuclear prelamin A recognition factor	-2.32
1417235_at	Ehd3	EH-domain containing 3	-2.33
1456423_at	Mbd5	methyl-CpG binding domain protein 5	-2.33
1455700_at	Mterfd3	MTERF domain containing 3	-2.34
1451566_at	Zfp810	zinc finger protein 810	-2.34
1442157_at	A930041H05Rik	RIKEN cDNA A930041H05 gene	-2.36
1419732_at	Rtn4r	reticulon 4 receptor	-2.36
1435311_s_at	Fbxo7	F-box protein 7	-2.37
1441327_a_at	Ssr1	signal sequence receptor, alpha	-2.38
1457628_at	Ccdc53	coiled-coil domain containing 53	-2.39
1424697_at	Dtwd1	DTW domain containing 1	-2.39
1435396_at	Stxbp6	syntaxin binding protein 6 (amisyn)	-2.39
1460588_at	---	10 days lactation, adult female mammary gland cDNA, RIKEN full-length enriched library, clone:D730013G10 product:unclassifiable, full insert sequence	-2.40
1435344_at	1110029I05Rik /// LOC100044848	RIKEN cDNA 1110029I05 gene /// hypothetical protein LOC100044848	-2.41
1455476_a_at	Gse1	genetic suppressor element 1	-2.41
1456764_at	Slc35f3	solute carrier family 35, member F3	-2.43
1436972_at	Gpr61 /// LOC100043654 /// LOC100047381	G protein-coupled receptor 61 /// hypothetical protein LOC100043654 /// hypothetical protein LOC100047381	-2.43
1455365_at	Cdh8	cadherin 8	-2.44
1422710_a_at	Cacna1h	calcium channel, voltage-dependent, T type, alpha 1H subunit	-2.44
1417941_at	Nanp	N-acetylneuraminic acid phosphatase	-2.48
1427646_a_at	Arhgef2	rho/rac guanine nucleotide exchange factor (GEF) 2	-2.48
1452092_at	4631426J05Rik	RIKEN cDNA 4631426J05 gene	-2.51
1415935_at	Smoc2	SPARC related modular calcium binding 2	-2.52
1429918_at	Arhgap20	Rho GTPase activating protein 20	-2.53

1435411_at	Neurod2	neurogenic differentiation 2	-2.54
1415774_at	Elp2	elongation protein 2 homolog (S. cerevisiae)	-2.54
1442272_at	1700021K10Rik	RIKEN cDNA 1700021K10 gene	-2.55
1452041_at	Klhl26	kelch-like 26 (Drosophila)	-2.57
1437966_at	Prrt3	proline-rich transmembrane protein 3	-2.57
1456197_x_at	Ajap1	Adherens junction associated protein 1	-2.60
1452248_at	Plekhg5	pleckstrin homology domain containing, family G (with RhoGef domain) member 5	-2.64
1421042_at	Arhgef2	rho/rac guanine nucleotide exchange factor (GEF) 2	-2.67
1437083_at	Tmem169	transmembrane protein 169	-2.68
1455000_at	Gpr68	G protein-coupled receptor 68	-2.69
1435469_at	Qsox2	quiescin Q6 sulfhydryl oxidase 2	-2.69
1431724_a_at	P2ry12	purinergic receptor P2Y, G-protein coupled 12	-2.69
1433793_s_at	Nrip2	nuclear receptor interacting protein 2	-2.70
1443287_at	Gm1337	gene model 1337, (NCBI)	-2.71
1434655_at	Foxk1	forkhead box K1	-2.71
1429741_at	Kcnv1	potassium channel, subfamily V, member 1	-2.72
1440181_at	Gm1568	gene model 1568, (NCBI)	-2.74
1446612_at	9330118A15Rik	RIKEN cDNA 9330118A15 gene	-2.74
1447385_at	---	Adult male corpora quadrigemina cDNA, RIKEN full-length enriched library, clone:B230036G05 product:unclassifiable, full insert sequence	-2.78
1457729_at	---	Transcribed locus	-2.79
1416798_a_at	Nme4	expressed in non-metastatic cells 4, protein	-2.81
1435991_at	Nr3c2	nuclear receptor subfamily 3, group C, member 2	-2.82
1433885_at	Iqgap2	IQ motif containing GTPase activating protein 2	-2.85
1455040_s_at	1110062M06Rik	RIKEN cDNA 1110062M06 gene	-2.89
1454877_at	Sertad4	SERTA domain containing 4	-2.89
1444687_at	C1ql2	complement component 1, q subcomponent-like 2	-2.90
1448530_at	Gmpr /// LOC100045393	guanosine monophosphate reductase /// similar to guanosine monophosphate reductase	-2.90
1460625_at	Gm1568	gene model 1568, (NCBI)	-2.94
1456838_at	BC072620	cDNA sequence BC072620	-2.96
1458406_at	AI429294	Expressed sequence AI429294	-2.98
1429167_at	Ccdc112	coiled-coil domain containing 112	-3.01
1434819_at	St6gal2	beta galactoside alpha 2,6 sialyltransferase 2	-3.05
1434052_at	AI593442	expressed sequence AI593442	-3.07
1443876_at	---	16 days neonate thymus cDNA, RIKEN full-length enriched library, clone:A130020E07 product:calcium/calmodulin-dependent protein kinase II alpha, full insert sequence	-3.12
1429191_at	Dhx33	DEAH (Asp-Glu-Ala-His) box polypeptide 33	-3.14
1453847_at	A930037O16Rik	RIKEN cDNA A930037O16 gene	-3.14
1444723_at	6530418L21Rik	RIKEN cDNA 6530418L21 gene	-3.17
1452966_at	Bcl11b	B-cell leukemia/lymphoma 11B	-3.25
1419008_at	Npy5r	neuropeptide Y receptor Y5	-3.27
1443800_at	---	Transcribed locus	-3.30
1435407_at	---	Adult male medulla oblongata cDNA, RIKEN full-length enriched library, clone:6332404K23 product:unclassifiable, full insert sequence	-3.42
1418047_at	Neurod6	neurogenic differentiation 6	-3.49

1456389_at	Zeb2	zinc finger E-box binding homeobox 2	-3.67
1438465_at	5830428H23Rik	RIKEN cDNA 5830428H23 gene	-3.99
1447726_at	Ripply2	ripply2 homolog (zebrafish)	-4.00
1433469_at	Lrrn2	leucine rich repeat protein 2, neuronal	-4.36
1427610_at	Dsp	desmoplakin	-4.84
