# Supplementary material for: Glial responses during epileptogenesis in Mus musculus point to potential therapeutic targets
Source: PLoS One. 2018 Aug 16;13(8):e0201742. doi: 10.1371/journal.pone.0201742 (PMC6095496; doi:10.1371/journal.pone.0201742)
Supplement: S2 Table — (PDF) [file pone.0201742.s006.pdf]

Table S2: Significant gene expression changes detected by microarrays following SAM analysis at 12 hours post-injection (thresholds: ≥ 2 fold and 0% median FDR).

Probe Set ID	Gene Symbol	Gene Title	Fold Change
1421134_at	Areg	amphiregulin	47.64
1422947_at	Hist1h4a	histone cluster 1, H4a	44.89
1417488_at	Fosl1	fos-like antigen 1	40.19
1459372_at	Npas4	neuronal PAS domain protein 4	31.03
1429157_at	4930507C10Rik	RIKEN cDNA 4930507C10 gene	30.74
1440576_at	Cpn1	carboxypeptidase N, polypeptide 1	29.20
1449982_at	Il11	interleukin 11	23.11
1420753_at	Tll1	tolloid-like	21.60
1422134_at	Fosb	FBJ osteosarcoma oncogene B	20.92
1417789_at	Ccl11	small chemokine (C-C motif) ligand 11	18.82
1427378_at	Krt75	keratin 75	18.44
1452388_at	Hspa1a	heat shock protein 1A	17.18
1423100_at	Fos	FBJ osteosarcoma oncogene	17.00
1422562_at	Rrad	Ras-related associated with diabetes	16.36
1448789_at	Aldh1a3	aldehyde dehydrogenase family 1, subfamily A3	16.21
1425359_at	Mall	mal, T-cell differentiation protein-like	15.84
1450297_at	Il6	interleukin 6	15.60
1427126_at	Hspa1b	heat shock protein 1B	15.56
1443475_at	Hist1h3i	Histone cluster 1, H3i	13.64
1427127_x_at	Hspa1b	heat shock protein 1B	13.43
1449254_at	Spp1	secreted phosphoprotein 1	12.21
1418932_at	LOC100046232 /// Nfil3	nuclear factor, interleukin 3, regulated /// similar to NFIL3/E4BP4 transcription factor	11.91
1453851_a_at	Gadd45g	growth arrest and DNA-damage-inducible 45 gamma	11.80
1425671_at	Homer1	homer homolog 1 (Drosophila)	11.52
1452409_at	Gltscr2	glioma tumor suppressor candidate region gene 2	11.33
1438133_a_at	Cyr61	cysteine rich protein 61	11.22
1439764_s_at	Igf2bp2	insulin-like growth factor 2 mRNA binding protein 2	10.90
1442340_x_at	Cyr61	cysteine rich protein 61	10.27
1425964_x_at	Hspb1	heat shock protein 1	10.10
1421811_at	LOC640441 /// Thbs1	thrombospondin 1 /// similar to thrombospondin 1	10.08
1427682_a_at	Egr2	early growth response 2	10.03
1417262_at	Ptgs2	prostaglandin-endoperoxide synthase 2	9.89
1451596_a_at	Sphk1	sphingosine kinase 1	9.68
1422943_a_at	Hspb1	heat shock protein 1	9.30
1421396_at	Pcsk1	proprotein convertase subtilisin/kexin type 1	9.26
1417263_at	Ptgs2	prostaglandin-endoperoxide synthase 2	9.23
1449960_at	Nptx2	neuronal pentraxin 2	9.22

1435137_s_at	1200015M12Rik /// 1200016E24Rik /// A130040M12Rik /// E430024C06Rik	RIKEN cDNA 1200015M12 gene /// RIKEN cDNA 1200016E24 gene /// RIKEN cDNA A130040M12 gene /// RIKEN cDNA E430024C06 gene	9.11
1418572_x_at	Tnfrsf12a	tumor necrosis factor receptor superfamily, member 12a	8.89
1452318_a_at	Hspa1b	heat shock protein 1B	8.71
1416039_x_at	Cyr61	cysteine rich protein 61	8.64
1456212_x_at	Socs3	suppressor of cytokine signaling 3	8.53
1452418_at	1200016E24Rik /// LOC100039378 /// LOC100039464 /// LOC100040148 /// LOC100041150 /// LOC100041274 /// LOC100042075 /// LOC100042387 /// LOC100043154 /// LOC100043406 /// LOC100043650 /// LOC100043680 /// LOC100047599 /// LOC100048290	RIKEN cDNA 1200016E24 gene /// similar to gag protein	8.46
1457984_at	Crh	corticotropin releasing hormone	8.35
1450533_a_at	Plagl1	pleiomorphic adenoma gene-like 1	8.22
1460302_at	LOC640441 /// Thbs1	thrombospondin 1 /// similar to thrombospondin 1	8.19
1419772_at	---	Transcribed locus	8.18
1417335_at	Sult2b1	sulfotransferase family, cytosolic, 2B, member 1	7.88
1419149_at	Serpine1	serine (or cysteine) peptidase inhibitor, clade E, member 1	7.67
1447863_s_at	Nr4a2	nuclear receptor subfamily 4, group A, member 2	7.63
1425288_at	Samd11	sterile alpha motif domain containing 11	7.52
1417936_at	Ccl9	chemokine (C-C motif) ligand 9	7.51
1450971_at	Gadd45b	growth arrest and DNA-damage-inducible 45 beta	7.46
1417268_at	Cd14	CD14 antigen	7.33
1418349_at	Hbegf	heparin-binding EGF-like growth factor	7.26

1416529_at	Emp1	epithelial membrane protein 1	7.25
1418350_at	Hbegf	heparin-binding EGF-like growth factor	7.25
1418687_at	Arc	activity regulated cytoskeletal-associated protein	7.19
1448239_at	Hmox1	heme oxygenase (decycling) 1	7.01
1420394_s_at	Gp49a /// Lilrb4	glycoprotein 49 A /// leukocyte immunoglobulin-like receptor, subfamily B, member 4	6.94
1434089_at	Synpo	synaptopodin	6.84
1424638_at	Cdkn1a	cyclin-dependent kinase inhibitor 1A (P21)	6.79
1418936_at	Maff	v-maf musculoaponeurotic fibrosarcoma oncogene family, protein F (avian)	6.77
1423760_at	Cd44	CD44 antigen	6.71
1421854_at	Fgl2	fibrinogen-like protein 2	6.62
1448898_at	Ccl9	chemokine (C-C motif) ligand 9	6.59
1430352_at	Adamts9	a disintegrin-like and metallopeptidase (reprolysin type) with thrombospondin type 1 motif, 9	6.58
1434376_at	Cd44	CD44 antigen	6.47
1419598_at	Ms4a6d	membrane-spanning 4-domains, subfamily A, member 6D	6.35
1426808_at	Lgals3	lectin, galactose binding, soluble 3	6.34
1417487_at	Fosl1	fos-like antigen 1	6.34
1418571_at	Tnfrsf12a	tumor necrosis factor receptor superfamily, member 12a	6.31
1427683_at	Egr2	early growth response 2	6.20
1418133_at	Bcl3	B-cell leukemia/lymphoma 3	6.13
1420380_at	Ccl2	chemokine (C-C motif) ligand 2	5.98
1448074_at	Rln1	Relaxin 1	5.98
1434350_at	Axud1	AXIN1 up-regulated 1	5.90
1433599_at	Baz1a	bromodomain adjacent to zinc finger domain 1A	5.75
1432831_at	Ccdc138	coiled-coil domain containing 138	5.67
1422452_at	Bag3	Bcl2-associated athanogene 3	5.62
1416266_at	Pdyn	prodynorphin	5.52
1450750_a_at	Nr4a2	nuclear receptor subfamily 4, group A, member 2	5.46
1437247_at	Fosl2 /// LOC634417	fos-like antigen 2 /// similar to fos-like antigen 2	5.43
1451680_at	Srxn1	sulfiredoxin 1 homolog (S. cerevisiae)	5.43
1427932_s_at	1200003I10Rik /// 1200015M12Rik /// 1200016E24Rik /// A130040M12Rik /// E430024C06Rik /// LOC100039464	RIKEN cDNA 1200003I10 gene /// RIKEN cDNA 1200015M12 gene /// RIKEN cDNA 1200016E24 gene /// RIKEN cDNA A130040M12 gene /// RIKEN cDNA E430024C06 gene /// similar to gag protein	5.39
1422931_at	Fosl2	fos-like antigen 2	5.31
1435872_at	---	Transcribed locus	5.30
1428909_at	A130040M12Rik	RIKEN cDNA A130040M12 gene	5.19
1427414_at	Prkar2a	protein kinase, cAMP dependent regulatory, type II alpha	5.14
1426063_a_at	Gem	GTP binding protein (gene overexpressed in skeletal muscle)	5.10

1420994_at	B3gnt5	UDP-GlcNAc:betaGal beta-1,3-N-acetylglucosaminyltransferase 5	4.91
1422256_at	Sstr2	somatostatin receptor 2	4.90
1455130_at	Spty2d1	SPT2, Suppressor of Ty, domain containing 1 (S. cerevisiae)	4.88
1426875_s_at	Srxn1	sulfiredoxin 1 homolog (S. cerevisiae)	4.87
1444982_at	---	---	4.84
1437173_at	Edg3	endothelial differentiation, sphingolipid G-protein-coupled receptor, 3	4.81
1454399_at	2010003H20Rik	RIKEN cDNA 2010003H20 gene	4.79
1438658_a_at	Edg3	endothelial differentiation, sphingolipid G-protein-coupled receptor, 3	4.76
1419599_s_at	Ms4a6d	membrane-spanning 4-domains, subfamily A, member 6D	4.75
1419091_a_at	Anxa2	annexin A2	4.66
1422825_at	Cartpt	CART prepropeptide	4.62
1451160_s_at	Pvr	poliovirus receptor	4.60
1417266_at	Ccl6	chemokine (C-C motif) ligand 6	4.60
1421855_at	Fgl2	fibrinogen-like protein 2	4.59
1431231_at	Hist1h3b /// Hist1h3d /// Hist1h3e /// Hist1h3f /// Hist2h3b /// Hist2h3c1 /// Hist2h3c2	histone cluster 2, H3c1 /// histone cluster 2, H3c2 /// histone cluster 1, H3f /// histone cluster 1, H3d /// histone cluster 1, H3b /// histone cluster 1, H3e /// histone cluster 2, H3b	4.59
1419247_at	Rgs2	regulator of G-protein signaling 2	4.58
1418547_at	Tfpi2	tissue factor pathway inhibitor 2	4.54
1434885_at	Spty2d1	SPT2, Suppressor of Ty, domain containing 1 (S. cerevisiae)	4.47
1456046_at	Cd93	CD93 antigen	4.47
1452534_a_at	Hmgb2	high mobility group box 2	4.45
1452160_at	Tiparp	TCDD-inducible poly(ADP-ribose) polymerase	4.44
1428776_at	Slc10a6	solute carrier family 10 (sodium/bile acid cotransporter family), member 6	4.41
1448830_at	Dusp1	dual specificity phosphatase 1	4.38
1451415_at	1810011O10Rik	RIKEN cDNA 1810011O10 gene	4.36
1426348_at	Col4a1	collagen, type IV, alpha 1	4.36
1421578_at	Ccl4	chemokine (C-C motif) ligand 4	4.36
1439407_x_at	Tagln2	transgelin 2	4.35
1421973_at	Gfra1	glial cell line derived neurotrophic factor family receptor alpha 1	4.32
1447825_x_at	Pcdh8	protocadherin 8	4.29
1448061_at	Msr1	macrophage scavenger receptor 1	4.25
1427299_at	Rps6ka3	ribosomal protein S6 kinase polypeptide 3	4.24
1417601_at	Rgs1	regulator of G-protein signaling 1	4.22
1454742_at	LOC100044232 /// Rasgef1b	RasGEF domain family, member 1B /// hypothetical protein LOC100044232	4.19
1452519_a_at	Zfp36	zinc finger protein 36	4.19
1419589_at	Cd93	CD93 antigen	4.19
1438783_at	AW742560	Expressed sequence AW742560	4.16
1450767_at	Nedd9	neural precursor cell expressed, developmentally down-regulated gene 9	4.16
1433675_at	Snhg1	small nucleolar RNA host gene (non-protein coding) 1	4.15

1416811_s_at	Ctla2a /// Ctla2b	cytotoxic T lymphocyte-associated protein 2 alpha /// cytotoxic T lymphocyte-associated protein 2 beta	4.14
1435595_at	1810011O10Rik	RIKEN cDNA 1810011O10 gene	4.13
1436659_at	Dclk1	doublecortin-like kinase 1	4.11
1424067_at	Icam1	intercellular adhesion molecule	4.10
1449227_at	Ch25h	cholesterol 25-hydroxylase	4.03
1422169_a_at	Bdnf	brain derived neurotrophic factor	4.02
1419004_s_at	Bcl2a1a /// Bcl2a1b /// Bcl2a1d	B-cell leukemia/lymphoma 2 related protein A1a /// B-cell leukemia/lymphoma 2 related protein A1b /// B-cell leukemia/lymphoma 2 related protein A1d	4.00
1419884_at	---	Transcribed locus	4.00
1423312_at	Tpbg	trophoblast glycoprotein	3.99
1450708_at	Scg2	secretogranin II	3.97
1417426_at	Srgn	serglycin	3.91
1437132_x_at	Nedd9	neural precursor cell expressed, developmentally down-regulated gene 9	3.89
1435458_at	Pim1	proviral integration site 1	3.88
1425434_a_at	Msr1	macrophage scavenger receptor 1	3.88
1457276_at	Snf1lk2	SNF1-like kinase 2	3.87
1456453_at	---	---	3.85
1416700_at	Rnd3	Rho family GTPase 3	3.85
1418674_at	Osmr	oncostatin M receptor	3.84
1416067_at	Ifrd1	interferon-related developmental regulator 1	3.75
1452352_at	Ctla2b	cytotoxic T lymphocyte-associated protein 2 beta	3.74
1455034_at	---	---	3.72
1416755_at	Dnajb1	DnaJ (Hsp40) homolog, subfamily B, member 1	3.70
1423905_at	Pvr	poliovirus receptor	3.66
1430407_at	3110035C09Rik	RIKEN cDNA 3110035C09 gene	3.66
1455872_at	BC065085	cDNA sequence BC065085	3.62
1419248_at	Rgs2	regulator of G-protein signaling 2	3.61
1437103_at	Igf2bp2	insulin-like growth factor 2 mRNA binding protein 2	3.60
1416600_a_at	Rcan1	regulator of calcineurin 1	3.60
1417051_at	Pcdh8	protocadherin 8	3.59
1428783_at	Prkar2a	protein kinase, cAMP dependent regulatory, type II alpha	3.59
1427540_at	Zwint	ZW10 interactor	3.58
1416893_at	3110001A13Rik	RIKEN cDNA 3110001A13 gene	3.57
1416762_at	S100a10	S100 calcium binding protein A10 (calpactin)	3.57
1450958_at	Tm4sf1	transmembrane 4 superfamily member 1	3.57
1416442_at	Ier2	immediate early response 2	3.55
1416756_at	Dnajb1	DnaJ (Hsp40) homolog, subfamily B, member 1	3.54
1449037_at	Crem	cAMP responsive element modulator	3.53
1447448_s_at	Klf6	Kruppel-like factor 6	3.52
1415855_at	Kitl	kit ligand	3.51
1444307_at	---	Transcribed locus	3.50
1450710_at	Jarid2	jumonji, AT rich interactive domain 2	3.48
1450716_at	Adamts1	a disintegrin-like and metallopeptidase (reprolysin type) with thrombospondin type 1 motif, 1	3.48
1426721_s_at	Tiparp	TCDD-inducible poly(ADP-ribose) polymerase	3.47

1418892_at	Rhoj	ras homolog gene family, member J	3.42
1417406_at	Sertad1	SERTA domain containing 1	3.39
1417719_at	Sap30	sin3 associated polypeptide	3.35
1432176_a_at	Eng	endoglin	3.33
1450379_at	Msn	moesin	3.32
1456642_x_at	S100a10	S100 calcium binding protein A10 (calpactin)	3.32
1448069_at	Tm4sf1	transmembrane 4 superfamily member 1	3.32
1442606_at	---	---	3.32
1417523_at	Plek	pleckstrin	3.31
1425993_a_at	Hsp110	heat shock protein 110	3.30
1445918_at	Tmem2	Transmembrane protein 2	3.29
1433842_at	Lrrfip1	leucine rich repeat (in FLII) interacting protein 1	3.27
1416601_a_at	Rcan1	regulator of calcineurin 1	3.27
1435906_x_at	Gbp2	guanylate nucleotide binding protein 2	3.27
1420249_s_at	Ccl6	chemokine (C-C motif) ligand 6	3.26
1417109_at	Tinagl	tubulointerstitial nephritis antigen-like	3.25
1419100_at	Serpina3n	serine (or cysteine) peptidase inhibitor, clade A, member 3N	3.25
1434447_at	Met	met proto-oncogene	3.23
1440831_at	Bach1	BTB and CNC homology 1	3.22
1417273_at	Pdk4	pyruvate dehydrogenase kinase, isoenzyme 4	3.22
1438761_a_at	EG666231 /// EG668343 /// LOC546355 /// LOC627245 /// LOC632337 /// LOC677259 /// Odc1	ornithine decarboxylase, structural 1 /// similar to Ornithine decarboxylase (ODC) /// similar to Odc1 protein /// predicted gene, EG666231 /// predicted gene, EG668343	3.21
1426246_at	Pros1	protein S (alpha)	3.21
1437481_at	LOC623451	hypothetical LOC623451	3.20
1423566_a_at	Hsp110	heat shock protein 110	3.19
1428083_at	2310043N10Rik	RIKEN cDNA 2310043N10 gene	3.18
1436305_at	Rnf217	ring finger protein 217	3.18
1419816_s_at	Errfi1	ERBB receptor feedback inhibitor 1	3.18
1456610_at	Jmjd3	jumonji domain containing 3	3.18
1452207_at	Cited2	Cbp/p300-interacting transactivator, with Glu/Asp-rich carboxy-terminal domain, 2	3.17
1438725_at	Med13	mediator complex subunit 13	3.16
1416881_at	LOC632101 /// Mcl1	myeloid cell leukemia sequence 1 /// similar to myeloid cell leukemia sequence 1	3.15
1416212_at	Magoh	mago-nashi homolog, proliferation-associated (Drosophila)	3.15
1426871_at	Fbxo33	F-box protein 33	3.15
1416129_at	Errfi1	ERBB receptor feedback inhibitor 1	3.14
1417818_at	Wwtr1	WW domain containing transcription regulator 1	3.14
1451264_at	Frmd6	FERM domain containing 6	3.13
1425162_at	Rorb	RAR-related orphan receptor beta	3.13
1433508_at	Klf6	Kruppel-like factor 6	3.12
1417409_at	Jun	Jun oncogene	3.11
1433674_a_at	Snhg1	small nucleolar RNA host gene (non-protein coding) 1	3.09
1434976_x_at	Eif4ebp1	eukaryotic translation initiation factor 4E binding protein 1	3.09
1419483_at	C3ar1	complement component 3a receptor 1	3.08
1448318_at	Adfp	adipose differentiation related protein	3.08

1417413_at	Cuzd1	CUB and zona pellucida-like domains 1	3.08
1423904_a_at	Pvr	poliovirus receptor	3.07
1425896_a_at	Fbn1	fibrillin 1	3.07
1448117_at	Kitl	kit ligand	3.06
1458308_at	Sbno2	strawberry notch homolog 2 (Drosophila)	3.06
1423903_at	Pvr	poliovirus receptor	3.06
1448503_at	LOC632101 /// Mcl1	myeloid cell leukemia sequence 1 /// similar to myeloid cell leukemia sequence 1	3.05
1457676_at	Tirap	toll-interleukin 1 receptor (TIR) domain-containing adaptor protein	3.04
1455247_at	Amotl1	angiomotin-like 1	3.02
1433883_at	Tpm4	tropomyosin 4	3.02
1453328_at	2700008G24Rik	RIKEN cDNA 2700008G24 gene	3.02
1460227_at	Timp1	tissue inhibitor of metalloproteinase 1	3.02
1435084_at	C730049O14Rik	RIKEN cDNA C730049O14 gene	3.01
1457451_at	---	---	2.99
1419132_at	Tlr2	toll-like receptor 2	2.97
1419647_a_at	Ier3	immediate early response 3	2.97
1451714_a_at	Map2k3	mitogen activated protein kinase kinase 3	2.95
1437277_x_at	Tgm2	transglutaminase 2, C polypeptide	2.93
1427364_a_at	Odc1	ornithine decarboxylase, structural 1	2.93
1460740_at	Cltb /// LOC100046457	clathrin, light polypeptide (Lcb) /// similar to Cltb protein	2.93
1436778_at	Cybb	cytochrome b-245, beta polypeptide	2.92
1450355_a_at	Capg	capping protein (actin filament), gelsolin-like	2.92
1451021_a_at	Klf5	Kruppel-like factor 5	2.90
1437711_x_at	EG666231 /// EG668343 /// LOC546355 /// LOC627245 /// LOC632337 /// LOC665017 /// LOC676173 /// Odc1	ornithine decarboxylase, structural 1 /// similar to Ornithine decarboxylase (ODC) /// similar to Odc1 protein /// predicted gene, EG666231 /// predicted gene, EG668343	2.90
1439399_a_at	Snhg1	small nucleolar RNA host gene (non-protein coding) 1	2.90
1453287_at	5730557B15Rik	RIKEN cDNA 5730557B15 gene	2.90
1415899_at	Junb	Jun-B oncogene	2.89
1428319_at	Pdlim7	PDZ and LIM domain 7	2.89
1435644_at	Sh3pxd2b	SH3 and PX domains 2B	2.88
1418240_at	Gbp2	guanylate nucleotide binding protein 2	2.87
1454768_at	Kcnf1	potassium voltage-gated channel, subfamily F, member 1	2.87
1439925_at	Tm4sf1	transmembrane 4 superfamily member 1	2.87
1451382_at	Chac1	ChaC, cation transport regulator-like 1 (E. coli)	2.86
1422120_at	Eaf2	ELL associated factor 2	2.84
1449141_at	Fblim1	filamin binding LIM protein 1	2.84
1417928_at	Pdlim4	PDZ and LIM domain 4	2.83
1434436_at	Morc4	microrchidia 4	2.83
1422818_at	Nedd9	neural precursor cell expressed, developmentally down-regulated gene 9	2.80
1452035_at	Col4a1	collagen, type IV, alpha 1	2.80
1416892_s_at	3110001A13Rik	RIKEN cDNA 3110001A13 gene	2.79
1450641_at	Vim	vimentin	2.79
1449184_at	Pglyrp1	peptidoglycan recognition protein 1	2.78
1415871_at	Tgfbi	transforming growth factor, beta induced	2.77

1452283_at	Rassf8	Ras association (RalGDS/AF-6) domain family 8	2.76
1439705_at	BB070754	Expressed sequence BB070754	2.76
1416184_s_at	Hmga1	high mobility group AT-hook 1	2.75
1424942_a_at	Myc	myelocytomatosis oncogene	2.75
1440621_at	---	Transcribed locus	2.75
1450744_at	Ell2	elongation factor RNA polymerase II 2	2.73
1435931_at	---	16 days neonate cerebellum cDNA, RIKEN full-length enriched library, clone:9630061A16 product:unclassifiable, full insert sequence	2.73
1441300_at	Kcnf1	potassium voltage-gated channel, subfamily F, member 1	2.72
1440657_at	---	---	2.72
1422622_at	Nos3	nitric oxide synthase 3, endothelial cell	2.72
1422537_a_at	Id2	inhibitor of DNA binding 2	2.70
1435367_at	Mapk4	mitogen-activated protein kinase 4	2.69
1452803_at	Glipr2	GLI pathogenesis-related 2	2.69
1416303_at	Litaf	LPS-induced TN factor	2.69
1449310_at	Ptger2	prostaglandin E receptor 2 (subtype EP2)	2.68
1450478_a_at	Ptpn12	protein tyrosine phosphatase, non-receptor type 12	2.68
1418250_at	Arl4d /// LOC100044157	ADP-ribosylation factor-like 4D /// hypothetical protein LOC100044157	2.68
1451458_at	Tmem2	transmembrane protein 2	2.67
1431225_at	Sox11	SRY-box containing gene 11	2.67
1455900_x_at	Tgm2	transglutaminase 2, C polypeptide	2.65
1419127_at	Npy	neuropeptide Y	2.65
1429759_at	Rps6ka6	ribosomal protein S6 kinase polypeptide 6	2.64
1449480_at	Sap18	Sin3-associated polypeptide 18	2.64
1418637_at	Etv3 /// LOC100045950	ets variant gene 3 /// similar to ets variant gene 3	2.64
1424751_at	Abt1	activator of basal transcription	2.61
1448688_at	Podxl	podocalyxin-like	2.60
1435484_at	Slc5a3	solute carrier family 5 (inositol transporters), member 3	2.59
1429444_at	Rasl11a	RAS-like, family 11, member A	2.59
1422045_a_at	Ptpn12	protein tyrosine phosphatase, non-receptor type 12	2.58
1434283_at	LOC100044968	similar to modulator recognition factor 2	2.57
1452217_at	Ahnak	AHNAK nucleoprotein (desmoyokin)	2.56
1452192_at	BC053440	cDNA sequence BC053440	2.56
1454018_at	Tlk2	tousled-like kinase 2 (Arabidopsis)	2.55
1455919_at	---	Transcribed locus	2.54
1440346_at	Jmjd3	Jumonji domain containing 3	2.54
1455372_at	Cpeb3	cytoplasmic polyadenylation element binding protein 3	2.54
1423584_at	Igfbp7	insulin-like growth factor binding protein 7	2.54
1437868_at	BC023892	cDNA sequence BC023892	2.52
1452398_at	Plce1	phospholipase C, epsilon 1	2.52
1448605_at	Rhoc	ras homolog gene family, member C	2.52
1424996_at	Cflar	CASP8 and FADD-like apoptosis regulator	2.52
1452157_at	Eprs /// LOC633677	glutamyl-prolyl-tRNA synthetase /// similar to Bifunctional aminoacyl-tRNA synthetase	2.51
1427893_a_at	Pmvk	phosphomevalonate kinase	2.51
1424440_at	Mrps6	mitochondrial ribosomal protein S6	2.51

1435701_at	---	13 days embryo lung cDNA, RIKEN full-length enriched library, clone:D430017B04 product:unclassifiable, full insert sequence	2.51
1424287_at	Prkx	protein kinase, X-linked	2.50
1460351_at	S100a11	S100 calcium binding protein A11 (calgizzarin)	2.50
1424051_at	Col4a2	collagen, type IV, alpha 2	2.50
1458588_at	---	---	2.50
1450685_at	Arpp19	cAMP-regulated phosphoprotein 19	2.49
1451299_at	Prkx	protein kinase, X-linked	2.49
1416304_at	Litaf	LPS-induced TN factor	2.47
1421392_a_at	Birc3	baculoviral IAP repeat-containing 3	2.47
1457983_s_at	Rwdd4a	RWD domain containing 4A	2.44
1433205_at	2810436B12Rik	RIKEN cDNA 2810436B12 gene	2.43
1428114_at	Slc14a1	solute carrier family 14 (urea transporter), member 1	2.42
1418135_at	Aff1	AF4/FMR2 family, member 1	2.42
1427165_at	Il13ra1	interleukin 13 receptor, alpha 1	2.41
1437451_at	1110006O17Rik	RIKEN cDNA 1110006O17 gene	2.41
1437166_at	Tinf2	Terf1 (TRF1)-interacting nuclear factor 2	2.41
1417562_at	Eif4ebp1	eukaryotic translation initiation factor 4E binding protein 1	2.41
1456381_x_at	Mcl1	myeloid cell leukemia sequence 1	2.40
1424271_at	Dclk1	doublecortin-like kinase 1	2.40
1416880_at	Mcl1	myeloid cell leukemia sequence 1	2.39
1438408_at	Ankrd56	ankyrin repeat domain 56	2.39
1421228_at	Ccl7	chemokine (C-C motif) ligand 7	2.38
1456292_a_at	Vim	vimentin	2.37
1449485_at	Ripk1	receptor (TNFRSF)-interacting serine-threonine kinase 1	2.36
1449164_at	Cd68	CD68 antigen	2.36
1430064_at	Ttll11	tubulin tyrosine ligase-like family, member 11	2.36
1460208_at	Fbn1	fibrillin 1	2.35
1418719_at	2410004L22Rik	RIKEN cDNA 2410004L22 gene	2.35
1416286_at	Rgs4	regulator of G-protein signaling 4	2.35
1417291_at	Tnfrsf1a	tumor necrosis factor receptor superfamily, member 1a	2.35
1448170_at	Siah2	seven in absentia 2	2.35
1417612_at	Ier5	immediate early response 5	2.35
1426870_at	Fbxo33	F-box protein 33	2.34
1421654_a_at	Lmna	lamin A	2.34
1416064_a_at	Hspa5	heat shock protein 5	2.33
1433748_at	LOC100046487 /// Zdhhc18	zinc finger, DHHC domain containing 18 /// similar to ribosomal protein L29	2.32
1460694_s_at	Svil	supervillin	2.32
1416226_at	Arpc1b	actin related protein 2/3 complex, subunit 1B	2.31
1436338_at	---	Adult male olfactory brain cDNA, RIKEN full-length enriched library, clone:6430546G07 product:unclassifiable, full insert sequence	2.31
1424065_at	Edem1	ER degradation enhancer, mannosidase alpha-like 1	2.30
1455994_x_at	Elovl1	elongation of very long chain fatty acids (FEN1/Elo2, SUR4/Elo3, yeast)-like 1	2.29
1424244_at	Rwdd4a	RWD domain containing 4A	2.28
1451201_s_at	Rnh1	ribonuclease/angiogenin inhibitor 1	2.27
1428942_at	Mt2	metallothionein 2	2.27
1422697_s_at	Jarid2	jumonji, AT rich interactive domain 2	2.27

1452358_at	Rai2	retinoic acid induced 2	2.27
1421756_a_at	Gpr19	G protein-coupled receptor 19	2.27
1433555_at	Eaf1	ELL associated factor 1	2.27
1435526_at	Tor1aip2	torsin A interacting protein 2	2.27
1417352_s_at	Snrpa1	small nuclear ribonucleoprotein polypeptide A'	2.26
1416554_at	LOC100048338 /// Pdlim1	PDZ and LIM domain 1 (elfin) /// similar to Pdlim1 protein	2.26
1460330_at	Anxa3	annexin A3	2.26
1420502_at	Sat1	spermidine/spermine N1-acetyl transferase 1	2.25
1435402_at	Gramd1b	GRAM domain containing 1B	2.24
1425503_at	Gcnt2	glucosaminyl (N-acetyl) transferase 2, I-branching enzyme	2.23
1454268_a_at	Cyba	cytochrome b-245, alpha polypeptide	2.23
1429863_at	Lonrf3	LON peptidase N-terminal domain and ring finger 3	2.23
1415802_at	Slc16a1	solute carrier family 16 (monocarboxylic acid transporters), member 1	2.22
1419047_at	Pcnx	pecanex homolog (Drosophila)	2.22
1420905_at	Il17ra	interleukin 17 receptor A	2.21
1422473_at	Pde4b	phosphodiesterase 4B, cAMP specific	2.21
1448596_at	Slc6a8	solute carrier family 6 (neurotransmitter transporter, creatine), member 8	2.21
1422506_a_at	Cstb	cystatin B	2.20
1460632_at	---	Transcribed locus	2.20
1417185_at	Ly6a	lymphocyte antigen 6 complex, locus A	2.20
1418296_at	Fxyd5	FXYD domain-containing ion transport regulator 5	2.20
1429564_at	LOC100048247 /// Pcgf5	polycomb group ring finger 5 /// similar to polycomb group ring finger 5	2.19
1417868_a_at	Ctsz	cathepsin Z	2.19
1453059_at	2310046A06Rik	RIKEN cDNA 2310046A06 gene	2.19
1417483_at	Nfkbiz	nuclear factor of kappa light polypeptide gene enhancer in B-cells inhibitor, zeta	2.19
1430353_at	Glis3	GLIS family zinc finger 3	2.19
1455200_at	Pak6	p21 (CDKN1A)-activated kinase 6	2.18
1431073_at	Ptar1	protein prenyltransferase alpha subunit repeat containing 1	2.18
1433582_at	1190002N15Rik /// LOC100044725	RIKEN cDNA 1190002N15 gene /// hypothetical protein LOC100044725	2.18
1426529_a_at	Tagln2	transgelin 2	2.18
1446860_at	---	---	2.18
1449901_a_at	Map3k6	mitogen-activated protein kinase kinase kinase 6	2.18
1449119_at	Arih2	ariadne homolog 2 (Drosophila)	2.18
1416543_at	Nfe2l2	nuclear factor, erythroid derived 2, like 2	2.17
1424594_at	Lgals7	lectin, galactose binding, soluble 7	2.15
1419444_at	LOC100041953 /// LOC100047209 /// Sap18	Sin3-associated polypeptide 18 /// similar to SAP18	2.15
1423233_at	Cebpd	CCAAT/enhancer binding protein (C/EBP), delta	2.15
1416458_at	Arf2	ADP-ribosylation factor 2	2.14
1437658_a_at	Snhg1	small nucleolar RNA host gene (non-protein coding) 1	2.13

1441823_at	Zmiz1	zinc finger, MIZ-type containing 1	2.13
1447585_s_at	Mrps6	mitochondrial ribosomal protein S6	2.13
1435926_at	Chml	choroideremia-like	2.13
1435803_a_at	Eif4e2	eukaryotic translation initiation factor 4E member 2	2.13
1436325_at	Rora	RAR-related orphan receptor alpha	2.13
1426724_at	Cnn3 /// LOC100047856	calponin 3, acidic /// similar to calponin 3, acidic	2.13
1449145_a_at	Cav1	caveolin, caveolae protein 1	2.12
1416101_a_at	Hist1h1c	histone cluster 1, H1c	2.12
1418031_at	Myo9b	myosin IXb	2.12
1418105_at	Stmn4	stathmin-like 4	2.12
1448694_at	Jun	Jun oncogene	2.12
1439862_at	Rorb	RAR-related orphan receptor beta	2.12
1425803_a_at	Mbd2	methyl-CpG binding domain protein 2	2.12
1415910_s_at	Ciapin1	cytokine induced apoptosis inhibitor 1	2.12
1456014_s_at	Fermt3	fermitin family homolog 3 (Drosophila)	2.11
1452783_at	Fndc3b	fibronectin type III domain containing 3B	2.11
1460735_at	Svil	supervillin	2.11
1420172_at	---	---	2.11
1418835_at	Phlda1	pleckstrin homology-like domain, family A, member 1	2.11
1455944_at	Zfp516	zinc finger protein 516	2.11
1460700_at	Stat3	signal transducer and activator of transcription 3	2.10
1449389_at	Tal1	T-cell acute lymphocytic leukemia 1	2.10
1416459_at	Arf2	ADP-ribosylation factor 2	2.10
1435636_at	2310051F07Rik	RIKEN cDNA 2310051F07 gene	2.10
1416435_at	Ltbr	lymphotoxin B receptor	2.09
1422573_at	Ampd3	AMP deaminase 3	2.09
1434646_s_at	LOC100041953 /// LOC100047209 /// Sap18	Sin3-associated polypeptide 18 /// similar to SAP18	2.08
1419445_s_at	LOC100041953 /// LOC100047209 /// Sap18	Sin3-associated polypeptide 18 /// similar to SAP18	2.07
1431339_a_at	Efhd2 /// LOC100048188	EF hand domain containing 2 /// similar to EF hand domain containing 2	2.07
1433696_at	Hn1l	hematological and neurological expressed 1-like	2.07
1451121_a_at	Gltscr2	glioma tumor suppressor candidate region gene 2	2.06
1444188_at	---	---	2.06
1448306_at	Nfkbia	nuclear factor of kappa light chain gene enhancer in B-cells inhibitor, alpha	2.06
1444180_at	---	12 days embryo spinal ganglion cDNA, RIKEN full-length enriched library, clone:D130055E11 product:unclassifiable, full insert sequence	2.05
1425053_at	Isoc1	isochorismatase domain containing 1	2.05
1422507_at	Cstb	cystatin B	2.05
1430089_at	5830469G19Rik	RIKEN cDNA 5830469G19 gene	2.04
1425305_at	Zfp295	zinc finger protein 295	2.04
1417869_s_at	Ctsz	cathepsin Z	2.04
1437766_at	---	---	2.03

1422615_at	Map4k4	mitogen-activated protein kinase kinase kinase kinase 4	2.03
1441437_at	---	---	2.03
1441578_at	Ccdc86	coiled-coil domain containing 86	2.03
1451208_at	Etf1	eukaryotic translation termination factor 1	2.02
1431065_at	Eaf1	ELL associated factor 1	2.02
1448935_at	Cdyl	chromodomain protein, Y chromosome-like	2.02
1417621_at	Nfatc1	nuclear factor of activated T-cells, cytoplasmic, calcineurin-dependent 1	2.01
1448419_at	Pop4	processing of precursor 4, ribonuclease P/MRP family, (S. cerevisiae)	2.01
1436100_at	Sh2d5	SH2 domain containing 5	2.01
1460239_at	Tspan13	tetraspanin 13	-2.00
1439975_at	BC062109	cDNA sequence BC062109	-2.00
1434140_at	Mcf2l	mcf.2 transforming sequence-like	-2.00
1435612_at	Opcml	opioid binding protein/cell adhesion molecule-like	-2.00
1456137_at	Nrxn3	neurexin III	-2.00
1450449_a_at	Rilpl1	Rab interacting lysosomal protein-like 1	-2.00
1433642_at	Arl15	ADP-ribosylation factor-like 15	-2.01
1431598_a_at	Lhx9	LIM homeobox protein 9	-2.01
1435993_at	OTTMUSG00000000421	predicted gene, OTTMUSG00000000421	-2.01
1436754_at	LOC100047659 /// Tmem179	transmembrane protein 179 /// hypothetical protein LOC100047659	-2.01
1418185_at	4733401H18Rik	RIKEN cDNA 4733401H18 gene	-2.01
1434655_at	Foxk1	forkhead box K1	-2.01
1440455_at	AI848599	expressed sequence AI848599	-2.01
1460081_at	Syt7	Synaptotagmin VII	-2.01
1457213_a_at	Dgkh	diacylglycerol kinase, eta	-2.01
1435239_at	Gria1	glutamate receptor, ionotropic, AMPA1 (alpha 1)	-2.01
1427186_a_at	LOC100047837	similar to Myocyte enhancer factor 2A	-2.01
1437774_at	1700020I14Rik	RIKEN cDNA 1700020I14 gene	-2.01
1440928_at	D630037F22Rik	RIKEN cDNA D630037F22 gene	-2.02
1435845_at	---	B16 F10Y cells cDNA, RIKEN full-length enriched library, clone:G370036C05 product:unclassifiable, full insert sequence	-2.02
1425833_a_at	Hpca	hippocalcin	-2.02
1421074_at	Cyp7b1	cytochrome P450, family 7, subfamily b, polypeptide 1	-2.02
1424474_a_at	Camkk2	calcium/calmodulin-dependent protein kinase kinase 2, beta	-2.02
1457671_at	9330120H11Rik	RIKEN cDNA 9330120H11 gene	-2.02
1454821_at	B3gat1	beta-1,3-glucuronyltransferase 1 (glucuronosyltransferase P)	-2.02
1449298_a_at	Pde1a	phosphodiesterase 1A, calmodulin-dependent	-2.03
1443379_at	LOC100048446	similar to RIKEN cDNA A430033K04 gene	-2.03
1433751_at	Slc39a10	solute carrier family 39 (zinc transporter), member 10	-2.03
1439610_at	Rab27b	RAB27b, member RAS oncogene family	-2.03
1434645_at	C530008M17Rik	RIKEN cDNA C530008M17 gene	-2.03
1418743_a_at	LOC100047138 /// Tesc	tescalcin /// similar to Tescalcin	-2.03
1417181_a_at	Kifap3	kinesin-associated protein 3	-2.03
1460675_at	Igsf8	immunoglobulin superfamily, member 8	-2.03

1455868_a_at	Tubgcp2	tubulin, gamma complex associated protein 2	-2.03
1438580_at	Zcchc7	zinc finger, CCHC domain containing 7	-2.03
1421928_at	Epha4	Eph receptor A4	-2.04
1429215_at	2310058N22Rik	RIKEN cDNA 2310058N22 gene	-2.04
1446382_at	---	0 day neonate cerebellum cDNA, RIKEN full-length enriched library, clone:C230094E06 product:unclassifiable, full insert sequence	-2.04
1456423_at	Mbd5	methyl-CpG binding domain protein 5	-2.04
1455194_at	Mapk8ip2	mitogen-activated protein kinase 8 interacting protein 2	-2.04
1421862_a_at	Vamp1	vesicle-associated membrane protein 1	-2.04
1460570_at	Pgbd5	piggyBac transposable element derived 5	-2.05
1422126_a_at	Nudt13	nudix (nucleoside diphosphate linked moiety X)-type motif 13	-2.05
1448676_at	Camk2b	calcium/calmodulin-dependent protein kinase II, beta	-2.05
1428240_at	Nrxn1	neurexin I	-2.05
1428647_at	LOC676870 /// Pbx1	pre B-cell leukemia transcription factor 1 /// region containing RIKEN cDNA 2310056B04 gene; pre B-cell leukemia transcription factor 1	-2.05
1434295_at	Rasgrp1	RAS guanyl releasing protein 1	-2.05
1427349_x_at	2810021G02Rik	RIKEN cDNA 2810021G02 gene	-2.05
1428748_at	5830428H23Rik	RIKEN cDNA 5830428H23 gene	-2.05
1451094_at	Ggtl3	gamma-glutamyltransferase-like 3	-2.05
1435767_at	Scn3b	sodium channel, voltage-gated, type III, beta	-2.05
1445766_at	---	---	-2.05
1433602_at	Gabra5	gamma-aminobutyric acid (GABA-A) receptor, subunit alpha 5	-2.06
1419664_at	Srr	serine racemase	-2.06
1418400_at	Larp6	La ribonucleoprotein domain family, member 6	-2.06
1435842_at	Nat8l	N-acetyltransferase 8-like	-2.07
1425514_at	Pik3r1	phosphatidylinositol 3-kinase, regulatory subunit, polypeptide 1 (p85 alpha)	-2.07
1424415_s_at	Spon1	spondin 1, (f-spondin) extracellular matrix protein	-2.07
1444046_at	D430041B17Rik	RIKEN cDNA D430041B17 gene	-2.07
1441815_at	AI851453	expressed sequence AI851453	-2.07
1416023_at	Fabp3	fatty acid binding protein 3, muscle and heart	-2.07
1450123_at	Ryr2	ryanodine receptor 2, cardiac	-2.07
1452353_at	Gpr155	G protein-coupled receptor 155	-2.07
1455526_at	Diras1	DIRAS family, GTP-binding RAS-like 1	-2.08
1441967_at	Pddc1	Parkinson disease 7 domain containing 1	-2.08
1434497_at	4933431E20Rik	RIKEN cDNA 4933431E20 gene	-2.08
1427012_at	Lancl1	LanC (bacterial lantibiotic synthetase component C)-like 1	-2.08
1434531_at	Mgat5b	mannoside acetylglucosaminyltransferase 5, isoenzyme B	-2.08
1457137_at	---	Transcribed locus	-2.08
1438473_at	Arl15	ADP-ribosylation factor-like 15	-2.08
1445676_at	---	0 day neonate cerebellum cDNA, RIKEN full-length enriched library, clone:C230043A04 product:unclassifiable, full insert sequence	-2.09
1427754_a_at	Dnm1	dynamin 1	-2.09
1434956_at	Rnf170	ring finger protein 170	-2.09
1421446_at	Prkcc	protein kinase C, gamma	-2.09
1429189_at	Arsb	arylsulfatase B	-2.09

1424464_s_at	2210010L05Rik	RIKEN cDNA 2210010L05 gene	-2.09
1442786_s_at	Rufy3	RUN and FYVE domain containing 3	-2.09
1435796_at	---	---	-2.09
1450202_at	Grin1	glutamate receptor, ionotropic, NMDA1 (zeta 1)	-2.10
1436450_at	D11Bwg0517e	DNA segment, Chr 11, Brigham & Women's Genetics 0517 expressed	-2.10
1415698_at	Golm1	golgi membrane protein 1	-2.10
1441768_at	9430051O21Rik	RIKEN cDNA 9430051O21 gene	-2.10
1449468_at	St6galnac5	ST6 (alpha-N-acetyl-neuraminyl-2,3-beta-galactosyl-1,3)-N-acetylgalactosaminide alpha-2,6-sialyltransferase 5	-2.10
1452050_at	Camk1d	calcium/calmodulin-dependent protein kinase ID	-2.10
1428447_at	Tmem14a	transmembrane protein 14A	-2.11
1438635_x_at	B930041F14Rik	RIKEN cDNA B930041F14 gene	-2.11
1454926_at	Sphkap	SPHK1 interactor, AKAP domain containing	-2.11
1440630_at	---	Adult male olfactory brain cDNA, RIKEN full-length enriched library, clone:6430514A06 product:unclassifiable, full insert sequence	-2.11
1438417_at	Pwwp2b	PWWP domain containing 2B	-2.12
1418746_at	Pnkd	paroxysmal nonkinesiogenic dyskinesia	-2.12
1457324_at	---	Transcribed locus	-2.12
1433577_at	A730017C20Rik	RIKEN cDNA A730017C20 gene	-2.12
1428669_at	Bmyc	brain expressed myelocytomatosis oncogene	-2.12
1440343_at	Rps6ka5	ribosomal protein S6 kinase, polypeptide 5	-2.12
1433596_at	Dnajc6	DnaJ (Hsp40) homolog, subfamily C, member 6	-2.12
1445837_at	---	12 days embryo spinal ganglion cDNA, RIKEN full-length enriched library, clone:D130073P12 product:unclassifiable, full insert sequence	-2.12
1442393_at	---	---	-2.12
1435260_at	---	---	-2.13
1434062_at	Rabgap1l	RAB GTPase activating protein 1-like	-2.13
1448063_at	Iqsec2	IQ motif and Sec7 domain 2	-2.13
1444008_at	LOC100039284	similar to TBP-associated factor 4	-2.13
1452848_at	Tmem181	transmembrane protein 181	-2.13
1429071_at	Me3	malic enzyme 3, NADP(+)-dependent, mitochondrial	-2.14
1428813_a_at	Drd1ip	dopamine receptor D1 interacting protein	-2.14
1452958_at	Asphd2	aspartate beta-hydroxylase domain containing 2	-2.14
1417664_a_at	Ndrg3	N-myc downstream regulated gene 3	-2.14
1453253_a_at	Rpusd1	RNA pseudouridylate synthase domain containing 1	-2.14
1439042_at	Adcyap1r1	adenylate cyclase activating polypeptide 1 receptor 1	-2.14
1428156_at	Gng2	guanine nucleotide binding protein (G protein), gamma 2 subunit	-2.15
1424403_a_at	Rufy3	RUN and FYVE domain containing 3	-2.16
1434763_at	Orai2	ORAI calcium release-activated calcium modulator 2	-2.16
1452825_at	Tmem59l	transmembrane protein 59-like	-2.16
1452251_at	Nbea	neurobeachin	-2.16
1453468_at	4930430F08Rik	RIKEN cDNA 4930430F08 gene	-2.16
1455272_at	Grm5	glutamate receptor, metabotropic 5	-2.16

1420876_a_at	6-Sep	septin 6	-2.16
1436548_at	1810012P15Rik	RIKEN cDNA 1810012P15 gene	-2.16
1422955_at	Syt17	synaptotagmin XVII	-2.17
1447211_at	Nrip1	nuclear receptor interacting protein 1	-2.17
1452763_at	Nipa1	non imprinted in Prader-Willi/Angelman syndrome 1 homolog (human)	-2.17
1419978_s_at	D10Ertd610e	DNA segment, Chr 10, ERATO Doi 610, expressed	-2.17
1457284_at	---	CDNA clone IMAGE:5256843	-2.17
1435961_at	Nat14	N-acetyltransferase 14	-2.18
1422197_at	Kcna2	potassium voltage-gated channel, shaker-related subfamily, member 2	-2.18
1449563_at	Cntn1	contactin 1	-2.19
1435501_at	Atg2b	ATG2 autophagy related 2 homolog B (S. cerevisiae)	-2.19
1429113_at	1500031I19Rik	RIKEN cDNA 1500031I19 gene	-2.19
1439086_at	A930009L07Rik	RIKEN cDNA A930009L07 gene	-2.19
1459107_at	Kcnh3	potassium voltage-gated channel, subfamily H (eag-related), member 3	-2.19
1441936_x_at	4930447M23Rik	RIKEN cDNA 4930447M23 gene	-2.19
1428525_at	4930488B01Rik	RIKEN cDNA 4930488B01 gene	-2.20
1426616_at	Tlcd1	TLC domain containing 1	-2.20
1452938_at	Anks1b	ankyrin repeat and sterile alpha motif domain containing 1B	-2.20
1434445_at	D15Wsu169e	DNA segment, Chr 15, Wayne State University 169, expressed	-2.20
1457423_at	LOC675405 /// Nalcn	sodium leak channel, non-selective /// similar to voltage gated channel like 1	-2.20
1443205_at	D5Buc30e	DNA segment, Chr 5, Bucan 30 expressed	-2.21
1452973_at	Ppm1k	protein phosphatase 1K (PP2C domain containing)	-2.21
1424037_at	Itpka	inositol 1,4,5-trisphosphate 3-kinase A	-2.21
1438730_at	BC028801	cDNA sequence BC028801	-2.22
1460101_at	---	---	-2.22
1457077_at	---	2 days neonate sympathetic ganglion cDNA, RIKEN full-length enriched library, clone:7120457A19 product:unclassifiable, full insert sequence	-2.22
1434653_at	Ptk2b	PTK2 protein tyrosine kinase 2 beta	-2.22
1439616_at	---	Transcribed locus	-2.22
1427951_s_at	Ccdc28a /// LOC100045472	coiled-coil domain containing 28A /// similar to coiled-coil domain containing 28A	-2.23
1435668_at	4932442K08Rik	RIKEN cDNA 4932442K08 gene	-2.23
1423559_at	Kcnc1	potassium voltage gated channel, Shaw-related subfamily, member 1	-2.23
1452056_s_at	Ppp3ca	protein phosphatase 3, catalytic subunit, alpha isoform	-2.23
1451569_at	Nr2c2	nuclear receptor subfamily 2, group C, member 2	-2.23
1460403_at	Psip1	PC4 and SFRS1 interacting protein 1	-2.23
1443824_s_at	Car7	carbonic anhydrase 7	-2.23
1435134_at	Aadacl1	arylacetamide deacetylase-like 1	-2.23
1457223_at	---	---	-2.24
1435095_at	C030009O12Rik	RIKEN cDNA C030009O12 gene	-2.24
1435404_at	Disp2	dispatched homolog 2 (Drosophila)	-2.24
1459051_at	6530418L21Rik	RIKEN cDNA 6530418L21 gene	-2.24

1434298_at	Zeb2	zinc finger E-box binding homeobox 2	-2.25
1452876_x_at	2610044O15Rik /// LOC100044296	RIKEN cDNA 2610044O15 gene /// hypothetical protein LOC100044296	-2.25
1453734_at	Atrx	alpha thalassemia/mental retardation syndrome X-linked homolog (human)	-2.25
1421075_s_at	Cyp7b1	cytochrome P450, family 7, subfamily b, polypeptide 1	-2.25
1440056_at	---	Adult male hippocampus cDNA, RIKEN full-length enriched library, clone:C630001C05 product:unclassifiable, full insert sequence	-2.25
1455410_at	Faim2	Fas apoptotic inhibitory molecule 2	-2.25
1433787_at	Nell1	NEL-like 1 (chicken)	-2.26
1436875_at	Dnm3	dynamin 3	-2.26
1433040_at	2900018E21Rik	RIKEN cDNA 2900018E21 gene	-2.26
1435417_at	AI464131	expressed sequence AI464131	-2.26
1443744_at	---	Transcribed locus	-2.26
1429201_at	Cyld	cylindromatosis (turban tumor syndrome)	-2.26
1419324_at	Lhx9	LIM homeobox protein 9	-2.26
1426339_at	Ak5	adenylate kinase 5	-2.26
1420872_at	Gucy1b3	guanylate cyclase 1, soluble, beta 3	-2.26
1420545_a_at	Chn1	chimerin (chimaerin) 1	-2.26
1435933_at	Scn2a1	sodium channel, voltage-gated, type II, alpha 1	-2.27
1419200_at	Fxyd7	FXYD domain-containing ion transport regulator 7	-2.27
1449634_a_at	Anks1b	ankyrin repeat and sterile alpha motif domain containing 1B	-2.27
1416586_at	Zfp239	zinc finger protein 239	-2.27
1417653_at	Pvalb	parvalbumin	-2.27
1428568_at	B230217C12Rik	RIKEN cDNA B230217C12 gene	-2.28
1425580_a_at	Pik3c3	phosphoinositide-3-kinase, class 3	-2.28
1428375_at	4932415G12Rik	RIKEN cDNA 4932415G12 gene	-2.28
1449620_s_at	D16Wsu65e	DNA segment, Chr 16, Wayne State University 65, expressed	-2.28
1452346_at	B3gnt1	UDP-GlcNAc:betaGal beta-1,3-N-acetylglucosaminyltransferase 1	-2.28
1455647_at	Ar	androgen receptor	-2.28
1431751_a_at	Mpped2	metallophosphoesterase domain containing 2	-2.29
1442614_at	Il1rap	interleukin 1 receptor accessory protein	-2.29
1441069_at	Zdhhc23	zinc finger, DHHC domain containing 23	-2.29
1456357_at	A930041I02Rik	RIKEN cDNA A930041I02 gene	-2.29
1454782_at	Bai3	brain-specific angiogenesis inhibitor 3	-2.30
1428207_at	Bcl7a	B-cell CLL/lymphoma 7A	-2.30
1440376_at	Fbxo41	F-box protein 41	-2.30
1429104_at	Limd2	LIM domain containing 2	-2.30
1452474_a_at	Art3	ADP-ribosyltransferase 3	-2.30
1438641_x_at	1500016O10Rik	RIKEN cDNA 1500016O10 gene	-2.30
1419401_at	Asb13	ankyrin repeat and SOCS box-containing protein 13	-2.31
1439181_at	BC043301	cDNA sequence BC043301	-2.31
1416388_at	Pip4k2c	phosphatidylinositol-5-phosphate 4-kinase, type II, gamma	-2.31
1433110_at	5830474E16Rik	RIKEN cDNA 5830474E16 gene	-2.32
1448312_at	Pcsk2	proprotein convertase subtilisin/kexin type 2	-2.32
1420871_at	Gucy1b3	guanylate cyclase 1, soluble, beta 3	-2.32
1460385_a_at	Zfp179	zinc finger protein 179	-2.33

1454941_at	LOC100045684	similar to N-myristoyltransferase 1	-2.33
1429225_at	Slc24a2	solute carrier family 24 (sodium/potassium/calcium exchanger), member 2	-2.33
1453994_at	C230094A16Rik	RIKEN cDNA C230094A16 gene	-2.33
1450121_at	Scn1a	sodium channel, voltage-gated, type I, alpha	-2.33
1455700_at	Mterfd3	MTERF domain containing 3	-2.33
1418478_at	Lmo1	LIM domain only 1	-2.33
1418840_at	Pdcd4	programmed cell death 4	-2.34
1451040_at	Dtd1 /// LOC100048650	D-tyrosyl-tRNA deacylase 1 homolog (S. cerevisiae) /// similar to D-tyrosyl-tRNA deacylase 1	-2.34
1418683_at	Lin7b	lin-7 homolog B (C. elegans)	-2.34
1439569_at	Gpr83	G protein-coupled receptor 83	-2.34
1451268_at	Tram1l1	translocation associated membrane protein 1-like 1	-2.34
1439959_at	Fgf11	fibroblast growth factor 11	-2.35
1433992_at	Shroom2	shroom family member 2	-2.35
1417050_at	C1qtnf4	C1q and tumor necrosis factor related protein 4	-2.35
1436602_x_at	Cacna1b	calcium channel, voltage-dependent, N type, alpha 1B subunit	-2.35
1455325_at	Rncr2	Retinal noncoding RNA 2	-2.35
1438423_at	Ssbp2	single-stranded DNA binding protein 2	-2.36
1428623_at	Plxna1	plexin A1	-2.36
1429881_at	Arhgap15	Rho GTPase activating protein 15	-2.36
1425691_at	B3gat1	beta-1,3-glucuronyltransferase 1 (glucuronosyltransferase P)	-2.36
1416959_at	Nr1d2	nuclear receptor subfamily 1, group D, member 2	-2.36
1435959_at	Arhgap15	Rho GTPase activating protein 15	-2.37
1434476_at	Crtc1	CREB regulated transcription coactivator 1	-2.37
1442032_at	BC030500	cDNA sequence BC030500	-2.37
1445631_at	Tmem16c	Transmembrane protein 16C	-2.37
1426328_a_at	Scn3b	sodium channel, voltage-gated, type III, beta	-2.37
1436066_at	Kalrn	kalirin, RhoGEF kinase	-2.37
1427281_at	Scn2a1	sodium channel, voltage-gated, type II, alpha 1	-2.38
1450143_at	Rasgrp1	RAS guanyl releasing protein 1	-2.38
1438262_at	Slc8a2	solute carrier family 8 (sodium/calcium exchanger), member 2	-2.38
1428157_at	Gng2	guanine nucleotide binding protein (G protein), gamma 2 subunit	-2.39
1419757_at	Pitpnm2	phosphatidylinositol transfer protein, membrane-associated 2	-2.39
1439497_at	4933415E08Rik	RIKEN cDNA 4933415E08 gene	-2.39
1438784_at	Bcl11b	B-cell leukemia/lymphoma 11B	-2.39
1429021_at	Epha4	Eph receptor A4	-2.39
1441963_at	RP23-100C5.8	ProSAPiP1 protein	-2.40
1424223_at	1700020C11Rik	RIKEN cDNA 1700020C11 gene	-2.40
1438217_at	A2bp1	ataxin 2 binding protein 1	-2.41
1426917_s_at	Scrn3	secernin 3	-2.41
1435033_at	Arhgef4	Rho guanine nucleotide exchange factor (GEF) 4	-2.41
1441780_at	---	Transcribed locus	-2.41
1435991_at	Nr3c2	nuclear receptor subfamily 3, group C, member 2	-2.42

1457587_at	Kcnq5	potassium voltage-gated channel, subfamily Q, member 5	-2.42
1424100_s_at	Cend1	cell cycle exit and neuronal differentiation 1	-2.42
1433791_at	Rab9b	RAB9B, member RAS oncogene family	-2.42
1452298_a_at	Myo5b	myosin Vb	-2.42
1434064_at	Orai3	ORAI calcium release-activated calcium modulator 3	-2.43
1448468_a_at	Kcnab1	potassium voltage-gated channel, shaker-related subfamily, beta member 1	-2.43
1429211_at	2900078E11Rik /// Cadm2	RIKEN cDNA 2900078E11 gene /// cell adhesion molecule 2	-2.43
1455447_at	D430019H16Rik	RIKEN cDNA D430019H16 gene	-2.43
1438752_at	A230058F20Rik	RIKEN cDNA A230058F20 gene	-2.43
1455426_at	Epha3	Eph receptor A3	-2.43
1456393_at	2310002J21Rik	RIKEN cDNA 2310002J21 gene	-2.43
1441662_at	Cyp4x1	cytochrome P450, family 4, subfamily x, polypeptide 1	-2.44
1422539_at	Extl2	exotoses (multiple)-like 2	-2.44
1434638_at	---	Adult male corpora quadrigemina cDNA, RIKEN full-length enriched library, clone:B230036G05 product:unclassifiable, full insert sequence	-2.44
1433826_at	Tspyl3	TSPY-like 3	-2.44
1426951_at	Crim1	cysteine rich transmembrane BMP regulator 1 (chordin like)	-2.45
1432294_at	9330177L23Rik	RIKEN cDNA 9330177L23 gene	-2.45
1438531_at	A730054J21Rik	RIKEN cDNA A730054J21 gene	-2.45
1445503_at	Gm715	gene model 715, (NCBI)	-2.45
1429443_at	Cpne4	copine IV	-2.46
1417569_at	Ncald	neurocalcin delta	-2.46
1427974_s_at	Cacna1d	calcium channel, voltage-dependent, L type, alpha 1D subunit	-2.46
1457990_at	Anks1b	ankyrin repeat and sterile alpha motif domain containing 1B	-2.47
1420660_at	Lrrc6	leucine rich repeat containing 6 (testis)	-2.47
1437422_at	Sema5a	sema domain, seven thrombospondin repeats (type 1 and type 1-like), transmembrane domain (TM) and short cytoplasmic domain, (semaphorin) 5A	-2.47
1420563_at	Gria3	glutamate receptor, ionotropic, AMPA3 (alpha 3)	-2.47
1438310_at	---	10 days neonate cerebellum cDNA, RIKEN full-length enriched library, clone:B930095L19 product:unclassifiable, full insert sequence	-2.48
1451840_at	Kcnip4	Kv channel interacting protein 4	-2.48
1437168_at	Srrp	serine-arginine repressor protein	-2.48
1434728_at	Gria3	glutamate receptor, ionotropic, AMPA3 (alpha 3)	-2.48
1436148_at	---	Adult male olfactory brain cDNA, RIKEN full-length enriched library, clone:6430531K17 product:unclassifiable, full insert sequence	-2.48
1431216_s_at	Dnajc6	DnaJ (Hsp40) homolog, subfamily C, member 6	-2.49
1441305_at	---	Adult male corpus striatum cDNA, RIKEN full-length enriched library, clone:C030044L04 product:unclassifiable, full insert sequence	-2.49

1440030_at	---	---	-2.49
1452952_at	9030418K01Rik	RIKEN cDNA 9030418K01 gene	-2.49
1456202_at	Elfn2	leucine rich repeat and fibronectin type III, extracellular 2	-2.49
1439106_at	Zfp462	zinc finger protein 462	-2.50
1450370_a_at	Kcnip4	Kv channel interacting protein 4	-2.50
1435227_at	Bcl11b	B-cell leukemia/lymphoma 11B	-2.51
1429269_at	BC068157	cDNA sequence BC068157	-2.51
1427086_at	Slit3	slit homolog 3 (Drosophila)	-2.52
1425749_at	Stxbp6	syntaxin binding protein 6 (amisyn)	-2.52
1425277_at	Slit1	slit homolog 1 (Drosophila)	-2.52
1427286_at	D11Bwg0517e	DNA segment, Chr 11, Brigham & Women's Genetics 0517 expressed	-2.53
1456119_at	Grm5	glutamate receptor, metabotropic 5	-2.53
1448083_at	LOC675405 /// Nalcn	sodium leak channel, non-selective /// similar to voltage gated channel like 1	-2.53
1448664_a_at	Speg	SPEG complex locus	-2.53
1442371_at	Lmbr1	limb region 1	-2.54
1417702_a_at	Hnmt	histamine N-methyltransferase	-2.54
1435115_at	Fndc5	fibronectin type III domain containing 5	-2.54
1436411_at	Atp13a5	ATPase type 13A5	-2.54
1447788_s_at	Tspyl3	TSPY-like 3	-2.55
1435285_at	Mpped2	metallophosphoesterase domain containing 2	-2.55
1453053_at	2610036L11Rik	RIKEN cDNA 2610036L11 gene	-2.55
1455258_at	Kcnc2	potassium voltage gated channel, Shaw-related subfamily, member 2	-2.56
1449172_a_at	Lin7b	lin-7 homolog B (C. elegans)	-2.56
1418619_at	Icam5	intercellular adhesion molecule 5, telencephalin	-2.56
1434271_at	Gba2	glucosidase beta 2	-2.56
1420403_at	Atp2b2	ATPase, Ca++ transporting, plasma membrane 2	-2.56
1415935_at	Smoc2	SPARC related modular calcium binding 2	-2.56
1434973_at	Car7	carbonic anhydrase 7	-2.57
1419442_at	Matn2	matrilin 2	-2.57
1456377_x_at	Limd2 /// LOC632329	LIM domain containing 2 /// similar to epithelial protein lost in neoplasm	-2.57
1435957_at	B830032F12	hypothetical protein B830032F12	-2.57
1436275_at	Kcnip2	Kv channel-interacting protein 2	-2.57
1443327_at	D130043K22Rik	RIKEN cDNA D130043K22 gene	-2.58
1426729_at	2900046G09Rik	RIKEN cDNA 2900046G09 gene	-2.58
1422748_at	Zeb2	zinc finger E-box binding homeobox 2	-2.59
1429105_at	Dlgap1	discs, large (Drosophila) homolog-associated protein 1	-2.61
1442370_at	---	10 days neonate cortex cDNA, RIKEN full-length enriched library, clone:A830028E23 product:unclassifiable, full insert sequence	-2.61
1455358_at	A2bp1	Ataxin 2 binding protein 1	-2.61
1433711_s_at	LOC100047324 /// Sesn1	sestrin 1 /// similar to Sesn1 protein	-2.61
1456089_at	Trim23	tripartite motif protein 23	-2.61
1434015_at	Slc2a6	solute carrier family 2 (facilitated glucose transporter), member 6	-2.61
1440910_at	C77370	expressed sequence C77370	-2.62
1444723_at	6530418L21Rik	RIKEN cDNA 6530418L21 gene	-2.62
1429447_at	Evc2	Ellis van Creveld syndrome 2 homolog (human)	-2.63

1440698_at	---	---	-2.64
1444693_at	---	---	-2.64
1431569_a_at	Lypd1	Ly6/Plaur domain containing 1	-2.65
1436961_at	Hspa12a	heat shock protein 12A	-2.65
1438540_at	Col25a1	collagen, type XXV, alpha 1	-2.65
1434535_at	Krt222	keratin 222	-2.65
1451678_at	Narf	nuclear prelamin A recognition factor	-2.65
1430237_at	Cldn22	claudin 22	-2.66
1447992_s_at	Pcsk2	proprotein convertase subtilisin/kexin type 2	-2.66
1426389_at	Camk1d	calcium/calmodulin-dependent protein kinase ID	-2.67
1451235_at	Cend1	cell cycle exit and neuronal differentiation 1	-2.67
1444077_at	---	Transcribed locus	-2.67
1455145_at	Pcdh19	protocadherin 19	-2.68
1423640_at	Synpr	synaptoporin	-2.70
1439289_s_at	Pnmal1	PNMA-like 1	-2.70
1460588_at	---	10 days lactation, adult female mammary gland cDNA, RIKEN full-length enriched library, clone:D730013G10 product:unclassifiable, full insert sequence	-2.70
1440681_at	Chrna7	cholinergic receptor, nicotinic, alpha polypeptide 7	-2.71
1421348_a_at	Cend1	cell cycle exit and neuronal differentiation 1	-2.71
1443855_at	Kcnc1	potassium voltage gated channel, Shaw-related subfamily, member 1	-2.71
1445691_at	Chn1	chimerin (chimaerin) 1	-2.72
1442701_at	---	---	-2.75
1441801_at	Kctd4	potassium channel tetramerisation domain containing 4	-2.75
1449312_at	Npy5r	neuropeptide Y receptor Y5	-2.75
1452456_at	Nrip2	nuclear receptor interacting protein 2	-2.76
1434051_s_at	Hspa12a	heat shock protein 12A	-2.77
1421176_at	Rasgrp1	RAS guanyl releasing protein 1	-2.77
1459563_x_at	---	---	-2.78
1419811_at	D16Wsu65e	DNA segment, Chr 16, Wayne State University 65, expressed	-2.78
1458114_at	Samd12	sterile alpha motif domain containing 12	-2.79
1431749_a_at	Rasgrp1	RAS guanyl releasing protein 1	-2.80
1428936_at	Atp2b1	ATPase, Ca++ transporting, plasma membrane 1	-2.80
1442019_at	B230343A10Rik	RIKEN cDNA B230343A10 gene	-2.81
1454969_at	Lypd6	LY6/PLAUR domain containing 6	-2.81
1439056_at	EG636791	predicted gene, EG636791	-2.81
1447385_at	---	Adult male corpora quadrigemina cDNA, RIKEN full-length enriched library, clone:B230036G05 product:unclassifiable, full insert sequence	-2.81
1429702_at	2900072G11Rik	RIKEN cDNA 2900072G11 gene	-2.81
1440589_at	---	Transcribed locus	-2.82
1437147_at	Gabrg2	gamma-aminobutyric acid (GABA-A) receptor, subunit gamma 2	-2.82
1422925_s_at	Acot3	acyl-CoA thioesterase 3	-2.82
1439240_x_at	Lin7b	lin-7 homolog B (C. elegans)	-2.82
1455373_at	Pclo	piccolo (presynaptic cytomatrix protein)	-2.83
1436088_at	0910001A06Rik	RIKEN cDNA 0910001A06 gene	-2.83
1456953_at	Col19a1	collagen, type XIX, alpha 1	-2.83

1434776_at	Sema5a	sema domain, seven thrombospondin repeats (type 1 and type 1-like), transmembrane domain (TM) and short cytoplasmic domain, (semaphorin) 5A	-2.83
1455374_at	Kcnj3	potassium inwardly-rectifying channel, subfamily J, member 3	-2.83
1448477_at	Chst12	carbohydrate sulfotransferase 12	-2.84
1418452_at	Gng2	guanine nucleotide binding protein (G protein), gamma 2 subunit	-2.85
1439757_s_at	Epha4	Eph receptor A4	-2.85
1460674_at	Paqr7	progestin and adipoQ receptor family member VII	-2.85
1455960_at	Megf9	multiple EGF-like-domains 9	-2.86
1421969_a_at	Faah	fatty acid amide hydrolase	-2.87
1439697_at	Il1rap	interleukin 1 receptor accessory protein	-2.87
1443612_at	Tmem16c	Transmembrane protein 16C	-2.87
1441927_at	Syt7	Synaptotagmin VII	-2.88
1433885_at	Iqgap2	IQ motif containing GTPase activating protein 2	-2.90
1418314_a_at	A2bp1	ataxin 2 binding protein 1	-2.90
1440438_at	---	Transcribed locus	-2.91
1434454_at	D16Wsu65e	DNA segment, Chr 16, Wayne State University 65, expressed	-2.91
1437631_at	Kcnip4	Kv channel interacting protein 4	-2.92
1452966_at	Bcl11b	B-cell leukemia/lymphoma 11B	-2.92
1440849_at	6330417G04Rik	RIKEN cDNA 6330417G04 gene	-2.93
1437750_at	Tmem158	Transmembrane protein 158	-2.94
1429918_at	Arhgap20	Rho GTPase activating protein 20	-2.95
1438710_at	Htr1a	5-hydroxytryptamine (serotonin) receptor 1A	-2.96
1453424_at	Fyco1	FYVE and coiled-coil domain containing 1	-2.96
1443790_x_at	4930414L22Rik	RIKEN cDNA 4930414L22 gene	-2.97
1450486_a_at	Oprl1	opioid receptor-like 1	-2.98
1427646_a_at	Arhgef2	rho/rac guanine nucleotide exchange factor (GEF) 2	-2.99
1429402_at	Glt8d2	glycosyltransferase 8 domain containing 2	-3.01
1431229_at	Ipw	imprinted gene in the Prader-Willi syndrome region	-3.02
1433681_x_at	Capn3	calpain 3	-3.02
1444687_at	C1ql2	complement component 1, q subcomponent-like 2	-3.02
1445234_at	C130030K03Rik	RIKEN cDNA C130030K03 gene	-3.04
1446632_at	---	---	-3.04
1417391_a_at	Il16	interleukin 16	-3.04
1454043_a_at	Kcnab1	potassium voltage-gated channel, shaker-related subfamily, beta member 1	-3.06
1453756_at	2900075N08Rik	RIKEN cDNA 2900075N08 gene	-3.06
1423305_at	Extl1	exostoses (multiple)-like 1	-3.08
1456798_at	9330118A15Rik	RIKEN cDNA 9330118A15 gene	-3.08
1452065_at	Vstm2a	V-set and transmembrane domain containing 2A	-3.08
1457072_at	Bcl11a /// Ppfia3	Protein tyrosine phosphatase, receptor type, f polypeptide (PTPRF), interacting protein (liprin), alpha 3 /// B-cell CLL/lymphoma 11A (zinc finger protein)	-3.09
1451525_at	Arhgap12	Rho GTPase activating protein 12	-3.11
1422710_a_at	Cacna1h	calcium channel, voltage-dependent, T type, alpha 1H subunit	-3.11

1423270_at	Nedd4l	neural precursor cell expressed, developmentally down-regulated gene 4-like	-3.11
1435135_at	Aadacl1	arylacetamide deacetylase-like 1	-3.12
1428305_at	Pcsk2	proprotein convertase subtilisin/kexin type 2	-3.12
1427610_at	Dsp	desmoplakin	-3.13
1430952_at	3110039C02Rik	RIKEN cDNA 3110039C02 gene	-3.16
1455365_at	Cdh8	cadherin 8	-3.16
1431052_at	Arhgap12	Rho GTPase activating protein 12	-3.16
1439870_at	A330008L17Rik	RIKEN cDNA A330008L17 gene	-3.19
1457843_at	Lypd6	LY6/PLAUR domain containing 6	-3.19
1449173_at	Mpp2	membrane protein, palmitoylated 2 (MAGUK p55 subfamily member 2)	-3.20
1460625_at	Gm1568	gene model 1568, (NCBI)	-3.25
1421318_at	LOC100047238	similar to N-deacetylase/N-sulfotransferase 4	-3.26
1457092_at	C630007B19Rik	RIKEN cDNA C630007B19 gene	-3.27
1423201_at	Ncor1	nuclear receptor co-repressor 1	-3.28
1425344_at	Narf	nuclear prelamin A recognition factor	-3.28
1457354_at	Krt222	keratin 222	-3.29
1437183_at	LOC100045241 /// Lrrc4b	leucine rich repeat containing 4B /// similar to Leucine rich repeat containing 4B	-3.29
1440181_at	Gm1568	gene model 1568, (NCBI)	-3.31
1446364_at	---	---	-3.33
1448530_at	Gmpr /// LOC100045393	guanosine monophosphate reductase /// similar to guanosine monophosphate reductase	-3.34
1419406_a_at	Bcl11a	B-cell CLL/lymphoma 11A (zinc finger protein)	-3.34
1439947_at	Cyp11a1	cytochrome P450, family 11, subfamily a, polypeptide 1	-3.35
1429183_at	Pkp2	plakophilin 2	-3.35
1455772_at	Pgr	progesterone receptor	-3.36
1439699_at	---	15 days embryo head cDNA, RIKEN full-length enriched library, clone:D930025A16 product:unclassifiable, full insert sequence	-3.37
1428400_at	2200002K05Rik	RIKEN cDNA 2200002K05 gene	-3.39
1437385_at	Ccbe1	collagen and calcium binding EGF domains 1	-3.40
1437870_at	Slco4c1	solute carrier organic anion transporter family, member 4C1	-3.40
1431362_a_at	Smoc2	SPARC related modular calcium binding 2	-3.41
1449799_s_at	Pkp2	plakophilin 2	-3.42
1442927_at	Ptk2b	PTK2 protein tyrosine kinase 2 beta	-3.43
1456389_at	Zeb2	zinc finger E-box binding homeobox 2	-3.47
1458403_at	Tnik	TRAF2 and NCK interacting kinase	-3.47
1459921_at	Nxph1	neurexophilin 1	-3.51
1435311_s_at	Fbxo7	F-box protein 7	-3.53
1427157_at	Ccdc85a	coiled-coil domain containing 85A	-3.53
1435396_at	Stxbp6	syntaxin binding protein 6 (amisyn)	-3.54
1440545_at	ENSMUSG00000075319	predicted gene, ENSMUSG00000075319	-3.56
1443287_at	Gm1337	gene model 1337, (NCBI)	-3.56
1430648_at	Scn2b	sodium channel, voltage-gated, type II, beta	-3.57
1452263_at	Slc35f4	solute carrier family 35, member F4	-3.57
1448930_at	3010026O09Rik	RIKEN cDNA 3010026O09 gene	-3.59
1452453_a_at	Camk2a	calcium/calmodulin-dependent protein kinase II alpha	-3.59
1436532_at	Dclk3	doublecortin-like kinase 3	-3.60
1442272_at	1700021K10Rik	RIKEN cDNA 1700021K10 gene	-3.60

1425337_at	Slc12a5	solute carrier family 12, member 5	-3.61
1434501_at	Ypel4	yippee-like 4 (Drosophila)	-3.63
1447005_at	---	---	-3.68
1452332_at	Ccdc85a	coiled-coil domain containing 85A	-3.68
1440418_at	---	---	-3.70
1436998_at	Ankrd43	ankyrin repeat domain 43	-3.71
1437018_at	Pnma2	paraneoplastic antigen MA2	-3.76
1438235_at	---	---	-3.77
1434052_at	AI593442	expressed sequence AI593442	-3.79
1436142_at	Akap5	A kinase (PRKA) anchor protein 5	-3.80
1450712_at	Kcnj9	potassium inwardly-rectifying channel, subfamily J, member 9	-3.82
1453245_at	9130024F11Rik	RIKEN cDNA 9130024F11 gene	-3.83
1438020_at	Hapln1	hyaluronan and proteoglycan link protein 1	-3.86
1447222_at	Hspa12a	heat shock protein 12A	-3.92
1459717_at	---	Transcribed locus	-3.95
1434819_at	St6gal2	beta galactoside alpha 2,6 sialyltransferase 2	-4.00
1455753_at	C630035N08Rik	RIKEN cDNA C630035N08 gene	-4.00
1426043_a_at	Capn3	calpain 3	-4.02
1455040_s_at	1110062M06Rik	RIKEN cDNA 1110062M06 gene	-4.02
1457066_at	Abcc8	ATP-binding cassette, sub-family C (CFTR/MRP), member 8	-4.03
1441482_at	---	---	-4.06
1458697_at	---	Transcribed locus	-4.08
1433032_at	2900064K03Rik	RIKEN cDNA 2900064K03 gene	-4.14
1438500_at	Prrt3	proline-rich transmembrane protein 3	-4.20
1433469_at	Lrrn2	leucine rich repeat protein 2, neuronal	-4.23
1418174_at	Dbp	D site albumin promoter binding protein	-4.26
1425870_a_at	Kcnip2	Kv channel-interacting protein 2	-4.26
1435407_at	---	Adult male medulla oblongata cDNA, RIKEN full-length enriched library, clone:6332404K23 product:unclassifiable, full insert sequence	-4.32
1422052_at	Cdh8	cadherin 8	-4.42
1442581_at	Ksr1	kinase suppressor of ras 1	-4.52
1436021_at	Mfsd4	major facilitator superfamily domain containing 4	-4.70
1417489_at	Npy2r	neuropeptide Y receptor Y2	-4.71
1426115_a_at	Kcnj9	potassium inwardly-rectifying channel, subfamily J, member 9	-4.90
1445415_at	EG328191	Predicted gene, EG328191	-5.13
1455531_at	Mfsd4	major facilitator superfamily domain containing 4	-5.15
1417638_at	Lefty1	left right determination factor 1	-5.16
1443876_at	---	16 days neonate thymus cDNA, RIKEN full-length enriched library, clone:A130020E07 product:calcium/calmodulin-dependent protein kinase II alpha, full insert sequence	-5.16
1439872_at	---	KCNA2 (Kcna2) mRNA, 3' UTR	-5.17
1435493_at	Dsp	desmoplakin	-5.30
1438211_s_at	Dbp	D site albumin promoter binding protein	-5.39
1435494_s_at	Dsp	desmoplakin	-5.49
1418047_at	Neurod6	neurogenic differentiation 6	-5.60
1440946_at	---	Transcribed locus	-5.98
1447726_at	Ripply2	ripply2 homolog (zebrafish)	-6.38
1449472_at	Gpr12	G-protein coupled receptor 12	-6.56
1447115_at	C78409	expressed sequence C78409	-6.95

1433793_s_at	Nrip2	nuclear receptor interacting protein 2	-7.10
1439479_at	Lct	lactase	-7.32
1418266_at	Alox12b /// LOC100045755	arachidonate 12-lipoxygenase, 12R type /// similar to arachidonate 12-lipoxygenase	-20.35
