# Supplementary material for: Glial responses during epileptogenesis in Mus musculus point to potential therapeutic targets
Source: PLoS One. 2018 Aug 16;13(8):e0201742. doi: 10.1371/journal.pone.0201742 (PMC6095496; doi:10.1371/journal.pone.0201742)
Supplement: S3 Table — (PDF) [file pone.0201742.s007.pdf]

Table S3: Significant gene expression changes detected by microarrays following SAM analysis at 24 hours post-injection (thresholds: $\geq$ 2 fold and 0% median FDR).

Probe Set ID	Gene Symbol	Gene Title	Fold Change
1459372_at	Npas4	neuronal PAS domain protein 4	27.33
1449254_at	Spp1	secreted phosphoprotein 1	22.48
1449827_at	Acan	aggrecan	19.91
1419149_at	Serpine1	serine (or cysteine) peptidase inhibitor, clade E, member 1	18.71
1436484_at	C030019I05Rik	RIKEN cDNA C030019I05 gene	16.03
1438133_a_at	Cyr61	cysteine rich protein 61	15.96
1425964_x_at	Hspb1	heat shock protein 1	14.48
1420720_at	LOC100044234 /// Nptx2	neuronal pentraxin 2 /// hypothetical protein LOC100044234	14.10
1425952_a_at	Gcg	glucagon	13.22
1416039_x_at	Cyr61	cysteine rich protein 61	13.06
1449982_at	Il11	interleukin 11	12.44
1442340_x_at	Cyr61	cysteine rich protein 61	11.92
1424638_at	Cdkn1a	cyclin-dependent kinase inhibitor 1A (P21)	10.59
1419599_s_at	Ms4a6d	membrane-spanning 4-domains, subfamily A, member 6D	9.44
1422943_a_at	Hspb1	heat shock protein 1	9.32
1420753_at	Tll1	tolloid-like	9.24
1453238_s_at	3930401B19Rik /// A130040M12Rik /// E430024C06Rik /// LOC100039378 /// LOC100039583 /// LOC100041150 /// LOC100041274 /// LOC100041962 /// LOC100043154 /// LOC100043406 /// LOC100048290	RIKEN cDNA 3930401B19 gene /// RIKEN cDNA A130040M12 gene /// RIKEN cDNA E430024C06 gene /// similar to gag protein	9.18
1416431_at	Tubb6	tubulin, beta 6	9.07
1449960_at	Nptx2	neuronal pentraxin 2	8.89
1428776_at	Slc10a6	solute carrier family 10 (sodium/bile acid cotransporter family), member 6	8.70
1440342_at	G530011O06Rik	RIKEN cDNA G530011O06 gene	8.22
1416529_at	Emp1	epithelial membrane protein 1	8.22
1423100_at	Fos	FBJ osteosarcoma oncogene	8.18
1434089_at	Synpo	synaptopodin	8.02
1424528_at	Cgref1	cell growth regulator with EF hand domain 1	7.71
1434376_at	Cd44	CD44 antigen	7.66
1418349_at	Hbegf	heparin-binding EGF-like growth factor	7.63
1424248_at	Arpp21	cyclic AMP-regulated phosphoprotein, 21	7.60
1424880_at	Trib1	tribbles homolog 1 (Drosophila)	7.17
1444982_at	---	---	7.09
1453851_a_at	Gadd45g	growth arrest and DNA-damage-inducible 45 gamma	6.99
1421207_at	Lif	leukemia inhibitory factor	6.95

1437270_a_at	Clcf1	cardiotrophin-like cytokine factor 1	6.88
1451798_at	Il1rn	interleukin 1 receptor antagonist	6.73
1424529_s_at	Cgref1	cell growth regulator with EF hand domain 1	6.73
1448061_at	Msr1	macrophage scavenger receptor 1	6.61
1452318_a_at	Hspa1b	heat shock protein 1B	6.49
1418936_at	Maff	v-maf musculoaponeurotic fibrosarcoma oncogene family, protein F (avian)	6.46
1449388_at	Thbs4	thrombospondin 4	6.38
1437056_x_at	Crispld2	cysteine-rich secretory protein LCCL domain containing 2	6.12
1424090_at	Sdcbp2	syndecan binding protein (syntenin) 2	5.86
1417936_at	Ccl9	chemokine (C-C motif) ligand 9	5.81
1423312_at	Tpbg	trophoblast glycoprotein	5.80
1433599_at	Baz1a	bromodomain adjacent to zinc finger domain 1A	5.75
1455900_x_at	Tgm2	transglutaminase 2, C polypeptide	5.63
1419091_a_at	Anxa2	annexin A2	5.61
1448471_a_at	Ctla2a	cytotoxic T lymphocyte-associated protein 2 alpha	5.57
1427355_at	Calca	calcitonin/calcitonin-related polypeptide, alpha	5.55
1418350_at	Hbegf	heparin-binding EGF-like growth factor	5.43
1424107_at	Kif18a	kinesin family member 18A	5.36
1425351_at	Srxn1	sulfiredoxin 1 homolog (S. cerevisiae)	5.24
1422706_at	Tmepai	transmembrane, prostate androgen induced RNA	5.21
1437247_at	Fosl2 /// LOC634417	fos-like antigen 2 /// similar to fos-like antigen 2	5.17
1418804_at	Sucnr1	succinate receptor 1	5.03
1460302_at	LOC640441 /// Thbs1	thrombospondin 1 /// similar to thrombospondin 1	4.99
1429637_at	2210419I08Rik	RIKEN cDNA 2210419I08 gene	4.85
1460227_at	Timp1	tissue inhibitor of metalloproteinase 1	4.82
1436778_at	Cybb	cytochrome b-245, beta polypeptide	4.75
1449872_at	Hspb3	heat shock protein 3	4.74
1429399_at	Rnf125	ring finger protein 125	4.74
1435137_s_at	1200015M12Rik /// 1200016E24Rik /// A130040M12Rik /// E430024C06Rik	RIKEN cDNA 1200015M12 gene /// RIKEN cDNA 1200016E24 gene /// RIKEN cDNA A130040M12 gene /// RIKEN cDNA E430024C06 gene	4.71
1437132_x_at	Nedd9	neural precursor cell expressed, developmentally down-regulated gene 9	4.70
1437173_at	Edg3	endothelial differentiation, sphingolipid G-protein-coupled receptor, 3	4.69
1437766_at	---	---	4.66
1421814_at	Msn	moesin	4.63
1442082_at	C3ar1	complement component 3a receptor 1	4.59
1417400_at	Rai14	retinoic acid induced 14	4.59
1448748_at	Plek	pleckstrin	4.59
1444307_at	---	Transcribed locus	4.51
1451280_at	Arpp21	cyclic AMP-regulated phosphoprotein, 21	4.51
1416762_at	S100a10	S100 calcium binding protein A10 (calpactin)	4.51
1426875_s_at	Srxn1	sulfiredoxin 1 homolog (S. cerevisiae)	4.43

1438761_a_at	EG666231 /// EG668343 /// LOC546355 /// LOC627245 /// LOC632337 /// LOC677259 /// Odc1	ornithine decarboxylase, structural 1 /// similar to Ornithine decarboxylase (ODC) /// similar to Odc1 protein /// predicted gene, EG666231 /// predicted gene, EG668343	4.42
1452352_at	Ctla2b	cytotoxic T lymphocyte-associated protein 2 beta	4.39
1456642_x_at	S100a10	S100 calcium binding protein A10 (calpactin)	4.36
1433675_at	Snhg1	small nucleolar RNA host gene (non-protein coding) 1	4.34
1424067_at	Icam1	intercellular adhesion molecule	4.32
1416342_at	Tnc	tenascin C	4.32
1450971_at	Gadd45b	growth arrest and DNA-damage-inducible 45 beta	4.27
1429848_at	Pvr	poliovirus receptor	4.25
1417426_at	Srgn	serglycin	4.25
1448303_at	Gpnmb	glycoprotein (transmembrane) nmb	4.20
1456251_x_at	Tspo	translocator protein	4.18
1435693_at	Mall	mal, T-cell differentiation protein-like	4.17
1417394_at	Klf4	Kruppel-like factor 4 (gut)	4.05
1431043_at	Kbtbd5	kelch repeat and BTB (POZ) domain containing 5	4.05
1421855_at	Fgl2	fibrinogen-like protein 2	4.02
1432176_a_at	Eng	endoglin	3.92
1440142_s_at	Gfap	glial fibrillary acidic protein	3.90
1451680_at	Srxn1	sulfiredoxin 1 homolog (S. cerevisiae)	3.78
1439399_a_at	Snhg1	small nucleolar RNA host gene (non-protein coding) 1	3.78
1422452_at	Bag3	Bcl2-associated athanogene 3	3.77
1437711_x_at	EG666231 /// EG668343 /// LOC546355 /// LOC627245 /// LOC632337 /// LOC665017 /// LOC676173 /// Odc1	ornithine decarboxylase, structural 1 /// similar to Ornithine decarboxylase (ODC) /// similar to Odc1 protein /// predicted gene, EG666231 /// predicted gene, EG668343	3.74
1457984_at	Crh	corticotropin releasing hormone	3.71
1417818_at	Wwtr1	WW domain containing transcription regulator 1	3.69
1415943_at	Sdc1	syndecan 1	3.68
1434891_at	Ptgfrn	prostaglandin F2 receptor negative regulator	3.67
1433674_a_at	Snhg1	small nucleolar RNA host gene (non-protein coding) 1	3.67
1451969_s_at	Parp3	poly (ADP-ribose) polymerase family, member 3	3.66
1419127_at	Npy	neuropeptide Y	3.65
1450641_at	Vim	vimentin	3.65
1452717_at	Slc25a24	solute carrier family 25 (mitochondrial carrier, phosphate carrier), member 24	3.61
1435084_at	C730049O14Rik	RIKEN cDNA C730049O14 gene	3.59
1422286_a_at	Tgif1	TG interacting factor 1	3.57
1447825_x_at	Pcdh8	protocadherin 8	3.56

1448870_at	Ltbp1	latent transforming growth factor beta binding protein 1	3.51
1418674_at	Osmr	oncostatin M receptor	3.49
1450708_at	Scg2	secretogranin II	3.40
1448323_a_at	Bgn	biglycan	3.38
1422629_s_at	Shroom3	shroom family member 3	3.37
1427164_at	Il13ra1	interleukin 13 receptor, alpha 1	3.37
1416067_at	Ifrd1	interferon-related developmental regulator 1	3.36
1449221_a_at	Rrbp1	ribosome binding protein 1	3.32
1421172_at	Adam12	a disintegrin and metallopeptidase domain 12 (meltrin alpha)	3.31
1452217_at	Ahnak	AHNAK nucleoprotein (desmoyokin)	3.31
1417051_at	Pcdh8	protocadherin 8	3.30
1428083_at	2310043N10Rik	RIKEN cDNA 2310043N10 gene	3.30
1452784_at	Itgav	integrin alpha V	3.30
1426063_a_at	Gem	GTP binding protein (gene overexpressed in skeletal muscle)	3.23
1454783_at	Il13ra1	interleukin 13 receptor, alpha 1	3.22
1448894_at	Akr1b8	aldo-keto reductase family 1, member B8	3.22
1436449_at	---	15 days embryo head cDNA, RIKEN full-length enriched library, clone:D930025C07 product:unclassifiable, full insert sequence	3.21
1457474_at	---	Transcribed locus	3.20
1435926_at	Chml	choroideremia-like	3.20
1423062_at	Igfbp3	insulin-like growth factor binding protein 3	3.18
1423904_a_at	Pvr	poliovirus receptor	3.18
1433555_at	Eaf1	ELL associated factor 1	3.17
1426246_at	Pros1	protein S (alpha)	3.17
1451171_at	2310008H04Rik	RIKEN cDNA 2310008H04 gene	3.17
1449036_at	Rnf128	ring finger protein 128	3.17
1452158_at	Eprs /// LOC633677	glutamyl-prolyl-tRNA synthetase /// similar to Bifunctional aminoacyl-tRNA synthetase	3.15
1437495_at	LOC100047187 /// Yy2	similar to zinc finger, X-linked, duplicated B /// Yy2 transcription factor	3.15
1420973_at	Arid5b /// LOC100044968	AT rich interactive domain 5B (Mrf1 like) /// similar to modulator recognition factor 2	3.14
1428783_at	Prkar2a	protein kinase, cAMP dependent regulatory, type II alpha	3.10
1439348_at	S100a10	S100 calcium binding protein A10 (calpactin)	3.08
1418135_at	Aff1	AF4/FMR2 family, member 1	3.07
1415844_at	Syt4	synaptotagmin IV	3.06
1436659_at	Dclk1	doublecortin-like kinase 1	3.04
1418666_at	Ptx3	pentraxin related gene	3.02
1455247_at	Amotl1	angiomotin-like 1	3.02
1416226_at	Arpc1b	actin related protein 2/3 complex, subunit 1B	3.01
1418071_s_at	Cdyl	chromodomain protein, Y chromosome-like	3.00
1417562_at	Eif4ebp1	eukaryotic translation initiation factor 4E binding protein 1	2.99
1434585_at	Tulp4	tubby like protein 4	2.97
1436866_at	Efna5	ephrin A5	2.96
1418492_at	Grem2	gremlin 2 homolog, cysteine knot superfamily (Xenopus laevis)	2.94
1449141_at	Fblim1	filamin binding LIM protein 1	2.93
1456381_x_at	Mcl1	myeloid cell leukemia sequence 1	2.91
1440866_at	Eif2ak2	eukaryotic translation initiation factor 2-alpha kinase 2	2.90

1441823_at	Zmiz1	zinc finger, MIZ-type containing 1	2.88
1423465_at	Frrs1 /// LOC100046401	ferric-chelate reductase 1 /// similar to SDR2	2.85
1435701_at	---	13 days embryo lung cDNA, RIKEN full-length enriched library, clone:D430017B04 product:unclassifiable, full insert sequence	2.84
1451201_s_at	Rnh1	ribonuclease/angiogenin inhibitor 1	2.84
1438725_at	Med13	mediator complex subunit 13	2.84
1448503_at	LOC632101 /// Mcl1	myeloid cell leukemia sequence 1 /// similar to myeloid cell leukemia sequence 1	2.80
1441081_a_at	1110038B12Rik	RIKEN cDNA 1110038B12 gene	2.80
1447868_x_at	Glrx3 /// LOC620016	glutaredoxin 3 /// similar to thioredoxin-like 2	2.79
1456397_at	Cdh4	cadherin 4	2.79
1421530_a_at	Grm8	glutamate receptor, metabotropic 8	2.78
1424065_at	Edem1	ER degradation enhancer, mannosidase alpha-like 1	2.78
1428942_at	Mt2	metallothionein 2	2.78
1422573_at	Ampd3	AMP deaminase 3	2.78
1416700_at	Rnd3	Rho family GTPase 3	2.77
1451065_a_at	Ddx39	DEAD (Asp-Glu-Ala-Asp) box polypeptide 39	2.75
1423903_at	Pvr	poliovirus receptor	2.75
1459170_at	---	---	2.75
1425719_a_at	Nmi	N-myc (and STAT) interactor	2.75
1419573_a_at	Lgals1	lectin, galactose binding, soluble 1	2.73
1449360_at	Csf2rb2	colony stimulating factor 2 receptor, beta 2, low-affinity (granulocyte-macrophage)	2.73
1422045_a_at	Ptpn12	protein tyrosine phosphatase, non-receptor type 12	2.72
1416303_at	Litaf	LPS-induced TN factor	2.72
1421392_a_at	Birc3	baculoviral IAP repeat-containing 3	2.68
1439434_x_at	Sh2d5	SH2 domain containing 5	2.68
1419248_at	Rgs2	regulator of G-protein signaling 2	2.68
1418825_at	Irgm	immunity-related GTPase family, M	2.68
1452157_at	Eprs /// LOC633677	glutamyl-prolyl-tRNA synthetase /// similar to Bifunctional aminoacyl-tRNA synthetase	2.67
1438168_x_at	Ddx39	DEAD (Asp-Glu-Ala-Asp) box polypeptide 39	2.66
1433696_at	Hn1l	hematological and neurological expressed 1-like	2.65
1419247_at	Rgs2	regulator of G-protein signaling 2	2.65
1448558_a_at	Pla2g4a	phospholipase A2, group IVA (cytosolic, calcium-dependent)	2.65
1424229_at	Dyrk3	dual-specificity tyrosine-(Y)-phosphorylation regulated kinase 3	2.64
1418397_at	Zfp275	zinc finger protein 275	2.63
1423254_x_at	Rps27l	ribosomal protein S27-like	2.63
1426912_at	EG621205 /// Rfwd2	ring finger and WD repeat domain 2 /// predicted gene, EG621205	2.63
1436202_at	---	---	2.63
1442308_at	Smyd4	SET and MYND domain containing 4	2.63
1455814_x_at	Ddx39	DEAD (Asp-Glu-Ala-Asp) box polypeptide 39	2.63
1417351_a_at	LOC100047155 /// Snrpa1	small nuclear ribonucleoprotein polypeptide A' /// similar to Small nuclear ribonucleoprotein polypeptide A	2.63
1460694_s_at	Svil	supervillin	2.62

1458802_at	Hivep3	human immunodeficiency virus type I enhancer binding protein 3	2.61
1438606_a_at	Clic4	chloride intracellular channel 4 (mitochondrial)	2.60
1460603_at	Samd9l	sterile alpha motif domain containing 9-like	2.59
1450767_at	Nedd9	neural precursor cell expressed, developmentally down-regulated gene 9	2.56
1435176_a_at	Id2	inhibitor of DNA binding 2	2.56
1447585_s_at	Mrps6	mitochondrial ribosomal protein S6	2.53
1422818_at	Nedd9	neural precursor cell expressed, developmentally down-regulated gene 9	2.49
1451264_at	Frmd6	FERM domain containing 6	2.49
1417719_at	Sap30	sin3 associated polypeptide	2.49
1423393_at	Clic4	chloride intracellular channel 4 (mitochondrial)	2.49
1436100_at	Sh2d5	SH2 domain containing 5	2.49
1418637_at	Etv3 /// LOC100045950	ets variant gene 3 /// similar to ets variant gene 3	2.48
1452283_at	Rassf8	Ras association (RalGDS/AF-6) domain family 8	2.48
1416881_at	LOC632101 /// Mcl1	myeloid cell leukemia sequence 1 /// similar to myeloid cell leukemia sequence 1	2.47
1451941_a_at	Fcgr2b	Fc receptor, IgG, low affinity IIb	2.47
1455396_at	Atp8b1	ATPase, class I, type 8B, member 1	2.46
1426708_at	Antxr2	anthrax toxin receptor 2	2.43
1443036_at	Zfp804a	zinc finger protein 804A	2.43
1447703_x_at	Zfp593	zinc finger protein 593	2.42
1437785_at	Adamts9	a disintegrin-like and metallopeptidase (reprolysin type) with thrombospondin type 1 motif, 9	2.42
1422506_a_at	Cstb	cystatin B	2.41
1454992_at	Slc7a1	solute carrier family 7 (cationic amino acid transporter, y+ system), member 1	2.40
1424552_at	Casp8	caspase 8	2.37
1448797_at	Elk3	ELK3, member of ETS oncogene family	2.37
1437318_at	Pak3	p21 (CDKN1A)-activated kinase 3	2.37
1452192_at	BC053440	cDNA sequence BC053440	2.36
1437236_a_at	Zfp110	zinc finger protein 110	2.35
1451208_at	Etf1	eukaryotic translation termination factor 1	2.35
1435429_x_at	Rps27l	ribosomal protein S27-like	2.35
1447804_x_at	BC031781	CDNA sequence BC031781	2.33
1423643_at	Ddx39	DEAD (Asp-Glu-Ala-Asp) box polypeptide 39	2.31
1437111_at	Zc3h12c	zinc finger CCCH type containing 12C	2.30
1416066_at	Cd9	CD9 antigen	2.30
1423948_at	Bag2	Bcl2-associated athanogene 2	2.29
1418822_a_at	Arf6	ADP-ribosylation factor 6	2.28
1424769_s_at	Cald1	caldesmon 1	2.28
1437237_x_at	Zfp110	zinc finger protein 110	2.28
1452759_s_at	Ppfibp1	PTPRF interacting protein, binding protein 1 (liprin beta 1)	2.27
1449049_at	Tlr1	toll-like receptor 1	2.27
1423431_a_at	Mybbp1a	MYB binding protein (P160) 1a	2.25
1449674_s_at	Pdcd6ip	programmed cell death 6 interacting protein	2.24
1450925_a_at	Rps27l	ribosomal protein S27-like	2.24
1439740_s_at	Uck2	uridine-cytidine kinase 2	2.23
1437807_x_at	Ctnna1	catenin (cadherin associated protein), alpha 1	2.23
1428011_a_at	Erbb2ip	Erbb2 interacting protein	2.22
1430357_at	H3f3b	H3 histone, family 3B	2.22

1424594_at	Lgals7	lectin, galactose binding, soluble 7	2.22
1455439_a_at	Lgals1	lectin, galactose binding, soluble 1	2.21
1424440_at	Mrps6	mitochondrial ribosomal protein S6	2.21
1450744_at	Ell2	elongation factor RNA polymerase II 2	2.20
1433768_at	Palld	palladin, cytoskeletal associated protein	2.20
1436444_at	6030405A18Rik	RIKEN cDNA 6030405A18 gene	2.20
1460735_at	Svil	supervillin	2.19
1433944_at	Hectd2	HECT domain containing 2	2.19
1435402_at	Gramd1b	GRAM domain containing 1B	2.18
1424290_at	Osgin2	oxidative stress induced growth inhibitor family member 2	2.18
1423596_at	Nek6	NIMA (never in mitosis gene a)-related expressed kinase 6	2.17
1436996_x_at	Lyz	lysozyme	2.17
1447669_s_at	Gng4	guanine nucleotide binding protein (G protein), gamma 4 subunit	2.16
1416382_at	Ctsc	cathepsin C	2.16
1450478_a_at	Ptpn12	protein tyrosine phosphatase, non-receptor type 12	2.16
1448306_at	Nfkbia	nuclear factor of kappa light chain gene enhancer in B-cells inhibitor, alpha	2.16
1435551_at	Fhod3	formin homology 2 domain containing 3	2.15
1428094_at	Lamp2	lysosomal-associated membrane protein 2	2.15
1434510_at	Papss2	3'-phosphoadenosine 5'-phosphosulfate synthase 2	2.15
1460556_at	---	---	2.15
1435091_at	Zfp568	zinc finger protein 568	2.15
1453782_at	3021401C12Rik	RIKEN cDNA 3021401C12 gene	2.13
1449090_a_at	Yes1	Yamaguchi sarcoma viral (v-yes) oncogene homolog 1	2.13
1417165_at	Mbd2	methyl-CpG binding domain protein 2	2.13
1428315_at	Ebna1bp2	EBNA1 binding protein 2	2.11
1448149_at	Ctnna1	catenin (cadherin associated protein), alpha 1	2.11
1423566_a_at	Hsp110	heat shock protein 110	2.10
1431394_a_at	Lrrk2	leucine-rich repeat kinase 2	2.10
1455039_a_at	Sin3b	transcriptional regulator, SIN3B (yeast)	2.10
1434424_at	Flvcr1	feline leukemia virus subgroup C cellular receptor 1	2.09
1423796_at	LOC100045887	similar to PTB-associated splicing factor	2.09
1422808_s_at	Dock2	dedicator of cyto-kinesis 2	2.08
1455897_x_at	Hmgn1	high mobility group nucleosomal binding domain 1	2.08
1436684_a_at	Riok2	RIO kinase 2 (yeast)	2.08
1438579_at	Utp14b	UTP14, U3 small nucleolar ribonucleoprotein, homolog B (yeast)	2.07
1427072_at	Stard8	START domain containing 8	2.07
1419272_at	Myd88	myeloid differentiation primary response gene 88	2.07
1416257_at	Capn2	calpain 2	2.06
1436506_a_at	Snhg6	small nucleolar RNA host gene (non-protein coding) 6	2.06
1437726_x_at	C1qb	complement component 1, q subcomponent, beta polypeptide	2.06
1450899_at	Nedd1	neural precursor cell expressed, developmentally down-regulated gene 1	2.06

1438750_at	Atrx	Alpha thalassemia/mental retardation syndrome X-linked homolog (human)	2.06
1447757_x_at	Inpp5f	inositol polyphosphate-5-phosphatase F	2.05
1460632_at	---	Transcribed locus	2.05
1448802_at	Nufip1	nuclear fragile X mental retardation protein interacting protein 1	2.05
1416312_at	Rars	arginyl-tRNA synthetase	2.05
1424244_at	Rwdd4a	RWD domain containing 4A	2.04
1442793_s_at	LOC100046166 /// Tbrg4	transforming growth factor beta regulated gene 4 /// similar to Transforming growth factor beta regulated gene 4	2.04
1417116_at	Slc6a8	solute carrier family 6 (neurotransmitter transporter, creatine), member 8	2.04
1435655_at	Snora65	small nucleolar RNA, H/ACA box 65	2.04
1428919_at	Fgfr1op	Fgfr1 oncogene partner	2.04
1446835_at	---	---	2.03
1419749_at	Trdmt1	tRNA aspartic acid methyltransferase 1	2.02
1456093_at	Zfp536	zinc finger protein 536	2.02
1448968_at	Ubfd1	ubiquitin family domain containing 1	2.02
1428078_at	0610013E23Rik	RIKEN cDNA 0610013E23 gene	2.00
1441716_at	---	12 days embryo eyeball cDNA, RIKEN full-length enriched library, clone:D230024G22 product:unclassifiable, full insert sequence	-2.00
1456900_at	---	---	-2.01
1455883_a_at	Lrrtm1	leucine rich repeat transmembrane neuronal 1	-2.02
1428156_at	Gng2	guanine nucleotide binding protein (G protein), gamma 2 subunit	-2.04
1426729_at	2900046G09Rik	RIKEN cDNA 2900046G09 gene	-2.07
1420925_at	Tub	tubby candidate gene	-2.08
1447992_s_at	Pcsk2	proprotein convertase subtilisin/kexin type 2	-2.08
1425987_a_at	Kcnma1	potassium large conductance calcium-activated channel, subfamily M, alpha member 1	-2.09
1449376_at	Nicn1	nicolin 1	-2.10
1426616_at	Tlcd1	TLC domain containing 1	-2.10
1429021_at	Epha4	Eph receptor A4	-2.11
1420871_at	Gucy1b3	guanylate cyclase 1, soluble, beta 3	-2.11
1455082_at	Cblb	Casitas B-lineage lymphoma b	-2.12
1448411_at	Wfs1	Wolfram syndrome 1 homolog (human)	-2.13
1445837_at	---	12 days embryo spinal ganglion cDNA, RIKEN full-length enriched library, clone:D130073P12 product:unclassifiable, full insert sequence	-2.14
1458697_at	---	Transcribed locus	-2.18
1450770_at	3632451O06Rik	RIKEN cDNA 3632451O06 gene	-2.18
1435668_at	4932442K08Rik	RIKEN cDNA 4932442K08 gene	-2.19
1449620_s_at	D16Wsu65e	DNA segment, Chr 16, Wayne State University 65, expressed	-2.20
1458421_at	Kcnq3	potassium voltage-gated channel, subfamily Q, member 3	-2.21
1453836_a_at	Mgll	monoglyceride lipase	-2.21
1420537_at	Kctd4	potassium channel tetramerisation domain containing 4	-2.24
1436493_at	BB181834	expressed sequence BB181834	-2.25
1457587_at	Kcnq5	potassium voltage-gated channel, subfamily Q, member 5	-2.31
1440343_at	Rps6ka5	ribosomal protein S6 kinase, polypeptide 5	-2.33

1432027_a_at	LOC100044325 /// LOC100045928 /// Tbc1d14	TBC1 domain family, member 14 /// similar to mKIAA1322 protein /// similar to TBC1 domain family, member 14	-2.35
1430237_at	Cldn22	claudin 22	-2.35
1454721_at	1110018G07Rik	RIKEN cDNA 1110018G07 gene	-2.36
1436532_at	Dclk3	doublecortin-like kinase 3	-2.38
1459971_at	---	Transcribed locus	-2.39
1431569_a_at	Lypd1	Ly6/Plaur domain containing 1	-2.41
1439870_at	A330008L17Rik	RIKEN cDNA A330008L17 gene	-2.41
1427281_at	Scn2a1	sodium channel, voltage-gated, type II, alpha 1	-2.42
1420191_s_at	Tmem191c	transmembrane protein 191C	-2.46
1416702_at	Serpini1	serine (or cysteine) peptidase inhibitor, clade I, member 1	-2.48
1460286_at	6-Sep	septin 6	-2.49
1433681_x_at	Capn3	calpain 3	-2.51
1428813_a_at	Drd1ip	dopamine receptor D1 interacting protein	-2.51
1435033_at	Arhgef4	Rho guanine nucleotide exchange factor (GEF) 4	-2.53
1422052_at	Cdh8	cadherin 8	-2.54
1450930_at	Hpca	hippocalcin	-2.55
1417664_a_at	Ndrg3	N-myc downstream regulated gene 3	-2.55
1435407_at	---	Adult male medulla oblongata cDNA, RIKEN full-length enriched library, clone:6332404K23 product:unclassifiable, full insert sequence	-2.58
1434581_at	---	---	-2.60
1434454_at	D16Wsu65e	DNA segment, Chr 16, Wayne State University 65, expressed	-2.69
1444723_at	6530418L21Rik	RIKEN cDNA 6530418L21 gene	-2.69
1451499_at	Cadps2	Ca2+-dependent activator protein for secretion 2	-2.75
1449429_at	Fkbp1b	FK506 binding protein 1b	-2.77
1452332_at	Ccdc85a	coiled-coil domain containing 85A	-2.77
1439904_at	Fstl5	follistatin-like 5	-2.81
1456765_at	6430511F03	hypothetical protein 6430511F03	-2.81
1449468_at	St6galnac5	ST6 (alpha-N-acetyl-neuraminyl-2,3-beta-galactosyl-1,3)-N-acetylgalactosaminide alpha-2,6-sialyltransferase 5	-2.83
1446265_at	Dnm3	dynamin 3	-2.86
1451583_a_at	BC025076	cDNA sequence BC025076	-2.88
1420876_a_at	6-Sep	septin 6	-2.90
1438540_at	Col25a1	collagen, type XXV, alpha 1	-2.99
1419332_at	Egfl6	EGF-like-domain, multiple 6	-3.00
1451894_a_at	Scn8a	sodium channel, voltage-gated, type VIII, alpha	-3.01
1417312_at	Dkk3	dickkopf homolog 3 (Xenopus laevis)	-3.02
1425834_a_at	Gpam	glycerol-3-phosphate acyltransferase, mitochondrial	-3.03
1440545_at	ENSMUSG00000075319	predicted gene, ENSMUSG00000075319	-3.04
1454043_a_at	Kcnab1	potassium voltage-gated channel, shaker-related subfamily, beta member 1	-3.04
1445691_at	Chn1	chimerin (chimaerin) 1	-3.10
1436142_at	Akap5	A kinase (PRKA) anchor protein 5	-3.13
1455145_at	Pcdh19	protocadherin 19	-3.19
1433469_at	Lrrn2	leucine rich repeat protein 2, neuronal	-3.29
1438784_at	Bcl11b	B-cell leukemia/lymphoma 11B	-3.30

1418683_at	Lin7b	lin-7 homolog B (C. elegans)	-3.35
1438710_at	Htr1a	5-hydroxytryptamine (serotonin) receptor 1A	-3.41
1429443_at	Cpne4	copine IV	-3.46
1425574_at	Epha3	Eph receptor A3	-3.48
1449172_a_at	Lin7b	lin-7 homolog B (C. elegans)	-3.49
1454653_at	Cpne9	copine family member IX	-3.51
1440534_at	ENSMUSG00000 056615	predicted gene, ENSMUSG00000056615	-3.60
1441801_at	Kctd4	potassium channel tetramerisation domain containing 4	-3.62
1418047_at	Neurod6	neurogenic differentiation 6	-3.82
1418082_at	LOC100045684	similar to N-myristoyltransferase 1	-4.20
1425833_a_at	Hpca	hippocalcin	-4.45
1432385_a_at	Agtpbp1	ATP/GTP binding protein 1	-4.57
1419250_a_at	Pftk1	PFTAIRE protein kinase 1	-4.96
1420563_at	Gria3	glutamate receptor, ionotropic, AMPA3 (alpha 3)	-5.00
1431717_at	Akap5	A kinase (PRKA) anchor protein 5	-5.38
1426328_a_at	Scn3b	sodium channel, voltage-gated, type III, beta	-5.71
1450143_at	Rasgrp1	RAS guanyl releasing protein 1	-6.28
1451800_at	Gcc2	GRIP and coiled-coil domain containing 2	-6.83
1442272_at	1700021K10Rik	RIKEN cDNA 1700021K10 gene	-7.60
1458363_at	Zdhhc17	zinc finger, DHHC domain containing 17	-7.70
1445858_at	4933439G19Rik	RIKEN cDNA 4933439G19 gene	-8.71
