# Supplementary material for: Glial responses during epileptogenesis in Mus musculus point to potential therapeutic targets
Source: PLoS One. 2018 Aug 16;13(8):e0201742. doi: 10.1371/journal.pone.0201742 (PMC6095496; doi:10.1371/journal.pone.0201742)
Supplement: S4 Table — (n/c: not changed) (PDF) [file pone.0201742.s008.pdf]

Table S4: qRT-PCR validation of selected microarray statistically significant gene expression changes in KA-versus saline-injected hippocampi, with reference to Gapdh. (n/c: not changed)

Gene Symbol	6 hours			12 hours		
	Microarrays	qRT-PCR		Microarrays	qRT-PCR	
	Fold Change	Fold Change	Standard Deviation	Fold Change	Fold Change	Standard Deviation
Bdnf	6.1	16.58	0.37	4.02	4.70	0.12
Casp8	n/c	n/c	-	n/c	2.67	0.05
Fos	12.2	40.52	3.55	17.00	59.64	2.67
Hspa1b	n/c	87.28	5.41	2.72	11.66	0.69
Nos3	n/c	3.85	0.04	15.56	5.40	0.28
Tnfrs1a	n/c	4.10	0.08	2.35	n/c	-
