# Supplementary material for: Glial responses during epileptogenesis in Mus musculus point to potential therapeutic targets
Source: PLoS One. 2018 Aug 16;13(8):e0201742. doi: 10.1371/journal.pone.0201742 (PMC6095496; doi:10.1371/journal.pone.0201742)
Supplement: S5 Table — All significantly changed genes at 6h were considered, and a threshold of p-value <0.05 was applied. (PDF) [file pone.0201742.s009.pdf]

Table S5: Significantly changed GO Biological Processes (level 6) at 6 hours post KA treatment, using the "Mapping to ontologies (TRANSPATH®)" workflow. All significantly changed genes at 6h were considered, and a threshold of p-value <0.05 was applied.

Gene Ontology Category ID	GO-Biologic Process (level 6)	Time point(s) of enrichment	Number of significantly changed genes at 6h	Symbol of significantly changed genes at 6h
GO:0031326	regulation of cellular biosynthetic process	6h, 12h, 24h	66	4933421E11Rik, Arhgef2, Arpp19, Atf7ip (ENSMUSG00000053935), Avpr1a, Bach2, Baz1a, Bcl11b, Bcl7a, Casc3, Cdkn1a, Cited2, Crem, Csrnp1, Cyr61, Dhx33, Dusp5, E2f3, Egr4, Ell2, Elp2, Fos, Fosb, Foxk1, Foxp1, Hbegf, Hcfc2, Htr1a, Jun, Junb, Kdm4b, Kdm6b, Kitl, Mcf2l, Mterfd3, Neurod2, Neurod6, Nfkbiz, Npas4, Nr3c2, Nr4a1, Nr4a2, Nr4a3, Nrip2, P2ry12, Pdk4, Per1, Plagl1, Ptgs2, Rcan1, Rcor2, Rgs2, Rorb, Sap18, Sap30, Setd1b, Smad7, Tfdp2, Tinf2, Wnt2, Zeb2, Zfp12, Zfp128, Zfp292, Zfp36, Zfp462
GO:0019219	regulation of nucleobase-containing compound metabolic process	6h, 12h, 24h	64	4933421E11Rik, Arhgef2, Atf7ip (ENSMUSG00000053935), Bach2, Bag4, Baz1a, Bcl11b, Bcl7a, Cdkn1a, Cited2, Crem, Csrnp1, Cyr61, Dhx33, Dusp5, E2f3, Egr4, Ell2, Elp2, Errfi1, Fos, Fosb, Foxk1, Foxp1, Hcfc2, Htr1a, Jun, Junb, Kdm4b, Kdm6b, Kitl, Mcf2l, Mterfd3, Neurod2, Neurod6, Nfkbiz, Npas4, Nr3c2, Nr4a1, Nr4a2, Nr4a3, Nrip2, P2ry12, Per1, Plagl1, Ptbp2, Rcan1, Rcor2, Rgs2, Rorb, Sap18, Sap30, Setd1b, Smad7, Srsf12, Tfdp2, Tinf2, Wnt2, Zeb2, Zfp12, Zfp128, Zfp292, Zfp36, Zfp462

GO:0010556	regulation of macromolecule biosynthetic process	6h, 12h, 24h	60	4933421E11Rik, Arhgef2, Atf7ip (ENSMUSG00000053935), Bach2, Baz1a, Bcl11b, Bcl7a, Casc3, Cdkn1a, Cited2, Crem, Csrnp1, Cyr61, Dhx33, Dusp5, E2f3, Egr4, Ell2, Elp2, Fos, Fosb, Foxk1, Foxp1, Hbegf, Hcfc2, Jun, Junb, Kdm4b, Kdm6b, Kitl, Mcf2l, Mterfd3, Neurod2, Neurod6, Nfkbiz, Npas4, Nr3c2, Nr4a1, Nr4a2, Nr4a3, Nrip2, Per1, Plagl1, Rcan1, Rcor2, Rgs2, Rorb, Sap18, Sap30, Setd1b, Smad7, Tfdp2, Tinf2, Wnt2, Zeb2, Zfp12, Zfp128, Zfp292, Zfp36, Zfp462
GO:0010468	regulation of gene expression	6h, 12h, 24h	59	4933421E11Rik, Arhgef2, Atf7ip (ENSMUSG00000053935), Bach2, Baz1a, Bcl11b, Bcl7a, Casc3, Cdkn1a, Cited2, Crem, Csrnp1, Cyr61, Dhx33, Dusp5, E2f3, Egr4, Ell2, Elp2, Fos, Fosb, Foxk1, Foxp1, Hcfc2, Jun, Junb, Kdm4b, Kdm6b, Mcf2l, Mterfd3, Neurod2, Neurod6, Nfkbiz, Npas4, Nr3c2, Nr4a1, Nr4a2, Nr4a3, Nrip2, Per1, Plagl1, Ptbp2, Rcan1, Rcor2, Rgs2, Rorb, Sap18, Sap30, Setd1b, Smad7, Srsf12, Tfdp2, Wnt2, Zeb2, Zfp12, Zfp128, Zfp292, Zfp36, Zfp462
GO:0051252	regulation of RNA metabolic process	6h, 12h, 24h	57	4933421E11Rik, Arhgef2, Atf7ip (ENSMUSG00000053935), Bach2, Bag4, Baz1a, Bcl11b, Bcl7a, Cited2, Crem, Csrnp1, Cyr61, Dhx33, Dusp5, E2f3, Egr4, Ell2, Elp2, Fos, Fosb, Foxk1, Foxp1, Hcfc2, Jun, Junb, Kdm4b, Kdm6b, Mcf2l, Mterfd3, Neurod2, Neurod6, Nfkbiz, Npas4, Nr3c2, Nr4a1, Nr4a2, Nr4a3, Nrip2, Per1, Plagl1, Ptbp2, Rcan1, Rcor2, Rorb, Sap18, Sap30, Setd1b, Smad7, Srsf12, Tfdp2, Wnt2, Zeb2, Zfp12, Zfp128, Zfp292, Zfp36, Zfp462

GO:0006351	transcription, DNA-dependent	6h, 12h	38	4933421E11Rik, Atf7ip (ENSMUSG00000053935), Bach2, Baz1a, Bcl11b, Crem, Csrnp1, E2f3, Egr4, Ell2, Elp2, Fos, Fosb, Foxk1, Foxp1, Jun, Junb, Kdm4b, Mterfd3, Neurod2, Neurod6, Nfkbiz, Npas4, Nr3c2, Nr4a1, Nr4a2, Nr4a3, Nrip2, Per1, Rcor2, Rorb, Sap30, Setd1b, Smad7, Tfdp2, Zeb2, Zfp12, Zfp292
GO:0031325	positive regulation of cellular metabolic process	6h, 12h, 24h	33	Arpp19, Atf7ip (ENSMUSG00000053935), Avpr1a, Bag4, Bcl11b, Cdkn1a, Cited2, Csrnp1, Cyr61, Dhx33, E2f3, Egr4, Ell2, Fos, Foxk1, Foxp1, Jun, Junb, Kitl, Mcf2l, Npas4, Nr4a1, Nr4a2, Nr4a3, Plagl1, Ptgs2, Rorb, Smad7, Tinf2, Wnt2, Zfp292, Zfp36, Zfp462
GO:0010604	positive regulation of macromolecule metabolic	6h, 12h, 24h	31	Atf7ip (ENSMUSG00000053935), Avpr1a, Bag4, Bcl11b, Cdkn1a, Cited2, Csrnp1, Cyr61, Dhx33, E2f3, Egr4, Ell2, Fos, Foxk1,
GO:0031324	negative regulation of cellular metabolic process	6h, 12h, 24h	30	Atf7ip (ENSMUSG00000053935), Bag4, Bcl7a, Cdkn1a, Cited2, Dusp5, Errfi1, Fosb, Foxk1, Foxp1, Hbegf, Hcfc2, Htr1a, Jun, Kdm6b, Nrip2, P2ry12, Per1, Ptbp2, Ptpn3, Rcor2, Sap30, Sh3bp5, Smad7, Srsf12, Tinf2, Wnt2, Zfp12, Zfp128, Zfp36
GO:0009891	positive regulation of biosynthetic process	6h, 12h, 24h	29	Arpp19, Atf7ip (ENSMUSG00000053935), Avpr1a, Bcl11b, Cited2, Csrnp1, Cyr61, Dhx33, E2f3, Egr4, Ell2, Fos, Foxk1, Foxp1, Jun, Junb, Kitl, Mcf2l, Npas4, Nr4a1, Nr4a2, Nr4a3, Plagl1, Ptgs2, Rorb, Wnt2, Zfp292, Zfp36, Zfp462
GO:0051173	positive regulation of nitrogen compound metabolic process	6h, 12h, 24h	28	Atf7ip (ENSMUSG00000053935), Bcl11b, Cited2, Csrnp1, Cyr61, Dhx33, E2f3, Egr4, Ell2, Fos, Foxk1, Foxp1, Jun, Junb, Kitl, Mcf2l, Npas4, Nr4a1, Nr4a2, Nr4a3, Plagl1, Ptgs2, Rorb, Tinf2, Wnt2, Zfp292, Zfp36, Zfp462

GO:0010605	negative regulation of macromolecule metabolic process	6h, 12h, 24h	27	Atf7ip (ENSMUSG00000053935), Bag4, Bcl7a, Cdkn1a, Cited2, Dusp5, Errfi1, Fosb, Foxk1, Foxp1, Hbegf, Hcfc2, Jun, Kdm6b, Nrip2, Per1, Ptbp2, Ptpn3, Rcor2, Sap30, Smad7, Srsf12, Tinf2, Wnt2, Zfp12, Zfp128, Zfp36
GO:0043067	regulation of programmed cell death	6h, 12h, 24h	26	Arhgef2, Bag3, Bag4, Bcl11b, Bdnf, Cdkn1a, Cited2, Cyr61, Dusp1, Jun, Kitl, Map3k5, Mcf2l, Nr4a1, Nr4a2, Nr4a3, Plagl1, Plekhg5, Plk2, Ptgs2, Rilpl1, Scg2, Siah2, Tiam1, Tnfrsf22, Tnfrsf22 (ENSMUSG00000010751)
GO:0051172	negative regulation of nitrogen compound metabolic process	6h, 12h, 24h	25	Atf7ip (ENSMUSG00000053935), Bag4, Bcl7a, Cited2, Dusp5, Fosb, Foxk1, Foxp1, Hcfc2, Htr1a, Jun, Kdm6b, Nrip2, P2ry12, Per1, Ptbp2, Rcor2, Sap30, Smad7, Srsf12, Tinf2, Wnt2, Zfp12, Zfp128, Zfp36
GO:0009890	negative regulation of biosynthetic process	6h, 12h, 24h	23	Atf7ip (ENSMUSG00000053935), Bcl7a, Cited2, Dusp5, Fosb, Foxk1, Foxp1, Hbegf, Hcfc2, Htr1a, Jun, Kdm6b, Nrip2, P2ry12, Per1, Rcor2, Sap30, Smad7, Tinf2, Wnt2, Zfp12, Zfp128, Zfp36
GO:0051246	regulation of protein metabolic process	6h, 12h, 24h	23	Bag4, Camkk1, Casc3, Cdkn1a, Cyr61, Dlgap1, Dusp1, Dusp5, Errfi1, Gadd45b, Gadd45g, Jun, Kitl, Map3k5, Nedd4l, Ptpn3, Rgs2, Sh3bp5, Siah2, Smad7, Ssr1, Tinf2, Zeb2
GO:0032268	regulation of cellular protein metabolic process	6h, 12h, 24h	22	Bag4, Camkk1, Casc3, Cdkn1a, Cyr61, Dlgap1, Dusp1, Dusp5, Errfi1, Gadd45b, Gadd45g, Jun, Kitl, Map3k5, Ptpn3, Rgs2, Sh3bp5, Siah2, Smad7, Ssr1, Tinf2, Zeb2
GO:0051174	regulation of phosphorus metabolic process	6h, 12h, 24h	18	Bag4, Camkk1, Cdkn1a, Cyr61, Dusp1, Dusp5, Errfi1, Gadd45b, Gadd45g, Jun, Kitl, Map3k5, Rcan1, Rgs2, Sh3bp5, Smad7, Ssr1, Zeb2
GO:0008284	positive regulation of cell proliferation	6h, 12h, 24h	17	Avpr1a, Cdkn1a, Cyr61, E2f3, Egr4, Foxp1, Hbegf, Htr1a, Jun, Kitl, Npy5r, Nr4a1, Odc1, Ptgs2, Scg2, Ssr1, Wnt2

GO:0007167	enzyme linked receptor protein signaling pathway	6h, 12h, 24h	15	5830428H23Rik, Arhgef2, Cited2, Csrnp1, Fos, Hbegf, Jun, Mcf2l, Nr4a1, Plekhg5, Rtn4r, Smad7, Tiam1, Tiparp, Zfp128
GO:0043549	regulation of kinase activity	6h, 12h, 24h	14	Camkk1, Cdkn1a, Cyr61, Dusp1, Dusp5, Errfi1, Gadd45b, Gadd45g, Kitl, Map3k5, Rgs2, Sh3bp5, Ssr1, Zeb2
GO:0060548	negative regulation of cell death	6h, 12h, 24h	14	Bag3, Bag4, Bcl11b, Bdnf, Cdkn1a, Cited2, Cyr61, Jun, Kitl, Nr4a2, Nr4a3, Plk2, Scg2, Siah2
GO:0010942	positive regulation of cell death	6h, 12h, 24h	13	Arhgef2, Cdkn1a, Cyr61, Dusp1, Jun, Map3k5, Mcf2l, Nr4a1, Nr4a3, Plagl1, Plekhg5, Ptgs2, Tiam1
GO:0010647	positive regulation of cell communication	6h, 12h, 24h	13	Arpp19, Bag4, Cited2, Cyr61, Hbegf, Homer1, Kitl, Neurod2, Plekhg5, Plk2, Ptgs2, Wnt2, Zeb2
GO:0007264	small GTPase mediated signal transduction	6h, 12h	12	Arhgap20, Arhgef2, Arl5b, Cdkn1a, Iqgap2, Ksr1, Mcf2l, Plekhg5, Plk2, Rrad, Siah2, Tiam1
GO:0043269	regulation of ion transport	6h, 12h, 24h	10	Cacna1h, Cxcl1, Homer1, Kcnf1, Kcnip2, Kcns2, Kcnv1, Nedd4l, P2ry12, Ptgs2
GO:0051592	response to calcium ion	6h, 12h, 24h	9	Dusp1, Fos, Fosb, Homer1, Jun, Junb, Kcnip2, Neurod2, Pcsk1
GO:0031960	response to corticosteroid stimulus	6h, 12h, 24h	9	Avpr1a, Cdkn1a, Cxcl1, Dusp1, Fos, Fosb, Junb, Pcsk1, Ptgs2
GO:0051056	regulation of small GTPase mediated signal transduction	6h, 12h	9	Arhgap20, Arhgef2, Arpp19, Iqgap2, Iqsec2, Kitl, Mcf2l, Plekhg5, Tiam1
GO:0051348	negative regulation of transferase activity	6h, 12h, 24h	8	Cdkn1a, Dusp1, Dusp5, Errfi1, Gadd45b, Gadd45g, Rgs2, Sh3bp5
GO:0019932	second-messenger-mediated signaling	6h, 12h, 24h	8	Avpr1a, Crem, Gucy1b3, Homer1, Htr1a, Mt1, P2ry12, Rcan1
GO:0007243	intracellular protein kinase cascade	6h, 12h, 24h	8	Errfi1, Fos, Jun, Map3k5, P2ry12, Scg2, Sh3bp5, Zfp36
GO:0071248	cellular response to metal ion	6h, 12h, 24h	7	Cacna1h, Fos, Fosb, Jun, Junb, Mt1, Neurod2

GO:0031644	regulation of neurological system process	6h, 12h	7	Arc, Avpr1a, Bdnf, Neurod2, Npy5r, Plk2, Ptgs2
GO:0008016	regulation of heart contraction	6h, 12h, 24h	6	Avpr1a, Cacna1h, Hbegf, Kcnip2, Rgs2, Smad7
GO:0045785	positive regulation of cell adhesion	6h, 12h, 24h	6	Bag4, Cited2, Cyr61, Selp, Smad7, Smoc2
GO:0050678	regulation of epithelial cell proliferation	6h, 12h	6	Foxp1, Jun, Nr4a1, Scg2, Tinf2, Wnt2
GO:0034765	regulation of ion transmembrane transport	6h, 12h, 24h	6	Cacna1h, Homer1, Kcnf1, Kcnip2, Kcns2, Kcnv1
GO:0048858	cell projection morphogenesis	6h, 24h	6	Bcl11b, Dclk1, Jun, Nr4a2, Rtn4r, Stxbp5
GO:0050804	regulation of synaptic transmission	6h, 12h	6	Arc, Bdnf, Neurod2, Npy5r, Plk2, Ptgs2
GO:0043401	steroid hormone mediated signaling pathway	6h, 12h	5	Nr3c2, Nr4a1, Nr4a2, Nr4a3, Rorb
GO:0071383	cellular response to steroid hormone stimulus	6h, 12h	5	Nr3c2, Nr4a1, Nr4a2, Nr4a3, Rorb
GO:0006470	protein dephosphorylation	6h	5	Dusp1, Dusp5, Mtmr7, Ptpn12, Ptpn3
GO:0010565	regulation of cellular ketone metabolic process	6h	5	Arpp19, Avpr1a, Odc1, Pdk4, Ptgs2
GO:0051970	negative regulation of transmission of nerve impulse	6h	4	Avpr1a, Npy5r, Plk2, Ptgs2
GO:0031645	negative regulation of neurological system process	6h, 12h	4	Avpr1a, Npy5r, Plk2, Ptgs2
GO:0048660	regulation of smooth muscle cell proliferation	6h, 12h, 24h	4	Hbegf, Jun, Npy5r, Ptgs2

GO:0051403	stress-activated MAPK cascade	6h, 12h, 24h	4	Fos, Jun, Map3k5, Sh3bp5
GO:0046890	regulation of lipid biosynthetic process	6h, 24h	4	Avpr1a, Cyp51, Cyr61, Ptgs2
GO:0060716	labyrinthine layer blood vessel development	6h	3	Cyr61, Junb, Wnt2
GO:0048745	smooth muscle tissue development	6h	3	5830428H23Rik, Foxp1, Tiparp
GO:0000080	G1 phase of mitotic cell cycle	6h	3	Cdkn1a, E2f3, Tfdp2
GO:0032570	response to progesterone stimulus	6h, 12h, 24h	3	Fos, Fosb, Junb
GO:0048145	regulation of fibroblast proliferation	6h, 12h, 24h	3	Cdkn1a, Jun, Wnt2
GO:0061041	regulation of wound healing	6h, 12h, 24h	3	Anxa2, Hbegf, Selp
GO:0002573	myeloid leukocyte differentiation	6h	3	Cited2, Jun, Junb
GO:0014032	neural crest cell development	6h	2	Cited2, Rdh10
GO:0045932	negative regulation of muscle contraction	6h, 24h	2	Ptgs2, Rgs2
GO:0060043	regulation of cardiac muscle cell proliferation	6h	2	Foxp1, Wnt2
GO:0045907	positive regulation of vasoconstrictio n	6h, 12h	2	Avpr1a, Ptgs2
GO:0055002	striated muscle cell development	6h, 12h	2	Homer1, Rcan1
GO:0014072	response to isoquinoline alkaloid	6h, 12h	2	Fosb, Pcsk1
GO:0043392	negative regulation of DNA binding	6h, 12h	2	Jun, Zfp462
GO:0050873	brown fat cell differentiation	6h	2	Ptgs2, Rgs2

GO:0001975	response to amphetamine	6h, 12h	2	Cxcl1, Nr4a2
GO:0006040	amino sugar metabolic process	6h	2	Nanp, St6gal2
GO:0030282	bone mineralization	6h	2	Gpnmb, Ptgs2
GO:0045933	positive regulation of muscle contraction	6h, 12h	2	Ptgs2, Rgs2
