# Supplementary material for: Glial responses during epileptogenesis in Mus musculus point to potential therapeutic targets
Source: PLoS One. 2018 Aug 16;13(8):e0201742. doi: 10.1371/journal.pone.0201742 (PMC6095496; doi:10.1371/journal.pone.0201742)
Supplement: S6 Table — All significantly changed genes at 12h were considered, and a threshold of p-value <0.05 was applied. (PDF) [file pone.0201742.s010.pdf]

**Table S6:** Significantly changed GO Biological Processes (level 6) at 12 hours post KA treatment, using the "Mapping to ontologies (TRANSPATH)" analysis tool. All significantly changed genes at 12h were considered, and a threshold of p-value <0.05 was applied.

| **Gene Ontology Category ID** | **GO-Biologic Process (level 6)** | **Time point(s) of enrichment** | **Number of significantly changed genes at 12h** | **Symbol of significantly changed genes at 12h** |
|---|---|---|---|---|
| GO:0031326 | regulation of cellular biosynthetic process | 6h, 12h, 24h | 156 | Abt1, Adcyap1r1, Aff1, Akap5, Anxa3, Ar, Areg, Arhgef2, Arpp19, Atrx, Bach1, Baz1a, Bcl11a, Bcl11b, Bcl3, Bcl7a, Bmyc, Camk1d, Camk2a, Camkk2, Capn3, Cav1, Ccl4, Cdkn1a, Cdyl, Cebpd, Cited2, Crem, Crh, Crtc1, Csrnp1, Cyld, Cyr61, Dbp, Eaf1, Eaf2, Egr2, Eif2c2, Eif4e2, Eif4ebp1, Ell2, Eng, Etf1, Etv3, Fam120b, Fos, Fosb, Fosl1, Fosl2, Foxk1, Glis3, Grin1, Grm5, Hbegf, Hmga1, Hmgb2, Hmox1, Hspb1, Htr1a, Icam1, Id2, Igf2bp2, Il11, Il16, Il6, Jarid2, Jun, Junb, Kcnh3, Kdm6b, Kitl, Klf5, Klf6, Lefty1, Lhx9, Litaf, Lmo1, Lrrfip1, Maff, Magoh, Map2k3, Mbd2, Mcf2l, Med13, Mef2a, Met, Mterfd3, Myc, Nat14, Ncor1, Neurod6, Nfatc1, Nfe2l2, Nfil3, Nfkbia, Nfkbiz, Nos3, Npas4, Npy2r, Nr1d2, Nr2c2, Nr3c2, Nr4a2, Nrip1, Nrip2, Oprl1, Pak6, Pcgf5, Pdcd4, Pdk4, Pdlim1, Phlda1, Pik3r1, Pim1, Plagl1, Plek, Ppp3ca, Psip1, Ptgs2, Ptk2b, Rasl11a, Rcan1, Rgs1, Rgs2, Rhoc, Ripk1, Rora, Rorb, Rps6ka3, Rps6ka5, S100a11, S1pr3, Samd4, Sap18, Sap30, Scai, Sertad1, Sphk1, Ssbp2, Sstr2, Stat3, Tal1, Tesc, Thbs1, Tinf2, Tlr2, Tnfrsf1a, Wwtr1, Zeb2, Zfp239, Zfp295, Zfp36, Zfp462, Zfp516, Zkscan4, Zmiz1 |

| | | | | |
|---|---|---|---|---|
| GO:0019219 | regulation of nucleobase-containing compound metabolic process | 6h, 12h, 24h | 155 | Abt1, Adcyap1r1, Aff1, Akap5, Anxa3, Ar, Areg, Arhgap15, Arhgef2, Atrx, Bach1, Baz1a, Bcl11a, Bcl11b, Bcl3, Bcl7a, Bmyc, Camk1d, Camk2a, Camkk2, Capn3, Cav1, Ccl11, Ccl4, Cdkn1a, Cdyl, Cebpd, Chn1, Cited2, Cnn3, Crem, Crh, Crtc1, Csrnp1, Cyld, Cyr61, D630037F22Rik, Dbp, Eaf1, Eaf2, Egr2, Eif2c2, Ell2, Eng, Epha3, Epha4, Errfi1, Etv3, Fam120b, Fos, Fosb, Fosl1, Fosl2, Foxk1, Glis3, Grin1, Grm5, Hmga1, Hmgb2, Hmox1, Htr1a, Icam1, Id2, Il11, Il16, Il6, Jarid2, Jun, Junb, Kcnh3, Kdm6b, Kitl, Klf5, Klf6, Lefty1, Lhx9, Litaf, Lmo1, Lrrfip1, Maff, Map2k3, Mbd2, Mcf2l, Med13, Mef2a, Met, Mterfd3, Myc, Myo9b, Nat14, Ncor1, Neurod6, Nfatc1, Nfe2l2, Nfil3, Nfkbia, Nfkbiz, Nos3, Npas4, Npy2r, Nr1d2, Nr2c2, Nr3c2, Nr4a2, Nrip1, Nrip2, Oprl1, Pak6, Pcgf5, Pdcd4, Pdlim1, Phlda1, Pik3r1, Pim1, Plagl1, Ppp3ca, Prkcc, Psip1, Ptk2b, Rabgap1l, Rasl11a, Rcan1, Rgs1, Rgs2, Rgs4, Ripk1, Rora, Rorb, Rps6ka3, Rps6ka5, S100a11, S1pr3, Sap18, Sap30, Scai, Sertad1, Sphk1, Srsf12, Ssbp2, Sstr2, Stat3, Tal1, Tesc, Tinf2, Tlr2, Tnfrsf1a, Wwtr1, Zeb2, Zfp239, Zfp295, Zfp36, Zfp462, Zfp516, Zkscan4, Zmiz1 |

| | | | | |
|---|---|---|---|---|
| GO:0010556 | regulation of macromolecule biosynthetic process | 6h, 12h, 24h | 143 | Abt1, Aff1, Anxa3, Ar, Areg, Arhgef2, Atrx, Bach1, Baz1a, Bcl11a, Bcl11b, Bcl3, Bcl7a, Bmyc, Camk1d, Camk2a, Camkk2, Capn3, Cav1, Ccl2, Cdkn1a, Cdyl, Cebpd, Cited2, Crem, Crtc1, Csrnp1, Cyld, Cyr61, Dbp, Eaf1, Eaf2, Egr2, Eif2c2, Eif4e2, Eif4ebp1, Ell2, Eng, Etf1, Etv3, Fam120b, Fos, Fosb, Fosl1, Fosl2, Foxk1, Glis3, Grin1, Grm5, Hbegf, Hmga1, Hmgb2, Hmox1, Hspb1, Hsph1, Icam1, Id2, Igf2bp2, Il11, Il16, Il6, Jarid2, Jun, Junb, Kcnh3, Kdm6b, Kitl, Klf5, Klf6, Lefty1, Lhx9, Litaf, Lmo1, Lrrfip1, Maff, Magoh, Map2k3, Mbd2, Mcf2l, Med13, Mef2a, Met, Mterfd3, Myc, Nat14, Ncor1, Neurod6, Nfatc1, Nfe2l2, Nfil3, Nfkbia, Nfkbiz, Npas4, Nr1d2, Nr2c2, Nr3c2, Nr4a2, Nrip1, Nrip2, Pak6, Pcgf5, Pdcd4, Pdlim1, Phlda1, Pik3r1, Pim1, Plagl1, Ppp3ca, Psip1, Ptk2b, Rasl11a, Rcan1, Rgs2, Rhoc, Ripk1, Rora, Rorb, Rps6ka3, Rps6ka5, S100a11, Samd4, Sap18, Sap30, Scai, Sertad1, Sphk1, Ssbp2, Stat3, Tal1, Tesc, Thbs1, Tinf2, Tlr2, Tnfrsf1a, Wwtr1, Zeb2, Zfp239, Zfp295, Zfp36, Zfp462, Zfp516, Zkscan4, Zmiz1 |

| | | | | |
|---|---|---|---|---|
| GO:0010468 | regulation of gene expression | 6h, 12h, 24h | 142 | Abt1, Aff1, Anxa3, Ar, Arhgef2, Atrx, Bach1, Baz1a, Bcl11a, Bcl11b, Bcl3, Bcl7a, Bmyc, Camk1d, Camk2a, Camkk2, Capn3, Cartpt, Cav1, Cd44, Cdkn1a, Cdyl, Cebpd, Cited2, Cntn1, Crem, Crh, Crtc1, Csrnp1, Cyld, Cyr61, Dbp, Eaf1, Eaf2, Egr2, Eif2c2, Eif4e2, Eif4ebp1, Ell2, Eng, Etf1, Etv3, Fam120b, Fos, Fosb, Fosl1, Fosl2, Foxk1, Glis3, Grin1, Grm5, Hmga1, Hmgb2, Hmox1, Hspb1, Icam1, Id2, Igf2bp2, Il11, Il16, Il6, Jarid2, Jun, Junb, Kcnh3, Kdm6b, Klf5, Klf6, Lefty1, Lhx9, Litaf, Lmo1, Lrrfip1, Maff, Magoh, Map2k3, Mbd2, Mcf2l, Med13, Mef2a, Met, Mterfd3, Myc, Nat14, Ncor1, Neurod6, Nfatc1, Nfe2l2, Nfil3, Nfkbia, Nfkbiz, Npas4, Nr1d2, Nr2c2, Nr3c2, Nr4a2, Nrip1, Nrip2, Pak6, Pcgf5, Pdcd4, Pdlim1, Phlda1, Pik3r1, Pim1, Plagl1, Ppp3ca, Psip1, Ptk2b, Rasl11a, Rcan1, Rgs2, Rhoc, Ripk1, Rora, Rorb, Rps6ka3, Rps6ka5, Samd4, Sap18, Sap30, Scai, Serpine1, Sertad1, Sphk1, Srsf12, Ssbp2, Stat3, Tal1, Tesc, Thbs1, Tlr2, Tnfrsf1a, Wwtr1, Zeb2, Zfp239, Zfp295, Zfp36, Zfp462, Zfp516, Zkscan4, Zmiz1 |

| | | | | |
|---|---|---|---|---|
| GO:0051252 | regulation of RNA metabolic process | 6h, 12h, 24h | 126 | Abt1, Aff1, Anxa3, Ar, Arhgef2, Atrx, Bach1, Baz1a, Bcl11a, Bcl11b, Bcl3, Bcl7a, Bmyc, Camk1d, Camk2a, Camkk2, Capn3, Cav1, Ccl4, Cdyl, Cebpd, Cited2, Crem, Crtc1, Csrnp1, Cyld, Cyr61, Dbp, Eaf1, Eaf2, Egr2, Eif2c2, Ell2, Eng, Etv3, Fam120b, Fos, Fosb, Fosl1, Fosl2, Foxk1, Glis3, Grin1, Grm5, Hmga1, Hmgb2, Hmox1, Icam1, Id2, Il11, Il16, Il6, Jarid2, Jun, Junb, Kcnh3, Kdm6b, Klf5, Klf6, Lefty1, Lhx9, Litaf, Lmo1, Lrrfip1, Maff, Map2k3, Mbd2, Mcf2l, Med13, Mef2a, Met, Mterfd3, Myc, Nat14, Ncor1, Neurod6, Nfatc1, Nfe2l2, Nfil3, Nfkbia, Nfkbiz, Npas4, Nr1d2, Nr2c2, Nr3c2, Nr4a2, Nrip1, Nrip2, Pak6, Pcgf5, Pdcd4, Pdlim1, Phlda1, Pik3r1, Pim1, Plagl1, Ppp3ca, Psip1, Rasl11a, Rcan1, Ripk1, Rora, Rorb, Rps6ka3, Rps6ka5, Sap18, Sap30, Scai, Sertad1, Sphk1, Srsf12, Ssbp2, Stat3, Tal1, Tesc, Tlr2, Tnfrsf1a, Wwtr1, Zeb2, Zfp239, Zfp295, Zfp36, Zfp462, Zfp516, Zkscan4, Zmiz1 |
| GO:0010604 | positive regulation of macromolecule metabolic process | 6h, 12h, 24h | 95 | Aff1, Akap5, Anxa3, Ar, Areg, Bcl11a, Bcl11b, Bcl3, Bmyc, Camkk2, Capn3, Cav1, Ccl2, Cd44, Cdkn1a, Cebpd, Cited2, Cntn1, Crh, Crtc1, Csrnp1, Cyr61, Dbp, Eaf1, Eaf2, Egr2, Ell2, Eng, Fos, Fosl1, Fosl2, Foxk1, Glis3, Grin1, Hmga1, Hmgb2, Hmox1, Hspa5, Hspb1, Hsph1, Id2, Ier3, Il11, Il6, Jarid2, Jun, Junb, Kitl, Klf5, Klf6, Lmo1, Map2k3, Mapk8ip2, Mcf2l, Med13, Mef2a, Met, Myc, Nat14, Ncor1, Nfatc1, Nfe2l2, Nfil3, Nfkbia, Npas4, Nr2c2, Nr4a2, Nrip1, Pik3r1, Plagl1, Ppp3ca, Prkcc, Psip1, Ptk2b, Rasl11a, Rhoc, Ripk1, Rora, Rorb, Rps6ka3, Rps6ka5, Samd4, Sertad1, Sphk1, Stat3, Tal1, Tesc, Thbs1, Tinf2, Tlr2, Tnfrsf1a, Wwtr1, Zfp36, Zfp462, Zmiz1 |

| | | | | |
|---|---|---|---|---|
| GO:0031325 | positive regulation of cellular metabolic process | 6h, 12h, 24h | 94 | Adcyap1r1, Aff1, Akap5, Anxa3, Ar, Areg, Arpp19, Bcl11a, Bcl11b, Bcl3, Bmyc, Camkk2, Capn3, Cav1, Cd44, Cdkn1a, Cebpd, Cited2, Crh, Crtc1, Csrnp1, Cyr61, Dbp, Eaf1, Eaf2, Egr2, Ell2, Eng, Fos, Fosl1, Fosl2, Foxk1, Glis3, Grin1, Hmga1, Hmgb2, Hmox1, Hspa5, Hspb1, Icam1, Id2, Il11, Il6, Jarid2, Jun, Junb, Kitl, Klf5, Klf6, Lmo1, Map2k3, Mapk8ip2, Mcf2l, Med13, Mef2a, Met, Myc, Nat14, Ncor1, Nfatc1, Nfe2l2, Nfkbia, Npas4, Nr2c2, Nr4a2, Nrip1, Pik3r1, Plagl1, Ppp3ca, Prkcc, Psip1, Ptgs2, Ptk2b, Rasl11a, Rhoc, Ripk1, Rora, Rorb, Rps6ka3, Rps6ka5, Samd4, Sertad1, Sphk1, Stat3, Tal1, Tesc, Thbs1, Tinf2, Tlr2, Tnfrsf1a, Wwtr1, Zfp36, Zfp462, Zmiz1 |
| GO:0009891 | positive regulation of biosynthetic process | 6h, 12h, 24h | 85 | Adcyap1r1, Aff1, Akap5, Ar, Areg, Arpp19, Bcl11a, Bcl11b, Bcl3, Bmyc, Camkk2, Capn3, Ccl2, Cebpd, Cited2, Crh, Crtc1, Csrnp1, Cyr61, Dbp, Eaf1, Eaf2, Egr2, Ell2, Eng, Fos, Fosl1, Fosl2, Foxk1, Glis3, Grin1, Hmga1, Hmgb2, Hmox1, Hspb1, Hsph1, Icam1, Id2, Il11, Il6, Jun, Junb, Kitl, Klf5, Klf6, Lmo1, Map2k3, Mcf2l, Med13, Mef2a, Met, Myc, Nat14, Nfatc1, Nfe2l2, Nfkbia, Npas4, Nr2c2, Nr4a2, Nrip1, Pik3r1, Plagl1, Ppp3ca, Psip1, Ptgs2, Ptk2b, Rasl11a, Rhoc, Ripk1, Rora, Rorb, Rps6ka3, Rps6ka5, Samd4, Sertad1, Stat3, Tal1, Tesc, Thbs1, Tlr2, Tnfrsf1a, Wwtr1, Zfp36, Zfp462, Zmiz1 |
| GO:0051173 | positive regulation of nitrogen compound metabolic process | 6h, 12h, 24h | 79 | Adcyap1r1, Aff1, Akap5, Anxa3, Ar, Areg, Bcl11a, Bcl11b, Bcl3, Bmyc, Camkk2, Capn3, Cebpd, Cited2, Crh, Crtc1, Csrnp1, Cyr61, Dbp, Eaf1, Eaf2, Egr2, Ell2, Eng, Fos, Fosl1, Fosl2, Foxk1, Glis3, Grin1, Hmga1, Hmgb2, Icam1, Id2, Il11, Il6, Jun, Junb, Kitl, Klf5, Klf6, Lmo1, Map2k3, Mcf2l, Med13, Mef2a, Met, Myc, Nat14, Nfatc1, Nfe2l2, Nfkbia, Npas4, Nr2c2, Nr4a2, Nrip1, Pik3r1, Plagl1, Ppp3ca, Prkcc, Psip1, Ptgs2, Rasl11a, Ripk1, Rora, Rorb, Rps6ka3, Rps6ka5, Sertad1, Stat3, Tal1, Tesc, Tinf2, Tlr2, Tnfrsf1a, Wwtr1, Zfp36, Zfp462, Zmiz1 |

| | | | | |
|---|---|---|---|---|
| GO:0043067 | regulation of programmed cell death | 6h, 12h, 24h | 78 | Aldh1a3, Ar, Arhgef2, Arhgef4, Bag3, Bcl11b, Bcl2a1a, Bcl2a1d, Bcl3, Bdnf, Birc3, Camk1d, Camk2b, Capn3, Cav1, Ccl2, Cd44, Cdkn1a, Cflar, Ciapin1, Cited2, Crh, Cstb, Cyld, Cyr61, Dusp1, Eaf2, Faim2, Fosl1, Gabra5, Grin1, Hmox1, Hspa1a, Hspa1b, Hspa5, Hspb1, Hsph1, Ier3, Il6, Jun, Kalrn, Kitl, Lmna, Ltbr, Mcf2l, Mcl1, Myc, Nell1, Nfkbia, Nmt1, Nos3, Nr4a2, Pde1a, Phlda1, Pik3r1, Pim1, Plagl1, Prkcc, Ptgs2, Ptk2b, Rasgrf2, Rhoc, Rilpl1, Ripk1, Rps6ka3, Ryr2, Scg2, Serpine1, Siah2, Socs3, Sphk1, Spp1, Tgm2, Thbs1, Timp1, Tlr2, Tnfrsf12a, Tnfrsf1a |
| GO:0051246 | regulation of protein metabolic process | 6h, 12h, 24h | 74 | Akap5, Bcl3, Camkk2, Capn3, Cartpt, Cav1, Ccl2, Cd44, Cdkn1a, Crh, Cyr61, Dgkh, Dlgap1, Dusp1, Edem1, Eif2c2, Eif4e2, Eif4ebp1, Eng, Epha4, Errfi1, Etf1, Gadd45b, Gadd45g, Grm1, Grm5, Hmox1, Hspa5, Hspb1, Ier3, Igf2bp2, Il11, Il6, Jarid2, Jun, Kitl, Lmna, Lrp8, Magoh, Map2k3, Map3k6, Mapk8ip2, Met, Ncor1, Nedd4l, Nfkbia, Pdcd4, Pik3r1, Pim1, Plce1, Prkar2a, Prkcc, Ptk2b, Rgs2, Rgs4, Rhoc, Ripk1, Rps6ka3, Rps6ka5, Samd4, Serpina3n, Serpine1, Sertad1, Siah2, Socs3, Sphk1, Thbs1, Timp1, Tinf2, Tlr2, Tnfrsf1a, Tnik, Wwtr1, Zeb2 |
| GO:0006351 | transcription, DNA-dependent | 6h, 12h | 72 | Abt1, Aff1, Ar, Bach1, Baz1a, Bcl11a, Bcl11b, Bcl3, Cdyl, Cebpd, Col4a2, Crem, Crtc1, Csrnp1, Dbp, Eaf1, Eaf2, Egr2, Eif2c2, Ell2, Etv3, Fam120b, Fos, Fosb, Fosl1, Fosl2, Foxk1, Glis3, Il16, Jarid2, Jun, Junb, Klf5, Litaf, Lrrfip1, Maff, Magoh, Mbd2, Med13, Mef2a, Mterfd3, Myc, Nat14, Ncor1, Neurod6, Nfatc1, Nfe2l2, Nfil3, Nfkbiz, Npas4, Nr1d2, Nr2c2, Nr3c2, Nr4a2, Nrip1, Nrip2, Pcgf5, Psip1, Rasl11a, Rora, Rorb, Sap30, Scai, Sertad1, Ssbp2, Stat3, Tal1, Wwtr1, Zeb2, Zfp239, Zfp516, Zkscan4 |

| | | | | |
|---|---|---|---|---|
| GO:0032268 | regulation of cellular protein metabolic process | 6h, 12h, 24h | 69 | Akap5, Bcl3, Camkk2, Capn3, Cartpt, Cav1, Ccl2, Cd44, Cdkn1a, Crh, Cyr61, Dgkh, Dlgap1, Dusp1, Edem1, Eif2c2, Eif4e2, Eif4ebp1, Eng, Epha4, Errfi1, Etf1, Gadd45b, Gadd45g, Grm1, Grm5, Hspa5, Hspb1, Igf2bp2, Il11, Il6, Jarid2, Jun, Kitl, Lmna, Lrp8, Magoh, Map2k3, Map3k6, Mapk8ip2, Met, Ncor1, Nfkbia, Pdcd4, Pik3r1, Pim1, Plce1, Prkar2a, Prkcc, Ptk2b, Rgs2, Rgs4, Rhoc, Ripk1, Rps6ka3, Rps6ka5, Samd4, Serpine1, Sertad1, Siah2, Socs3, Sphk1, Thbs1, Timp1, Tinf2, Tnfrsf1a, Tnik, Wwtr1, Zeb2 |
| GO:0031324 | negative regulation of cellular metabolic process | 6h, 12h, 24h | 64 | Bach1, Bcl3, Bcl7a, Camk2n1, Capn3, Cav1, Ccl4, Cdkn1a, Cebpd, Cited2, Eif2c2, Eif4ebp1, Eng, Errfi1, Etv3, Fosb, Foxk1, Glis3, Hbegf, Hmga1, Hmgb2, Hspb1, Htr1a, Id2, Ier3, Igf2bp2, Il6, Jarid2, Jun, Kdm6b, Lefty1, Lhx9, Lmo1, Lrrfip1, Mbd2, Mef2a, Met, Myc, Ncor1, Nfil3, Npy2r, Nrip1, Nrip2, Oprl1, Pdcd4, Pik3r1, Plek, Prkcc, Rgs1, Rps6ka5, S100a11, S1pr3, Samd4, Sap30, Serpine1, Srsf12, Sstr2, Stat3, Tal1, Timp1, Tinf2, Wwtr1, Zfp295, Zfp36 |
| GO:0010605 | negative regulation of macromolecule metabolic process | 6h, 12h, 24h | 59 | Ar, Bach1, Bcl11a, Bcl3, Bcl7a, Capn3, Cav1, Ccl4, Cdkn1a, Cebpd, Cited2, Crh, Eif2c2, Eif4ebp1, Eng, Errfi1, Etv3, Fosb, Foxk1, Glis3, Hbegf, Hmga1, Hmgb2, Id2, Ier3, Igf2bp2, Il6, Jarid2, Jun, Kdm6b, Lefty1, Lhx9, Lmo1, Lrrfip1, Mbd2, Mef2a, Met, Myc, Ncor1, Nfil3, Nrip1, Nrip2, Pdcd4, Pik3r1, Prkcc, Rps6ka5, S100a11, Samd4, Sap30, Serpine1, Srsf12, Stat3, Tal1, Timp1, Tinf2, Tnfrsf1a, Wwtr1, Zfp295, Zfp36 |
| GO:0009890 | negative regulation of biosynthetic process | 6h, 12h, 24h | 55 | Ar, Bach1, Bcl3, Bcl7a, Capn3, Cav1, Ccl4, Cebpd, Cited2, Eif2c2, Eif4ebp1, Eng, Etv3, Fosb, Foxk1, Glis3, Hbegf, Hmga1, Hmgb2, Htr1a, Id2, Igf2bp2, Il6, Jarid2, Jun, Kdm6b, Lefty1, Lhx9, Lmo1, Lrrfip1, Mbd2, Mef2a, Met, Myc, Ncor1, Nfil3, Npy2r, Nrip1, Nrip2, Oprl1, Pdcd4, Plek, Rgs1, Rps6ka5, S100a11, S1pr3, Samd4, Sap30, Sstr2, Stat3, Tal1, Tinf2, Wwtr1, Zfp295, Zfp36 |

| | | | | |
|---|---|---|---|---|
| GO:0051174 | regulation of phosphorus metabolic process | 6h, 12h, 24h | 55 | Akap5, Ar, Areg, Camk2n1, Camkk2, Cartpt, Cav1, Ccl2, Cd44, Cdkn1a, Crh, Cyr61, Dgkh, Dusp1, Edem1, Elfn2, Eng, Epha4, Errfi1, Gadd45b, Gadd45g, Grm1, Grm5, Hspa5, Hspb1, Il11, Il6, Jun, Kitl, Lmna, Lrp8, Map2k3, Map3k6, Mapk8ip2, Met, Pdcd4, Pik3r1, Pim1, Plce1, Plek, Prkar2a, Ptk2b, Rcan1, Rgs2, Rgs4, Ripk1, Rps6ka5, Sertad1, Socs3, Sphk1, Thbs1, Tnfrsf1a, Tnik, Wwtr1, Zeb2 |
| GO:0016310 | phosphorylation | 12h, 24h | 50 | Ak5, Akt3, Camk1d, Camk2a, Camk2b, Camkk2, Cartpt, Ccl11, Ccl2, Cdkn1a, Dclk1, Dclk3, Epha3, Epha4, Gadd45b, Gadd45g, Grm1, Grm5, Itpka, Kalrn, Kcnh3, Map2k3, Map3k6, Map4k4, Mapk4, Mapk8ip2, Met, Pak6, Pdk4, Pik3c3, Pik3r1, Pim1, Pip4k2c, Plce1, Pmvk, Prkar2a, Prkcc, Prkx, Ptk2b, Ripk1, Rps6ka3, Rps6ka5, Rps6ka6, Sik2, Speg, Sphk1, Thbs1, Tlk2, Tlr2, Tnik |
| GO:0060548 | negative regulation of cell death | 6h, 12h, 24h | 48 | Adcyap1r1, Ar, Bag3, Bcl11b, Bcl2a1a, Bcl2a1d, Bcl3, Bdnf, Birc3, Capn3, Ccl2, Cd44, Cdkn1a, Cflar, Ciapin1, Cited2, Crh, Cyr61, Faim2, Grin1, Hmox1, Hspa1a, Hspa1b, Hspa5, Hspb1, Hsph1, Ier3, Il6, Jun, Kitl, Mcl1, Myc, Nfkbia, Nos3, Nr4a2, Pik3r1, Pim1, Prkcc, Ptk2b, Rps6ka3, Scg2, Serpine1, Siah2, Socs3, Sphk1, Spp1, Thbs1, Timp1 |
| GO:0051172 | negative regulation of nitrogen compound metabolic process | 6h, 12h, 24h | 48 | Bach1, Bcl3, Bcl7a, Capn3, Cav1, Ccl4, Cebpd, Cited2, Eng, Etv3, Fosb, Foxk1, Glis3, Hmga1, Hmgb2, Htr1a, Id2, Jarid2, Jun, Kdm6b, Lefty1, Lhx9, Lmo1, Lrrfip1, Mbd2, Mef2a, Met, Myc, Ncor1, Nfil3, Npy2r, Nrip1, Nrip2, Oprl1, Pdcd4, Rgs1, Rps6ka5, S100a11, S1pr3, Sap30, Srsf12, Sstr2, Stat3, Tal1, Tinf2, Wwtr1, Zfp295, Zfp36 |
| GO:0006468 | protein phosphorylation | 12h, 24h | 42 | Akt3, Camk1d, Camk2a, Camk2b, Camkk2, Cartpt, Ccl11, Ccl2, Dclk1, Dclk3, Epha3, Epha4, Gadd45b, Gadd45g, Grm1, Grm5, Itpka, Kalrn, Map2k3, Map3k6, Map4k4, Mapk4, Met, Pak6, Pdk4, Pik3c3, Pik3r1, Pim1, Plce1, Prkcc, Prkx, Ptk2b, Ripk1, Rps6ka3, Rps6ka5, Rps6ka6, Sik2, Speg, Thbs1, Tlk2, Tlr2, Tnik |

| | | | | |
|---|---|---|---|---|
| GO:0010647 | positive regulation of cell communication | 6h, 12h, 24h | 41 | AI464131, Akap5, Aldh1a3, Ar, Arpp19, Camk2b, Cartpt, Cav1, Ccl2, Ccl4, Cd44, Cflar, Cited2, Crh, Cyld, Cyr61, Eng, Gria1, Hbegf, Hmox1, Homer1, Il11, Il6, Kitl, Litaf, Ltbr, Mapk8ip2, Mbd2, Nrxn1, Ptgs2, Ptk2b, Rhoc, Ripk1, Slc24a2, Stat3, Tgm2, Thbs1, Tlr2, Tnfrsf12a, Tnfrsf1a, Zeb2 |
| GO:0010942 | positive regulation of cell death | 6h, 12h, 24h | 39 | Aldh1a3, Arhgef2, Arhgef4, Bcl3, Camk2b, Cav1, Cd44, Cdkn1a, Cflar, Crh, Cyld, Cyr61, Dusp1, Eaf2, Fosl1, Grin1, Hmox1, Ier3, Jun, Kalrn, Ltbr, Mcf2l, Myc, Nell1, Nmt1, Nos3, Phlda1, Pik3r1, Plagl1, Ptgs2, Rasgrf2, Rhoc, Ripk1, Ryr2, Tgm2, Thbs1, Tlr2, Tnfrsf12a, Tnfrsf1a |
| GO:0043269 | regulation of ion transport | 6h, 12h, 24h | 39 | Abcc8, Adcyap1r1, Akap5, Akt3, Atp2b2, Cacna1b, Cacna1d, Cacna1h, Camk2b, Capn3, Cav1, Ccl4, Cntn1, Crh, Cybb, Fxyd7, Homer1, Icam1, Kcna2, Kcnab1, Kcnc1, Kcnc2, Kcnf1, Kcnh3, Kcnip2, Kcnip4, Kcnj3, Kcnj9, Kcnq5, Nalcn, Nedd4l, Nos3, Ptgs2, Ptk2b, Rhoc, Ryr2, Scn1a, Scn2a1, Scn3b |
| GO:0043549 | regulation of kinase activity | 6h, 12h, 24h | 39 | Camkk2, Cartpt, Cav1, Ccl2, Cdkn1a, Cyr61, Dgkh, Dusp1, Edem1, Epha4, Errfi1, Gadd45b, Gadd45g, Grm1, Grm5, Hspa5, Hspb1, Il6, Kitl, Lmna, Lrp8, Map2k3, Map3k6, Met, Pdcd4, Pik3r1, Pim1, Plce1, Prkar2a, Ptk2b, Rgs2, Rgs4, Ripk1, Sertad1, Sphk1, Thbs1, Tnik, Wwtr1, Zeb2 |
| GO:0050801 | ion homeostasis | 12h, 24h | 38 | Atp2b2, Bdnf, C3ar1, Cacna1b, Cav1, Ccl11, Ccl2, Ccl7, Crh, Gpr12, Gria1, Grin1, Grm1, Grm5, Hmox1, Jun, Kcnc2, Mapk8ip2, Mt2, Myc, Nedd4l, Nlgn3, Npy2r, Nr3c2, Nrxn1, Oprl1, Plce1, Ppp3ca, Ptk2b, Ryr2, S1pr3, Scn1a, Scn2a1, Scn3b, Slc12a5, Slc24a2, Tesc, Tgm2 |
| GO:0030001 | metal ion transport | 12h, 24h | 37 | Abcc8, Atp2b1, Atp2b2, Cacna1b, Cacna1d, Cacna1h, Camk2a, Camk2b, Cav1, Grin1, Kcna2, Kcnab1, Kcnc1, Kcnc2, Kcnf1, Kcnh3, Kcnip2, Kcnip4, Kcnj3, Kcnj9, Kcnq5, Kctd4, Nalcn, Nedd4l, Nfatc1, Npy, Ppp3ca, Ryr2, Scn1a, Scn2a1, Scn3b, Slc10a6, Slc12a5, Slc24a2, Slc39a10, Slc5a3, Slc6a8 |

| | | | | |
|---|---|---|---|---|
| GO:0008284 | positive regulation of cell proliferation | 6h, 12h, 24h | 37 | Ar, Areg, Bmyc, Ccl11, Ccl2, Cdkn1a, Crh, Cyba, Cyp7b1, Cyr61, Fosl1, Fosl2, Hbegf, Hmgb2, Hmox1, Htr1a, Id2, Il11, Il13ra1, Il6, Jun, Kitl, Klf5, Myc, Npy5r, Odc1, Osmr, Ptgs2, Ptk2b, S1pr3, Scg2, Sertad1, Sphk1, Tgm2, Timp1, Wwtr1, Zmiz1 |
| GO:0007167 | enzyme linked receptor protein signaling pathway | 6h, 12h, 24h | 36 | Adcyap1r1, Areg, Arhgef2, Arhgef4, Ccl2, Chn1, Cited2, Crim1, Csrnp1, Eif4ebp1, Eng, Epha3, Epha4, Fos, Gfra1, Hbegf, Jun, Kalrn, Lefty1, Mcf2l, Mef2a, Met, Nfkbia, Pde1a, Pik3c3, Pik3r1, Plce1, Prkar2a, Ptk2b, Rasgrf2, Rps6ka3, Rps6ka5, Ryr2, Sik2, Stat3, Tiparp |
| GO:0008285 | negative regulation of cell proliferation | 12h, 24h | 35 | Adamts1, Ar, Atp2b1, Bcl11b, Bdnf, Cav1, Cdkn1a, Eaf2, Eng, Fabp3, Fosl1, Hmga1, Hmox1, Hspa1a, Hspa1b, Id2, Igfbp7, Il6, Jarid2, Jun, Kifap3, Lefty1, Myc, Nos3, Pik3r1, Ptgs2, Ptk2b, S100a11, Scg2, Sesn1, Speg, Sstr2, Tesc, Thbs1, Tinf2 |
| GO:0009967 | positive regulation of signal transduction | 12h, 24h | 33 | AI464131, Akap5, Aldh1a3, Ar, Arpp19, Cav1, Ccl4, Cd44, Cflar, Cited2, Cyld, Cyr61, Eng, Hbegf, Hmox1, Homer1, Il11, Il6, Kitl, Litaf, Ltbr, Mapk8ip2, Mbd2, Ptk2b, Rhoc, Ripk1, Stat3, Tgm2, Thbs1, Tlr2, Tnfrsf12a, Tnfrsf1a, Zeb2 |
| GO:0006461 | protein complex assembly | 12h | 32 | Birc3, C1ql2, Caly, Capg, Capn3, Cav1, Crtc1, Cyba, Dgkh, Eprs, Fermt3, Hmga1, Hmox1, Il1rap, Kcna2, Kcnip2, Kcnq5, Kifap3, Mapk8ip2, Mbd2, Ndufaf3, Nell1, Nrxn1, Prkar2a, Prosapip1, Ptk2b, Ripk1, Sh3pxd2b, Srr, Tgm2, Tnfrsf1a, Tubgcp2 |
| GO:0007264 | small GTPase mediated signal transduction | 6h, 12h | 32 | Arf2, Arhgap12, Arhgap15, Arhgap20, Arhgap39, Arhgef2, Arhgef4, Arl15, Arl4d, Cdkn1a, Chn1, Diras1, Gem, Hmox1, Iqgap2, Kalrn, Kifap3, Mcf2l, Myo9b, Plce1, Rab27b, Rab9b, Rasgef1b, Rasgrf2, Rasgrp1, Rasl11a, Rhoc, Rhoj, Rnd3, Rrad, Siah2, Trim23 |
| GO:0010627 | regulation of intracellular protein kinase cascade | 12h, 24h | 31 | AI464131, Akap5, Ar, Capn3, Cav1, Cd44, Cflar, Cyr61, Epha4, Grm1, Hbegf, Hmox1, Hspb1, Il11, Il6, Litaf, Ltbr, Map3k6, Map4k4, Mapk8ip2, Myc, Ncor1, Pdcd4, Ptk2b, Rhoc, Ripk1, Rps6ka6, Tgm2, Thbs1, Tnfrsf1a, Zeb2 |

| | | | | |
|---|---|---|---|---|
| GO:0051345 | positive regulation of hydrolase activity | 12h, 24h | 30 | Adcyap1r1, Arhgap15, Cav1, Ccl11, Ccl2, Chn1, Cyr61, Edem1, Errfi1, Grm1, Grm5, Hmgb2, Homer1, Hspa5, Jun, Lmna, Myc, Myo9b, Pde1a, Plce1, Plek, Prkar2a, Ptk2b, Rabgap1l, Rgs1, Rgs2, Rgs4, Rhoc, Ripk1, Tgm2 |
| GO:0007010 | cytoskeleton organization | 12h, 24h | 29 | Arc, Arhgef2, Capn3, Cav1, Ccl11, Ccl2, Ccl7, Cnn3, Haus8, Lmna, Mef2a, Nav1, Ncor1, Nedd9, Pak6, Pclo, Pdlim7, Plce1, Plek, Ptk2b, Rhoj, Rnd3, Shroom2, Svil, Synpo, Tnik, Tubgcp2, Vim, Zwint |
| GO:0010648 | negative regulation of cell communication | 12h, 24h | 29 | Cav1, Cd44, Cyld, Cyp7b1, Eng, Errfi1, Fbn1, Grm5, Hspa5, Mcl1, Myc, Ncor1, Nfkbia, Npy2r, Npy5r, Plek, Ptgs2, Rgs1, Rgs2, Rgs4, Rhoc, Ripk1, Rps6ka6, Scai, Siah2, Slc24a2, Socs3, Thbs1, Wwtr1 |
| GO:0060284 | regulation of cell development | 12h, 24h | 28 | Akap5, Arhgef2, Bcl11a, Bdnf, Camk1d, Camk2b, Chn1, D130043K22Rik, Epha3, Epha4, Grin1, Grm5, Id2, Il6, Kalrn, Met, Pik3r1, Ptk2b, Rhoc, Rufy3, Slit1, Sphk1, Spp1, Tal1, Tnfrsf12a, Tnik, Vim, Wwtr1 |
| GO:0009117 | nucleotide metabolic process | 12h | 27 | Ak5, Ampd3, Arl4d, Atp13a5, Atp2b1, Atp2b2, Diras1, Dnm1, Dnm3, Gbp2, Gem, Gmpr, Gng2, Gucy1b3, Hspa5, Myo9b, Pde1a, Pde4b, Rab27b, Rasl11a, Rhoc, Rhoj, Rnd3, Rora, Rrad, Sult2b1, Trim23 |
| GO:0045597 | positive regulation of cell differentiation | 12h, 24h | 27 | Adamts9, Akap5, Bcl11a, Bdnf, Cebpd, Cyr61, Fndc3b, Grm5, Hmgb2, Id2, Il6, Jun, Junb, Kitl, Mef2a, Met, Msr1, Pdlim7, Pik3r1, Ptgs2, Rps6ka3, Sh3pxd2b, Socs3, Tal1, Tesc, Tnfrsf12a, Wwtr1 |
| GO:0051347 | positive regulation of transferase activity | 12h, 24h | 27 | Camkk2, Cartpt, Ccl2, Cyr61, Dgkh, Edem1, Epha4, Gadd45b, Gadd45g, Grm1, Grm5, Hspa5, Kitl, Lmna, Lrp8, Map2k3, Map3k6, Met, Pim1, Plce1, Prkar2a, Ptk2b, Ripk1, Sphk1, Thbs1, Tnik, Zeb2 |
| GO:0030334 | regulation of cell migration | 12h, 24h | 27 | Amotl1, Anxa3, C3ar1, Camk1d, Ccl11, Ccl2, Ccl4, Ccl7, Cited2, Cyr61, Hbegf, Hmox1, Icam1, Il6, Lmna, Msn, Pik3r1, Podxl, Prkx, Ptgs2, Ptk2b, Rhoc, Scai, Serpine1, Sphk1, Thbs1, Tlr2 |

| | | | | |
|---|---|---|---|---|
| GO:0051960 | regulation of nervous system development | 12h, 24h | 27 | Akap5, Arhgef2, Bcl11a, Bdnf, Camk1d, Camk2b, Chn1, D130043K22Rik, Epha3, Epha4, Grin1, Grm5, Id2, Il6, Kalrn, Met, Nlgn3, Nrxn1, Ptk2b, Rhoc, Rufy3, Slit1, Sphk1, Spp1, Tnfrsf12a, Tnik, Vim |
| GO:0007243 | intracellular protein kinase cascade | 6h, 12h, 24h | 26 | Bcl3, Camkk2, Cav1, Ccl11, Ccl2, Errfi1, Fos, Hmox1, Jun, Map2k3, Map4k4, Mapk8ip2, Mef2a, Myc, Plek, Ptk2b, Rhoc, Rps6ka3, Rps6ka5, Rps6ka6, Scg2, Sik2, Socs3, Stat3, Tnik, Zfp36 |
| GO:0006163 | purine nucleotide metabolic process | 12h | 26 | Ak5, Ampd3, Arl4d, Atp13a5, Atp2b1, Atp2b2, Diras1, Dnm1, Dnm3, Gbp2, Gem, Gng2, Gucy1b3, Hspa5, Myo9b, Pde1a, Pde4b, Rab27b, Rasl11a, Rhoc, Rhoj, Rnd3, Rora, Rrad, Sult2b1, Trim23 |
| GO:0031644 | regulation of neurological system process | 6h, 12h | 26 | Arc, Atp2b2, Bdnf, Cacna1b, Camk2a, Camk2b, Cartpt, Ccl2, Crh, Egr2, Gria1, Grin1, Grm1, Grm5, Il6, Mapk8ip2, Nat8l, Nlgn3, Npy2r, Npy5r, Nrxn1, Pdyn, Ppp3ca, Ptgs2, Slc24a2, Sphk1 |
| GO:0051046 | regulation of secretion | 12h, 24h | 26 | Abcc8, Akap5, Cacna1b, Cacna1d, Cacna1h, Camk2a, Cartpt, Cd14, Crh, Grm1, Hmox1, Il11, Il6, Kcnc2, Npy2r, Npy5r, Pclo, Pcsk1, Pdyn, Pik3c3, Ppp3ca, Prkar2a, Rab27b, Sphk1, Srgn, Syt7 |
| GO:0031960 | response to corticosteroid stimulus | 6h, 12h, 24h | 26 | Anxa3, Areg, Atp2b1, Cav1, Ccl2, Cdkn1a, Cpn1, Crh, Dusp1, Eng, Fos, Fosb, Fosl1, Gpr83, Hnmt, Igfbp7, Il6, Junb, Pcsk1, Pik3r1, Ptgs2, Rhoc, Serpina3n, Serpine1, Socs3, Sstr2 |
| GO:0030003 | cellular cation homeostasis | 12h, 24h | 25 | Atp2b2, C3ar1, Cav1, Ccl11, Ccl2, Ccl7, Gpr12, Grin1, Grm1, Grm5, Hmox1, Mt2, Myc, Nedd4l, Npy2r, Nr3c2, Oprl1, Plce1, Ptk2b, Ryr2, S1pr3, Slc12a5, Slc24a2, Tesc, Tgm2 |
| GO:0009968 | negative regulation of signal transduction | 12h, 24h | 25 | Cav1, Cd44, Cyld, Cyp7b1, Eng, Errfi1, Fbn1, Grm5, Hspa5, Mcl1, Myc, Ncor1, Nfkbia, Plek, Rgs1, Rgs2, Rgs4, Rhoc, Ripk1, Rps6ka6, Scai, Siah2, Socs3, Thbs1, Wwtr1 |
| GO:0071345 | cellular response to cytokine stimulus | 12h | 24 | Camk2a, Camk2b, Ccl2, Cd44, Cyba, Eif4e2, Gbp2, Icam1, Il13ra1, Il17ra, Il1rap, Il6, Klf6, Lrp8, Met, Osmr, Ptk2b, Ripk1, Rps6ka5, Serpina3n, Socs3, Stat3, Thbs1, Tnfrsf1a |

| | | | | |
|---|---|---|---|---|
| GO:0050804 | regulation of synaptic transmission | 6h, 12h | 24 | Arc, Atp2b2, Bdnf, Cacna1b, Camk2a, Camk2b, Ccl2, Crh, Egr2, Gria1, Grin1, Grm1, Grm5, Mapk8ip2, Nat8l, Nlgn3, Npy2r, Npy5r, Nrxn1, Pdyn, Ppp3ca, Ptgs2, Slc24a2, Sphk1 |
| GO:0015672 | monovalent inorganic cation transport | 12h, 24h | 23 | Abcc8, Kcna2, Kcnab1, Kcnc1, Kcnc2, Kcnf1, Kcnh3, Kcnip2, Kcnip4, Kcnj3, Kcnj9, Kcnq5, Kctd4, Nalcn, Nedd4l, Scn1a, Scn2a1, Scn3b, Slc10a6, Slc12a5, Slc24a2, Slc5a3, Slc6a8 |
| GO:0031347 | regulation of defense response | 12h, 24h | 23 | Crh, Cyld, Fos, Ier3, Il6, Jun, Map2k3, Mef2a, Nfkbia, Npy5r, Osmr, Pglyrp1, Ptgs2, Pvr, Ripk1, Rps6ka3, Rps6ka5, Serpine1, Socs3, Tgm2, Tlr2, Tnfrsf1a, Zfp36 |
| GO:0019932 | second-messenger-mediated signaling | 6h, 12h, 24h | 23 | Adcyap1r1, Cacna1d, Camkk2, Ccl2, Crem, Grm1, Grm5, Gucy1b3, Homer1, Htr1a, Mt2, Npy, Npy2r, Oprl1, Pclo, Plce1, Rcan1, Rgs1, Ryr2, S1pr3, Sphk1, Sstr2, Tgm2 |
| GO:0034765 | regulation of ion transmembrane transport | 6h, 12h, 24h | 22 | Cacna1b, Cacna1d, Cacna1h, Crh, Cybb, Homer1, Kcna2, Kcnab1, Kcnc1, Kcnc2, Kcnf1, Kcnh3, Kcnip2, Kcnip4, Kcnj3, Kcnj9, Kcnq5, Nalcn, Rhoc, Scn1a, Scn2a1, Scn3b |
| GO:0051056 | regulation of small GTPase mediated signal transduction | 6h, 12h | 22 | Arhgap12, Arhgap15, Arhgap20, Arhgap39, Arhgef2, Arhgef25, Arhgef4, Arpp19, Chn1, Iqgap2, Iqsec2, Kalrn, Kitl, Mcf2l, Myo9b, Plce1, Rasgef1b, Rasgrf2, Rasgrp1, Rhoc, Rhoj, Scai |
| GO:0045596 | negative regulation of cell differentiation | 12h, 24h | 21 | Areg, Arhgef2, Bcl11a, Bdnf, Cartpt, Cav1, Epha4, Id2, Il6, Lilrb4, Myc, Nfkbia, Pik3r1, Ptk2b, Rcan1, Rhoc, Rufy3, Slit1, Spp1, Wwtr1, Zfp36 |
| GO:2000147 | positive regulation of cell motility | 12h, 24h | 21 | Amotl1, Anxa3, C3ar1, Camk1d, Ccl11, Ccl2, Ccl4, Ccl7, Cyr61, Hbegf, Icam1, Il6, Pik3r1, Podxl, Ptgs2, Ptk2b, Rhoc, Serpine1, Sphk1, Thbs1, Tlr2 |
| GO:0051272 | positive regulation of cellular component movement | 12h, 24h | 21 | Amotl1, Anxa3, C3ar1, Camk1d, Ccl11, Ccl2, Ccl4, Ccl7, Cyr61, Hbegf, Icam1, Il6, Pik3r1, Podxl, Ptgs2, Ptk2b, Rhoc, Serpine1, Sphk1, Thbs1, Tlr2 |
| GO:0051346 | negative regulation of hydrolase activity | 12h, 24h | 20 | Bcl2a1d, Cd44, Cflar, Cnn3, Cstb, Ctla2b, Elfn2, Hspa1b, Hspa5, Il6, Nos3, Plek, Pros1, Rgs2, Rps6ka3, Serpina3n, Serpine1, Siah2, Tfpi2, Thbs1 |

| | | | | |
|---|---|---|---|---|
| GO:0009166 | nucleotide catabolic process | 12h | 20 | Ampd3, Arl4d, Atp2b2, Diras1, Dnm1, Dnm3, Gbp2, Gem, Gng2, Hspa5, Myo9b, Pde1a, Pde4b, Rab27b, Rasl11a, Rhoc, Rhoj, Rnd3, Rrad, Trim23 |
| GO:0031349 | positive regulation of defense response | 12h, 24h | 20 | Crh, Cyld, Fos, Il6, Jun, Map2k3, Mef2a, Nfkbia, Npy5r, Osmr, Pglyrp1, Ptgs2, Pvr, Ripk1, Rps6ka3, Rps6ka5, Serpine1, Tgm2, Tlr2, Tnfrsf1a |
| GO:0072523 | purine-containing compound catabolic process | 12h | 20 | Ampd3, Arl4d, Atp2b2, Diras1, Dnm1, Dnm3, Gbp2, Gem, Gng2, Hspa5, Myo9b, Pde1a, Pde4b, Rab27b, Rasl11a, Rhoc, Rhoj, Rnd3, Rrad, Trim23 |
| GO:0031329 | regulation of cellular catabolic process | 12h | 20 | Arhgap15, Ccl11, Chn1, Cnn3, D630037F22Rik, Dlgap1, Epha3, Epha4, Errfi1, Ier3, Jun, Myo9b, Ncor1, Ptk2b, Rabgap1l, Rgs1, Rgs2, Rgs4, Timp1, Zfp36 |
| GO:0010975 | regulation of neuron projection development | 12h, 24h | 20 | Akap5, Bcl11a, Camk1d, Camk2b, Chn1, D130043K22Rik, Epha3, Epha4, Grin1, Kalrn, Met, Ptk2b, Rhoc, Rufy3, Slit1, Sphk1, Spp1, Tnfrsf12a, Tnik, Vim |
| GO:0052547 | regulation of peptidase activity | 12h, 24h | 20 | Bcl2a1d, Cav1, Cd44, Cflar, Cstb, Ctla2b, Cyr61, Hspa1b, Hspa5, Il6, Myc, Pros1, Rhoc, Ripk1, Rps6ka3, Serpina3n, Serpine1, Siah2, Tfpi2, Thbs1 |
| GO:0043627 | response to estrogen stimulus | 12h | 20 | Adcyap1r1, Areg, Arsb, Cav1, Cited2, Crh, Dusp1, Fosl2, Gria1, Hmox1, Lct, Nos3, Pik3r1, Ptgs2, Rcan1, Serpine1, Socs3, Sstr2, Stat3, Tnfrsf1a |
| GO:0019221 | cytokine-mediated signaling pathway | 12h, 24h | 19 | Camk2a, Camk2b, Ccl2, Cd44, Eif4e2, Gbp2, Icam1, Il13ra1, Il1rap, Il6, Klf6, Lrp8, Osmr, Ptk2b, Ripk1, Rps6ka5, Socs3, Stat3, Tnfrsf1a |
| GO:0032103 | positive regulation of response to external stimulus | 12h, 24h | 18 | C3ar1, Camk1d, Ccl2, Ccl4, Ccl7, Il16, Il6, Npy, Npy5r, Osmr, Ptgs2, Ptk2b, Scg2, Serpine1, Tgm2, Thbs1, Tlr2, Tnfrsf1a |
| GO:0045765 | regulation of angiogenesis | 12h, 24h | 18 | Adamts1, Anxa3, Bai3, C3ar1, Ccl11, Ccl2, Col4a2, Cyr61, Eng, Hmox1, Il6, Nos3, Ptgs2, Rnh1, Serpine1, Sphk1, Thbs1, Tnfrsf1a |
| GO:0070201 | regulation of establishment of protein localization | 12h, 24h | 18 | Akap5, Ar, Bcl3, Cd14, Cdkn1a, Cyld, Il6, Nfkbia, Nrxn1, Pcsk1, Pik3c3, Pik3r1, Ptgs2, Sphk1, Srgn, Tlr2, Tnfrsf1a, Wwtr1 |
| GO:0050778 | positive regulation of immune response | 12h, 24h | 17 | Bcl2a1d, C3ar1, Cd44, Cyld, Fos, Hspa1b, Jun, Map2k3, Mef2a, Nfkbia, Pglyrp1, Pik3r1, Pvr, Ripk1, Rps6ka3, Rps6ka5, Tlr2 |

| | | | | |
|---|---|---|---|---|
| GO:0043405 | regulation of MAP kinase activity | 12h | 16 | Cav1, Dusp1, Epha4, Grm1, Kitl, Map2k3, Map3k6, Met, Pdcd4, Plce1, Ptk2b, Rgs2, Rgs4, Ripk1, Thbs1, Zeb2 |
| GO:0042391 | regulation of membrane potential | 12h, 24h | 16 | Bdnf, Cacna1b, Cav1, Gria1, Grin1, Grm1, Jun, Kcnc2, Mapk8ip2, Nlgn3, Npy2r, Nrxn1, Ppp3ca, Scn1a, Scn2a1, Scn3b |
| GO:0051592 | response to calcium ion | 6h, 12h, 24h | 16 | Capn3, Cav1, Dusp1, Fos, Fosb, Grin1, Homer1, Il6, Jun, Junb, Kcnip2, Mef2a, Pcsk1, Ptk2b, Ryr2, Thbs1 |
| GO:0072511 | divalent inorganic cation transport | 12h | 15 | Atp2b1, Atp2b2, Cacna1b, Cacna1d, Cacna1h, Camk2a, Camk2b, Cav1, Grin1, Nfatc1, Npy, Ppp3ca, Ryr2, Slc24a2, Slc39a10 |
| GO:0050678 | regulation of epithelial cell proliferation | 6h, 12h | 15 | Ar, Cav1, Ccl11, Ccl2, Cyba, Cyp7b1, Eaf2, Eng, Hmgb2, Il6, Jun, Myc, Scg2, Thbs1, Tinf2 |
| GO:0051223 | regulation of protein transport | 12h | 15 | Akap5, Bcl3, Cd14, Cdkn1a, Cyld, Il6, Nfkbia, Pcsk1, Pik3c3, Ptgs2, Sphk1, Srgn, Tlr2, Tnfrsf1a, Wwtr1 |
| GO:0071363 | cellular response to growth factor stimulus | 12h | 14 | Ccl2, Cited2, Col4a2, Gria1, Hspb1, Lrp8, Met, Nos3, Pde1a, Pik3r1, Prkar2a, Ptk2b, Sphk1, Thbs1 |
| GO:0048520 | positive regulation of behavior | 12h, 24h | 14 | C3ar1, Camk1d, Ccl2, Ccl4, Ccl7, Crh, Il16, Il6, Npy2r, Nr2c2, Ptk2b, Scg2, Serpine1, Thbs1 |
| GO:0010769 | regulation of cell morphogenesis involved in differentiation | 12h, 24h | 14 | Akap5, Bcl11a, Camk2b, Chn1, Epha3, Epha4, Grin1, Met, Rufy3, Slit1, Spp1, Tnfrsf12a, Tnik, Wwtr1 |
| GO:0043087 | regulation of GTPase activity | 12h, 24h | 14 | Arhgap15, Ccl11, Chn1, D630037F22Rik, Epha3, Epha4, Errfi1, Jun, Myo9b, Ptk2b, Rabgap1l, Rgs1, Rgs2, Rgs4 |
| GO:0001819 | positive regulation of cytokine production | 12h, 24h | 13 | Bcl3, C3ar1, Ccl4, Cd14, Hspb1, Il17ra, Il6, Ptgs2, Ripk1, Serpine1, Thbs1, Tlr2, Tnfrsf1a |
| GO:0010638 | positive regulation of organelle organization | 12h, 24h | 13 | Anxa2, Cav1, Ccl11, Jarid2, Met, Ncor1, Plek, Ptk2b, Rhoc, Rps6ka5, Synpo, Tal1, Tinf2 |
| GO:0051493 | regulation of cytoskeleton organization | 12h, 24h | 13 | Arhgef2, Arpc1b, Capg, Cav1, Ccl11, Cyld, Epha3, Hsph1, Plek, Ptk2b, Rhoc, Shroom2, Synpo |
| GO:0051052 | regulation of DNA metabolic process | 12h | 13 | Anxa3, Areg, Bmyc, Cdkn1a, Hmgb2, Il6, Jun, Kitl, Met, Myc, Prkcc, S100a11, Tinf2 |

| | | | | |
|---|---|---|---|---|
| GO:0045088 | regulation of innate immune response | 12h, 24h | 13 | Cyld, Fos, Jun, Map2k3, Mef2a, Nfkbia, Pglyrp1, Pvr, Ripk1, Rps6ka3, Rps6ka5, Socs3, Tlr2 |
| GO:0048660 | regulation of smooth muscle cell proliferation | 6h, 12h, 24h | 13 | Atp2b1, Cyba, Hbegf, Hmox1, Id2, Il6, Jun, Nos3, Npy5r, Pde1a, Pik3r1, Ptgs2, Tgm2 |
| GO:0071383 | cellular response to steroid hormone stimulus | 6h, 12h | 12 | Ar, Atp2b1, Crh, Nr1d2, Nr2c2, Nr3c2, Nr4a2, Rora, Rorb, Serpina3n, Sstr2, Tnfrsf1a |
| GO:0051348 | negative regulation of transferase activity | 6h, 12h, 24h | 12 | Cav1, Cdkn1a, Dusp1, Errfi1, Gadd45b, Gadd45g, Hspb1, Il6, Pdcd4, Rgs2, Rgs4, Wwtr1 |
| GO:0045766 | positive regulation of angiogenesis | 12h, 24h | 12 | Anxa3, C3ar1, Ccl11, Cyr61, Eng, Hmox1, Nos3, Ptgs2, Serpine1, Sphk1, Thbs1, Tnfrsf1a |
| GO:0043270 | positive regulation of ion transport | 12h, 24h | 12 | Abcc8, Adcyap1r1, Akap5, Akt3, Atp2b2, Capn3, Cav1, Ccl4, Cntn1, Crh, Homer1, Scn3b |
| GO:0032386 | regulation of intracellular transport | 12h, 24h | 12 | Akap5, Bcl3, Cdkn1a, Cyld, Il6, Nfkbia, Ptgs2, Ryr2, Sphk1, Tlr2, Tnfrsf1a, Wwtr1 |
| GO:0090257 | regulation of muscle system process | 12h, 24h | 12 | Camk2b, Cav1, Mef2a, Nos3, Npy2r, Plce1, Ptgs2, Rgs2, Ryr2, Scn3b, Sphk1, Sstr2 |
| GO:0032570 | response to progesterone stimulus | 6h, 12h, 24h | 12 | Cav1, Ccl2, Fos, Fosb, Fosl1, Fosl2, Junb, Pik3r1, Socs3, Sphk1, Thbs1, Tlr2 |
| GO:0030308 | negative regulation of cell growth | 12h, 24h | 11 | Bcl11a, Cdkn1a, Eaf2, Foxk1, Hspa1a, Hspa1b, Ndrg3, Rrad, Sertad1, Slit1, Spp1 |
| GO:0002221 | pattern recognition receptor signaling pathway | 12h, 24h | 11 | Cyld, Fos, Jun, Map2k3, Mef2a, Nfkbia, Pglyrp1, Ripk1, Rps6ka3, Rps6ka5, Tlr2 |
| GO:0045785 | positive regulation of cell adhesion | 6h, 12h, 24h | 11 | Cited2, Cyr61, Kifap3, Npy2r, Podxl, Ptk2b, Rhoc, Smoc2, Spp1, Tgm2, Thbs1 |
| GO:0050921 | positive regulation of chemotaxis | 12h, 24h | 11 | C3ar1, Camk1d, Ccl2, Ccl4, Ccl7, Il16, Il6, Ptk2b, Scg2, Serpine1, Thbs1 |
| GO:0002687 | positive regulation of leukocyte migration | 12h, 24h | 11 | C3ar1, Camk1d, Ccl2, Ccl4, Ccl7, Icam1, Il6, Ptk2b, Serpine1, Thbs1, Tlr2 |

| | | | | |
|---|---|---|---|---|
| GO:0051092 | positive regulation of NF-kappaB transcription factor activity | 12h, 24h | 11 | Ar, Arhgef2, Camk2a, Capn3, Icam1, Il6, Nfkbia, Ripk1, Rps6ka5, Sphk1, Tlr2 |
| GO:0060191 | regulation of lipase activity | 12h, 24h | 11 | Adcyap1r1, Cyr61, Grm1, Grm5, Homer1, Pde1a, Plce1, Plek, Prkar2a, Rgs2, Tgm2 |
| GO:0090087 | regulation of peptide transport | 12h, 24h | 11 | Abcc8, Akap5, Cacna1d, Cartpt, Crh, Kcnc2, Npy2r, Pcsk1, Ppp3ca, Prkar2a, Syt7 |
| GO:0042306 | regulation of protein import into nucleus | 12h | 11 | Akap5, Bcl3, Cdkn1a, Cyld, Il6, Nfkbia, Ptgs2, Sphk1, Tlr2, Tnfrsf1a, Wwtr1 |
| GO:0070302 | regulation of stress-activated protein kinase signaling cascade | 12h | 11 | Epha4, Ltbr, Map3k6, Map4k4, Mapk8ip2, Myc, Ncor1, Pdcd4, Ptk2b, Ripk1, Zeb2 |
| GO:0034284 | response to monosaccharide stimulus | 12h, 24h | 11 | Cyba, Icam1, Mef2a, Met, Pcsk1, Pik3r1, Ppp3ca, Ptgs2, Ptk2b, Rhoc, Thbs1 |
| GO:0071248 | cellular response to metal ion | 6h, 12h, 24h | 10 | Cacna1h, Capn3, Fos, Fosb, Grin1, Hmox1, Id2, Jun, Junb, Mef2a |
| GO:0032102 | negative regulation of response to external stimulus | 12h, 24h | 10 | Cartpt, Ccl2, Epha4, Grin1, Ier3, Nrxn1, Serpine1, Slit1, Spp1, Zfp36 |
| GO:0051048 | negative regulation of secretion | 12h, 24h | 10 | Abcc8, Cartpt, Crh, Hmox1, Il11, Il6, Npy2r, Npy5r, Ppp3ca, Srgn |
| GO:0050727 | regulation of inflammatory response | 12h, 24h | 10 | Ier3, Il6, Npy5r, Osmr, Ptgs2, Serpine1, Tgm2, Tlr2, Tnfrsf1a, Zfp36 |
| GO:0090276 | regulation of peptide hormone secretion | 12h, 24h | 10 | Abcc8, Akap5, Cacna1d, Cartpt, Crh, Kcnc2, Pcsk1, Ppp3ca, Prkar2a, Syt7 |
| GO:0008063 | Toll signaling pathway | 12h, 24h | 10 | Cd14, Fos, Jun, Map2k3, Mef2a, Nfkbia, Ripk1, Rps6ka3, Rps6ka5, Tlr2 |
| GO:0071222 | cellular response to lipopolysaccharide | 12h, 24h | 9 | Ccl2, Cd14, Gbp2, Hmgb2, Icam1, Nfkbia, Nos3, Serpine1, Tlr2 |
| GO:0030198 | extracellular matrix organization | 12h, 24h | 9 | Anxa2, Bcl3, Col4a2, Cyr61, Eng, Lgals3, Sh3pxd2b, Smoc2, Tgfbi |

| | | | | |
|---|---|---|---|---|
| GO:0031346 | positive regulation of cell projection organization | 12h | 9 | Akap5, Bcl11a, Camk1d, Camk2b, Epha3, Met, Ptk2b, Sphk1, Tnfrsf12a |
| GO:0031646 | positive regulation of neurological system process | 12h | 9 | Camk2b, Cartpt, Ccl2, Crh, Gria1, Il6, Nrxn1, Ptgs2, Slc24a2 |
| GO:0051971 | positive regulation of transmission of nerve impulse | 12h | 9 | Camk2b, Cartpt, Ccl2, Crh, Gria1, Il6, Nrxn1, Ptgs2, Slc24a2 |
| GO:0008016 | regulation of heart contraction | 6h, 12h, 24h | 9 | Cacna1b, Cacna1h, Hbegf, Kcnip2, Mef2a, Pik3r1, Rgs2, Ryr2, Scn3b |
| GO:0002688 | regulation of leukocyte chemotaxis | 12h, 24h | 9 | C3ar1, Camk1d, Ccl2, Ccl4, Ccl7, Il6, Ptk2b, Serpine1, Thbs1 |
| GO:0032680 | regulation of tumor necrosis factor production | 12h, 24h | 9 | Bcl3, Ccl4, Cd14, Hspb1, Ripk1, Thbs1, Tlr2, Tnfrsf1a, Zfp36 |
| GO:0061041 | regulation of wound healing | 6h, 12h, 24h | 9 | Anxa2, Capn3, Cav1, Hbegf, Nfe2l2, Nos3, Plek, Serpine1, Thbs1 |
| GO:0033500 | carbohydrate homeostasis | 12h | 8 | Cartpt, Cyba, Icam1, Mef2a, Met, Ppp3ca, Serpine1, Stat3 |
| GO:0021953 | central nervous system neuron differentiatio n | 12h | 8 | Abt1, Atp2b2, Bcl11b, Faim2, Met, Nr4a2, Rora, Tal1 |
| GO:0007215 | glutamate receptor signaling pathway | 12h, 24h | 8 | Camk2a, Gria1, Gria3, Grin1, Grm1, Grm5, Homer1, Homer2 |
| GO:0045787 | positive regulation of cell cycle | 12h | 8 | Cited2, Eif4ebp1, Fosl1, Id2, Met, Pim1, Sphk1, Tal1 |
| GO:0030307 | positive regulation of cell growth | 12h | 8 | Bcl11a, Cyba, Hbegf, Ptk2b, Rhoc, Rps6ka3, Sphk1, Tnfrsf12a |
| GO:0010810 | regulation of cell-substrate adhesion | 12h, 24h | 8 | Cyr61, Epha3, Pik3r1, Ptk2b, Serpine1, Smoc2, Spp1, Thbs1 |
| GO:0033044 | regulation of chromosome organization | 12h | 8 | Bmyc, Jarid2, Myc, Ncor1, Rps6ka5, Tal1, Tinf2, Tlk2 |

| | | | | |
|---|---|---|---|---|
| GO:0042035 | regulation of cytokine biosynthetic process | 12h, 24h | 8 | Bcl3, Hmox1, Hspb1, Igf2bp2, Il6, Map2k3, Thbs1, Tlr2 |
| GO:0002761 | regulation of myeloid leukocyte differentiation | 12h | 8 | Cartpt, Id2, Jun, Kitl, Lilrb4, Myc, Pik3r1, Tal1 |
| GO:0042542 | response to hydrogen peroxide | 12h | 8 | Areg, Dusp1, Fosl1, Hmox1, Il6, Jun, Ptk2b, Sphk1 |
| GO:0034341 | response to interferon-gamma | 12h | 8 | Camk2a, Camk2b, Ccl2, Cd44, Gbp2, Icam1, Serpina3n, Socs3 |
| GO:0070555 | response to interleukin-1 | 12h | 8 | Ccl2, Cyba, Hnmt, Icam1, Pcsk1, Rps6ka5, Serpina3n, Sphk1 |
| GO:0031345 | negative regulation of cell projection organization | 12h, 24h | 7 | Bcl11a, D130043K22Rik, Epha4, Rufy3, Slit1, Spp1, Vim |
| GO:0043271 | negative regulation of ion transport | 12h, 24h | 7 | Akap5, Icam1, Nedd4l, Nos3, Ptgs2, Ptk2b, Rhoc |
| GO:0045638 | negative regulation of myeloid cell differentiation | 12h | 7 | Cartpt, Lilrb4, Myc, Nfkbia, Pik3r1, Ptk2b, Zfp36 |
| GO:0008064 | regulation of actin polymerization or depolymerization | 12h, 24h | 7 | Arpc1b, Capg, Ccl11, Plek, Ptk2b, Rhoc, Shroom2 |
| GO:0045761 | regulation of adenylate cyclase activity | 12h | 7 | Adcyap1r1, Htr1a, Npy2r, Oprl1, Rgs1, S1pr3, Sstr2 |
| GO:2001233 | regulation of apoptotic signaling pathway | 12h | 7 | Cav1, Cyld, Ltbr, Mcl1, Ripk1, Siah2, Tnfrsf12a |
| GO:0030193 | regulation of blood coagulation | 12h, 24h | 7 | Anxa2, Cav1, Nfe2l2, Nos3, Plek, Serpine1, Thbs1 |
| GO:0006109 | regulation of carbohydrate metabolic process | 12h | 7 | Adcyap1r1, Arpp19, Ier3, Il6, Ncor1, Plek, Ptk2b |
| GO:0010675 | regulation of cellular carbohydrate metabolic process | 12h | 7 | Adcyap1r1, Arpp19, Ier3, Il6, Ncor1, Plek, Ptk2b |

| | | | | |
|---|---|---|---|---|
| GO:0045667 | regulation of osteoblast differentiation | 12h | 7 | Areg, Cebpd, Cyr61, Id2, Il6, Nell1, Pdlim7 |
| GO:0034612 | response to tumor necrosis factor | 12h | 7 | Ccl2, Cyba, Icam1, Ptk2b, Ripk1, Thbs1, Tnfrsf1a |
| GO:0043401 | steroid hormone mediated signaling pathway | 6h, 12h | 7 | Ar, Nr1d2, Nr2c2, Nr3c2, Nr4a2, Rora, Rorb |
| GO:0051403 | stress-activated MAPK cascade | 6h, 12h, 24h | 7 | Fos, Jun, Map2k3, Mapk8ip2, Mef2a, Rps6ka3, Rps6ka5 |
| GO:0008306 | associative learning | 12h | 6 | Crh, Fos, Fosl2, Gabra5, Grin1, Nlgn3 |
| GO:0030183 | B cell differentiation | 12h | 6 | Bcl11a, Bcl3, Il11, Klf6, Pik3r1, Ptk2b |
| GO:0008584 | male gonad development | 12h | 6 | Ar, Cited2, Hmgb2, Lhx9, Met, Tesc |
| GO:0016525 | negative regulation of angiogenesis | 12h, 24h | 6 | Adamts1, Bai3, Ccl2, Col4a2, Serpine1, Thbs1 |
| GO:0031280 | negative regulation of cyclase activity | 12h | 6 | Htr1a, Npy2r, Oprl1, Rgs1, S1pr3, Sstr2 |
| GO:0046888 | negative regulation of hormone secretion | 12h, 24h | 6 | Abcc8, Cartpt, Crh, Il11, Il6, Ppp3ca |
| GO:0051350 | negative regulation of lyase activity | 12h | 6 | Htr1a, Npy2r, Oprl1, Rgs1, S1pr3, Sstr2 |
| GO:0045807 | positive regulation of endocytosis | 12h, 24h | 6 | Caly, Camk1d, Cav1, Nedd4l, Pros1, Serpine1 |
| GO:0032388 | positive regulation of intracellular transport | 12h | 6 | Akap5, Il6, Ptgs2, Sphk1, Tlr2, Tnfrsf1a |
| GO:0045639 | positive regulation of myeloid cell differentiation | 12h | 6 | Hmgb2, Id2, Jun, Kitl, Tal1, Tesc |
| GO:0031334 | positive regulation of protein complex assembly | 12h | 6 | Cav1, Ccl11, Plek, Ptk2b, Rhoc, Tal1 |

| | | | | |
|---|---|---|---|---|
| GO:0034764 | positive regulation of transmembrane transport | 12h | 6 | Akap5, Crh, Il6, Ptgs2, Sphk1, Tlr2 |
| GO:0016485 | protein processing | 12h | 6 | Cuzd1, Myc, Pcsk1, Pcsk2, Pik3c3, Srgn |
| GO:0022407 | regulation of cell-cell adhesion | 12h, 24h | 6 | Cited2, Fermt3, Fxyd5, Kifap3, Pik3r1, Podxl |
| GO:0045598 | regulation of fat cell differentiation | 12h | 6 | Fndc3b, Id2, Il6, Ptgs2, Sh3pxd2b, Wwtr1 |
| GO:0048145 | regulation of fibroblast proliferation | 6h, 12h, 24h | 6 | Cdkn1a, Fosl2, Jun, Myc, Sphk1, Zmiz1 |
| GO:2000377 | regulation of reactive oxygen species metabolic process | 12h, 24h | 6 | Cdkn1a, Ier3, Nfe2l2, Ptk2b, Ripk1, Thbs1 |
| GO:0071322 | cellular response to carbohydrate stimulus | 12h | 5 | Cyba, Icam1, Mef2a, Met, Ppp3ca |
| GO:0071333 | cellular response to glucose stimulus | 12h | 5 | Cyba, Icam1, Mef2a, Met, Ppp3ca |
| GO:0008045 | motor axon guidance | 12h, 24h | 5 | Chn1, Egr2, Epha4, Lhx9, Slit1 |
| GO:0043392 | negative regulation of DNA binding | 6h, 12h | 5 | Hmox1, Id2, Jun, Nfkbia, Zfp462 |
| GO:2001235 | positive regulation of apoptotic signaling pathway | 12h | 5 | Cav1, Cyld, Ltbr, Ripk1, Tnfrsf12a |
| GO:0043255 | regulation of carbohydrate biosynthetic process | 12h | 5 | Adcyap1r1, Arpp19, Il6, Plek, Ptk2b |
| GO:2000401 | regulation of lymphocyte migration | 12h | 5 | Ccl2, Ccl4, Ccl7, Msn, Ptk2b |
| GO:0051588 | regulation of neurotransmitter transport | 12h | 5 | Cacna1b, Camk2a, Nat8l, Pdyn, Sphk1 |
| GO:0045428 | regulation of nitric oxide biosynthetic process | 12h, 24h | 5 | Cav1, Icam1, Il6, Ptgs2, Tlr2 |

| | | | | |
|---|---|---|---|---|
| GO:0032069 | regulation of nuclease activity | 12h | 5 | Ccl2, Edem1, Hmgb2, Hspa5, Lmna |
| GO:0019229 | regulation of vasoconstriction | 12h | 5 | Cav1, Hspa1b, Icam1, Ptgs2, Rhoc |
| GO:0042220 | response to cocaine | 12h | 5 | Crh, Gria1, Hnmt, Homer1, Ptk2b |
| GO:0010332 | response to gamma radiation | 12h | 5 | Cav1, Ccl2, Cyba, Myc, Socs3 |
| GO:0014072 | response to isoquinoline alkaloid | 6h, 12h | 5 | Fosb, Grin1, Pcsk1, Prkcc, Srr |
| GO:0014073 | response to tropane | 12h | 5 | Crh, Gria1, Hnmt, Homer1, Ptk2b |
| GO:0046631 | alpha-beta T cell activation | 12h, 24h | 4 | Bcl11b, Bcl3, Gadd45g, Ncor1 |
| GO:0031663 | lipopolysaccharide-mediated signaling pathway | 12h | 4 | Ccl2, Nfkbia, Nos3, Tlr2 |
| GO:0007616 | long-term memory | 12h | 4 | Crh, Gria1, Grin1, Pcdh8 |
| GO:0050819 | negative regulation of coagulation | 12h, 24h | 4 | Anxa2, Nos3, Pros1, Serpine1 |
| GO:0031645 | negative regulation of neurological system process | 6h, 12h | 4 | Npy2r, Npy5r, Ptgs2, Slc24a2 |
| GO:0051970 | negative regulation of transmission of nerve impulse | 6h, 12h | 4 | Npy2r, Npy5r, Ptgs2, Slc24a2 |
| GO:0010524 | positive regulation of calcium ion transport into cytosol | 12h | 4 | Adcyap1r1, Akap5, Capn3, Cav1 |
| GO:0045933 | positive regulation of muscle contraction | 6h, 12h | 4 | Npy2r, Ptgs2, Rgs2, Sphk1 |
| GO:0045907 | positive regulation of vasoconstriction | 6h, 12h | 4 | Cav1, Icam1, Ptgs2, Rhoc |
| GO:0033628 | regulation of cell adhesion mediated by integrin | 12h | 4 | Fermt3, Podxl, Serpine1, Tesc |

| | | | | |
|---|---|---|---|---|
| GO:0045187 | regulation of circadian sleep/wake cycle, sleep | 12h | 4 | Crh, Il6, Npy2r, Pglyrp1 |
| GO:0032677 | regulation of interleukin-8 production | 12h, 24h | 4 | Bcl3, Ripk1, Serpine1, Tlr2 |
| GO:0048169 | regulation of long-term neuronal synaptic plasticity | 12h | 4 | Bdnf, Camk2b, Grin1, Grm5 |
| GO:0046928 | regulation of neurotransmitter secretion | 12h | 4 | Cacna1b, Camk2a, Pdyn, Sphk1 |
| GO:0010574 | regulation of vascular endothelial growth factor production | 12h | 4 | C3ar1, Ccl2, Il6, Ptgs2 |
| GO:0001975 | response to amphetamine | 6h, 12h | 4 | Grin1, Icam1, Nr4a2, Ppp3ca |
| GO:0070542 | response to fatty acid | 12h | 4 | Fabp3, Pcsk1, Pik3r1, Tlr2 |
| GO:0070741 | response to interleukin-6 | 12h | 4 | Il6, Met, Serpina3n, Stat3 |
| GO:0010226 | response to lithium ion | 12h, 24h | 4 | Gria1, Gria3, Id2, Ptk2b |
| GO:0033574 | response to testosterone stimulus | 12h | 4 | Cacna1b, Dusp1, Pik3r1, Thbs1 |
| GO:0055002 | striated muscle cell development | 6h, 12h | 4 | AI464131, Homer1, Ppp3ca, Rcan1 |
| GO:0000038 | very long chain fatty acid metabolic process | 12h | 4 | Acot3, Elovl1, Ptgs2, Sphk1 |
| GO:0021702 | cerebellar Purkinje cell differentiation | 12h | 3 | Atp2b2, Faim2, Rora |
| GO:0031497 | chromatin assembly | 12h | 3 | Cenpw, Hist1h1c, Hmga1 |
| GO:0002544 | chronic inflammatory response | 12h, 24h | 3 | Bdnf, Ccl11, Thbs1 |
| GO:2001234 | negative regulation of apoptotic signaling pathway | 12h | 3 | Mcl1, Ripk1, Siah2 |

| | | | | |
|---|---|---|---|---|
| GO:0048521 | negative regulation of behavior | 12h | 3 | Ccl2, Crh, Slit1 |
| GO:0045912 | negative regulation of carbohydrate metabolic process | 12h | 3 | Ier3, Il6, Plek |
| GO:0048640 | negative regulation of development al growth | 12h | 3 | Bcl11a, Slit1, Spp1 |
| GO:0051954 | positive regulation of amine transport | 12h | 3 | Cartpt, Nat8l, Npy2r |
| GO:0050820 | positive regulation of coagulation | 12h, 24h | 3 | Nfe2l2, Serpine1, Thbs1 |
| GO:0032793 | positive regulation of CREB transcription factor activity | 12h | 3 | Camk1d, Crtc1, Rps6ka5 |
| GO:0045648 | positive regulation of erythrocyte differentiatio n | 12h | 3 | Hmgb2, Id2, Tal1 |
| GO:1900048 | positive regulation of hemostasis | 12h, 24h | 3 | Nfe2l2, Serpine1, Thbs1 |
| GO:0051590 | positive regulation of neurotransmi tter transport | 12h | 3 | Cacna1b, Nat8l, Sphk1 |
| GO:0006471 | protein ADP-ribosylation | 12h | 3 | Art3, Tiparp, Trim23 |
| GO:0071675 | regulation of mononuclear cell migration | 12h, 24h | 3 | C3ar1, Ccl2, Thbs1 |
| GO:0051279 | regulation of release of sequestered calcium ion into cytosol | 12h | 3 | Capn3, Ptk2b, Ryr2 |
| GO:0032026 | response to magnesium ion | 12h | 3 | Ryr2, Sphk1, Thbs1 |
| GO:0014805 | smooth muscle adaptation | 12h | 3 | Cyba, Hmox1, Nos3 |

| | | | | |
|---|---|---|---|---|
| GO:0002286 | T cell activation involved in immune response | 12h, 24h | 3 | Bcl3, Gadd45g, Icam1 |
| GO:0003214 | cardiac left ventricle morphogenesis | 12h | 2 | Npy2r, Npy5r |
| GO:0071223 | cellular response to lipoteichoic acid | 12h | 2 | Cd14, Tlr2 |
| GO:0060600 | dichotomous subdivision of an epithelial terminal unit | 12h | 2 | Areg, Plxna1 |
| GO:0043583 | ear development | 12h | 2 | Rdh10, Shroom2 |
| GO:0031076 | embryonic camera-type eye development | 12h | 2 | Aldh1a3, Rdh10 |
| GO:0022617 | extracellular matrix disassembly | 12h | 2 | Eng, Sh3pxd2b |
| GO:0006704 | glucocorticoid biosynthetic process | 12h | 2 | Cacna1h, Crh |
| GO:0030219 | megakaryocyte differentiation | 12h | 2 | Il11, Tal1 |
| GO:0032528 | microvillus organization | 12h | 2 | Fxyd5, Klf5 |
| GO:2000171 | negative regulation of dendrite development | 12h | 2 | Bcl11a, D130043K22Rik |
| GO:0090084 | negative regulation of inclusion body assembly | 12h | 2 | Hspa1a, Hspa1b |
| GO:0070571 | negative regulation of neuron projection regeneration | 12h, 24h | 2 | Epha4, Spp1 |
| GO:0090343 | positive regulation of cell aging | 12h | 2 | Hmga1, Lmna |

| | | | | |
|---|---|---|---|---|
| GO:0048087 | positive regulation of developmental pigmentation | 12h | 2 | Adamts9, Kitl |
| GO:0040019 | positive regulation of embryonic development | 12h | 2 | Hspa5, Nr2c2 |
| GO:0090004 | positive regulation of establishment of protein localization in plasma membrane | 12h | 2 | Nrxn1, Pik3r1 |
| GO:0001956 | positive regulation of neurotransmitter secretion | 12h | 2 | Cacna1b, Sphk1 |
| GO:0090129 | positive regulation of synapse maturation | 12h | 2 | Camk2b, Nrxn1 |
| GO:0003084 | positive regulation of systemic arterial blood pressure | 12h | 2 | Cyba, Eng |
| GO:0048670 | regulation of collateral sprouting | 12h | 2 | Bcl11a, Spp1 |
| GO:0045652 | regulation of megakaryocyte differentiation | 12h | 2 | Hmgb2, Tesc |
| GO:0050932 | regulation of pigment cell differentiation | 12h | 2 | Adamts9, Kitl |
| GO:0090128 | regulation of synapse maturation | 12h | 2 | Camk2b, Nrxn1 |
| GO:0043618 | regulation of transcription from RNA polymerase II promoter in response to stress | 12h | 2 | Bach1, Hmox1 |
| GO:0007172 | signal complex assembly | 12h | 2 | Mapk8ip2, Ptk2b |

| | | | | |
|---|---|---|---|---|
| GO:0002246 | wound healing involved in inflammatory response | 12h | 2 | Cd44, Hmox1 |
