# Supplementary material for: Glial responses during epileptogenesis in Mus musculus point to potential therapeutic targets
Source: PLoS One. 2018 Aug 16;13(8):e0201742. doi: 10.1371/journal.pone.0201742 (PMC6095496; doi:10.1371/journal.pone.0201742)
Supplement: S7 Table — All significantly changed genes at 24h were considered, and a threshold of p-value <0.05 was applied. (PDF) [file pone.0201742.s011.pdf]

Table S7: Significantly changed GO Biological Processes (level 6) at 24 hours post KA treatment, using the "Mapping to ontologies (TRANSPATH)" analysis tool. All significantly changed genes at 24h were considered, and a threshold of p-value <0.05 was applied.

Gene Ontology Category ID	GO-Biologic Process (level 6)	Time point(s) of enrichment	Number of significantly changed genes at 24h	Symbol of significantly changed genes at 24h
GO:0031326	regulation of cellular biosynthetic process	6h, 12h, 24h	73	Aff1, Akap5, Arid5b, Atp8b1, Baz1a, Bcl11b, Calca, Capn3, Cdkn1a, Cdyl, Crh, Cyr61, Dkk3, Eaf1, Eif4ebp1, Elk3, Ell2, Eng, Etf1, Etv3, Fam120b, Fos, Fosl2, Gcg, Gpam, Grm8, Hbegf, Hivep3, Hmgn1, Hspb1, Htr1a, Icam1, Id2, Il11, Klf4, Lif, Litaf, Maff, Mbd2, Med13, Mybbp1a, Myd88, Neurod6, Nfkbia, Npas4, Nufip1, Pla2g4a, Plek, Ptgfrn, Ptx3, Rgs2, Rnf128, Rps27l, Rps6ka5, S1pr3, Samd4, Sap30, Sfpq, Sin3b, Tgif1, Thbs1, Tlr1, Trib1, Tspo, Tulp4, Wfs1, Wwtr1, Yy2, Zfp110, Zfp275, Zfp536, Zfp593, Zmiz1
GO:0019219	regulation of nucleobase-containing compound metabolic process	6h, 12h, 24h	66	Aff1, Akap5, Arid5b, Atp8b1, Baz1a, Bcl11b, Calca, Capn3, Cdkn1a, Cdyl, Chn1, Clcf1, Crh, Cyr61, Dkk3, Eaf1, Efna5, Efna5 (ENSMUSG00000090425), Elk3, Ell2, Eng, Epha3, Epha4, Etv3, Fam120b, Fos, Fosl2, Gcg, Grm8, Hivep3, Hmgn1, Htr1a, Icam1, Id2, Il11, Klf4, Lif, Litaf, Lrrk2, Maff, Mbd2, Med13, Mybbp1a, Myd88, Neurod6, Nfkbia, Npas4, Nufip1, Rgs2, Rps6ka5, S1pr3, Sap30, Sfpq, Sin3b, Tbc1d14, Tgif1, Trib1, Tulp4, Wfs1, Wwtr1, Yy2, Zfp110, Zfp275, Zfp536, Zfp593, Zmiz1
GO:0010468	regulation of gene expression	6h, 12h, 24h	64	Aff1, Arid5b, Atp8b1, Baz1a, Bcl11b, Calca, Capn3, Cd44, Cdkn1a, Cdyl, Crh, Cyr61, Dkk3, Eaf1, Eif4ebp1, Elk3, Ell2, Eng, Etf1, Etv3, Fam120b, Fos, Fosl2, Hivep3, Hmgn1, Hspb1, Icam1, Id2, Il11, Klf4, Lif, Litaf, Maff, Mbd2, Med13, Mybbp1a, Myd88, Neurod6, Nfkbia, Npas4, Nufip1, Ptgfrn, Rgs2, Rps27l, Rps6ka5, Samd4, Sap30, Serpine1, Sfpq, Sin3b, Tgif1, Thbs1, Tnc, Trdmt1, Trib1, Tulp4, Wfs1, Wwtr1, Yy2, Zfp110, Zfp275, Zfp536, Zfp593, Zmiz1

GO:0010556	regulation of macromolecule biosynthetic process	6h, 12h, 24h	64	Aff1, Arid5b, Atp8b1, Baz1a, Bcl11b, Calca, Capn3, Cdkn1a, Cdyl, Cyr61, Dkk3, Eaf1, Eif4ebp1, Elk3, Ell2, Eng, Etf1, Etv3, Fam120b, Fos, Fosl2, Hbegf, Hivep3, Hmgn1, Hspb1, Hsph1, Icam1, Id2, Il11, Itgav, Klf4, Lif, Litaf, Maff, Mbd2, Med13, Mybbp1a, Myd88, Neurod6, Nfkbia, Npas4, Nufip1, Ptgfrn, Rgs2, Rnf128, Rps27l, Rps6ka5, Samd4, Sap30, Sfpq, Sin3b, Tgif1, Thbs1, Tlr1, Trib1, Tulp4, Wfs1, Wwtr1, Yy2, Zfp110, Zfp275, Zfp536, Zfp593, Zmiz1
GO:0051252	regulation of RNA metabolic process	6h, 12h, 24h	50	Aff1, Arid5b, Atp8b1, Baz1a, Bcl11b, Calca, Capn3, Cdyl, Cyr61, Dkk3, Eaf1, Elk3, Ell2, Eng, Etv3, Fam120b, Fos, Fosl2, Hivep3, Hmgn1, Icam1, Id2, Il11, Klf4, Lif, Litaf, Maff, Mbd2, Med13, Mybbp1a, Myd88, Neurod6, Nfkbia, Npas4, Nufip1, Rps6ka5, Sap30, Sfpq, Sin3b, Tgif1, Trib1, Tulp4, Wfs1, Wwtr1, Yy2, Zfp110, Zfp275, Zfp536, Zfp593, Zmiz1
GO:0051246	regulation of protein metabolic process	6h, 12h, 24h	48	Akap5, Calca, Capn3, Cblb, Cd44, Cdkn1a, Clcf1, Crh, Cyr61, Edem1, Efna5, Efna5 (ENSMUSG00000090425), Eif4ebp1, Eng, Epha4, Etf1, Fgfr1op, Gadd45b, Gadd45g, Gcg, Grm1, Hspb1, Igfbp3, Il11, Il1rn, Itgav, Klf4, Lif, Lrrk2, Mbtps2, Myd88, Nfkbia, Prkar2a, Ptgfrn, Rgs2, Rnf128, Rps27l, Rps6ka5, Samd4, Serpine1, Serpini1, Thbs1, Thbs4, Timp1, Tlr1, Trib1, Wfs1, Wwtr1
GO:0031325	positive regulation of cellular metabolic process	6h, 12h, 24h	46	Aff1, Akap5, Bcl11b, Calca, Capn3, Cd44, Cdkn1a, Clcf1, Crh, Cyr61, Eaf1, Efna5, Efna5 (ENSMUSG00000090425), Ell2, Eng, Fos, Fosl2, Gcg, Gpam, Hivep3, Hmgn1, Hspb1, Icam1, Id2, Il11, Klf4, Lif, Lrrk2, Med13, Myd88, Nfkbia, Npas4, Nufip1, Pla2g4a, Ptx3, Rps27l, Rps6ka5, Samd4, Thbs1, Thbs4, Tlr1, Trib1, Tspo, Wfs1, Wwtr1, Zmiz1

GO:0010604	positive regulation of macromolecule metabolic process	6h, 12h, 24h	43	Aff1, Akap5, Bcl11b, Capn3, Cblb, Cd44, Cdkn1a, Clcf1, Crh, Cyr61, Eaf1, Efna5, Efna5 (ENSMUSG00000090425), Ell2, Eng, Fos, Fosl2, Gcg, Hivep3, Hmgn1, Hspb1, Hsph1, Id2, Il11, Klf4, Lif, Lrrk2, Med13, Myd88, Nfkbia, Npas4, Nufip1, Rps27l, Rps6ka5, Samd4, Thbs1, Thbs4, Tlr1, Tnc, Trib1, Wfs1, Wwtr1, Zmiz1
GO:0032268	regulation of cellular protein metabolic process	6h, 12h, 24h	43	Akap5, Calca, Capn3, Cd44, Cdkn1a, Clcf1, Crh, Cyr61, Edem1, Efna5, Efna5 (ENSMUSG00000090425), Eif4ebp1, Eng, Epha4, Etf1, Fgfr1op, Gadd45b, Gadd45g, Gcg, Grm1, Hspb1, Igfbp3, Il11, Il1rn, Itgav, Lif, Lrrk2, Mbtps2, Nfkbia, Prkar2a, Ptgfrn, Rgs2, Rps27l, Rps6ka5, Samd4, Serpine1, Thbs1, Thbs4, Timp1, Tlr1, Trib1, Wfs1, Wwtr1
GO:0043067	regulation of programmed cell death	6h, 12h, 24h	42	Arhgef4, Bag3, Bcl11b, Birc3, Capn3, Casp8, Cd44, Cdkn1a, Clcf1, Crh, Cstb, Ctnna1, Ctsc, Cyr61, Eif2ak2, Gcg, Gpam, Grm8, Hspa1b, Hspb1, Hsph1, Igfbp3, Il1rn, Itgav, Kcnma1, Lgals1, Lrrk2, Mcl1, Mybbp1a, Myd88, Nfkbia, Nmt1, Pla2g4a, Rps27l, Scg2, Serpine1, Spp1, Tgm2, Thbs1, Timp1, Tspo, Wfs1
GO:0009891	positive regulation of biosynthetic process	6h, 12h, 24h	37	Aff1, Akap5, Bcl11b, Calca, Capn3, Crh, Cyr61, Eaf1, Ell2, Eng, Fos, Fosl2, Gcg, Gpam, Hivep3, Hmgn1, Hspb1, Hsph1, Icam1, Id2, Il11, Klf4, Lif, Med13, Myd88, Nfkbia, Npas4, Nufip1, Pla2g4a, Ptx3, Rps27l, Rps6ka5, Samd4, Thbs1, Tlr1, Wwtr1, Zmiz1
GO:0051174	regulation of phosphorus metabolic process	6h, 12h, 24h	36	Akap5, Calca, Cd44, Cdkn1a, Clcf1, Crh, Cyr61, Edem1, Efna5, Efna5 (ENSMUSG00000090425), Eng, Epha4, Fgfr1op, Fkbp1b, Gadd45b, Gadd45g, Gcg, Grm1, Hspb1, Igfbp3, Il11, Il1rn, Lif, Lrrk2, Mbtps2, Pcdh11x, Plek, Prkar2a, Rgs2, Rps6ka5, Thbs1, Thbs4, Tlr1, Trib1, Wfs1, Wwtr1
GO:0031324	negative regulation of cellular metabolic process	6h, 12h, 24h	33	Arid5b, Atp8b1, Calca, Capn3, Cdkn1a, Dkk3, Eif4ebp1, Elk3, Eng, Etv3, Grm8, Hbegf, Hspb1, Htr1a, Id2, Igfbp3, Itgav, Klf4, Mbd2, Mybbp1a, Plek, Ptgfrn, Rnf128, Rps6ka5, S1pr3, Samd4, Sap30, Serpine1, Sin3b, Tgif1, Timp1, Tspo, Wwtr1

GO:0008284	positive regulation of cell proliferation	6h, 12h, 24h	30	Cdkn1a, Clcf1, Crh, Cyr61, Fgfr1op, Fosl2, Gfap, Gpam, Hbegf, Htr1a, Id2, Il11, Il13ra1, Itgav, Lif, Myd88, Odc1, Osmr, Pla2g4a, S1pr3, Scg2, Tbrg4, Tgif1, Tgm2, Thbs4, Timp1, Tnc, Tspo, Wwtr1, Zmiz1
GO:0051173	positive regulation of nitrogen compound metabolic process	6h, 12h, 24h	30	Aff1, Akap5, Bcl11b, Calca, Capn3, Clcf1, Crh, Cyr61, Eaf1, Ell2, Eng, Fos, Fosl2, Gcg, Hivep3, Hmgn1, Icam1, Id2, Il11, Klf4, Lif, Med13, Myd88, Nfkbia, Npas4, Nufip1, Ptx3, Rps6ka5, Wwtr1, Zmiz1
GO:0009890	negative regulation of biosynthetic process	6h, 12h, 24h	28	Arid5b, Atp8b1, Calca, Capn3, Dkk3, Eif4ebp1, Elk3, Eng, Etv3, Grm8, Hbegf, Htr1a, Id2, Itgav, Klf4, Mbd2, Mybbp1a, Plek, Ptgfrn, Rnf128, Rps6ka5, S1pr3, Samd4, Sap30, Sin3b, Tgif1, Tspo, Wwtr1
GO:0010605	negative regulation of macromolecule metabolic process	6h, 12h, 24h	28	Arid5b, Atp8b1, Calca, Capn3, Cdkn1a, Crh, Dkk3, Eif4ebp1, Elk3, Eng, Etv3, Hbegf, Id2, Igfbp3, Itgav, Klf4, Mbd2, Mybbp1a, Ptgfrn, Rnf128, Rps6ka5, Samd4, Sap30, Serpine1, Sin3b, Tgif1, Timp1, Wwtr1
GO:0060548	negative regulation of cell death	6h, 12h, 24h	26	Bag3, Bcl11b, Birc3, Capn3, Cd44, Cdkn1a, Clcf1, Crh, Ctnna1, Cyr61, Gcg, Gpam, Hspa1b, Hspb1, Hsph1, Il1rn, Itgav, Mcl1, Myd88, Nfkbia, Scg2, Serpine1, Spp1, Thbs1, Timp1, Wfs1
GO:0016310	phosphorylation	12h, 24h	26	Calca, Cdk14, Cdkn1a, Dclk1, Dclk3, Dyrk3, Eif2ak2, Epha3, Epha4, Gadd45b, Gadd45g, Grm1, Igfbp3, Lif, Lrrk2, Nek6, Pak3, Papss2, Prkar2a, Riok2, Rps6ka5, Tbrg4, Thbs1, Trib1, Uck2, Yes1
GO:0010647	positive regulation of cell communication	6h, 12h, 24h	25	Akap5, Casp8, Cd44, Clcf1, Crh, Ctnna1, Cyr61, Dock2, Eng, Gcg, Gfap, Hbegf, Igfbp3, Il11, Itgav, Lgals1, Lif, Litaf, Lrrk2, Mbd2, Myd88, Nek6, Tgm2, Thbs1, Zdhhc17
GO:0010627	regulation of intracellular protein kinase cascade	12h, 24h	25	Akap5, Capn3, Casp8, Cd44, Clcf1, Cyr61, Epha4, Gcg, Grm1, Hbegf, Hspb1, Igfbp3, Il11, Il1rn, Itgav, Klf4, Lgals1, Lif, Litaf, Myd88, Nek6, Tgm2, Thbs1, Zdhhc17, Zfp110

GO:0009967	positive regulation of signal transduction	12h, 24h	23	Akap5, Casp8, Cd44, Clcf1, Ctnna1, Cyr61, Dock2, Eng, Gcg, Hbegf, Igfbp3, Il11, Itgav, Lgals1, Lif, Litaf, Lrrk2, Mbd2, Myd88, Nek6, Tgm2, Thbs1, Zdhhc17
GO:0006468	protein phosphorylation	12h, 24h	22	Calca, Cdk14, Dclk1, Dclk3, Dyrk3, Eif2ak2, Epha3, Epha4, Gadd45b, Gadd45g, Grm1, Igfbp3, Lif, Lrrk2, Nek6, Pak3, Riok2, Rps6ka5, Tbrg4, Thbs1, Trib1, Yes1
GO:0008285	negative regulation of cell proliferation	12h, 24h	21	Bcl11b, Cblb, Cd9, Cdkn1a, Cgref1, Ctnna1, Eif2ak2, Eng, Fcgr2b, Gpnmb, Hspa1b, Id2, Igfbp3, Klf4, Lif, Lrrk2, Scg2, Tgif1, Thbs1, Trib1, Tspo
GO:0051172	negative regulation of nitrogen compound metabolic process	6h, 12h, 24h	21	Arid5b, Atp8b1, Calca, Capn3, Dkk3, Elk3, Eng, Etv3, Grm8, Htr1a, Id2, Klf4, Mbd2, Mybbp1a, Rps6ka5, S1pr3, Sap30, Sin3b, Tgif1, Tspo, Wwtr1
GO:0043549	regulation of kinase activity	6h, 12h, 24h	21	Calca, Cdkn1a, Cyr61, Edem1, Epha4, Fgfr1op, Gadd45b, Gadd45g, Gcg, Grm1, Hspb1, Il1rn, Lrrk2, Mbtps2, Prkar2a, Rgs2, Thbs1, Tlr1, Trib1, Wfs1, Wwtr1
GO:0007167	enzyme linked receptor protein signaling pathway	6h, 12h, 24h	20	Adam12, Arhgef4, Arid5b, Chn1, Efna5, Efna5 (ENSMUSG00000090425), Eif4ebp1, Eng, Epha3, Epha4, Erbb2ip, Fos, Grem2, Hbegf, Lif, Ltbp1, Myd88, Nfkbia, Prkar2a, Rps6ka5
GO:0060284	regulation of cell development	12h, 24h	20	Akap5, Arf6, Cdh4, Chn1, Clcf1, Ctnna1, Epha3, Epha4, Gfap, Id2, Igfbp3, Lgals1, Lif, Lrrk2, Spp1, Tgif1, Tspo, Vim, Wwtr1, Zfp536
GO:0007010	cytoskeleton organization	12h, 24h	19	Arf6, Cald1, Capn3, Dock2, Erbb2ip, Fgfr1op, Fhod3, Gcc2, Gfap, Kif18a, Nedd1, Nedd9, Nek6, Plek, Rnd3, Shroom3, Svil, Synpo, Vim
GO:0010648	negative regulation of cell communication	12h, 24h	19	Cblb, Cd44, Dkk3, Eng, Igfbp3, Il1rn, Klf4, Lif, Ltbp1, Mcl1, Mgll, Nfkbia, Plek, Rgs2, Tgif1, Thbs1, Trib1, Wwtr1, Zfp536
GO:0009968	negative regulation of signal transduction	12h, 24h	18	Cblb, Cd44, Dkk3, Eng, Igfbp3, Il1rn, Klf4, Lif, Ltbp1, Mcl1, Nfkbia, Plek, Rgs2, Tgif1, Thbs1, Trib1, Wwtr1, Zfp536
GO:0010942	positive regulation of cell death	6h, 12h, 24h	18	Arhgef4, Casp8, Cd44, Cdkn1a, Crh, Ctsc, Cyr61, Eif2ak2, Igfbp3, Kcnma1, Lrrk2, Mybbp1a, Nmt1, Pla2g4a, Rps27l, Tgm2, Thbs1, Tspo

GO:0031347	regulation of defense response	12h, 24h	18	Calca, Casp8, Clcf1, Crh, Dock2, Fcgr2b, Fos, Klf4, Mgll, Myd88, Nfkbia, Osmr, Pla2g4a, Pvr, Rps6ka5, Serpine1, Tgm2, Tlr1
GO:0043269	regulation of ion transport	6h, 12h, 24h	18	Akap5, Calca, Capn3, Clic4, Crh, Cybb, Fkbp1b, Gcg, Icam1, Kcnab1, Kcnma1, Kcnq3, Kcnq5, Scn2a1, Scn3b, Scn8a (ENSMUSG00000023033), Tspo, Wfs1
GO:0051960	regulation of nervous system development	12h, 24h	18	Akap5, Arf6, Cdh4, Chn1, Clcf1, Ctnna1, Epha3, Epha4, Gfap, Id2, Lgals1, Lif, Lrrk2, Spp1, Tgif1, Tspo, Vim, Zfp536
GO:0030001	metal ion transport	12h, 24h	16	Fkbp1b, Itgav, Kcnab1, Kcnma1, Kcnq3, Kcnq5, Kcnt2, Kctd4, Mmgt2, Npy, Scn2a1, Scn3b, Scn8a (ENSMUSG00000023033), Slc10a6, Slc6a8, Zdhhc17
GO:0030334	regulation of cell migration	12h, 24h	16	Acan, Amotl1, C3ar1, Clic4, Cyr61, Fgfr1op, Hbegf, Icam1, Igfbp3, Itgav, Klf4, Msn, Serpine1, Thbs1, Thbs4, Trib1
GO:0051046	regulation of secretion	12h, 24h	16	Akap5, Cadps2, Clcf1, Crh, Efna5, Efna5 (ENSMUSG00000090425), Gcg, Gpam, Grm1, Grm8, Il11, Il1rn, Lif, Prkar2a, Srgn, Syt4
GO:0050801	ion homeostasis	12h, 24h	15	C3ar1, Calca, Crh, Fkbp1b, Grm1, Itgav, Kcnma1, Lrrk2, Mt2, S1pr3, Scn2a1, Scn3b, Scn8a (ENSMUSG00000023033), Tgm2, Wfs1
GO:0045597	positive regulation of cell differentiation	12h, 24h	15	Adamts9, Akap5, Calca, Cdh4, Clcf1, Ctnna1, Cyr61, Gfap, Id2, Igfbp3, Lif, Msr1, Tgif1, Tspo, Wwtr1
GO:0051347	positive regulation of transferase activity	12h, 24h	15	Calca, Cyr61, Edem1, Epha4, Gadd45b, Gadd45g, Gcg, Grm1, Il1rn, Lrrk2, Mbtps2, Prkar2a, Thbs1, Tlr1, Wfs1
GO:0045596	negative regulation of cell differentiation	12h, 24h	13	Calca, Ctnna1, Epha4, Id2, Itgav, Klf4, Lif, Lrrk2, Nfkbia, Spp1, Tspo, Wwtr1, Zfp536
GO:0051346	negative regulation of hydrolase activity	12h, 24h	13	Cd44, Cstb, Ctla2b, Fkbp1b, Hspa1b, Klf4, Pcdh11x, Plek, Pros1, Rgs2, Serpine1, Serpini1, Thbs1
GO:0018193	peptidyl-amino acid modification	12h, 24h	13	Agtpbp1, Bgn, Dyrk3, Edem1, Epha4, Fkbp1b, Lif, Lrrk2, Nek6, Nmt1, Pros1, Rps6ka5, Tgm2
GO:0051345	positive regulation of hydrolase activity	12h, 24h	13	Casp8, Chn1, Cyr61, Edem1, Grm1, Lrrk2, Mbtps2, Plek, Prkar2a, Rgs2, Rps27l, Tgm2, Wfs1
GO:0007243	intracellular protein kinase cascade	6h, 12h, 24h	12	Clcf1, Fos, Gcg, Itgav, Lrrk2, Myd88, Nmi, Pak3, Plek, Rps6ka5, Scg2, Trib1

GO:0015672	monovalent inorganic cation transport	12h, 24h	12	Agtpbp1, Kcnab1, Kcnma1, Kcnq3, Kcnq5, Kcnt2, Kctd4, Scn2a1, Scn3b, Scn8a (ENSMUSG00000023033), Slc10a6, Slc6a8
GO:0031349	positive regulation of defense response	12h, 24h	12	Casp8, Crh, Fos, Myd88, Nfkbia, Osmr, Pla2g4a, Pvr, Rps6ka5, Serpine1, Tgm2, Tlr1
GO:0050778	positive regulation of immune response	12h, 24h	12	C1qb, C3ar1, Casp8, Cd44, Clcf1, Fos, Hspa1b, Myd88, Nfkbia, Pvr, Rps6ka5, Tlr1
GO:0034765	regulation of ion transmembrane transport	6h, 12h, 24h	12	Clic4, Crh, Cybb, Fkbp1b, Gcg, Kcnab1, Kcnma1, Kcnq3, Kcnq5, Scn2a1, Scn3b, Scn8a (ENSMUSG00000023033)
GO:0010975	regulation of neuron projection development	12h, 24h	12	Akap5, Arf6, Cdh4, Chn1, Epha3, Epha4, Gfap, Lgals1, Lif, Lrrk2, Spp1, Vim
GO:0052547	regulation of peptidase activity	12h, 24h	12	Casp8, Cd44, Cstb, Ctla2b, Cyr61, Hspa1b, Klf4, Pros1, Rps27l, Serpine1, Serpini1, Thbs1
GO:0019932	second-messenger-mediated signaling	6h, 12h, 24h	11	Calca, Fkbp1b, Gcg, Grm1, Grm8, Gucy1b3, Htr1a, Mt2, Npy, S1pr3, Tgm2
GO:0048858	cell projection morphogenesis	6h, 24h	10	Bcl11b, Ctnna1, Dclk1, Epha4, Gfap, Pak3, Thbs4, Tnc, Tspo, Wwtr1
GO:0030003	cellular cation homeostasis	12h, 24h	10	C3ar1, Calca, Fkbp1b, Grm1, Itgav, Kcnma1, Mt2, S1pr3, Tgm2, Wfs1
GO:0050867	positive regulation of cell activation	24h	10	Capn3, Cdkn1a, Clcf1, Gpam, Hsph1, Il13ra1, Myd88, Plek, Thbs1, Yes1
GO:2000147	positive regulation of cell motility	12h, 24h	10	Amotl1, C3ar1, Cyr61, Fgfr1op, Hbegf, Icam1, Itgav, Serpine1, Thbs1, Thbs4
GO:0051272	positive regulation of cellular component movement	12h, 24h	10	Amotl1, C3ar1, Cyr61, Fgfr1op, Hbegf, Icam1, Itgav, Serpine1, Thbs1, Thbs4
GO:0051493	regulation of cytoskeleton organization	12h, 24h	10	Arf6, Arpc1b, Clic4, Efna5, Efna5 (ENSMUSG00000090425), Epha3, Hsph1, Pak3, Plek, Synpo
GO:0050727	regulation of inflammatory response	12h, 24h	10	Calca, Clcf1, Fcgr2b, Klf4, Mgll, Myd88, Osmr, Pla2g4a, Serpine1, Tgm2
GO:0051249	regulation of lymphocyte activation	24h	10	Cblb, Cdkn1a, Clcf1, Fcgr2b, Gpam, Hsph1, Id2, Il13ra1, Myd88, Yes1
GO:0031960	response to corticosteroid stimulus	6h, 12h, 24h	10	C1qb, Cdkn1a, Crh, Eng, Fos, Il1rn, Kcnma1, Pla2g4a, Sdc1, Serpine1

GO:0032103	positive regulation of response to external stimulus	12h, 24h	9	C3ar1, Npy, Osmr, Pla2g4a, Scg2, Serpine1, Tgm2, Thbs1, Thbs4
GO:0045765	regulation of angiogenesis	12h, 24h	9	C3ar1, Cyr61, Eng, Klf4, Lif, Rnh1, Serpine1, Thbs1, Thbs4
GO:0010769	regulation of cell morphogenesis involved in differentiation	12h, 24h	9	Akap5, Cdh4, Chn1, Epha3, Epha4, Lif, Lrrk2, Spp1, Wwtr1
GO:0010810	regulation of cell-substrate adhesion	12h, 24h	9	Cyr61, Efna5, Efna5 (ENSMUSG00000090425), Egfl6, Epha3, Lgals1, Serpine1, Spp1, Thbs1
GO:0070201	regulation of establishment of protein localization	12h, 24h	9	Akap5, Arf6, Cdkn1a, Gcc2, Gpam, Nedd1, Nfkbia, Srgn, Wwtr1
GO:0019216	regulation of lipid metabolic process	24h	9	Bcl11b, Cyr61, Dkk3, Gcg, Gpam, Id2, Kcnma1, Pla2g4a, Tspo
GO:0019221	cytokine-mediated signaling pathway	12h, 24h	8	Cd44, Clcf1, Csf2rb2, Icam1, Il13ra1, Myd88, Osmr, Rps6ka5
GO:0051348	negative regulation of transferase activity	6h, 12h, 24h	8	Cdkn1a, Fgfr1op, Gadd45b, Gadd45g, Hspb1, Rgs2, Trib1, Wwtr1
GO:0045785	positive regulation of cell adhesion	6h, 12h, 24h	8	Calca, Cyr61, Egfl6, Itgav, Lgals1, Spp1, Tgm2, Thbs1
GO:0002696	positive regulation of leukocyte activation	24h	8	Cdkn1a, Clcf1, Gpam, Hsph1, Il13ra1, Myd88, Thbs1, Yes1
GO:0006457	protein folding	24h	8	Bag2, Bag3, Edem1, Fkbp1b, Hspa1b, Hsph1, Tubb6, Wfs1
GO:0032956	regulation of actin cytoskeleton organization	24h	8	Arf6, Arpc1b, Efna5, Efna5 (ENSMUSG00000090425), Epha3, Pak3, Plek, Synpo
GO:0043087	regulation of GTPase activity	12h, 24h	8	Chn1, Efna5, Efna5 (ENSMUSG00000090425), Epha3, Epha4, Lrrk2, Rgs2, Tbc1d14
GO:0030198	extracellular matrix organization	12h, 24h	7	Acan, Anxa2, Crispld2, Cyr61, Egfl6, Eng, Gfap
GO:2000146	negative regulation of cell motility	24h	7	Acan, Clic4, Igfbp3, Klf4, Serpine1, Thbs1, Trib1
GO:0051271	negative regulation of cellular component movement	24h	7	Acan, Clic4, Igfbp3, Klf4, Serpine1, Thbs1, Trib1

GO:0045807	positive regulation of endocytosis	12h, 24h	7	Caly, Fcgr2b, Itgav, Pros1, Ptx3, Serpine1, Tub
GO:0043270	positive regulation of ion transport	12h, 24h	7	Akap5, Capn3, Crh, Gcg, Scn3b, Tspo, Wfs1
GO:0045088	regulation of innate immune response	12h, 24h	7	Casp8, Fos, Myd88, Nfkbia, Pvr, Rps6ka5, Tlr1
GO:0070663	regulation of leukocyte proliferation	24h	7	Cblb, Cdkn1a, Clcf1, Fcgr2b, Gpam, Il13ra1, Myd88
GO:0060191	regulation of lipase activity	12h, 24h	7	Cyr61, Fkbp1b, Grm1, Plek, Prkar2a, Rgs2, Tgm2
GO:0090257	regulation of muscle system process	12h, 24h	7	Calca, Cald1, Fkbp1b, Kcnma1, Klf4, Rgs2, Scn3b
GO:0048660	regulation of smooth muscle cell proliferation	6h, 12h, 24h	7	Hbegf, Id2, Igfbp3, Klf4, Myd88, Tgm2, Trib1
GO:0051592	response to calcium ion	6h, 12h, 24h	7	Capn3, Clic4, Fos, Kcnma1, Pla2g4a, Sdc1, Thbs1
GO:0007162	negative regulation of cell adhesion	24h	6	Il1rn, Klf4, Lgals1, Serpine1, Thbs1, Tnc
GO:0030308	negative regulation of cell growth	12h, 24h	6	Cdkn1a, Cgref1, Gng4, Hspa1b, Ndrg3, Spp1
GO:0001818	negative regulation of cytokine production	24h	6	Fcgr2b, Klf4, Rnf125, Rps6ka5, Thbs1, Tspo
GO:0032102	negative regulation of response to external stimulus	12h, 24h	6	Epha4, Fcgr2b, Gcg, Klf4, Serpine1, Spp1
GO:0002221	pattern recognition receptor signaling pathway	12h, 24h	6	Casp8, Fos, Myd88, Nfkbia, Rps6ka5, Tlr1
GO:0048520	positive regulation of behavior	12h, 24h	6	C3ar1, Crh, Scg2, Serpine1, Thbs1, Thbs4
GO:0001819	positive regulation of cytokine production	12h, 24h	6	C3ar1, Calca, Hspb1, Myd88, Serpine1, Thbs1
GO:0010638	positive regulation of organelle organization	12h, 24h	6	Anxa2, Arf6, Pla2g4a, Plek, Rps6ka5, Synpo
GO:0042035	regulation of cytokine biosynthetic process	12h, 24h	6	Hspb1, Klf4, Myd88, Rnf128, Thbs1, Tlr1

GO:0032386	regulation of intracellular transport	12h, 24h	6	Akap5, Cdkn1a, Fkbp1b, Gcc2, Nfkbia, Wwtr1
GO:0042391	regulation of membrane potential	12h, 24h	6	Grm1, Kcnma1, Lrrk2, Scn2a1, Scn3b, Scn8a (ENSMUSG00000023033)
GO:0090276	regulation of peptide hormone secretion	12h, 24h	6	Akap5, Crh, Efna5, Efna5 (ENSMUSG00000090425), Gcg, Prkar2a
GO:0090087	regulation of peptide transport	12h, 24h	6	Akap5, Crh, Efna5, Efna5 (ENSMUSG00000090425), Gcg, Prkar2a
GO:0061041	regulation of wound healing	6h, 12h, 24h	6	Anxa2, Capn3, Hbegf, Plek, Serpine1, Thbs1
GO:0034284	response to monosaccharide stimulus	12h, 24h	6	Acan, Fkbp1b, Gpam, Icam1, Lgals1, Thbs1
GO:0034623	cellular macromolecular complex disassembly	24h	5	Akap5, Etf1, Kif18a, Nedd1, Rpl12
GO:0071222	cellular response to lipopolysaccharide	12h, 24h	5	Icam1, Myd88, Nfkbia, Serpine1, Tspo
GO:0071248	cellular response to metal ion	6h, 12h, 24h	5	Capn3, Clic4, Fos, Id2, Tspo
GO:0016525	negative regulation of angiogenesis	12h, 24h	5	Klf4, Lif, Serpine1, Thbs1, Thbs4
GO:0031345	negative regulation of cell projection organization	12h, 24h	5	Epha4, Gfap, Lgals1, Spp1, Vim
GO:0051048	negative regulation of secretion	12h, 24h	5	Crh, Il11, Il1rn, Lif, Srgn
GO:0031175	neuron projection development	24h	5	Arf6, Cd44, Npy, Pak3, Tnc
GO:0045766	positive regulation of angiogenesis	12h, 24h	5	C3ar1, Cyr61, Eng, Serpine1, Thbs1
GO:0050921	positive regulation of chemotaxis	12h, 24h	5	C3ar1, Scg2, Serpine1, Thbs1, Thbs4
GO:0002687	positive regulation of leukocyte migration	12h, 24h	5	C3ar1, Icam1, Serpine1, Thbs1, Thbs4
GO:0051092	positive regulation of NF-kappaB transcription factor activity	12h, 24h	5	Capn3, Icam1, Myd88, Nfkbia, Rps6ka5

GO:0043241	protein complex disassembly	24h	5	Akap5, Etf1, Kif18a, Nedd1, Rpl12
GO:0002819	regulation of adaptive immune response	24h	5	Cd44, Clcf1, Fcgr2b, Hspa1b, Pvr
GO:0022407	regulation of cell-cell adhesion	12h, 24h	5	Efna5, Efna5 (ENSMUSG00000090425), Il1rn, Klf4, Lgals1
GO:0008277	regulation of G-protein coupled receptor protein signaling pathway	24h	5	Gng4, Lrrk2, Mgll, Plek, Rgs2
GO:0008016	regulation of heart contraction	6h, 12h, 24h	5	Calca, Fkbp1b, Hbegf, Rgs2, Scn3b
GO:0046890	regulation of lipid biosynthetic process	6h, 24h	5	Cyr61, Dkk3, Gpam, Pla2g4a, Tspo
GO:0050764	regulation of phagocytosis	24h	5	Fcgr2b, Itgav, Pros1, Ptx3, Tub
GO:0032680	regulation of tumor necrosis factor production	12h, 24h	5	Hspb1, Myd88, Thbs1, Tlr1, Tspo
GO:0030325	adrenal gland development	24h	4	Arid5b, Crh, Dkk3, Tspo
GO:0034620	cellular response to unfolded protein	24h	4	Edem1, Eif2ak2, Mbtps2, Wfs1
GO:0030968	endoplasmic reticulum unfolded protein response	24h	4	Edem1, Eif2ak2, Mbtps2, Wfs1
GO:0007229	integrin-mediated signaling pathway	24h	4	Erbb2ip, Itgav, Nedd9, Plek
GO:0046887	positive regulation of hormone secretion	24h	4	Clcf1, Crh, Gcg, Lif
GO:0008064	regulation of actin polymerization or depolymerization	12h, 24h	4	Arf6, Arpc1b, Pak3, Plek
GO:0030193	regulation of blood coagulation	12h, 24h	4	Anxa2, Plek, Serpine1, Thbs1
GO:0048145	regulation of fibroblast proliferation	6h, 12h, 24h	4	Cdkn1a, Fosl2, Tgif1, Zmiz1

GO:0051893	regulation of focal adhesion assembly	24h	4	Efna5, Efna5 (ENSMUSG00000090425), Epha3, Thbs1
GO:0002688	regulation of leukocyte chemotaxis	12h, 24h	4	C3ar1, Serpine1, Thbs1, Thbs4
GO:0032368	regulation of lipid transport	24h	4	Itgav, Nfkbia, Thbs1, Tspo
GO:0070507	regulation of microtubule cytoskeleton organization	24h	4	Efna5, Efna5 (ENSMUSG00000090425), Epha3, Hsph1
GO:0033762	response to glucagon stimulus	24h	4	Gcg, Gng2, Gng4, Prkar2a
GO:0032570	response to progesterone stimulus	6h, 12h, 24h	4	Fos, Fosl2, Thbs1, Tspo
GO:0051403	stress-activated MAPK cascade	6h, 12h, 24h	4	Fos, Myd88, Rps6ka5, Trib1
GO:0008063	Toll signaling pathway	12h, 24h	4	Fos, Myd88, Nfkbia, Rps6ka5
GO:0046631	alpha-beta T cell activation	12h, 24h	3	Bcl11b, Dock2, Gadd45g
GO:0048708	astrocyte differentiation	24h	3	Gfap, Lif, Vim
GO:0031103	axon regeneration	24h	3	Ctnna1, Tnc, Tspo
GO:0050829	defense response to Gram-negative bacterium	24h	3	Lyz1, Lyz2, Serpine1
GO:0050830	defense response to Gram-positive bacterium	24h	3	Lyz1, Lyz2, Myd88
GO:0030218	erythrocyte differentiation	24h	3	Dyrk3, Id2, Mfsd7b
GO:0007215	glutamate receptor signaling pathway	12h, 24h	3	Gria3, Grm1, Grm8
GO:0050819	negative regulation of coagulation	12h, 24h	3	Anxa2, Pros1, Serpine1
GO:0046888	negative regulation of hormone secretion	12h, 24h	3	Crh, Il11, Lif
GO:0043271	negative regulation of ion transport	12h, 24h	3	Akap5, Calca, Icam1
GO:0045932	negative regulation of muscle contraction	6h, 24h	3	Calca, Kcnma1, Rgs2

GO:0034763	negative regulation of transmembrane transport	24h	3	Nfkbia, Thbs1, Wwtr1
GO:0018149	peptide cross-linking	24h	3	Bgn, Tgm2, Thbs1
GO:0046850	regulation of bone remodeling	24h	3	Calca, Itgav, Spp1
GO:0032652	regulation of interleukin-1 production	24h	3	Calca, Hspb1, S1pr3
GO:0032677	regulation of interleukin-8 production	12h, 24h	3	Calca, Klf4, Serpine1
GO:0010743	regulation of macrophage derived foam cell differentiation	24h	3	Itgav, Msr1, Nfkbia
GO:2000177	regulation of neural precursor cell proliferation	24h	3	Ctnna1, Id2, Lrrk2
GO:0045428	regulation of nitric oxide biosynthetic process	12h, 24h	3	Icam1, Ptx3, Tspo
GO:0033688	regulation of osteoblast proliferation	24h	3	Cyr61, Eif2ak2, Itgav
GO:2000377	regulation of reactive oxygen species metabolic process	12h, 24h	3	Cdkn1a, Thbs1, Tspo
GO:0010226	response to lithium ion	12h, 24h	3	Gria3, Id2, Pla2g4a
GO:0033280	response to vitamin D	24h	3	Pla2g4a, Spp1, Tnc
GO:0043277	apoptotic cell clearance	24h	2	Itgav, Tgm2
GO:0014002	astrocyte development	24h	2	Gfap, Vim
GO:0060602	branch elongation of an epithelium	24h	2	Rdh10, Tnc
GO:0002544	chronic inflammatory response	12h, 24h	2	Il1rn, Thbs1
GO:0042089	cytokine biosynthetic process	24h	2	Gadd45g, Myd88
GO:0001660	fever generation	24h	2	Epha3, Il1rn
GO:0035329	hippo signaling cascade	24h	2	Amotl1, Wwtr1
GO:0007019	microtubule depolymerization	24h	2	Kif18a, Nedd1

GO:0008045	motor axon guidance	12h, 24h	2	Chn1, Epha4
GO:0032369	negative regulation of lipid transport	24h	2	Itgav, Thbs1
GO:0070571	negative regulation of neuron projection regeneration	12h, 24h	2	Epha4, Spp1
GO:0014003	oligodendrocyte development	24h	2	Cd9, Id2
GO:0010770	positive regulation of cell morphogenesis involved in differentiation	24h	2	Lif, Wwtr1
GO:0050820	positive regulation of coagulation	12h, 24h	2	Serpine1, Thbs1
GO:1900048	positive regulation of hemostasis	12h, 24h	2	Serpine1, Thbs1
GO:0035774	positive regulation of insulin secretion involved in cellular response to glucose stimulus	24h	2	Crh, Gcg
GO:0031340	positive regulation of vesicle fusion	24h	2	Anxa2, Pla2g4a
GO:0031077	post-embryonic camera-type eye development	24h	2	Bcl11b, Klf4
GO:0022417	protein maturation by protein folding	24h	2	Fkbp1b, Wfs1
GO:0032344	regulation of aldosterone metabolic process	24h	2	Dkk3, Kcnma1
GO:0048679	regulation of axon regeneration	24h	2	Epha4, Spp1
GO:0051459	regulation of corticotropin secretion	24h	2	Crh, Lif
GO:0060251	regulation of glial cell proliferation	24h	2	Gfap, Tspo
GO:0071675	regulation of mononuclear cell migration	12h, 24h	2	C3ar1, Thbs1

GO:0070570	regulation of neuron projection regeneration	24h	2	Epha4, Spp1
GO:0048385	regulation of retinoic acid receptor signaling pathway	24h	2	Tgif1, Zfp536
GO:0001914	regulation of T cell mediated cytotoxicity	24h	2	Hspa1b, Pvr
GO:0031338	regulation of vesicle fusion	24h	2	Anxa2, Pla2g4a
GO:0060087	relaxation of vascular smooth muscle	24h	2	Kcnma1, Rgs2
GO:0043330	response to exogenous dsRNA	24h	2	Eif2ak2, Nfkbia
GO:0052572	response to host immune response	24h	2	Eif2ak2, Tlr1
GO:0033197	response to vitamin E	24h	2	Eif2ak2, Fkbp1b
GO:0031290	retinal ganglion cell axon guidance	24h	2	Efna5, Efna5 (ENSMUSG00000090425)
GO:0031529	ruffle organization	24h	2	Arf6, Plek
GO:0002286	T cell activation involved in immune response	12h, 24h	2	Gadd45g, Icam1
GO:0060707	trophoblast giant cell differentiation	24h	2	Itgav, Lif
