# Supplementary material for: Glial responses during epileptogenesis in Mus musculus point to potential therapeutic targets
Source: PLoS One. 2018 Aug 16;13(8):e0201742. doi: 10.1371/journal.pone.0201742 (PMC6095496; doi:10.1371/journal.pone.0201742)
Supplement: S8 Table — All significantly changed genes at 6h were considered, and a threshold of p-value <0.05 was applied. (PDF) [file pone.0201742.s012.pdf]

Table S8: Significantly changed GO Molecular Functions (level 6) at 6 hours post KA treatment, using the "Mapping to ontologies (TRANSPATH®)" workflow. All significantly changed genes at 6h were considered, and a threshold of p-value <0.05 was applied.

Gene Ontology Category ID	GO-Molecular Function (level 6)	Time point(s) of enrichment	Number of significantly changed genes at 6h	Symbol of significantly changed genes at 6h
GO:0046914	transition metal ion binding	6h, 12h	31	Adamts1, Arhgef2, Arih2, Baz1a, Bcl11b, Cyp51, Egr4, Foxp1, Gucy1b3, Kdm4b, Mt1, Nr3c2, Nr4a1, Nr4a2, Nr4a3, Plagl1, Ptgs2, Rnf217, Rorb, Siah2, Tiparp, Usp45, Zc3h12c, Zdbf2, Zeb2, Zfp12, Zfp292, Zfp36, Zfp462, Zfp763, Zswim6
GO:0044212	transcription regulatory region DNA binding	6h, 12h	9	Bcl11b, E2f3, Egr4, Fos, Jun, Mterfd3, Neurod2, Per1, Smad7
GO:0022836	gated channel activity	6h, 12h, 24h	8	Cacna1h, Gabrg2, Kcnf1, Kcnip2, Kcns2, Kcnv1, Kctd4, Kctd6
GO:0003690	double-stranded DNA binding	6h, 12h	7	Fos, Fosb, Foxk1, Foxp1, Jun, Junb, Nr3c2
GO:0016791	phosphatase activity	6h	7	Acpl2, Dusp1, Dusp5, Mtmr7, Nanp, Ptpn12, Ptpn3
GO:0008201	heparin binding	6h, 12h, 24h	6	Adamts1, Cyr61, Gpnmb, Hbegf, Selp, Smoc2
GO:0001077	RNA polymerase II core promoter proximal region sequence-specific DNA binding transcription factor activity involved in positive regulation of transcription	6h	5	Bcl11b, Jun, Nr4a1, Nr4a3, Zfp292
GO:0005088	Ras guanyl-nucleotide exchange factor activity	6h, 12h	4	Arhgef2, Mcf2l, Plekhg5, Tiam1

GO:0000976	transcription regulatory region sequence-specific DNA binding	6h	4	Bcl11b, Egr4, Jun, Per1
GO:0001102	RNA polymerase II activating transcription factor binding	6h, 12h	2	Cited2, Jun
