# Supplementary material for: Glial responses during epileptogenesis in Mus musculus point to potential therapeutic targets
Source: PLoS One. 2018 Aug 16;13(8):e0201742. doi: 10.1371/journal.pone.0201742 (PMC6095496; doi:10.1371/journal.pone.0201742)
Supplement: S9 Table — All significantly changed genes at 12h were considered, and a threshold of p-value <0.05 was applied. (PDF) [file pone.0201742.s013.pdf]

Table S9: Significantly changed GO Molecular Functions (level 6) at 12 hours post KA treatment, using the "Mapping to ontologies (TRANSPATH®)" workflow. All significantly changed genes at 12h were considered, and a threshold of p-value <0.05 was applied.

Gene Ontology Category ID	GO-Molecular Function (level 6)	Time point(s) of enrichment	Number of significantly changed genes at 12h	Symbol of significantly changed genes at 12h
GO:0032555	purine ribonucleotide binding	12h, 24h	75	Abcc8, Ak5, Akt3, Arf2, Arl15, Arl4d, Atp13a5, Atp2b1, Atp2b2, Atrx, Cacna1b, Camk1d, Camk2a, Camk2b, Camkk2, Dclk1, Dclk3, Dgkh, Diras1, Dnm1, Dnm3, Epha3, Epha4, Eprs, Gbp2, Gem, Gucy1b3, Hspa12a, Hspa1a, Hspa1b, Hspa5, Hsph1, Itpka, Kalrn, Map2k3, Map3k6, Map4k4, Mapk4, Met, Morc4, Myo5b, Myo9b, Pak6, Pde1a, Pdk4, Pik3c3, Pim1, Pip4k2c, Pmvk, Prkar2a, Prkcc, Prkx, Ptk2b, Rab27b, Rab9b, Rasl11a, Rhoc, Rhoj, Ripk1, Rnd3, Rnf112, Rps6ka3, Rps6ka5, Rps6ka6, Rrad, Sept6, Sik2, Speg, Sphk1, Srr, Srxn1, Tgm2, Tlk2, Tnik, Trim23
GO:0046914	transition metal ion binding	6h, 12h	71	Adamts1, Adamts9, Alox12b, Ar, Arhgef2, Arih2, Atrx, Bach1, Baz1a, Bcl11a, Bcl11b, Birc3, Car7, Ch25h, Cpn1, Cyba, Cybb, Cyld, Cyp4x1, Cyp7b1, Egr2, Fblim1, Fbxo41, Glis3, Gucy1b3, Hmox1, Klf5, Klf6, Lancl1, Lhx9, Limd2, Lmo1, Lonrf3, Morc4, Mt2, Mtfp1, Myo9b, Nos3, Nr1d2, Nr2c2, Nr3c2, Nr4a2, Pcgf5, Pdlim1, Pdlim4, Pdlim7, Pglyrp1, Pim1, Plagl1, Pnkd, Prkcc, Ptgs2, Rnf112, Rnf170, Rnf217, Rora, Rorb, Siah2, Tiparp, Tll1, Trim23, Zcchc7, Zdhhc18, Zeb2, Zfp239, Zfp295, Zfp36, Zfp462, Zfp516, Zkscan4, Zmiz1

GO:0005509	calcium ion binding	12h, 24h	43	Anxa2, Anxa3, Atp2b2, Camkk2, Capn3, Cd93, Cdh8, Edem1, Efhd2, Fbn1, Grin1, Hpca, Hspa5, Kcnip2, Kcnip4, Lrp8, Matn2, Megf9, Ncald, Nell1, Nrxn1, Pcdh19, Pcdh8, Pclo, Pitpnm2, Plce1, Ppp3ca, Pros1, Pvalb, Rasgrp1, Ryr2, S100a10, S100a11, Slit1, Smoc2, Srr, Stat3, Syt7, Tesc, Tgm2, Thbs1, Tll1, Tpm4
GO:0004672	protein kinase activity	12h, 24h	38	Akt3, Bdnf, Camk1d, Camk2a, Camk2b, Camkk2, Ccl2, Cdkn1a, Crim1, Dclk1, Dclk3, Eng, Epha3, Epha4, Itpka, Kalrn, Kcnh3, Map2k3, Map3k6, Map4k4, Mapk4, Met, Pak6, Pdk4, Pik3c3, Pim1, Prkcc, Prkx, Ptk2b, Ripk1, Rps6ka3, Rps6ka5, Rps6ka6, Sik2, Speg, Stat3, Tlk2, Tnik
GO:0005216	ion channel activity	12h, 24h	32	Abcc8, Ano3, Cacna1b, Cacna1d, Cacna1h, Cybb, Fxyd5, Fxyd7, Gabra5, Gabrg2, Gria1, Gria3, Grin1, Kcna2, Kcnab1, Kcnc1, Kcnc2, Kcnf1, Kcnh3, Kcnip2, Kcnip4, Kcnj3, Kcnj9, Kcnq5, Kctd4, Nalcn, Ryr2, Scn1a, Scn2a1, Scn3b, Shroom2, Slc24a2
GO:0008324	cation transmembrane transporter activity	12h, 24h	31	Abcc8, Atp2b1, Atp2b2, Cacna1b, Cacna1d, Cacna1h, Grin1, Kcna2, Kcnab1, Kcnc1, Kcnc2, Kcnf1, Kcnh3, Kcnip2, Kcnip4, Kcnj3, Kcnj9, Kcnq5, Kctd4, Nalcn, Ryr2, Scn1a, Scn2a1, Scn3b, Shroom2, Slc10a6, Slc12a5, Slc24a2, Slc39a10, Slc5a3, Slc6a8
GO:0022836	gated channel activity	6h, 12h, 24h	28	Abcc8, Cacna1b, Cacna1d, Cacna1h, Cybb, Gabra5, Gabrg2, Gria1, Gria3, Grin1, Kcna2, Kcnab1, Kcnc1, Kcnc2, Kcnf1, Kcnh3, Kcnip2, Kcnip4, Kcnj3, Kcnj9, Kcnq5, Kctd4, Nalcn, Ryr2, Scn1a, Scn2a1, Scn3b, Shroom2
GO:0019901	protein kinase binding	12h, 24h	25	Akap5, Akt3, Camk2n1, Cd44, Cyld, Errfi1, Gria1, Hspb1, Map2k3, Mapk4, Mapk8ip2, Mef2a, Msn, Nbea, Nell1, Nfatc1, Pik3r1, Plek, Prkar2a, Ptk2b, Rps6ka3, Ryr2, Stat3, Vim, Zfp36

GO:0019001	guanyl nucleotide binding	12h, 24h	21	Arf2, Arl15, Arl4d, Diras1, Dnm1, Dnm3, Gbp2, Gem, Gucy1b3, Pde1a, Rab27b, Rab9b, Rasl11a, Rhoc, Rhoj, Rnd3, Rnf112, Rrad, Sept6, Tgm2, Trim23
GO:0044212	transcription regulatory region DNA binding	6h, 12h	17	Ar, Bcl11b, Bmyc, Cebpd, Dbp, Egr2, Fos, Hmgb2, Jun, Mef2a, Mterfd3, Myc, Ncor1, Nfatc1, Nfil3, Stat3, Tal1
GO:0003690	double-stranded DNA binding	6h, 12h	10	Fos, Fosb, Fosl2, Foxk1, Hmgb2, Jun, Junb, Klf6, Myc, Nr3c2
GO:0000287	magnesium ion binding	12h	10	Foxk1, Gem, Map3k6, Rps6ka3, Rps6ka5, Rps6ka6, Sik2, Sphk1, Srr, Tesc
GO:0005088	Ras guanyl-nucleotide exchange factor activity	6h, 12h	9	Arhgef2, Arhgef25, Arhgef4, Kalrn, Kifap3, Mcf2l, Rasgef1b, Rasgrf2, Rasgrp1
GO:0008201	heparin binding	6h, 12h, 24h	9	Adamts1, Ccl2, Ccl7, Col25a1, Cyr61, Hbegf, Nell1, Smoc2, Thbs1
GO:0035257	nuclear hormone receptor binding	12h	8	Ar, Grm1, Hmga1, Med13, Ncor1, Nrip1, Pik3r1, Stat3
GO:0031625	ubiquitin protein ligase binding	12h	8	Egr2, Eif4e2, Hspa1a, Hspa1b, Hspa5, Nfkbia, Pik3r1, Prkar2a
GO:0042379	chemokine receptor binding	12h	7	Ccl11, Ccl2, Ccl4, Ccl6, Ccl7, Ccl9, Stat3
GO:0008009	chemokine activity	12h	6	Ccl11, Ccl2, Ccl4, Ccl6, Ccl7, Ccl9
GO:0008375	acetylglucosaminyltransferase activity	12h	6	B3gnt1, B3gnt5, Extl1, Extl2, Gcnt2, Mgat5b
GO:0004869	cysteine-type endopeptidase inhibitor activity	12h	5	Bcl2a1d, Cstb, Ctla2b, Hspa5, Rps6ka3
GO:0004983	neuropeptide Y receptor activity	12h	4	Gpr83, Npy, Npy2r, Npy5r
GO:0005184	neuropeptide hormone activity	12h	4	Cartpt, Crh, Npy, Pdyn
GO:0005100	Rho GTPase activator activity	12h	4	Arhgap15, Errfi1, Jun, Myo9b

GO:0046965	retinoid X receptor binding	12h	3	Hmga1, Ncor1, Nrip1
GO:0004970	ionotropic glutamate receptor activity	12h	3	Gria1, Gria3, Grin1
GO:0031420	alkali metal ion binding	12h	3	Capn3, Scn1a, Scn2a1
GO:0001105	RNA polymerase II transcription coactivator activity	12h	3	Cited2, Mef2a, Psip1
GO:0001102	RNA polymerase II activating transcription factor binding	12h	3	Cited2, Egr2, Jun
GO:0005154	epidermal growth factor receptor binding	12h	3	Areg, Cd44, Hbegf
GO:0001639	PLC activating G-protein coupled glutamate receptor activity	12h	2	Grm1, Grm5
GO:0035256	G-protein coupled glutamate receptor binding	12h	2	Homer1, Homer2
GO:0004985	opioid receptor activity	12h	2	Opcml, Oprl1
GO:0008140	cAMP response element binding protein binding	12h	2	Crem, Crtc1
GO:0016175	superoxide-generating NADPH oxidase activity	12h	2	Cyba, Cybb
GO:0042834	peptidoglycan binding	12h	2	Pglyrp1, Tlr2
GO:0017040	ceramidase activity	12h	2	Gba2, Lct
