# Supplementary material for: Glial responses during epileptogenesis in Mus musculus point to potential therapeutic targets
Source: PLoS One. 2018 Aug 16;13(8):e0201742. doi: 10.1371/journal.pone.0201742 (PMC6095496; doi:10.1371/journal.pone.0201742)
Supplement: S10 Table — All significantly changed genes at 6h were considered, and a threshold of p-value <0.05 was applied. (PDF) [file pone.0201742.s014.pdf]

Table S10: Significantly changed GO Cellular Components (level 5) at 6 hours post KA treatment, using the "Mapping to ontologies (TRANSPATH®)" workflow. All significantly changed genes at 6h were considered, and a threshold of p-value <0.05 was applied.

Gene Ontology Category ID	GO-Cellular Component (level 5)	Time point(s) of enrichment	Number of significantly changed genes at 6h	Symbol of significantly changed genes at 6h
GO:0005634	nucleus	6h, 12h, 24h	81	2310046A06Rik, 4933421E11Rik, Arih2, Atf7ip (ENSMUSG00000053935), Bach2, Bag4, Baz1a, Bcl11b, Cacna1h, Camkk1, Casc3, Cdkn1a, Cited2, Crem, Csrnp1, Dhx33, Dusp1, Dusp5, E2f3, Egr4, Ehd3, Ell2, Elp2, Errfi1, Fos, Fosb, Foxk1, Foxp1, Gadd45b, Gadd45g, Hcfc2, Ifrd1, Jun, Junb, Kdm4b, Kdm6b, Map3k5, Mbd5, Narf, Neurod2, Neurod6, Nfkbiz, Npas4, Nr3c2, Nr4a1, Nr4a2, Nr4a3, Nrip2, Per1, Plagl1, Prkx, Ptbp2, Ptgs2, Pvr, Qsox2, Rcan1, Rcor2, Rdh10, Rgs2, Rilpl1, Ripply2, Rorb, Sap18, Sap30, Selp, Senp7, Setd1b, Siah2, Smad7, Srsf12, Tfdp2, Tinf2, Tnpo2, Tspyl3, Zeb2, Zfp12, Zfp128, Zfp292, Zfp36, Zfp462, Zwint
GO:0044451	nucleoplasm part	6h	20	2310046A06Rik, Atf7ip (ENSMUSG00000053935), Casc3, Crem, E2f3, Ell2, Elp2, Fos, Foxk1, Foxp1, Jun, Npas4, Nr4a1, Nr4a3, Rcor2, Sap18, Sap30, Setd1b, Smad7, Tfdp2
GO:0016023	cytoplasmic membrane-bounded vesicle	6h, 12h, 24h	13	1190002N15Rik, Anxa2, Arc, Bdnf, Clvs2, Ehd3, Gpnmb, Pcsk1, Pcsk2, Scg2, Srgn, Stxbp5, Syt4
GO:0034703	cation channel complex	6h, 12h, 24h	7	Cacna1h, Kcnf1, Kcnip2, Kcns2, Kcnv1, Kctd4, Kctd6

GO:0008076	voltage-gated potassium channel complex	6h, 12h, 24h	6	Kcnf1, Kcnip2, Kcns2, Kcnv1, Kctd4, Kctd6
GO:0031981	nuclear lumen	6h, 12h	2	2310046A06Rik, Narf
