# Supplementary material for: Glial responses during epileptogenesis in Mus musculus point to potential therapeutic targets
Source: PLoS One. 2018 Aug 16;13(8):e0201742. doi: 10.1371/journal.pone.0201742 (PMC6095496; doi:10.1371/journal.pone.0201742)
Supplement: S11 Table — All significantly changed genes at 12h were considered, and a threshold of p-value <0.05 was applied. (PDF) [file pone.0201742.s015.pdf]

Table S11: Significantly changed GO Cellular Components (level 5) at 12 hours post KA treatment, using the "Mapping to ontologies (TRANSPATH®)" workflow. All significantly changed genes at 12h were considered, and a threshold of p-value <0.05 was applied.

Gene Ontology Category ID	GO-Cellular Component (level 5)	Time point(s) of enrichment	Number of significantly changed genes at 12h	Symbol of significantly changed genes at 12h
GO:0005634	nucleus	6h, 12h, 24h	198	2310046A06Rik, 2900092E17Rik, Abt1, Aff1, Ahnak, Akt3, Anks1b, Ar, Areg, Arhgap39, Arih2, Arl4d, Atrx, Bach1, Baz1a, Bcl11a, Bcl11b, Bcl3, Birc3, Bmyc, Cacna1h, Camk1d, Capg, Capn3, Ccdc86, Cd44, Cdkn1a, Cdyl, Cebpd, Cenpw, Ciapin1, Cited2, Crem, Crtc1, Csrnp1, Cstb, Dbp, Dclk3, Dgkh, Dnajb1, Dusp1, Eaf1, Eaf2, Egr2, Eif2c2, Eif4ebp1, Ell2, Eng, Errfi1, Etv3, Evc2, Fam120b, Fgf11, Fos, Fosb, Fosl1, Fosl2, Foxk1, Gadd45b, Gadd45g, Glis3, Gltscr2, Grm1, Hist1h1c, Hist1h3f, Hist1h4a, Hmga1, Hmgb2, Hmox1, Hn1l, Hnmt, Hspa1a, Hspa1b, Hspa5, Hspb1, Hsph1, Id2, Ifrd1, Igf2bp2, Il16, Jarid2, Jun, Junb, Kdm6b, Klf5, Klf6, Lancl1, Larp6, Lgals3, Lhx9, Lmna, Lmo1, Lrrfip1, Maff, Magoh, Mapk4, Mbd2, Mbd5, Mcl1, Med13, Mef2a, Msn, Myc, Naf1, Narf, Nat14, Ncor1, Ndufaf3, Nedd9, Neurod6, Nfatc1, Nfe2l2, Nfil3, Nfkbia, Nfkbiz, Nos3, Npas4, Nr1d2, Nr2c2, Nr3c2, Nr4a2, Nrip1, Nrip2, Pak6, Pcgf5, Pdcd4, Pde1a, Pde4b, Phlda1, Pik3r1, Pim1, Pip4k2c, Pkp2, Plagl1, Plin2, Pnkd, Pnma2, Pop4, Ppp3ca, Prkcc, Prkx, Psip1, Ptgs2, Ptk2b, Pvr, Rabgap1l, Rasl11a, Rbfox1, Rbfox3, Rcan1, Rdh10, [illegible]

GO:0016021	integral to membrane	12h	191	2200002K05Rik, A730017C20Rik, AI464131, AI593442, Abcc8, Adcyap1r1, Ano3, Areg, Art3, Asphd2, Atp13a5, Atp2b1, Atp2b2, B3gat1, B3gnt1, B3gnt5, Bai3, C3ar1, Cacna1b, Cacna1d, Cacna1h, Cadm2, Caly, Cav1, Cd44, Cd68, Cd93, Cdh8, Cend1, Ch25h, Chst12, Cldn22, Col25a1, Crim1, Cuzd1, Cybb, Cyyr1, D130043K22Rik, Dclk1, Disp2, Ecscr, Edem1, Elfn2, Elovl1, Emp1, Eng, Epha3, Epha4, Evc2, Extl1, Extl2, Faah, Faim2, Fam163b, Fam23a, Fam57b, Fndc3b, Fndc5, Fxyd5, Fxyd7, Gabra5, Gabrg2, Gba2, Gcnt2, Ggt7, Glt8d2, Golm1, Gp49a, Gpr12, Gpr155, Gpr19, Gpr83, Gramd1b, Gria1, Gria3, Grin1, Grm1, Grm5, Hbegf, Homer1, Hspa5, Htr1a, Icam1, Icam5, Ier3, Igsf8, Il13ra1, Il17ra, Il1rap, Kcna2, Kcnab1, Kcnc1, Kcnc2, Kcnf1, Kcnh3, Kcnj3, Kcnj9, Kcnq5, Kitl, Lancl1, Lct, Lilrb4, Lmbr1, Lrp8, Lrrc4b, Lrrn2, Ltbr, Mall, Mcl1, Megf9, Met, Mfsd4, Mfsd6, Mgat5b, Mpp2, Ms4a6d, Msr1, Mtfp1, Nalcn, Nat14, Nat8l, Nceh1, Ndst4, Nlgn3, Npy2r, Npy5r, Nrxn1, Nrxn3, Opcml, Oprl1, Orai2, Orai3, Osmr, Paqr7, Pcdh19, Pcdh8, Pcnx, Pgbd5, Pitpnm2, Pkp2, Plxna1, Podxl, Prrt2, Prrt3, Ptger2, Pvr, Rdh10, Rnf170, Rnf217, Ryr2,

GO:0005829	cytosol	12h, 24h	99	Acot3, Ak5, Akap5, Akt3, Aldh1a3, Ampd3, Arhgap12, Arhgap15, Arhgap20, Arhgap39, Arhgef2, Arhgef25, Arhgef4, Atp2b2, Bach1, Bag3, Camk2a, Camk2b, Capn3, Cav1, Cdkn1a, Ch25h, Chac1, Chn1, Cyld, Dnajc6, Dnm3, Eif2c2, Eif4e2, Eif4ebp1, Eprs, Errfi1, Etf1, Fabp3, Fblim1, Fosl1, Gbp2, Gmpr, Gria1, Gucy1b3, Hmga1, Hmox1, Hspa1a, Hspa1b, Hspb1, Id2, Igf2bp2, Jun, Kalrn, Kifap3, Magoh, Map2k3, Mcf2l, Msr1, Myo9b, Nbea, Ncald, Nfe2l2, Nfkbia, Nmt1, Nos3, Odc1, Pdcd4, Pde1a, Pde4b, Pik3c3, Pik3r1, Plce1, Plek, Pmvk, Ppp3ca, Prkar2a, Prkcc, Psip1, Ptk2b, Ptpn12, Rasgrf2, Rasgrp1, Rgs2, Rgs4, Rhoc, Rhoj, Rilpl1, Ripk1, Rps6ka3, Rps6ka6, Sat1, Siah2, Socs3, Sphk1, Srxn1, Stat3, Sult2b1, Tgm2, Tpm4, Tubgcp2, Vim, Zfp36, Zwint
GO:0005624	membrane fraction	12h, 24h	64	Anxa2, Atp2b2, Bag3, Cacna1b, Camk2n1, Cav1, Cd68, Cntn1, Cyp4x1, Cyp7b1, Dsp, Emp1, Eng, Fos, Fosl1, Gba2, Gcnt2, Gria1, Gria3, Grin1, Grm1, Grm5, Hmox1, Hnmt, Homer1, Kcnip4, Lct, Lin7b, Lrp8, Met, Mpp2, Myo5b, Nat8l, Ncald, Nceh1, Npy2r, Nrxn1, Pik3r1, Plce1, Plek, Plxna1, Ppp3ca, Prkar2a, Prkcc, Ptgs2, Rasgrp1, Rdh10, Rgs4, Rhoc, Ryr2, Samd4, Scn1a, Scn2a1, Siah2, Slc16a1, Slc24a2, Sphk1, Sstr2, Synpr, Syt7, Tmem59l, Tspan13, Vamp1, Zwint
GO:0031226	intrinsic to plasma membrane	12h, 24h	58	Adcyap1r1, Art3, Atp2b1, C3ar1, Cacna1d, Caly, Cav1, Cd44, Cdh8, Col25a1, Cybb, Dclk1, Epha3, Epha4, Gabra5, Gabrg2, Gfra1, Ggt7, Golm1, Gpr12, Gpr19, Grin1, Grm1, Grm5, Hbegf, Homer1, Htr1a, Icam1, Icam5, Il17ra, Il1rap, Lancl1, Lct, Met, Mpp2, Msr1, Nlgn3, Npy2r, Npy5r, Nrxn1, Nrxn3, Opcml, Oprl1, Osmr, Pcdh8, Podxl, Ptger2, S1pr3, Slc14a1, Slc5a3, Slc6a8, Sstr2, Tlr2, Tm4sf1, Tnfrsf1a, Tpbg, Tspan13, Vamp1

GO:0005730	nucleolus	12h	54	Abt1, Akt3, Arl4d, Bach1, Bmyc, Capg, Ccdc86, Ciapin1, Crtc1, Csrnp1, Cstb, Dnajb1, Dtd1, Eaf2, Ell2, Eng, Fos, Fosl2, Foxk1, Gltscr2, Hmgb2, Hmox1, Hnmt, Hsph1, Id2, Junb, Klf5, Larp6, Lmo1, Mbd2, Msn, Myc, Nos3, Nr2c2, Pdcd4, Phlda1, Pip4k2c, Pnma2, Podxl, Pop4, Ppp3ca, Psip1, Ptk2b, Pvr, Rasl11a, Rgs2, Rilpl1, Sap18, Stat3, Tnik, Wwtr1, Ypel4, Zcchc7, Zeb2
GO:0005654	nucleoplasm	12h	35	Akt3, Ar, Bmyc, Camk2a, Camk2b, Cdkn1a, Dusp1, Eaf2, Fos, Hmga1, Hmgb2, Jun, Lmna, Maff, Magoh, Map2k3, Mcl1, Med13, Mef2a, Myc, Ncor1, Nr1d2, Nr2c2, Nr3c2, Nr4a2, Psip1, Rora, Rorb, Rps6ka3, Rps6ka5, Rps6ka6, Snrpa1, Srsf12, Stat3, Tinf2
GO:0016023	cytoplasmic membrane-bounded vesicle	6h, 12h, 24h	29	1190002N15Rik, Anxa2, Anxa3, Arc, Bdnf, Capg, Cartpt, Cd93, Cuzd1, Cyba, Cybb, Dnm3, Grin1, Hspa5, Mall, Nlgn3, Nrxn1, Pclo, Pcsk1, Pcsk2, Rab27b, Scg2, Sept6, Sphk1, Srgn, Synpr, Syt17, Syt7, Thbs1
GO:0030425	dendrite	12h, 24h	27	Anxa3, Ar, Atp2b2, Cacna1b, Cacna1h, Camk2a, Camk2n1, Cnn3, Cyba, Cybb, Epha4, Gabra5, Gria1, Gria3, Grin1, Grm1, Homer1, Homer2, Kcnip2, Kcnip4, Lrp8, Met, Pcdh8, Pcsk1, Prkcc, Synpo, Zwint
GO:0048471	perinuclear region of cytoplasm	12h, 24h	23	Akap5, Anxa2, Bcl3, Cav1, Cyld, Dnm3, Hmgb2, Hspa1a, Hspa1b, Hspa5, Kalrn, Lmna, Myo5b, Myo9b, Ncor1, Pcsk1, Pde4b, Prkar2a, Prkcc, Ptk2b, Spp1, Tlk2, Tnik
GO:0030424	axon	12h, 24h	17	Anxa3, Ar, Cadm2, Epha4, Gabrg2, Grm1, Myc, Ptk2b, Pvalb, Rhoc, Scn1a, Scn2a1, Sphk1, Stmn4, Synpo, Tnfrsf1a, Vim
GO:0030659	cytoplasmic vesicle membrane	12h, 24h	17	Abcc8, Anxa3, Caly, Camk2a, Camk2b, Cav1, Cltb, Cuzd1, Gria1, Gria3, Msn, Nos3, Phlda1, Rab27b, Synpr, Syt7, Vamp1
GO:0034703	cation channel complex	6h, 12h, 24h	15	Cacna1b, Cacna1d, Cacna1h, Kcna2, Kcnc1, Kcnc2, Kcnf1, Kcnip2, Kcnj3, Kcnq5, Kctd4, Ryr2, Scn1a, Scn2a1, Scn3b

GO:0000785	chromatin	12h	15	Ar, Atrx, Cited2, Hist1h3e, Hmga1, Id2, Jun, Junb, Mbd2, Mef2a, Ncor1, Nfatc1, Nfe2l2, Psip1, Tal1
GO:0044309	neuron spine	12h, 24h	13	Akap5, Anks1b, Arc, Cnn3, Dnm3, Gria1, Gria3, Grin1, Grm1, Grm5, Myo5b, Prosapip1, Synpo
GO:0016324	apical plasma membrane	12h, 24h	13	Amotl1, Atp2b1, Atp2b2, Cacna1d, Cav1, Cd44, Cyba, Hspa1b, Lct, Msn, Podxl, Rab27b, Shroom2
GO:0008076	voltage-gated potassium channel complex	6h, 12h, 24h	8	Kcna2, Kcnc1, Kcnc2, Kcnf1, Kcnip2, Kcnj3, Kcnq5, Kctd4
GO:0031256	leading edge membrane	12h	8	Akap5, Arhgef2, Arhgef4, Gabrg2, Grin1, Kcnc1, Plek, Slc12a5
GO:0005923	tight junction	12h	7	Adcyap1r1, Amotl1, Arhgef2, Cldn22, Lin7b, Shroom2, Synpo
GO:0000118	histone deacetylase complex	12h	6	Mbd2, Ncor1, Nrip1, Sap18, Sap30, Tal1
GO:0005901	caveola	12h, 24h	6	Adcyap1r1, Cav1, Hmox1, Lrp8, Nos3, Ptgs2
GO:0031227	intrinsic to endoplasmic reticulum membrane	12h	6	Asphd2, Edem1, Elovl1, Extl1, Extl2, Hspa5
GO:0030315	T-tubule	12h, 24h	5	Capn3, Kcnj3, Prkar2a, Scn1a, Scn2a1
GO:0032589	neuron projection membrane	12h, 24h	5	Akap5, Gabrg2, Grin1, Kcnc1, Slc12a5
GO:0060205	cytoplasmic membrane-bounded vesicle lumen	12h, 24h	5	Pros1, Serpine1, Srgn, Thbs1, Timp1
GO:0044298	cell body membrane	12h	3	Cadm2, Gabra5, Kcnc1
GO:0001518	voltage-gated sodium channel complex	12h, 24h	3	Scn1a, Scn2a1, Scn3b
GO:0005891	voltage-gated calcium channel complex	12h	3	Cacna1b, Cacna1d, Cacna1h
GO:0031981	nuclear lumen	6h, 12h	2	2310046A06Rik, Narf
GO:0043194	initial segment	12h	2	Scn1a, Scn2a1
GO:0005638	lamin filament	12h	2	Lmna, Narf

GO:0030130	clathrin coat of trans-Golgi network vesicle	12h	2	Cltb, Ncald
GO:0030935	sheet-forming collagen	12h	2	Col4a1, Col4a2
GO:0005652	nuclear lamina	12h	2	Lmna, Narf
GO:0033268	node of Ranvier	12h, 24h	2	Scn1a, Scn2a1
GO:0031527	filopodium membrane	12h, 24h	2	Akap5, Msn
