# Supplementary material for: Glial responses during epileptogenesis in Mus musculus point to potential therapeutic targets
Source: PLoS One. 2018 Aug 16;13(8):e0201742. doi: 10.1371/journal.pone.0201742 (PMC6095496; doi:10.1371/journal.pone.0201742)
Supplement: S12 Table — All significantly changed genes at 24h were considered, and a threshold of p-value <0.05 was applied. (PDF) [file pone.0201742.s016.pdf]

Table S12: Significantly changed GO Cellular Components (level 5) at 24 hours post KA treatment, using the "Mapping to ontologies (TRANSPATH®)" workflow. All significantly changed genes at 24h were considered, and a threshold of p-value <0.05 was applied.

Gene Ontology Category ID	GO-Cellular Component (level 5)	Time point(s) of enrichment	Number of significantly changed genes at 24h	Symbol of significantly changed genes at 24h
GO:0005829	cytosol	12h, 24h	52	Agtpbp1, Akap5, Ampd3, Arhgef4, Bag3, Cald1, Capn3, Casp8, Cdk14, Cdkn1a, Chn1, Clic4, Ctnna1, Dnm3, Dock2, Eif2ak2, Eif4ebp1, Eprs, Etf1, Fblim1, Fgfr1op, Fkbp1b, Gfap, Gucy1b3, Hspa1b, Hspb1, Id2, Kif18a, Msr1, Myd88, Nedd1, Nfkbia, Nmt1, Odc1, Papss2, Pdcd6ip, Pla2g4a, Plek, Prkar2a, Ptpn12, Rars, Rasgrp1, Rfwd2, Rgs2, Rpl12, Srxn1, Stard8, Tgm2, Tub, Uck2, Vim, Yes1
GO:0031226	intrinsic to plasma membrane	12h, 24h	32	Atp8b1, C3ar1, Caly, Cd44, Cd9, Cdh4, Cdh8, Col25a1, Cybb, Dclk1, Efna5, Efna5 (ENSMUSG00000090425), Epha3, Epha4, Fcgr2b, Gpnmb, Grm1, Grm8, Hbegf, Htr1a, Icam1, Itgav, Msr1, Osmr, Pcdh11x, Pcdh8, S1pr3, Sdc1, Slc6a8, Slc7a1, Tlr1, Tpbg
GO:0005624	membrane fraction	12h, 24h	26	Anxa2, Arf6, Atp8b1, Bag3, Cald1, Ebna1bp2, Emp1, Eng, Fkbp1b, Fos, Gfap, Gria3, Grm1, Lamp2, Lin7b, Lrrk2, Pla2g4a, Plek, Prkar2a, Rasgrp1, Rdh10, Samd4, Scn2a1, Scn8a (ENSMUSG00000023033), Uck2, Yes1
GO:0048471	perinuclear region of cytoplasm	12h, 24h	12	Akap5, Anxa2, Clic4, Dnm3, Fgfr1op, Hspa1b, Kcnma1, Pla2g4a, Prkar2a, Rnf128, Spp1, Syt4
GO:0030425	dendrite	12h, 24h	11	Cald1, Cybb, Epha4, Gria3, Grm1, Kcnma1, Lrrk2, Pcdh8, Scn8a (ENSMUSG00000023033), Synpo, Wfs1
GO:0030659	cytoplasmic vesicle membrane	12h, 24h	10	Cadps2, Caly, Cd9, Clic4, Gria3, Lamp2, Msn, Syt4, Tlr1, Zdhhc17

GO:0030424	axon	12h, 24h	8	Calca, Epha4, Grm1, Lrrk2, Lrrtm1, Scn2a1, Synpo, Vim
GO:0016324	apical plasma membrane	12h, 24h	8	Amotl1, Atp8b1, Cd44, Cd9, Hspa1b, Kcnma1, Msn, Shroom3
GO:0044309	neuron spine	12h, 24h	6	Akap5, Cald1, Dnm3, Gria3, Grm1, Synpo
GO:0060205	cytoplasmic membrane-bounded vesicle lumen	12h, 24h	5	Pros1, Serpine1, Srgn, Thbs1, Timp1
GO:0005901	caveola	12h, 24h	4	Efna5, Efna5 (ENSMUSG00000090425), Kcnma1, Kif18a
GO:0031527	filopodium membrane	12h, 24h	3	Akap5, Arf6, Msn
GO:0001518	voltage-gated sodium channel complex	12h, 24h	3	Scn2a1, Scn3b, Scn8a (ENSMUSG00000023033)
GO:0030315	T-tubule	12h, 24h	3	Capn3, Prkar2a, Scn2a1
GO:0033268	node of Ranvier	12h, 24h	2	Scn2a1, Scn8a (ENSMUSG00000023033)
GO:0005856	cytoskeleton	24h	31	Arpc1b, Cald1, Casp8, Clic4, Ctnna1, Dnm3, Dock2, Fblim1, Fcgr2b, Fgfr1op, Fhod3, Frmd6, Hspb1, Kif18a, Micall1, Msn, Nedd1, Nedd9, Nek6, Nmt1, Nufip1, Parp3, Pdcd6ip, Rai14, Sept6, Shroom3, Svil, Synpo, Tubb6, Vim, Yes1
GO:0005815	microtubule organizing center	24h	14	Casp8, Clic4, Fgfr1op, Il1rn, Kif18a, Naf1, Nedd1, Nek6, Parp3, Pdcd6ip, Prkar2a, Rfwd2, Yes1, Zfp110
GO:0009897	external side of plasma membrane	24h	8	Cd44, Cd9, Eng, Fcgr2b, Icam1, Itgav, Kcnma1, Thbs1
GO:0005925	focal adhesion	24h	7	Cald1, Fblim1, Msn, Nedd9, Rfwd2, Sdc1, Stard8
GO:0016323	basolateral plasma membrane	24h	6	Akap5, Cd44, Erbb2ip, Hspa1b, Lin7b, Msn
GO:0019897	extrinsic to plasma membrane	24h	5	Anxa2, Ctnna1, Gng2, Gng4, S100a10
GO:0016363	nuclear matrix	24h	4	Clic4, Nufip1, Sfpq, Vim
GO:0009898	internal side of plasma membrane	24h	3	Gem, Msn, Rgs2

GO:0005634	nucleus	6h, 12h, 24h	99	Adam12, Aff1, Agtpbp1, Ahnak, Arid5b, Baz1a, Bcl11b, Birc3, Calca, Capn2, Capn3, Casp8, Cblb, Cd44, Cdk14, Cdkn1a, Cdyl, Clic4, Cstb, Dclk3, Ddx39, Dock2, Dyrk3, Eaf1, Ebna1bp2, Eif2ak2, Eif4ebp1, Elk3, Ell2, Eng, Erbb2ip, Etv3, Fam120b, Fgfr1op, Fhod3, Fos, Fosl2, Gadd45b, Gadd45g, Gcc2, Grm1, H3f3b, Hivep3, Hmgn1, Hn1l, Hspa1b, Hspb1, Hspb3, Hsph1, Id2, Ifrd1, Igfbp3, Il1rn, Kif18a, Klf4, Lgals1, Maff, Mbd2, Mcl1, Med13, Msn, Mybbp1a, Naf1, Nedd9, Nek6, Neurod6, Nfkbia, Nicn1, Nmi, Npas4, Nufip1, Parp3, Pla2g4a, Pvr, Rars, Rdh10, Rfwd2, Rgs2, Rpl12, Rps27l, Rps6ka5, Sap30, Sfpq, Sin3b, Snord22, Snrpa1, Svil, Tgif1, Trdmt1, Trib1, Tub, Utp14b, Vim, Wwtr1, Yy2, Zfp110, Zfp536, Zfp593, Zmiz1
GO:0016023	cytoplasmic membrane-bounded vesicle	6h, 12h, 24h	23	Anxa2, Bgn, Cadps2, Crispld2, Ctnna1, Cybb, Dnm3, Gcg, Gpnmb, Itgav, Lrrk2, Lyz2, Mall, Pcsk2, Pdcd6ip, Pla2g4a, Scg2, Scn8a (ENSMUSG00000023033), Sept6, Srgn, Syt4, Thbs1, Tlr1
GO:0034703	cation channel complex	6h, 12h, 24h	9	Fkbp1b, Kcnma1, Kcnq3, Kcnq5, Kcnt2, Kctd4, Scn2a1, Scn3b, Scn8a (ENSMUSG00000023033)
GO:0008076	voltage-gated potassium channel complex	6h, 12h, 24h	5	Kcnma1, Kcnq3, Kcnq5, Kcnt2, Kctd4
