# Supplementary material for: Glial responses during epileptogenesis in Mus musculus point to potential therapeutic targets
Source: PLoS One. 2018 Aug 16;13(8):e0201742. doi: 10.1371/journal.pone.0201742 (PMC6095496; doi:10.1371/journal.pone.0201742)
Supplement: S13 Table — All significantly changed genes at 24h were considered, and a threshold of p-value <0.05 was applied. (PDF) [file pone.0201742.s017.pdf]

Table S13: Significantly changed GO Molecular Functions (level 6) at 24 hours post KA treatment, using the "Mapping to ontologies (TRANSPATH®)" workflow. All significantly changed genes at 24h were considered, and a threshold of p-value <0.05 was applied.

Gene Ontology Category ID	GO-Molecular Function (level 6)	Time point(s) of enrichment	Number of significantly changed genes at 24h	Symbol of significantly changed genes at 24h
GO:0032555	purine ribonucleotide binding	12h, 24h	37	Arf6, Atp8b1, Cdk14, Dclk1, Dclk3, Ddx39, Dnm3, Dock2, Dyrk3, Eif2ak2, Epha3, Epha4, Eprs, Gem, Gucy1b3, Hspa1b, Hsph1, Irgm1, Kcnt2, Kif18a, Lrrk2, Nek6, Pak3, Papss2, Prkar2a, Rars, Riok2, Rnd3, Rps6ka5, Scn8a (ENSMUSG00000023033), Sept6, Srxn1, Tgm2, Trib1, Tubb6, Uck2, Yes1
GO:0005509	calcium ion binding	12h, 24h	26	Acan, Anxa2, Capn2, Capn3, Cblb, Cdh4, Cdh8, Cgref1, Edem1, Egfl6, Fstl5, Hpca, Ltbp1, Pcdh11x, Pcdh19,
GO:0004672	protein kinase activity	12h, 24h	18	Cdk14, Cdkn1a, Dclk1, Dclk3, Dyrk3, Eif2ak2, Eng, Epha3, Epha4, Lrrk2, Ltbp1, Nek6, Pak3, Riok2, Rps6ka5, Tbrg4, Trib1, Yes1
GO:0008324	cation transmembrane transporter activity	12h, 24h	16	Agtpbp1, Fkbp1b, Kcnab1, Kcnma1, Kcnq3, Kcnq5, Kcnt2, Kctd4, Mmgt2, Scn2a1, Scn3b, Scn8a (ENSMUSG00000023033), Slc10a6, Slc6a8, Slc7a1, Zdhhc17
GO:0022836	gated channel activity	6h, 12h, 24h	13	Clic4, Cybb, Fkbp1b, Gria3, Kcnab1, Kcnma1, Kcnq3, Kcnq5, Kcnt2, Kctd4, Scn2a1, Scn3b, Scn8a (ENSMUSG00000023033)
GO:0019901	protein kinase binding	12h, 24h	13	Akap5, Cblb, Cd44, Fgfr1op, Hspb1, Itgav, Msn, Nek6, Plek, Prkar2a, Tgif1, Trib1, Vim
GO:0005216	ion channel activity	12h, 24h	13	Clic4, Cybb, Fkbp1b, Gria3, Kcnab1, Kcnma1, Kcnq3, Kcnq5, Kcnt2, Kctd4, Scn2a1, Scn3b, Scn8a (ENSMUSG00000023033)
GO:0019001	guanyl nucleotide binding	12h, 24h	11	Arf6, Dnm3, Dock2, Gem, Gucy1b3, Irgm1, Lrrk2, Rnd3, Sept6, Tgm2, Tubb6

GO:0008201	heparin binding	6h, 12h, 24h	7	Col25a1, Crispld2, Cyr61, Gpnmb, Hbegf, Thbs1, Thbs4
GO:0008234	cysteine-type peptidase activity	24h	6	Capn2, Capn3, Casp8, Ctla2a, Ctla2b, Ctsc
GO:0031625	ubiquitin protein ligase binding	12h, 24h	5	Hspa1b, Nfkbia, Prkar2a, Trib1, Wfs1
GO:0031420	alkali metal ion binding	12h, 24h	3	Capn3, Scn2a1, Scn8a (ENSMUSG00000023033)
GO:0005154	epidermal growth factor receptor binding	12h, 24h	3	Cd44, Hbegf, Yes1
GO:0005184	neuropeptide hormone activity	12h, 24h	3	Calca, Crh, Npy
GO:0005534	galactose binding	24h	2	Eng, Lgals1
GO:0031994	insulin-like growth factor I binding	24h	2	Igfbp3, Itgav
GO:0003796	lysozyme activity	24h	2	Lyz1, Lyz2
GO:0004622	lysophospholipase activity	24h	2	Mgll, Pla2g4a
GO:0005149	interleukin-1 receptor binding	24h	2	Il1rn, Myd88
GO:0004033	aldo-keto reductase (NADP) activity	24h	2	Akr1b8, Rdh10
GO:0004675	transmembrane receptor protein serine/threonine kinase activity	24h	2	Eng, Ltbp1
GO:0005540	hyaluronic acid binding	24h	2	Acan, Cd44
GO:0030291	protein serine/threonine kinase inhibitor activity	24h	2	Cdkn1a, Hspb1
