# Supplementary material for: Glial responses during epileptogenesis in Mus musculus point to potential therapeutic targets
Source: PLoS One. 2018 Aug 16;13(8):e0201742. doi: 10.1371/journal.pone.0201742 (PMC6095496; doi:10.1371/journal.pone.0201742)
Supplement: S14 Table — The analysis was performed using the "Master regulators in networks (TRANSPATH®)" workflow, for all the significantly changed transcripts at 24h, applying the thresholds FDR <0.05, Score >0.2 and Z-Score >1. The predicted upstream regulators were filtered to exclude those that did not present with statistically significant overexpression themselves at 12h or 24h. The number and symbol of their respective significantly overexpressed downstream gene targets at 24h is included. (n/c: not changed) (PDF) [file pone.0201742.s018.pdf]

Table S14. Predicted statistically significant upstream regulators of the 24h gene expression changes. The analysis was performed using the "Master regulators in networks (TRANSPATH®)" workflow, for all the significantly changed transcripts at 24h, applying the thresholds FDR>0.05, Score>0.2 and Z-Score>1. The predicted upstream regulators were filtered to exclude those that did not present with statistically significant overexpression themselves at 12h or 24h. The number and symbol of their respective significantly overexpressed downstream gene targets at 24h is included. (n/c: not changed)

Upstream regulator	Fold Change at 12h	Fold Change at 24h	Z-Score	Number of regulated genes reached from dataset	Regulated genes reached from dataset
**Birc3**	2.47	2.68	3.39	38	Birc3, Capn2, Casp8, Ccl9, Cdkn1a, Cybb, Eif2ak2, Eif4ebp1, Elk3, Fcgr2b, Fos, Gfap, H3f3b, Hbegf, Hmgn1, Hspb1, Icam1, Id2, Il1rn, Itgav, Klf4, Lif, Msn, Mybbp1a, Myd88, Nedd9, Nfkbia, Pdcd6ip, Pla2g4a, Ptpn12, Rai14, Rnd3, S100a10,Tnc, Tubb6, Vim, Wwtr1, Yes1
**Calca**	n/c	5.55	8.26	38	Birc3, Capn2, Casp8, Ccl9, Cdkn1a, Cybb, Eif2ak2, Eif4ebp1, Elk3, Fcgr2b, Fos, Gfap, H3f3b, Hbegf, Hmgn1, Hspb1, Icam1, Id2, Il1rn, Itgav, Klf4, Lif, Msn, Mybbp1a, Myd88, Nedd9, Nfkbia, Pdcd6ip, Pla2g4a, Ptpn12, Rai14, Rnd3, S100a10, Tnc, Tubb6, Vim, Wwtr1, Yes1
**Capn2**	n/c	2.06	2.12	37	Arf6, Arpp21, Bag2, Birc3, C3ar1, Capn2, Casp8, Ccl9, Cdkn1a, Eif2ak2, Eif4ebp1, Elk3, Fcgr2b, Fos, Gem, Gfap, H3f3b, Hmgn1, Hspb1, Icam1, Il1rn, Klf4, Msn, Mybbp1a, Nedd9, Nfkbia, Pdcd6ip, Pla2g4a, Ptpn12, Rai14, Rnd3, Timp1, Tnc, Tubb6, Vim, Wwtr1, Yes1
**Cd9**	n/c	2.30	6.38	11	Capn2, Cd9, Eif4ebp1, Hbegf, Nedd9, Nfkbia, Pdcd6ip, Ptpn12, Sdc1, Tnc, Tubb6
**Cyba**	2.23	n/c	1.81	42	Bag2, Bgn, Birc3, C3ar1, Calca, Capn2, Casp8, Ccl9, Cdkn1a, Cybb, Eif2ak2, Eif4ebp1, Elk3, Eng, Fcgr2b, Fos, Fosl2, Gfap, H3f3a, H3f3b, Hmgn1, Hspb1, Icam1, Il1rn, Itgav, Klf4, Lif, Msn, Mybbp1a, Nedd9, Nfkbia, Pdcd6ip, Pla2g4a, Ptpn12, Rai14, Rnd3, Tgm2, Timp1, Tubb6, Vim, Wwtr1, Yes1
**Cybb**	2.92	4.75	4.52	15	Capn2, Cdkn1a, Cybb, Eif4ebp1, Elk3, Fos, H3f3b, Hmgn1, Klf4, Mybbp1a, Nedd9, Nfkbia, Pla2g4a, Ptpn12, Rai14
**Eif2ak2**	n/c	2.90	3.99	32	Bag2, Bgn, Capn2, Casp8, Ccl9, Cdkn1a, Eif2ak2, Eif4ebp1, Elk3, Fos, Gfap, H3f3b, Hmgn1, Hspb1, Icam1, Il1rn, Klf4, Lif, Msn, Mybbp1a, Nedd9, Nfkbia, Pdcd6ip, Pla2g4a, Ptpn12, Rai14, Rnd3, Timp1, Tubb6, Vim, Wwtr1, Yes1

**Gcg**	n/c	13.22	6.31	15	Capn2, Cdkn1a, Eif4ebp1, Elk3, Fos, Gcg, H3f3b, Hmgn1, Klf4, Mybbp1a, Nedd9, Nfkbia, Pla2g4a, Ptpn12, Rai14
**Hmgn1**	n/c	2.08	5.17	15	Bag2, Casp8, Cdkn1a, Eif2ak2, Eif4ebp1, Elk3, H3f3b, Hmgn1, Hspb1, Nedd9, Nfkbia, Pla2g4a, Ptpn12, Vim, Yes1
**Icam1**	4.10	4.32	4.56	36	Arf6, Bag2, C3ar1, Capn2, Ccl9, Cdkn1a, Cyr61, Eif4ebp1, Elk3, Eng, Fcgr2b, Fos, Gfap, H3f3b, Hmgn1, Hspb1, Icam1, Il1rn, Klf4, Msn, Mybbp1a, Myd88, Nedd9, Nfkbia, Pdcd6ip, Pla2g4a, Ptpn12, Rai14, Rnd3, Spp1, Timp1, Tnc, Tubb6, Vim, Wwtr1, Yes1
**Il11**	2.31	1.24	5.06	17	Birc3, Capn2, Cybb, Eif4ebp1, Elk3, Fos, Icam1, Il11, Mybbp1a, Nedd9, Nfkbia, Pdcd6ip, Pla2g4a, Ptpn12, Rai14, Tubb6, Yes1
**Itgav**	n/c	3.30	5.97	34	Bag2, C3ar1, Capn2, Casp8, Ccl9, Cdkn1a, Cyr61, Eif4ebp1, Elk3, Eng, Fcgr2b, Fos, H3f3b, Hmgn1, Hspb1, Icam1, Itgav, Klf4, Msn, Mybbp1a, Nedd9, Nfkbia, Pdcd6ip, Pla2g4a, Ptpn12, Rai14, Rnd3, Spp1, Tgm2, Tnc, Tubb6, Vim, Wwtr1, Yes1
**Lgals1**	n/c	2.73	6.58	20	Capn2, Cdkn1a, Eif4ebp1, Elk3, Fcgr2b, Fos, Gfap, H3f3b, Hmgn1, Klf4, Lgals1, Mybbp1a, Nedd9, Nfkbia, Pdcd6ip, Pla2g4a, Ptpn12, Rai14, Vim, Yes1
**Lif**	n/c	6.95	4.83	40	Bag2, Birc3, C3ar1, Capn2, Casp8, Ccl9, Cdkn1a, Cybb, Eif2ak2, Eif4ebp1, Elk3, Eng, Fcgr2b, Fos, Gfap, H3f3b, Hmgn1, Hspb1, Icam1, Il1rn, Itgav, Klf4, Lif, Msn, Mybbp1a, Nedd9, Nfkbia, Npy, Pak3, Pdcd6ip, Pla2g4a, Ptpn12, Rai14, Rnd3, Timp1, Tnc, Tubb6, Vim, Wwtr1, Yes1
**Myd88**	n/c	2.07	2.49	40	Bag2, Bgn, Birc3, Capn2, Casp8, Ccl9, Cdkn1a, Crh, Cybb, Eif2ak2, Eif4ebp1, Elk3, Fcgr2b, Fos, Gfap, H3f3b, Hmgn1, Hspb1, Icam1, Il1rn, Itgav, Klf4, Msn, Msr1, Mybbp1a, Myd88, Nedd9, Nfkbia, Nmi, Pdcd6ip, Pla2g4a, Ptpn12, Rai14, Rnd3, Tgm2, Timp1, Tubb6, Vim, Wwtr1, Yes1
**Nedd9**	4.16	4.70	3.83	41	Arf6, Arpp21, Birc3, C3ar1, Capn2, Casp8, Ccl9, Cdkn1a, Cybb, Eif2ak2, Eif4ebp1, Elk3, Eng, Fcgr2b, Fos, Gfap, H3f3b, Hbegf, Hmgn1, Hspb1, Icam1, Il1rn, Itgav, Klf4, Msn, Mybbp1a, Nedd9, Nfkbia, Pdcd6ip, Pla2g4a, Plek, Ptpn12, Rai14, Rnd3, S100a10, Timp1, Tnc, Tubb6, Vim, Wwtr1, Yes1

**Nek6**	n/c	2.17	4.01	38	Arpp21, Bag2, Bgn, C3ar1, Capn2, Casp8, Ccl9, Cdkn1a, Crh, Cybb, Eif2ak2, Eif4ebp1, Elk3, Fcgr2b, Fos, H3f3b, Hmgn1, Hspb1, Icam1, Il1rn, Itgav, Klf4, Msn, Mybbp1a, Nedd9, Nek6, Nfkbia, Odc1, Pdcd6ip, Pla2g4a, Ptpn12, Rai14, Tgm2, Timp1, Tubb6, Vim, Wwtr1, Yes1
**Nfkbia**	2.06	2.16	5.33		Ccl9, Cdkn1a, Cybb, Eif4ebp1, Elk3, Fos, H3f3b, Hmgn1, Hspb1, Icam1, Klf4, Mybbp1a, Nfkbia, Ptpn12, Rai14, Vim, Wwtr1
**Pak3**	n/c	2.37	3.51	40	Bag2, Bgn, Birc3, C3ar1, Calca, Capn2, Casp8, Cdkn1a, Eif2ak2, Eif4ebp1, Elk3, Erbb2ip, Fos, Fosl2, Gfap, H3f3b, Hmgn1, Hspb1, Icam1, Il1rn, Itgav, Klf4, Lif, Msn, Mybbp1a, Nedd9, Nfkbia, Odc1, Pak3, Pdcd6ip, Pla2g4a, Ptpn12, Rai14, Rnd3, Tgm2, Timp1, Tubb6, Vim, Wwtr1, Yes1
**Rfwd2**	n/c	2.63	2.73	35	Bag2, Birc3, Calca, Capn2, Casp8, Cdkn1a, Cybb, Eif2ak2, Eif4ebp1, Elk3, Eng, Fos, Fosl2, Gfap, H3f3b, Hmgn1, Hspb1, Icam1, Itgav, Klf4, Msn, Mybbp1a, Nedd9, Nfkbia, Pdcd6ip, Pla2g4a, Ptpn12, Rai14, Rfwd2, Rnd3, Timp1, Tnc, Tubb6, Vim, Yes1
**Rnd3**	3.85	2.77	4.06	32	Bag2, Bgn, Casp8, Ccl9, Cdkn1a, Eif4ebp1, Elk3, Erbb2ip, Fos, Gem, Gfap, H3f3b, Hmgn1, Hspb1, Icam1, Il13ra1, Il1rn, Itgav, Klf4, Msn, Mybbp1a, Nedd9, Nfkbia, Pdcd6ip, Pla2g4a, Ptpn12, Rai14, Rnd3, Timp1, Tubb6, Vim, Wwtr1
**S1pr3**	4.81	4.69	7.31	17	Cdkn1a, Edg3, Eif4ebp1, Elk3, Fos, Gfap, H3f3b, Hmgn1, Klf4, Msn, Mybbp1a, Nedd9, Nfkbia, Pla2g4a, Rai14, Rnd3, Vim
**Spp1**	1.22	2.25	6.69	26	Bag2, Calca, Capn2, Eif4ebp1, Elk3, Fos, H3f3b, Hmgn1, Hspb1, Icam1, Il1rn, Itgav, Klf4, Msn, Mybbp1a, Myd88, Nedd9, Nfkbia, Pla2g4a, Ptpn12, Rai14, Spp1, Tgm2, Timp1, Tubb6, Vim
**Tgm2**	2.65	5.63	4.98	41	Arf6, Bag2, Birc3, Capn2, Ccl9, Cdkn1a, Cyr61, Eif2ak2, Eif4ebp1, Elk3, Eng, Fcgr2b, Fos, Gfap, H3f3b, Hbegf, Hmgn1, Hspb1, Icam1, Itgav, Klf4, Ltbp1, Mybbp1a, Nedd9, Nfkbia, Odc1, Pdcd6ip, Pla2g4a, Plek, Ptpn12, Rai14, S100a10, Sdc1, Spp1, Tgm2, Timp1, Tnc, Tubb6, Vim, Wwtr1, Yes1
**Vim**	2.79	3.65	5.47	15	Capn2, Cdkn1a, Eif4ebp1, Elk3, Fos, H3f3b, Hmgn1, Klf4, Mybbp1a, Nedd9, Nfkbia, Pla2g4a, Ptpn12, Rai14, Vim

**Yes1**	n/c	2.13	3.82	41	Arf6, Arpp21, Birc3, C3ar1, Calca, Capn2, Casp8, Ccl9, Cdkn1a, Cybb, Eif2ak2, Eif4ebp1, Elk3, Eng, Fcgr2b, Fos, Gfap, H3f3b, Hbegf, Hmgn1, Hspb1, Icam1, Il1rn, Itgav, Klf4, Msn, Mybbp1a, Nedd9, Nfkbia, Pdcd6ip, Pla2g4a, Ptpn12, Rai14, Rnd3, S100a10, Timp1, Tnc, Tubb6, Vim, Wwtr1, Yes1
