# Supplementary material for: Glial responses during epileptogenesis in Mus musculus point to potential therapeutic targets
Source: PLoS One. 2018 Aug 16;13(8):e0201742. doi: 10.1371/journal.pone.0201742 (PMC6095496; doi:10.1371/journal.pone.0201742)
Supplement: S15 Table — The microRNAs are sorted by descending number of target genes in the dataset. (PDF) [file pone.0201742.s019.pdf]

Table S15: Experimentally validated mRNA-microRNA interactions for the underexpressed genes at 24h, according to miRWalk search tool. The microRNAs are sorted by descending number of target genes in the dataset.

microRNA	Number of target genes	Target genes
mmu-miR-466	10	Akap5, Ccdc85a, Dnm3, Egfl6, Epha4, Gria3, Hpca, Neurod6, Scn2a1, Wfs1
mmu-miR-297	8	Cpne9, Dnm3, Egfl6, Kcnab1, Rasgrp1, Scn2a1, Scn8a, Serpini1
mmu-let-7b	6	Dnm3, Epha4, Gria3, Scn8a, Tbc1d14, Tub
mmu-miR-17	6	Mgll, Scn2a1, Scn3b, Scn8a, St6galnac5, Wfs1
mmu-miR-223	6	Cblb, Dnm3, Gcc2, Gria3, Scn8a, Tbc1d14
mmu-miR-301b	6	Cblb, Fkbp1b, Fstl5, Hpca, Mgll, Scn3b
mmu-miR-15	5	3632451O06Rik, Bcl11b, Mgll, Scn2a1, Scn8a
mmu-miR-181a	5	Epha4, Gria3, Sept6, Tub, Zdhhc17
mmu-miR-19b	5	Cblb, Fkbp1b, Hpca, Rasgrp1, Tub
mmu-miR-9	5	Gucy1b3, Rasgrp1, Scn3b, Scn8a, Serpini1
mmu-miR-129	4	Bcl11b, Kcnma1, Mgll, Scn8a
mmu-miR-149	4	Epha4, Fkbp1b, Gria3, Scn8a
mmu-miR-24	4	Fkbp1b, Gria3, Ndrg3, Scn2a1
mmu-miR-26a	4	Fkbp1b, Gpam, Lrrtm1, Scn2a1
mmu-miR-30e	4	Epha4, Rps6ka5, Scn2a1, Scn8a
mmu-miR-425	4	Fkbp1b, Scn3b, Scn8a, Serpini1
mmu-miR-669	4	Dnm3, Gng2, Gria3, Scn2a1
mmu-miR-1187	3	Ccdc85a, Egfl6, Scn2a1
mmu-miR-124	3	3632451O06Rik, Lrrn2, Sept6
mmu-miR-128	3	Hpca, Neurod6, Wfs1
mmu-miR-27	3	Cblb, Epha4, Neurod6
mmu-miR-3087	3	Ccdc85a, Gng2, Rasgrp1
mmu-miR-324	3	Dkk3, Gpam, Scn3b
mmu-miR-329	3	Akap5, Epha4, Wfs1
mmu-miR-34b	3	Rasgrp1, Scn2a1, Zdhhc17
mmu-miR-362	3	Akap5, Epha4, Wfs1
mmu-miR-1195	2	Akap5, Gng2
mmu-miR-1198	2	Ccdc85a, Egfl6
mmu-miR-139	2	Akap5, Gng2
mmu-miR-1894	2	Akap5, Cblb
mmu-miR-1912	2	Gng2, Htr1a
mmu-miR-3089	2	Akap5, Gng2
mmu-miR-467	2	Dnm3, Scn2a1
mmu-miR-511	2	Ccdc85a, Egfl6
mmu-miR-5135	2	Akap5, Gng2
mmu-miR-541	2	Kcnma1, Scn2a1
mmu-miR-665	2	Akap5, Gng2
mmu-miR-673	2	Cblb, Epha4
mmu-miR-693	2	Cpne9, Gng2
mmu-miR-694	2	Epha4, Gng2
mmu-miR-804	2	Akap5, Cpne9
