# Supplementary material for: Glial responses during epileptogenesis in Mus musculus point to potential therapeutic targets
Source: PLoS One. 2018 Aug 16;13(8):e0201742. doi: 10.1371/journal.pone.0201742 (PMC6095496; doi:10.1371/journal.pone.0201742)
Supplement: S16 Table — The microRNAs are sorted by descending number of target genes in the dataset. (PDF) [file pone.0201742.s020.pdf]

Table S16: Experimentally validated mRNA-microRNA interactions for the overexpressed genes at 24h, according to miRWalk search tool. The microRNAs are sorted by descending number of target genes in the dataset.

miRNA	Number of target genes	Gene Symbols
mmu-miR-466	22	Adamts9, Atp8b1, Casp8, Ell2, Etv3, Fblim1, Fosl2, Hivep3, Id2, Igfbp3, Maff, Msr1, Nfkbia, Osgin2, Rwdd4a, Scg2, Shroom3, Slc6a8, Slc7a1, Srxn1, Stard8, Tll1
mmu-miR-340	16	Aff1, Dyrk3, Edem1, Fhod3, Grem2, Hbegf, Id2, Igfbp3, Mbd2, Mcl1, Med13, Slc25a24, Slc7a1, Spp1, Tgif1, Yes1
mmu-miR-669	16	Adamts9, Atp8b1, C3ar1, Cdh4, Chml, Efna5, Ell2, Etv3, Hivep3, Hspa1b, Id2, Mcl1, Nfkbia, Slc6a8, Stard8, Tll1
mmu-miR-124	14	Amotl1, Arpc1b, Dclk1, Ebna1bp2, Icam1, Litaf, Myd88, Palld, Ptpn12, Rai14, S100a10, Spp1, Svil, Zfp536
mmu-miR-297	12	Adam12, Atp8b1, Capn2, Casp8, Elk3, Ell2, Etv3, Fblim1, Nfkbia, Stard8, Uck2, Wwtr1
mmu-miR-362	11	Adamts9, Amotl1, Fosl2, Hivep3, Id2, Msr1, Pcdh8, Rwdd4a, Scg2, Slc6a8, Tll1
mmu-miR-9	11	Adamts9, Antxr2, Arpc1b, Arpp21, Eaf1, Gfap, Gramd1b, Pcdh8, Prkar2a, Rnf128, Zfp275
mmu-miR-19b	10	Gramd1b, Igfbp3, Pak3, Papss2, Pros1, Rassf8, Rnf128, Slc6a8, Tgif1, Zfp275
mmu-miR-15a	9	Dclk1, Etv3, Grem2, Igfbp3, Itgav, Prkar2a, Smyd4, Tulp4, Zfp275
mmu-miR-129	9	Casp8, Cdh4, Cybb, Efna5, Il11, Med13, Parp3, Smyd4, Zfp568
mmu-miR-329	9	Adamts9, Amotl1, Fosl2, Hivep3, Msr1, Rwdd4a, Scg2, Slc6a8, Tll1
mmu-miR-467	9	Adamts9, Atp8b1, Casp8, Ell2, Hivep3, Id2, Msr1, Nfkbia, Stard8
mmu-miR-7b	9	Ahnak, Arpp21, Bag3, Dclk1, Fgfr1op, Fos, Gramd1b, Mbd2, Ptgfrn
mmu-miR-181a	8	Adamts9, Cald1, Dclk1, Eif2ak2, Id2, Mcl1, Spp1, Zfp568
mmu-miR-149	8	Edem1, Efna5, Gramd1b, Ptgfrn, Ptpn12, Slc6a8, Slc7a1, Wwtr1
mmu-miR-30	8	Arid5b, Mcl1, Osmr, Ppfibp1, Ptgfrn, Sap30, Serpine1, Slc7a1
mmu-miR-223	7	Antxr2, Inpp5f, Mt2, Rgs2, Syt4, Ubfd1, Vim
mmu-miR-301b	7	Cald1, Grem2, Papss2, Pros1, Pvr, Rnf128, Zfp275
mmu-miR-34b	7	Cald1, Dclk1, Edem1, Igfbp3, Pcdh8, Rnf128, Syt4
mmu-miR-17	6	Cald1, Capn2, Gng4, Mcl1, Pvr, Timp1
mmu-miR-1a	5	Casp8, Ctla2a, Frmd6, Hspa1b, Klf4
mmu-let-7b	5	Arpp21, Cald1, Gramd1b, Slc6a8, Thbs1
mmu-miR-324	5	Arpp21, Etv3, Gfap, Wwtr1, Zmiz1

mmu-miR-344	5	Etv3, Id2, Igfbp3, Mcl1, Wwtr1
mmu-miR-425	5	Cald1, Grem2, Mcl1, Prkar2a, Tnc
mmu-miR-205	4	Efna5, Eif2ak2, Fgl2, Lrrk2
mmu-miR-10	4	Ccl9, Cdkn1a, Klf4, Plek
mmu-miR-3470	4	Il11, Serpine1, Smyd4, Srgn
mmu-miR-410	4	Etv3, Id2, Igfbp3, Wwtr1
mmu-miR-5101	4	Cdh4, Efna5, Med13, Slc7a1
mmu-miR-541	4	Arpp21, Efna5, Sh2d5, Zmiz1
mmu-miR-1896	3	Casp8, Il11, Smyd4
mmu-miR-125b-2	3	Antxr2, Il1rn, Serpine1
mmu-miR-1198	3	Casp8, Ell2, Fblim1
mmu-miR-1192	3	Ccl9, Chml, Plek
mmu-miR-1187	3	Casp8, Ell2, Fblim1
mmu-miR-26a	3	Eaf1, Slc7a1, Wwtr1
mmu-miR-24	3	Hbegf, Slc7a1, Srxn1
mmu-miR-224	3	Npas4, Ptx3, Serpine1
mmu-miR-27	3	Ccl9, Odc1, Spp1
mmu-miR-3064	3	Ebna1bp2, Il11, Smyd4
mmu-miR-302	3	Cdh4, Cdkn1a, Zfp568
mmu-miR-3089	3	Chml, Ebna1bp2, Srxn1
mmu-miR-3108	3	Ctla2a, Eif2ak2, Zfp568
mmu-miR-345	3	Atp8b1, Eaf1, Zmiz1
mmu-miR-335	3	Med13, Smyd4, Zfp568
mmu-miR-3473	3	Ccl9, Edem1, Riok2
mmu-miR-377	3	Adamts9, Igfbp3, Spp1
mmu-miR-450b	3	Aff1, Cdh4, Ctla2a
mmu-miR-511	3	Casp8, Ell2, Fblim1
mmu-miR-495	3	Ccl9, Chml, Plek
mmu-miR-665	3	Ccl9, Ebna1bp2, Srxn1
mmu-miR-758	3	Ptgfrn, Syt4, Tnc
mmu-miR-204	2	Efna5, Fgl2
mmu-miR-1953	2	Hn1l, Zmiz1
mmu-miR-194	2	Eif2ak2, Scg2
mmu-miR-1930	2	Ctla2a, Ebna1bp2
mmu-miR-155	2	Etv3, Fos
mmu-miR-150	2	Myd88, Zfp568
mmu-miR-145a	2	Klf4, Lamp2
mmu-miR-136	2	Ctla2a, Prkar2a
mmu-miR-1251	2	Ctla2a, Mcl1
mmu-miR-1195	2	Ebna1bp2, Srxn1
mmu-miR-208b	2	Frmd6, Mcl1
mmu-miR-212	2	Cdh4, Ctla2a
mmu-miR-211	2	Efna5 Fgl2
mmu-miR-294	2	Cdkn1a, Zfp568
mmu-miR-291	2	Cdkn1a, Zfp568
mmu-miR-295	2	Cdkn1a, Zfp568
mmu-miR-3065	2	Prkar2a, Spp1

mmu-miR-296	2	Ctla2a, Slc7a1
mmu-miR-3087	2	Il11, Smyd4
mmu-miR-3085	2	Il11, Smyd4
mmu-miR-3095	2	Casp8, Frmd6
mmu-miR-320	2	Fosl2, Serpine1
mmu-miR-342	2	Aff1, Igfbp3
mmu-miR-339	2	Ccl9, Plek
mmu-miR-351	2	Antxr2, Il1rn
mmu-miR-448	2	Lamp2, Tll1
mmu-miR-5125	2	Il11, Zfp568
mmu-miR-590	2	Efna5, Parp3
mmu-miR-543	2	Efna5, Scg2
mmu-miR-5128	2	Ctla2a, Eif2ak2
mmu-miR-709	2	Chml, Parp3
mmu-miR-706	2	Ccl9, Plek
mmu-miR-692	2	Clic4, Frrs1
mmu-miR-804	2	Ebna1bp2, Srxn1
mmu-miR-761	2	Ccl9, Itgav
mmu-miR-875	2	Rwdd4a, Serpine1
