# Supplementary material for: Glial responses during epileptogenesis in Mus musculus point to potential therapeutic targets
Source: PLoS One. 2018 Aug 16;13(8):e0201742. doi: 10.1371/journal.pone.0201742 (PMC6095496; doi:10.1371/journal.pone.0201742)
Supplement: S17 Table — (PDF) [file pone.0201742.s021.pdf]

Table S17: Primer sequences used for the evaluation of gene expression by qRT-PCR analysis.

Gene Symbol	Forward primer Sequence (5'->3')	Reverse primer Sequence (5'->3')
Bdnf	CAAAAATTACCTGGATGCCG	ACTGCTTCAGTTGGCCTTTG
Casp8	GTTGCCACCTTCAGTTTTGG	CCTTCATTTTTCGGAGTTGG
Cav1	GAAACCTCCTCAGAGCCTGC	GTGTGCGCGTCATACACTTG
Cybb	AACCTTTGCTAACATGGGGAA	CCAGAGCCAGTGCTGACC
Fos	CCTGAAGAGGAAGAGAAACGG	TGGGCTGCCAAAATAAACTCC
Gapdh	CACGTGGAGATGGATGTG	CGGCGATAGTCGTTAGCT
Hspa1b	CTTGGGCACCGATTACTGTC	CATAATCCCCTGGTACAGTGC
Il6	TCTCCACGGTCTGTTCGGGCG	AGGTCTGCCCTTTCTCCCCTTCTT
Nos3	GGCAGCATCACCTACGACA	GAGCCACTCCTTTTGATGGA
Nrif/Zfp110	GGCAGCACCCATCTTCTGT	AGACTCATGGGGCCAGGTT
Socs3	GATTTCGCTTCGGGACTAGG	GACGGGAATCAGGGATCTTC
Tnfrs1a	CCGTGACAATCCCCTGTAAG	GATAACCAGGGGCAACAGC
